# Co‐morbidity between mood and anxiety disorders: A systematic review and meta‐analysis

**DOI:** 10.1002/da.23113

**Published:** 2020-11-22

**Authors:** Sukanta Saha, Carmen C. W. Lim, Danielle L. Cannon, Lucinda Burton, Monique Bremner, Peter Cosgrove, Yan Huo, John J. McGrath

**Affiliations:** ^1^ Queensland Brain Institute University of Queensland St Lucia Australia; ^2^ Queensland Centre for Mental Health Research The Park Centre for Mental Health Wacol Australia; ^3^ Business School, Faculty of Business, Economics and Law University of Queensland St Lucia Australia; ^4^ National Centre for Register‐based Research Aarhus University Aarhus Denmark

**Keywords:** agoraphobia, anxiety disorders, bipolar disorder, depression, epidemiology, mood disorder

## Abstract

There is consistent evidence that mood disorders often co‐occur with anxiety disorders, however, the strength of the association of these two broad groups of disorders has been challenging to summarize across different studies. The aim was to conduct a meta‐analysis of publications reporting on the pairwise comorbidity between mood and anxiety disorders after sorting into comparable study types. We searched MEDLINE, Embase, CINAHL, Web of Science, and the grey literature for publications between 1980 and 2017 regardless of geographical locations and languages. We meta‐analyzed estimates from original articles after sorting by: (a) broad or narrow diagnostic criteria, (b) study time‐frame, and (c) estimates with or without covariate adjustments. Over 43 000 unique studies were identified through electronic searches, of which 391 were selected for full‐text review. Finally, 171 studies were eligible for inclusion, including 53 articles from additional snowball searching. In general, regardless of variations in diagnosis type, study time‐frame, temporal order, or use of adjustments, there was substantial comorbidity between mood and anxiety disorders. Based on the entire 90 separate meta‐analyses, the median OR was 6.1 (range 1.5–18.7). Of these estimates, all 90 were above 1, and 87 were significantly greater than 1 (i.e., the 95% confidence intervals did not include 1). Fourteen of the 90 pooled estimates had ORs that were greater than 10. This systematic review found robust and consistent evidence of comorbidity between broadly defined mood and anxiety disorders. Clinicians should be vigilant for the prompt identification and treatment of this common type of comorbidity.

## 1 INTRODUCTION

It is widely recognized that mood and anxiety disorders frequently co‐occur—the presence of one of these two disorders increases the risk of subsequently developing the other (comorbid) disorder. Comorbidity refers to the presence of one or more disorders in relation to an index disorder either within the same time period (concurrent comorbidity) or across different phases of the life span (cumulative comorbidity) (van den Akker et al., 1996). The presence of comorbidity between these two disorders is important because anxiety and depressive disorders independently contribute to a significant portion of the global burden of disease, with depression being the second largest contributor to years lived with disability in those 15–44 years of age (Ferrari et al., 2013). In addition, comorbidity between mood and anxiety disorders is associated with greater symptom severity (Bernstein, 1991; Coryell et al., 1988), increased substance use, and suicidal risk (Lewinsohn, Gotlib, et al., 1995; Lewinsohn, Rohde, et al., 1995; Rohde et al., 2001), and treatment resistance compared with those who have either disease in isolation (Brent et al., 1998; Emslie et al., 1998; Lewinsohn, Gotlib, et al., 1995; Melton et al., 2016).

Based on recent large studies, both the concurrent and cumulative risk of comorbidity between anxiety and mood disorders is substantial and bidirectional (Moffitt et al., 2007; Plana‐Ripoll et al., 2019), and exists in individuals of all ages. For example, a birth cohort study from Dunedin found that among those aged 11–32 years, anxiety concurrently occurs in 37% of depression cases, while depression occurs in 32% of anxiety cases (Moffitt et al., 2007). Cumulatively, 72% of lifetime anxiety cases had a history of depression, and 48% of lifetime depression cases had anxiety disorders (odds ratio [OR] between 4.7 and 5.5). A recent large Danish register‐based study (*n* = 5 940 778) found that those who developed a mood disorder by age 20 had a four‐fold risk of subsequently developing an anxiety disorder (OR = 4.3; 95% CI = 4.1–4.4) (Plana‐Ripoll et al., 2019). An analysis of community‐based surveys from 27 countries also showed a very high rate of comorbidity between mood and anxiety disorders; the risk of developing generalized anxiety disorder was about seven times in those with major depression even after 15 years (hazard ratio = 6.6; 95% CI = 5.7–7.7) (McGrath et al., 2020).

Previous systematic reviews have provided pooled estimates for selected types of comorbid mood and anxiety disorders (Lemstra et al., 2008; Nabavi et al., 2015; Pavlova et al., 2015; Rytwinski et al., 2013; Zavaglia & Bergeron, 2017). However, the pooling of estimates related to comorbidity can be challenging because of the heterogeneity in the study design and between‐site variations in the prevalence of the underlying disorders. Estimates of pair‐wise comorbidity can be based on: (a) broad or narrow diagnostic criteria (e.g., any type of depression‐related disorder, specific depression‐related disorder); (b) different time periods and temporal framework (e.g., cumulative comorbidity over one year or a lifetime regardless of the temporal order of the two disorders; estimates based on temporally ordered disorders with prior depression leading to subsequent anxiety or vice versa); and (c) the presentation of unadjusted risk estimates or estimates adjusted for a range of covariates (e.g., sex, age, etc.). There is a need for a more comprehensive systematic review and meta‐analysis that (a) takes into account a comprehensive range of mood and anxiety‐related disorders, and (b) provides pooled estimates based on features related to the period of observation (e.g., last 12 months, lifetime prevalence), and adjustments of covariates, and temporally ordering between disorder pairs.

The aims of the current systematic review are to provide an up‐to‐date list of studies that have examined comorbidity between broadly defined mood‐ and anxiety‐related disorders and to meta‐analyze the risk estimates according to key design features related to the types of prevalence estimates for the two disorders.

## 2 METHODS

### 2.1 Search strategy and identification of studies

Based on a protocol registered with PROSPERO (Saha et al., 2019), this systematic review is PRISMA (Preferred Reporting Items for Systematic Reviews and Meta‐Analyses) (Moher et al., 2015; Shamseer et al., 2015), and MOOSE (Meta‐analysis of Observational Studies in Epidemiology) (Stroup et al., 2000) compliant. A comprehensive search strategy was developed for identifying research publications on comorbidity of mood and anxiety disorders. In the absence of optimized search filters for bibliographic search (Waffenschmidt et al., 2017), to design this search algorithm with optimal sensitivity and specificity, different tests were performed to optimize the best combination of terms in different databases. Detailed search strategies are found in the Supporting Information eMethods 1‐2. A panel of experts (JM, SS) independently validated and revised search algorithms in different databases. Studies were identified through four electronic databases namely, PubMed‐Medline, Embase, CINAHL, and Web of Science between January 1, 1980, and December 30, 2017. No restrictions were made regarding the geographical location or language of publications.

### 2.2 Screening, snowball searching, and data extraction

**Figure 1:**
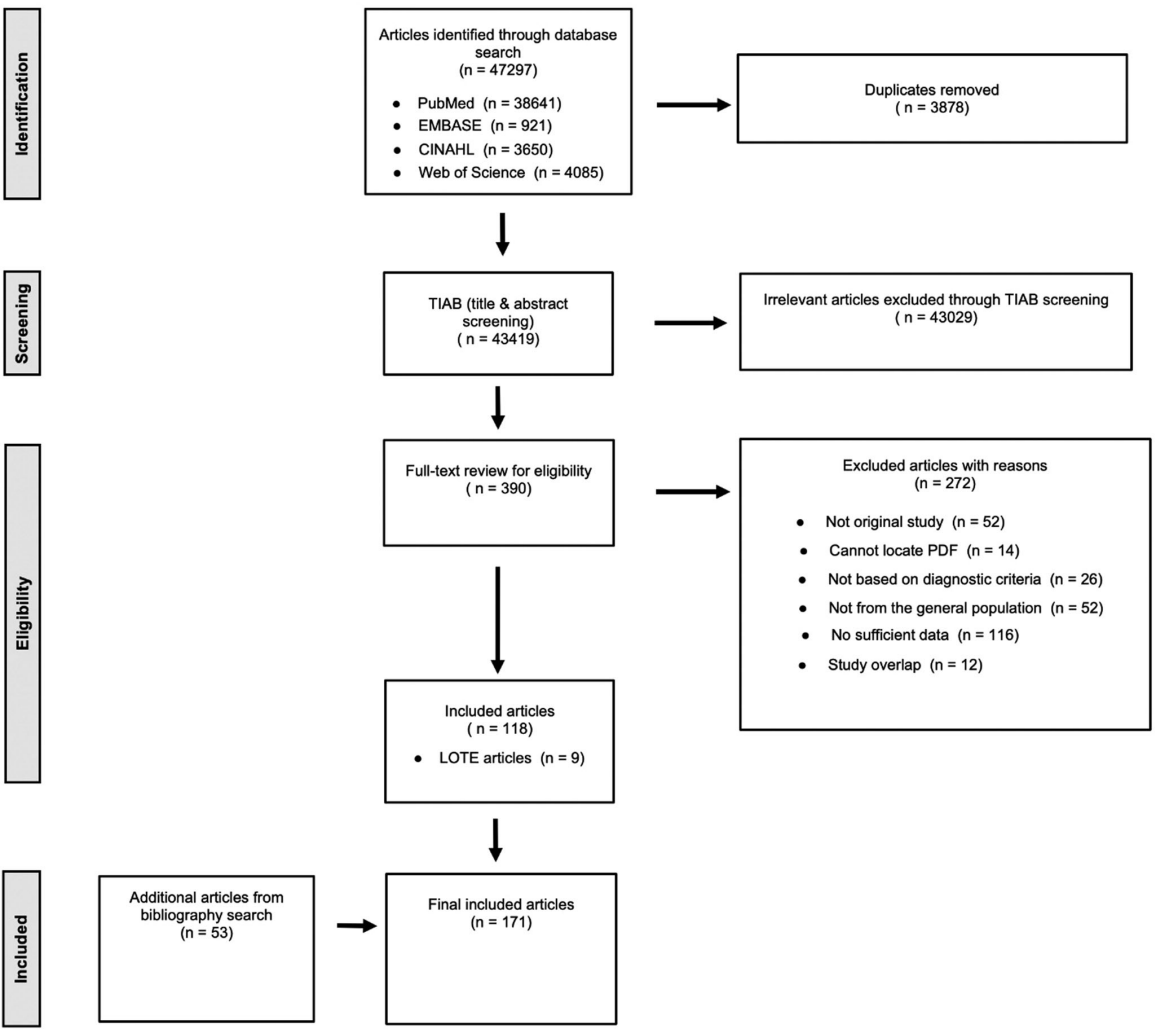
Flow diagram (selection strategy) of included studies

All potential articles from four database searches were uploaded to a commercial software, *Covidence* for the management of Title and Abstract (TIAB) screening followed by full‐text scrutiny. Any discrepancy for the screening process was resolved by the consensus of the two reviewers (PRISMA flowchart, Figure 1). Articles were screened followed by full‐text scrutiny for final inclusion into the relevant article pool. Articles with LOTE (Language Other Than English) were translated and assessed. We conducted TIAB screening based on inclusion and exclusion criteria (details in Supporting Information eMethods 2) where discrepancies were resolved by consensus among senior authors. This was followed by full‐text scrutiny for the final inclusion of relevant studies. Any discrepancy at this stage was also resolved by the consensus of the two reviewers.

We conducted snowball search for additional articles. The references cited by each relevant article, and grey literature (e.g., systematic reviews, book chaptersetc.) were searched by title, and screened followed by full‐text scrutiny for final inclusion into the relevant article pool. As stated, the final inclusion of the relevant articles from the snowball search was also accomplished by the consensus of two reviewers.

Data were extracted from the final list of the “included” articles and entered into a three‐level database. We collected information on (i) study characteristics (author, year, site etc.), (ii) methodology (sample size used, diagnostic criteria, type of mood and anxiety disorder etc.), and (iii) risk estimates (odds ratios, hazard ratios). In general, we extracted data from studies that provided: (1) both cross‐sectional and temporally ordered risk estimates (e.g., odds ratio, risk ratio), and (2) both unadjusted and adjusted estimates. We also extracted data from studies that provided sufficient information to allow the calculation of odds ratios. These estimates were considered as unadjusted estimates. For uniform presentation, we used pooled odds ratio as our primary risk measure as 95.1% of the studies reported ORs. Additional details of these steps can be found in Supporting Information eMethods 3.1.

### 2.3 Operationalized features: Diagnostic criteria, sequential filters, and quality reporting scale

In keeping with our previous systematic reviews (McGrath et al., 2004; Saha et al., 2005, 2007), and following standard systematic review guidelines (von Elm et al., 2007), we used several operationalized features. We used studies with defined diagnostic criteria (e.g., ICD, DSM etc.) for case definitions for both mood and anxiety disorders. Thus, any studies with symptom‐based disorders as well as those with an unclear methodology for any case definitions were excluded. Detailed diagnostic criteria and instruments are shown in Supporting Information eMethods 4.

The second key operationalized feature was to apply a sorting algorithm using a series of *sequential filters*. When multiple studies overlapped partially or fully in both time and space, we used a sequential filter for delineating discrete estimates. We divided the estimates into three different types (based on study types): lifetime prevalence, period prevalence, and temporally ordered estimates related to respective cross‐sectional, case‐control, or cohort studies. We also divided estimates based on adjusted or unadjusted estimates to avoid adjustment reporting bias (Peters & Mengersen, 2008). The aim was to avoid double counting of any study estimates while sorting estimates into meaningful groups to minimize possible biases.

Next, for identifying discrete data from overlapping studies, we used two types of filters, a “study‐level filter” and an “estimate‐level filter”; the former delineated overlapping studies while the latter parsed overlapping estimates between studies (details are in Supporting Information eMethods 3.2 and assessments are in Supporting Information eMethods 5). Using the study‐level filter we were able to exclude 43 papers that were completely overlapping by time and place.

To assess the overall quality of the study estimates, we employed a “Quality Reporting Scale” (QRS). The scale contains 14 criteria based on features that were operationalized, and were based on simple, categorical judgments (criteria met vs. not met) (McGrath et al., 2004; Saha et al., 2005, 2007). Detailed criteria and scales are in Supporting Information eMethods 6.

We found that a variety of labels were used for mood‐and anxiety‐related disorders. For computational tractability, we combined similar terms into a “broad” category. For example, we used “broadly defined mood disorder” (henceforth “MOOD”) for any mood disorders. Similarly, we used “broadly defined depressive disorder” (henceforth “DEP”) for depressive disorders, “broadly defined dysthymic disorder” (henceforth “DYS”) for dysthymic disorders, and “broadly defined anxiety disorders” (henceforth “ANX”) for anxiety disorders. Details of these categories with subtypes are available in Supporting Information eMethods 7. We used four broad mood disorders (MOOD, DEP, DYS, BIPOLAR) with seven subtypes of ANX pairs as follows: generalized anxiety disorder (GAD), obsessive‐compulsive disorder (OCD), posttraumatic stress disorder (PTSD), social phobia, specific phobia, panic, and agoraphobia.

### 2.4 Data analysis and presentation

We reported pooled risk estimates (i.e., odds ratio) using inverse‐variance random‐effects models. Based on expected differences in the prevalence of anxiety‐ and mood‐related disorders between the included studies, we predicted considerable heterogeneity between studies. To quantify this, we used the Q‐statistic which is sensitive to the number of studies especially when the number of studies is small (DerSimonian & Laird, 1986, 2015). We have also included I^2^, a measure of the proportion of total variation in estimates that is due to heterogeneity with values 25%, 50%, and 75% corresponding to low, moderate, and a high degree of heterogeneity, respectively. For disorder pairs with a sufficient number of studies (>10), we visually inspected the funnel plot and used Egger's test for the possibility of publication bias (Egger et al., 1997).

Data were presented in both tabular and graphical format (e.g., forest plots, funnel plots). To avoid bias in meta‐analysis, we presented pooled estimates separately for crude (unadjusted) and adjusted models (Peters & Mengersen, 2008), and for a lifetime, period prevalence, and temporally ordered estimates. Period prevalence estimates included any estimates above 1‐month (1, 3, 6, and 12 months). In the temporally ordered estimates, we present pairwise risk estimates for both directions.

The “metafor” package in R version 3.6.2 was used to produce pooled estimates, forest and funnel plots.  ORs from each study were transformed using natural logarithm, and standard errors (SE) were calculated from the reported confidence intervals (CIs). In studies with missing CIs, reported p‐values were used to calculate SEs. Comorbidity between pairwise disorders was presented as pooled risk estimates using crude OR or adjusted OR (aOR) with not less than three studies for disorder pairs.

## 3 RESULTS

The results of the detailed search strategy are shown in Supporting Information eMethods 1‐2 and Figure 1. We identified 43 419 studies from four electronic databases, of which 390 were screened for full‐text reviews including nine LOTE papers. More than half (*n* = 272) were excluded for various reasons (Figure 1). We also found 53 papers from citation search of “included” papers and grey literature. Consequently, 171 studies were included in our quantitative synthesis drawn from 37 countries (efigure 1). Using 56 pairs of mood and anxiety disorders, 90 estimates were investigated in the meta‐analysis including 76 unidirectional lifetime and period prevalence risk estimates and 14 temporally ordered bidirectional estimates.

Study characteristics of the 171 studies are presented in Table 1 and etable 1. Table 1 includes 147 studies (eReference 1) that presented lifetime and period prevalence estimates whereas eTable 1 includes 36 studies (eReference 2) that presented temporally ordered estimates.

**Table 1:** Study characteristics of included studies that provided lifetime and period prevalence (*n *= 146)

First author, year, country	Sample size	Study design	Mood disorder type^a^	Anxiety disorder type^a^	Diagnostic criteria	Risk estimate type	Control variables	Quality reporting score
Adam, 2012, Germany	4181	CS	DEP, DYS, BIPOLAR, MOOD	OCD	DSM‐IV	aOR	Age, sex	15
Alonso, 2004, Multiple countries	21 425	CS	DEP, DYS	GAD, PTSD, SO, SP, AGO, PD	DSM‐IV	OR	‐	15
Alvarenga, 2016, Brazil	2512	CS	DEP, BIPOLAR, MOOD	OCD	DSM‐IV	OR^b^	‐	12
Andrews, 2002, Australia	10 641	CS	DEP, DYS	GAD, OCD, PTSD, PD, AGO, SO	DSM‐IV	OR, aOR	Mental disorders	14
Angst, 2005, Switzerland	591	PC	DEP, BIPOLAR	OCD	DSM‐IV	aOR	Sex	12
Arillo Crespo, 1998, Spain	237	CS	DEP	SP, AGO, SO, PTSD, PD, ANX	DSM‐III‐R	OR	‐	11
Autonell, 2007, Spain	5473	CS	DYS	GAD, PTSD, AGO, SO, SP	DSM‐IV	aOR	Sociodemographic variables	14
Beekman, 2000, the Netherlands	3056	PC	DEP	ANX	DSM‐III	OR^b^	‐	12
Beesdo, 2007, Germany^c^	3021	PC	MOOD	SO	DSM‐IV	OR	‐	15
Biederman, 2005, USA	1031	CC	DEP	PD	DSM‐III‐R	OR^b^	‐	11
Blanco, 2017, USA	36 309	PC	BIPOLAR	AGO, PTSD	DSM‐5	aOR	Sex, age, race, marital status, education, household income, urbanicity, region, and mental disorders	16
Boyd, 1984, USA	11 519	CS	DEP	PD, AGO, SP, OCD	DSM‐III	OR	‐	11
Bromet, 2005, Ukraine	4725	CS	DEP, MOOD	GAD, PD, ANX	DSM‐IV	OR	‐	15
Bruce, 2001, USA	1492	PC	DEP, DYS	PTSD	DSM‐IV	OR^b^		10
Cairney, 2007, Canada	12 792	CS	DEP	SO	DSM‐IV	aOR	Did not specify	15
Cairney, 2008, Canada	36 984	CS	DEP	SO, ANX	DSM‐IV	OR^b^	‐	11
Carter, 2001, Germany	4181	CS	DEP, DYS, MOOD	GAD, PD, AGO, SO, SP, ANX	DSM‐IV	aOR	Age, sex	15
Cederlof, 2015, Sweden^c^	19 814	CC	BIPOLAR	OCD	ICD	RR		13
Chartier, 2003, Canada	8116	CS	DEP, DYS, BIPOLAR	SO	DSM‐III‐R	aOR	Age, sex, education	16
Chavira, 2004, USA	1173	CS	DEP	SAD, GAD, SP, SO	DSM‐IV	aOR	Sex	14
Chen, 1995, USA	18 103	CS	DEP, BIPOLAR	PD	DSM‐III	aOR	Age, sex, race, marital status, education, socioeconomic status, history of alcohol and drug abuse	14
Chen, 2017, China	512 891	CS	DEP	GAD	DSM‐IV	aOR	Education, household income, occupation, smoking, alcohol, BMI, physical activity, chronic disease, mental disorders	16
Chou, 2009, USA	13 420	PC	BIPOLAR, DYS	SO	DSM‐IV	aOR	Age, sex, race, marital status, education, employment status, personal income, psychosocial risk factor, childhood experience, childlessness, foreign‐born, living alone, number of stressful life events, comorbid disorders	15
Chou, 2009, USA	34 653	PC	DYS, BIPOLAR	SP	DSM‐IV	aOR	Demographic variables, psychiatric disorders	15
Chou, 2010, USA	13 420	PC	DYS	PD	DSM‐IV	aOR	Age, sex, race, marital status, education, employment status, personal income, psychosocial risk factor, number of stressful life events, comorbid disorders	15
Choy, 2007, USA	5877	CS	DEP	SP	DSM‐III‐R	OR, aOR	Age, sex, race, marital status, income, education, mental disorders	14
Chuan, 2008, Singapore	1092	CS	DEP	ANX	DSM‐IV	OR, aOR	Age, sex, race, education, housing type, employment status, marital status, living arrangements	16
Copeland, 2013, USA	2967	CS, PC	DEP, DYS	GAD, SAD, SO	DSM‐IV	OR, aOR	Mental disorders	16
Corna, 2007, Canada	12 792	CS	DEP	PD	DSM‐IV	OR, aOR	Age, sex, marital status, income adequacy, education, language spoken, geographic location of residence, chronic health problems, limitations in ADLs/IADLS, mental disorders	15
Costello, 2003, USA^c^	6674	PC	MOOD	ANX	DSM‐IV	OR, aOR	Mental disorders	13
Coyne, 1994, USA	1928	CS	DEP	ANX	DSM‐IV	OR^b^	‐	10
Cyranowski, 2012, USA	939	PC	DEP	ANX	DSM‐IV	OR^b^	‐	12
Davidson, 1991, USA	4423	PC	DEP	PTSD	DSM‐III	OR	‐	10
de Graaf, 2002, the Netherlands	7076	PC	DEP, DYS, BIPOLAR	ANX	DSM‐IV	OR	‐	12
de Graaf, 2003, the Netherlands	7076	PC	DEP, DYS, BIPOLAR	PD, AGO, SP, SO, GAD, OCD	DSM‐IV	OR	‐	13
Depla, 2008, the Netherlands	7076	PC	DEP, DYS, BIPOLAR	SP	DSM‐III‐R	aOR	Age, sex, other specific fears	15
Douglass, 1995, New Zealand	1037	PC	DEP, DYS	OCD	DSM‐III‐R	OR^b^	‐	11
Essau, 1999, Germany	1035	CS	DEP	PD	DSM‐IV	OR^b^	‐	11
Essau, 2003, Germany	1444	CS	DEP, DYS	ANX	DSM‐IV	OR	‐	13
Faravelli, 2004a, Italy	2500	CS	DEP, DYS	GAD, OCD, PD, SO, SP	DSM‐IV	OR^b^	‐	9
Faravelli, 2004b, Italy	2363	CS	DEP, DYS	GAD, OCD, PD, SO, SP	DSM‐IV	OR^b^	‐	11
Fehm, 2008, Germany	4181	CS	DEP, DYS, BIPOLAR, MOOD	SO	DSM‐IV	OR	‐	13
Fineberg, 2013, Switzerland	591	PC	DEP, BIPOLAR	OCD	DSM‐III	OR	‐	13
Fleitlich‐Bilyk, 2004, Brazil	1251	CS	DEP	ANX	DSM‐IV	aOR	Mental disorders	15
Ford, 2003, Multiple countries	‐d	CS	DEP	ANX	DSM‐IV	aOR	Mental disorders	15
Fung, 2017, Hong Kong	5719	CS	DEP	ANX	ICD‐10	OR^b^	‐	11
Gabilondo, 2010, Spain	5473	CS	DEP	GAD, AGO, SP, SO, PTSD, PD, ANX	DSM‐IV	aOR	Sex, education, marital status	15
Glaesmer, 2012, Germany	5033	CS	DEP	PTSD	DSM‐IV, ICD‐10	aOR	Age, sex	15
Goncalves, 2011, Australia	8841	CS	DEP	GAD	DSM‐IV	OR, aOR	Age, sex, living arrangements, financial problems, frequency family contact, caregiver status, stressful life events, worry about illness, exercise, chronic illness, functional limitations, self‐assessed health, medication, smoking, family history of anxiety	13
Goodwin, 2004, Germany^c^	3021	PC	DEP, DYS, BIPOLAR, MOOD	PD	DSM‐IV	aOR	Age, sex	13
Grabe, 2001, Germany	4093	PC	DEP, DYS, BIPOLAR	OCD	DSM‐IV	OR	‐	14
Grant, 2005, USA	43 093	PC	BIPOLAR, DYS, MOOD	GAD	DSM‐IV	aOR	Age, race, sex, marital status, education, income, urbanicity, region of country	15
Grant, 2005, USA	43 093	PC	DYS, BIPOLAR, MOOD	SO	DSM‐IV	aOR	Age, race, marital status, education, income, urbanicity, region	15
Grant, 2005, USA	43 093	PC	BIPOLAR	PD, SO, SP, GAD, ANX	DSM‐IV	OR, aOR	Age, race, marital status, education, income, urbanicity, region	15
Gratzer, 2004, Canada	8116	CS	DEP	ANX	DSM‐III‐R	OR^b^	‐	10
Grenier, 2011, Canada	2798	CS	DEP	SP	DSM‐IV	aOR	Age, sex, socioeconomic status	16
Gureje, 2010, Nigeria	6752	CS	DEP	ANX	DSM‐IV	aOR	Sex, education, marital status	16
Hasin, 2005, USA	43 093	PC	DEP	PD, SO, SP, GAD, ANX	DSM‐IV	OR, aOR	Age, sex, race, marital status, education, income, region, urbanicity	15
Hauffa, 2011, Germany	2510	CS	DEP	PTSD	DSM‐IV	OR^b^	‐	11
He, 2009, China	8487	CS	MOOD	ANX	DSM‐IV	OR^b^	‐	10
Hecht, 1990, Germany	1366	PC	MOOD	ANX	DSM‐III	OR^b^	‐	10
Hek, 2011, the Netherlands	6007	PC	MOOD	GAD, AGO, ANX	DSM‐IV	aOR	Age, sex, education, living alone, cognitive status	16
Hunt, 2002, Australia	10 641	CS	DEP, DYS, MOOD	GAD	DSM‐IV	aOR	Mental disorders	15
Jaffee, 2002, New Zealand^c^	1037	PC	DEP	ANX	DSM	aOR	Sex	13
Jeon, 2007, South Korea	6275	CS	DEP, MOOD	PTSD	DSM‐IV	aOR	Age, sex, education	15
Kang, 2016, South Korea^c^	1204	PC	DEP	ANX	AGECAT	OR, aOR	Sex, housing, stressful life event, clinical factor	15
Kashani, 1988, USA	150	CS	DEP	ANX	DSM‐III	OR^b^	‐	10
Katona, 1997, UK	774	CS	DEP	GAD, PHOBIA	CARE	OR^b^	‐	8
Kawakami, 2004, Japan	1029	CS	MOOD	ANX	DSM‐III‐R	OR^b^	‐	15
Kessler, 1995, USA	8098	CS	DYS	PTSD	DSM‐III‐R	OR	‐	13
Kessler, 1996, USA^c^	8098	CS	DEP	GAD, AGO, SP, SO, PTSD, ANX	DSM‐III‐R	OR	‐	15
Kessler, 1997, USA	8098	CS	BIPOLAR	GAD, AGO, SP, SO, PD, PTSD, ANX	DSM‐III‐R	OR	‐	13
Kessler, 1999, USA	11 130	CS	DEP	GAD	DSM‐III‐R	OR^b^	‐	11
Kessler, 1999, USA^c^	8098	CS	BIPOLAR	SO	DSM‐III‐R	aOR	Age, sex, race, person‐year	14
Kessler, 2002a, Multiple countries^c^	20 189	CS	DEP, DYS, MOOD	GAD	DSM‐III‐R	OR	‐	15
Kessler, 2002b, USA	8098	CS	DEP	GAD	DSM‐III‐R	OR^b^	‐	11
Kim, 2016, South Korea	3013	CS	BIPOLAR	GAD, OCD, AGO, SO, SP, PTSD, PD, ANX	DSM‐IV	OR	‐	14
Kolada, 1994, Canada	3258	CS	DEP, DYS	OCD	DSM‐III	OR^b^	‐	11
Lampe, 2003, Australia	10 641	CS	DEP, DYS	SO	DSM‐IV	aOR	Mental disorders	14
Leray, 2011, France	36 015	CS	DEP	GAD, SO, PD, AGO, PTSD, ANX	ICD‐10	aOR	Socio‐demographics	15
Lewinsohn, 1993, USA	1710	PC	BIPOLAR	ANX	DSM‐III‐R	OR	‐	12
Lewinsohn, 1995, USA	1709	PC	BIPOLAR	SAD, PD, PHOBIA, ANX	DSM‐III‐R	OR^b^	‐	11
Lewinsohn, 1997, USA	1709	PC	DEP, DYS, BIPOLAR	PD, SO, SP, OCD, SAD, OVRANX, ANX	DSM‐III‐R	aOR	Sex, mental disorders	15
Lim, 2005, Singapore	2847	CS	DEP, DYS	GAD	DSM‐IV	OR^b^	‐	12
Magee, 1996, USA	8098	CS	DYS, MOOD	AGO, SP, SO	DSM‐III‐R	OR	‐	15
Magklara, 2015, Greece	5614	CS	DEP	GAD, OCD, PD, AGO, PHOBIA	ICD‐10	aOR	Age, sex	16
Martin‐Merino, 2010, Multiple countries	97 170	nested CC	DEP	ANX	READ	aOR	Age, sex, calendar year, smoking, primary care physician visits, stress, sleep and ANXs	12
McCabe, 2006, Canada	12 792	CS	DEP	AGO	DSM‐IV	OR, aOR	Did not specify	15
McEvoy, 2011, Australia	8841	CS	DEP, DYS, BIPOLAR, MOOD	ANX	DSM‐IV	OR	‐	13
Mergl, 2007, Germany	394	CS	DEP, DYS	ANX	ICD‐10	OR		10
Merikangas, 1996, Multiple countries	‐d	CS, PC	DEP	GAD, PD, AGO, SP, SO, ANX	DSM‐III, DSM‐III‐R	OR	‐	13
Merikangas, 2003, Switzerland	4547	PC	DEP	ANX	DSM	aOR	Mental disorders, sex, SCL‐90 risk group	14
Merikangas, 2011, Multiple countries	61 392	CS	BIPOLAR	GAD, PTSD, OCD, AGO, PD, SP, SO, SAD, ANX	DSM‐IV	aOR	Age, age‐squared, country	16
Mitchell, 2004, Australia	10 641	CS	BIPOLAR	GAD, OCD, PTSD, PD, AGO, SO	DSM‐IV	aOR	Mental disorders	15
Moffitt, 2007, New Zealand	1037	PC	DEP	GAD	DSM	OR		13
Mohammadi, 2006, Iran	25 180	CS	DEP, BIPOLAR	SO	DSM‐IV	OR	‐	13
Mohammadi, 2007, Iran	25 180	CS	DEP, BIPOLAR	OCD	DSM‐IV	OR	‐	10
Munyandamutsa, 2012, Rwanda	1000	CS	DEP	PTSD	DSM‐IV	OR	‐	15
Murphy, 2004, Canada	1201	PC	DEP	ANX	DSM‐III‐R	OR^b^		11
Newman, 1996, New Zealand	1037	PC	MOOD	ANX	DSM‐III‐R	OR^b^	‐	10
Ohayon, 2000, Canada	2516	CS	DEP, BIPOLAR	GAD, AGO, PD, SO, SP, OCD, PTSD	DSM‐IV	OR^b^	‐	11
Ohayon, 2010, Multiple countries	18 980	CS	DEP	GAD, OCD, PTSD, AGO, PD, SP	DSM‐IV	OR	‐	10
Pakriev, 1998, Russia	855	CS	DEP	SO, SP	ICD‐10	OR	‐	12
Pietrzak, 2012, USA	34 653	PC	DEP, DYS, BIPOLAR, MOOD	PTSD	DSM‐IV	aOR	Sociodemographic variables, mental disorders	16
Pirkola, 2005, Finland	6038	CS	DEP	ANX	DSM‐IV	OR^b^	‐	13
Preville, 2008, Canada	2798	CS	DEP	GAD, AGO	DSM‐IV	OR^b^	‐	12
Prina, 2011, Multiple countries	‐d	CS	DEP	ANX	ICD‐10	OR^b^	‐	13
Rihmer, 2001, Hungary	2953	CS	DEP, BIPOLAR	GAD, PD, AGO, SO, SP	DSM‐III‐R	OR^b^	‐	10
Ritchie, 2013, France^c^	1968	PC	DEP	AGO	DSM‐IV	OR^b^	‐	12
Rohde, 1991, USA	3770	PC	DEP	ANX	DSM‐III‐R	OR	‐	13
Romano, 2005, Canada	2000	PC	DEP	ANX	DSM‐III‐R	OR	‐	13
Roy‐Byrne, 2000, USA	8098	CS	DEP	PD	DSM‐III‐R	OR	‐	14
Rueda‐Jaimes, 2008, Colombia	474	CS	DEP	OCD	DSM‐IV	OR, aOR	Did not specify	15
Ruscio, 2008, USA	9282	CS	DEP, DYS, BIPOLAR, MOOD	SO	DSM‐III, DSM‐IV	aOR	Age, sex, race	15
Ruscio, 2010, USA	9282	CS	DEP, DYS, BIPOLAR, MOOD	OCD	DSM‐IV	aOR	Age, sex, race	15
Sahoo, 2010, India	500	CS	DEP	ANX	DSM‐IV, ICD‐10	OR^b^	‐	12
Sartorius, 1996, Multiple countries	5438	CS	DEP	ANX	ICD‐10	OR	‐	11
Schaffer, 2006, Canada	36 984	CS	BIPOLAR	ANX	DSM‐IV	OR	‐	14
Schaub, 2000, Germany	516	CS	DEP	ANX	DSM‐III‐R	OR	‐	12
Schneier, 1992, USA	13 537	CS	DEP, DYS, BIPOLAR	SO	DSM‐III	aOR	Age, sex, site	15
Schoevers, 2003, the Netherlands	4051	PC	DEP	GAD	GMS‐AGECAT	OR^b^	‐	11
Schrier, 2012, the Netherlands	725	CS	DEP	ANX	DSM‐IV	OR^b^	‐	12
Scott, 2006, New Zealand	12 992	CS	DEP, DYS, BIPOLAR	PD, AGO, SP, SO, GAD, PTSD, OCD	DSM‐IV	OR	‐	12
Serrano‐Blanco, 2010, Spain	3820	CS	DEP, DYS	SO, SP, AGO, PD	DSM‐IV	OR	‐	14
Sicras‐Mainar, 2008, Spain	65 767	PC	DEP	GAD	ICD‐9	aOR	Age, sex, mental disorders	14
Spaner, 1994, Canada	3258	CS	DEP	OCD, PD, PHOBIA	DSM‐III	OR^b^	‐	11
Stein, 2004, Canada	9953	CS	DEP	GAD	DSM‐III‐R	aOR	Age, sex, social class of parents, DYS	15
Stylianidis, 2014, Greece	900	CS	DEP	GAD, PD, AGO	ICD‐10	OR^b^	‐	11
Subramaniam, 2013, Singapore	6616	CS	BIPOLAR	GAD, OCD	DSM‐IV	aOR	Age, sex, race	16
Szadoczky, 2002, Hungary	2953	CS	DEP	PD, GAD, AGO, SO, SP, OCD, ANX	DSM‐III‐R	OR	‐	14
Thompson, 1989, Canada	3258	CS	DEP	AGO, PD	DSM‐III	OR	‐	13
Trumpf, 2010, Germany	1538	PC	DEP, MOOD	SP	DSM‐IV	OR, aOR	Mental disorders	15
Tsuchiya, 2009, Japan^c^	2437	CS	DEP	SO, AGO, SP	DSM‐IV	aOR	Sex, birth‐cohort, number of other ANXs, marital status, education	15
Vaiva, 2008, France	36 105	CS	DEP, DYS	PTSD	DSM‐IV	aOR	Variables that were significant in the univariate model	12
Van Ameringen, 2008, Canada	2991	CS	DEP	PTSD	DSM‐IV	OR	‐	13
van Balkom, 2000, the Netherlands	3107	PC	DEP	GAD, OCD, PD, PHOBIA	DSM‐III	OR	‐	13
van Loo, 2016, the Netherlands	167 729	PC	DEP	GAD	DSM‐IV	OR	‐	13
Weissman, 1994, Multiple countries	‐d	CS, PC	DEP	OCD	DSM‐III	OR	‐	13
Weissman, 1996, Multiple countries	‐d	CS, PC	DEP	OCD, PD	DSM‐III	aOR	Age, sex	13
Weissman, 1997, Multiple countries	1746	PC	DEP	PD	DSM‐III	aOR	Age, sex	13
Wichstrom, 2012, Norway	2475	CS	DEP	ANX	DSM‐IV	OR		14
Wittchen, 1992, Germany	1974	PC	MOOD	ANX	DSM‐III	OR^b^	‐	11
Wittchen, 1994, USA	8098	CS	DEP, DYS	GAD	DSM‐IV	OR	‐	13
Wittchen, 1999, Germany	3021	PC	DEP, DYS, MOOD	SO	DSM‐IV	aOR	Sex	14
Wittchen, 2000, Germany	4181	CS	DEP	GAD	DSM‐IV	OR^b^	‐	12
Zhang, 2015, France	2189	CS	DEP	GAD	DSM‐IV	OR, aOR	Age, sex, mental disorders	16
Zutshi, 2006, India	130	CC	BIPOLAR	GAD, OCD, SO, ANX	DSM‐IV	OR^b^	‐	10

Abbreviations: AGO, agoraphobia with or without panic disorder; ANX, broadly defined anxiety disorder; aOR, adjusted odds ratio; BIPOLAR, bipolar I, II or bipolar disorder; CC, case‐control studies; CS, cross‐sectional studies; DEP, depressive disorder; DYS, dysthymia; GAD, generalized anxiety disorder; MOOD, mood disorder; OCD, obsessive‐compulsive disorder; OR, odds ratio; OVRANX, overanxious disorder; PC, prospective cohort studies; PD, panic disorder; PHOBIA, phobia disorder; PTSD, posttraumatic stress disorder; RR, risk ratio; SAD, separation anxiety disorder; SO, social phobia or social anxiety disorder; SP, specific phobia or simple phobia.

^a^ Disorders assessed in the study. We did not extract comorbidity estimates within the same disorder class.

^b^ These are extracted from 2 × 2 table.

^c^ These studies have both temporally and non‐temporally ordered estimates.

^d^ Multiple sample sizes.

### 3.1 Pairwise association between mood and anxiety disorders

**Figure 2:**
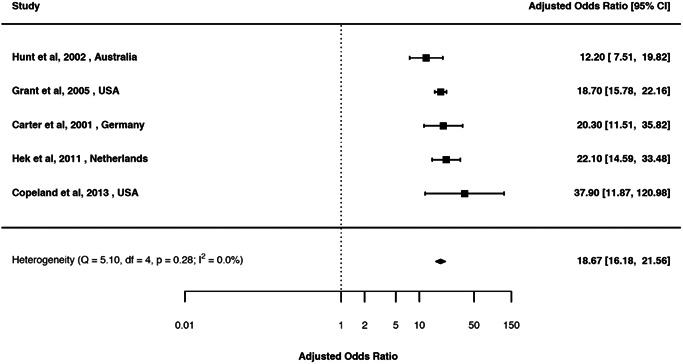
Forest plot of the random‐effects meta‐analysis of period prevalence comorbidity between broadly defined mood and generalized anxiety disorders (adjusted) (aOR 18.7; 95% CI 16.2, 21.6)

**Figure 3:**
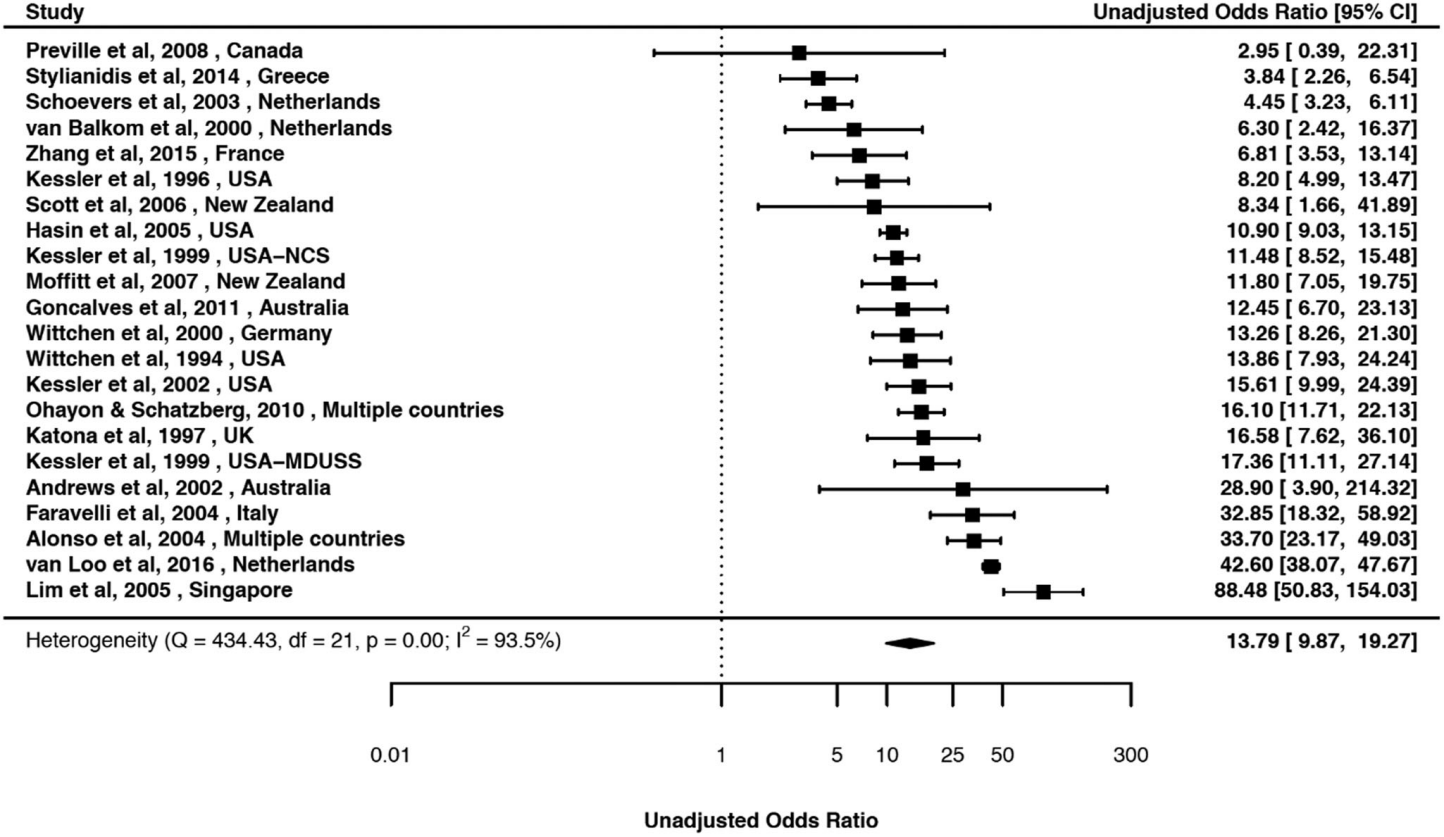
Forest plot of the random‐effects meta‐analysis of period prevalence comorbidity between broadly defined depressive disorder and generalized anxiety disorders (unadjusted) (aOR 13.8; 95% CI 9.9, 19.3)

**Figure 4:**
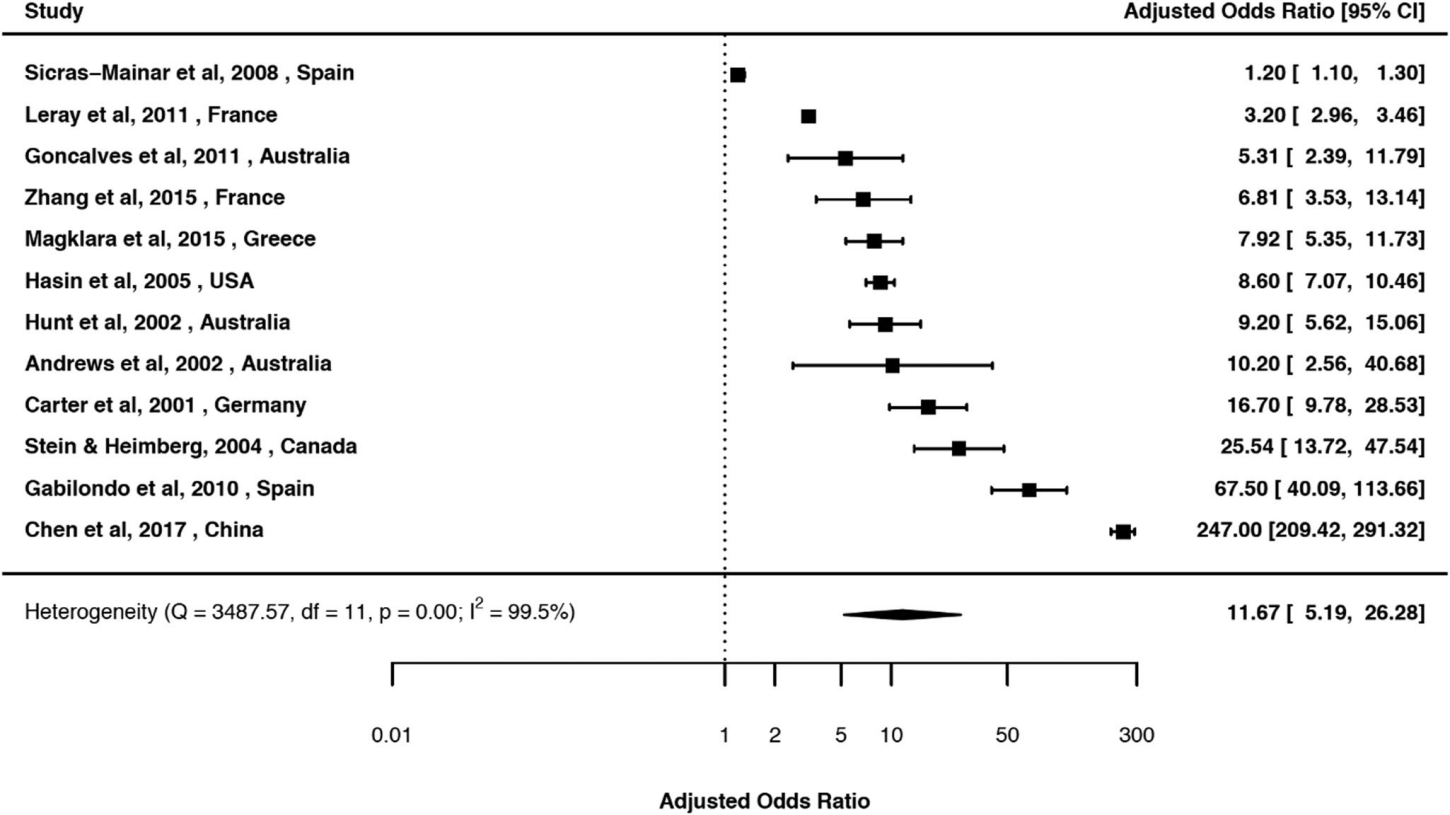
Forest plot of the random‐effects meta‐analysis of period prevalence comorbidity between broadly defined depressive disorder and generalized anxiety disorders (adjusted) (aOR 11.7; 95% CI 5.2, 26.3)

Estimates from the pairwise associations of mood and anxiety disorders are presented in Tables 2, 3, 4, 5. Forest plots and associated funnel plots are presented in efigures 2–90 (includes 14 funnel plots). Of these, we presented three forest plots in the main text (Figures 2, 3, 4). Overall, there was substantial comorbidity between various mood and anxiety disorders with a median OR of 6.1 (range 1.5–18.7). For example, those with MOOD had 19 times elevated risk of GAD (Figure 2); while those with DEP had 13.8 times elevated risk of GAD after pooling all the unadjusted estimates (Figure 3), and 11.7 times elevated risk of GAD after pooling all the adjusted estimates (Figure 4).

**Table 2:** Pooled estimates for broadly defined mood disorder (MOOD) and anxiety disorders

Broadly defined mood disorder	Anxiety disorder type	Crude estimates				Adjusted estimates			
		*n*	Pooled OR (95% CI)	*I* ^2^	*p* value	*n*	Pooled aOR (95% CI)	*I* ^2^	*p* value
I. Lifetime estimates									
MOOD	ANX	5	7.7 (3.3–17.7)	96.5%	<.001	0	‐a	‐	‐
MOOD	GAD	1	‐a	‐	‐	1	‐a	‐	‐
MOOD	Social phobia	3	2.8 (1.9–4.3)	88.8%	<.001	2	‐a	‐	‐
MOOD	Specific phobia	3	2.4 (1.1–5.2)	85.4%	<.001	1	‐a	‐	‐
II. Period prevalence estimates									
MOOD	ANX	10	13.4 (8.6–27.2)	98.3%	<.001	3	11.8 (4.6–30.1)	85.1	<.001
MOOD	GAD	2	‐a	‐	‐	5	18.7 (16.2–21.6)	0.0%	.28
MOOD	Social phobia	3	18.4 (13.3–27.2)	0.0%	.09	3	5.2 (2.9–10.0)	79.6%	.02
MOOD	Specific phobia	1	‐a	‐	‐	1	‐a	‐	‐

Abbreviations: ANX, broadly defined anxiety disorder, GAD, generalized anxiety disorder.

^a^ Estimates were not pooled if the number of studies are less than 3 for that disorder pair.

**Table 3:** Pooled estimates for broadly defined depressive disorder (DEP) and anxiety disorders

Broadly defined depressive disorder	Anxiety disorder type	Crude estimates				Adjusted estimates			
		*n*	Pooled OR (95% CI)	*I* ^2^	*p* value	*n*	Pooled aOR (95% CI)	*I* ^2^	*p* value
I. Lifetime estimates	
DEP	ANX	17	4.4 (3.1–6.2)	97.2%	<.001	3	3.2 (2.2–4.7)	97.9%	<.001
DEP	Agoraphobia	11	4.0 (3.0–5.2)	80.8%	<.001	1	‐a	‐	‐
DEP	OCD	14	5.6 (4.4–7.2)	61.3%	<.001	8	4.8 (3.8–6.2)	9.4%	.25
DEP	GAD	11	9.0 (5.6–14.5)	96.7%	<.001	4	5.8 (3.6–9.5)	62.7%	.08
DEP	Panic disorder	15	7.6 (5.2–11.1)	92.1%	<.001	11	7.4 (4.4–12.4)	87.4%	<.001
DEP	PTSD	5	6.1 (2.7–13.7)	94.5%	<.001	1	‐a	‐	‐
DEP	Social phobia	13	4.6 (3.8–5.6)	76.2%	<.001	8	3.7 (3.1–4.4)	53.4%	.06
DEP	Specific phobia	14	3.3 (2.7–4.0)	85.5%	<.001	7	2.4 (1.6–3.5)	90.4%	<.001
II. Period prevalence estimates									
DEP	ANX	23	9.4 (6.3–14.3)	96.5%	<.001	11	6.6 (4.7–9.1)	94.2%	<.001
DEP	Agoraphobia	10	6.6 (3.6–11.8)	90.7%	<.001	5	5.6 (3.3–9.4)	82.1%	<.001
DEP	OCD	10	7.7 (4.8–12.2)	65.2%	.01	5	4.4 (2.2–8.9)	74.2%	.01
DEP	GAD	22	13.8 (9.9–19.3)	93.5%	<.001	12	11.7 (5.2–26.3)	99.5%	<.001
DEP	Panic disorder	13	9.9 (6.8–14.3)	83.6%	<.001	7	6.2 (3.2–11.9)	94.7%	<.001
DEP	PTSD	6	14.7 (8.2–26.4)	86.9%	<.001	6	4.1 (1.4–11.4)	97.8%	<.001
DEP	Social phobia	9	8.8 (5.4–14.2)	90.4%	<.001	6	6.6 (9.2–13.0)	96.3%	<.001
DEP	Specific phobia	8	3.7 (2.7–5.0)	80.4%	<.001	3	2.9 (2.3–3.7)	46.3%	.16

Abbreviations: ANX, broadly defined anxiety disorder; GAD, generalized anxiety disorder; OCD, obsessive‐compulsive disorder; PTSD, posttraumatic stress disorder.

^a^ Estimates were not pooled if the number of studies are less than 3 for that disorder pair.

**Table 4:** Pooled estimates for broadly defined dysthymic disorder (DYS) and anxiety disorders

Broadly defined dysthymic disorder	Anxiety disorder type	Crude estimates				Adjusted estimates			
		*n*	Pooled OR (95% CI)	*I* ^2^	*p* value	*n*	Pooled aOR (95% CI)	*I* ^2^	*p* value
I. Lifetime estimates									
DYS	ANX	2	‐a	‐	‐	1	‐a	‐	‐
DYS	Agoraphobia	2	‐a	‐	‐	0	‐a	‐	‐
DYS	OCD	4	6.6 (3.8–11.3)	40.9%	.19	1	‐a	‐	‐
DYS	GAD	5	13.8 (12.3–15.4)	0.0%	.1	1	‐a	‐	‐
DYS	Panic disorder	2	‐a	‐	‐	2	‐a	‐	‐
DYS	PTSD	2	‐a	‐	‐	1	‐a	‐	‐
DYS	Social phobia	3	5.8 (2.9–11.6)	87.7%	<.001	5	4.1 (2.1–8.2)	94.1%	<.001
DYS	Specific phobia	3	6.8 (2.1–21.6)	97.1%	<.001	2	‐a	‐	‐
II. Period prevalence estimates									
DYS	ANX	2	‐a	‐	‐	0	‐a	‐	‐
DYS	Agoraphobia	3	7.3 (1.8–30.0)	89.3%	<.001	1	‐a	‐	‐
DYS	OCD	4	14.0 (7.3–26.7)	0.0%	.99	2	‐a	‐	‐
DYS	GAD	6	16.9 (7.0–40.8)	81.6%	<.001	5	12.3 (9.8–15.5)	0.0%	.87
DYS	Panic disorder	5	11.2 (4.6–27.6)	78.8%	<.001	2	‐a	‐	‐
DYS	PTSD	3	17.2 (10.4–28.4)	0.0%	0.59	3	2.3 (0.7–7.8)	95.5%	<.001
DYS	Social phobia	6	9.1 (4.6–18.0)	69.8%	<.001	3	3.3 (1.4–7.7)	81.1%	<.001
DYS	Specific phobia	4	6.9 (1.6–29.3)	93.0%	<.001	2	‐a	‐	‐

Abbreviations: ANX, broadly defined anxiety disorder; GAD, generalized anxiety disorder.

^a^ Estimates were not pooled if the number of studies is less than 3 for that disorder pair.

**Table 5:** Pooled estimates for broadly defined bipolar disorder (BIPOLAR) and anxiety disorder subtypes

Mood disorder (Bipolar disorder)	Anxiety disorder type	Crude estimates				Adjusted estimates			
		*n*	Pooled OR (95% CI)	*I* ^2^	*p* value	*n*	Pooled aOR (95% CI)	*I* ^2^	*p* value
I. Lifetime estimates									
BIPOLAR	ANX	8	7.8 (6.5–9.4)	33.3%	.19	3	7.7 (5.4–10.8)	75.3%	.02
BIPOLAR	Agoraphobia	4	5.9 (1.7–21.1)	86.6%	<.001	2	‐a	‐	‐
BIPOLAR	OCD	6	6.7 (1.3–35.4)	96.8%	<.001	3	8.4 (6.2–11.2)	0.0%	.77
BIPOLAR	GAD	6	6.9 (4.4–10.9)	58.7%	.02	4	8.9 (5.2–15.3)	92.7%	<.001
BIPOLAR	Panic disorder	6	5.4 (3.0–9.7)	68.5%	.02	4	7.1 (5.7–8.8)	25.4%	.21
BIPOLAR	PTSD	2	‐a	‐	‐	3	3.7 (1.6–8.5)	93.5%	<.001
BIPOLAR	Social phobia	6	4.4 (2.2–9.0)	80.7%	<.001	6	4.5 (2.7–7.6)	88.8%	<.001
BIPOLAR	Specific phobia	5	4.7 (2.6–8.5)	82.1%	.01	4	3.5 (1.9–6.6)	95.8%	<.001
II. Period prevalence estimates									
BIPOLAR	ANX	3	4.9 (2.0–‐12.2)	78.0%	.02	2	‐a	‐	‐
BIPOLAR	Agoraphobia	2	‐a	‐	‐	2	‐a	‐	‐
BIPOLAR	OCD	4	7.0 (3.0–16.1)	17.8%	.46	3	7.4 (1.6–34.0)	77.4%	.01
BIPOLAR	GAD	4	8.0 (3.7–17.3)	65.7%	.01	3	7.1 (3.4–14.8)	87.0%	<.001
BIPOLAR	Panic disorder	3	11.6 (8.9–15.0)	0.0%	.68	2	‐a	‐	‐
BIPOLAR	PTSD	2	‐a	‐	‐	2	‐a	‐	‐
BIPOLAR	Social phobia	3	7.9 (6.3–9.9)	0.0%	.64	4	3.7 (2.1–6.5)	83.5%	<.001
BIPOLAR	Specific phobia	3	4.9 (4.1–5.8)	0.0%	.56	2	‐a	‐	‐

Abbreviations: ANX, broadly defined anxiety disorder, GAD, generalized anxiety disorder; OCD, obsessive‐compulsive disorder; PTSD, posttraumatic stress disorder.

^a^ Estimates were not pooled if the number of studies are less than 3 for that disorder pair.

### 3.2 MOOD and anxiety disorder: Lifetime comorbidity

We identified nine studies (eReference 3) for lifetime comorbidity between MOOD and different anxiety disorders (Table 2). The pooled OR (95% CI) ranged between 2.4 (1.1, 5.2) for MOOD and specific phobia based on three studies, and 7.7 (3.3, 17.7) for MOOD and ANX based on five studies.

### 3.3 MOOD and anxiety disorder: Period prevalence comorbidity

We identified 20 studies (eReference 4) for period prevalence comorbidity between MOOD and anxiety disorders (Table 2). The crude OR (95% CI) ranged between 9.1 (3.9, 21.3) for MOOD and ANX based on 10 studies, and 18.4 (13.3, 27.2) for MOOD and social phobia based on three studies. The aOR (95% CI) ranged between 5.2 (2.9, 10.0) for MOOD and social phobia based on three studies, and 18.7 (16.2, 21.6) for MOOD and GAD based on five studies.

### 3.4 DEP and anxiety disorder: Lifetime comorbidity

We identified 48 studies (eReference 5) for lifetime comorbidity between DEP and anxiety disorders (Table 3). The crude OR (95% CI) ranged between 3.3 (2.7, 4.0) for DEP and specific phobia based on 14 studies, and 9.0 (5.6, 14.5) for DEP and GAD based on 11 studies. The aOR (95% CI) ranged between 2.4 (1.6, 3.5) for DEP and specific phobia based on seven studies, and 7.4 (4.4, 12.4) for DEP and panic disorder based on 11 studies.

### 3.5 DEP and anxiety disorder: Period prevalence comorbidity

We identified 68 studies (eReference 6) for period prevalence comorbidity between DEP and anxiety disorders (Table 3). The crude OR (95% CI) ranged between 3.7 (2.7, 5.0) for DEP and specific phobia based on eight studies, and 13.8 (9.9, 19.3) for DEP and GAD based on 22 studies. The aOR (95% CI) ranged between 2.9 (2.3, 3.7) for DEP and specific phobia based on three studies, and 11.7 (5.2, 26.3) for DEP and GAD based on 12 studies.

### 3.6 DYS and anxiety disorder: Lifetime comorbidity

We identified 16 studies (eReference 7) for lifetime comorbidity between DYS and anxiety disorders (Table 4). The crude OR (95% CI) ranged between 6.6 (3.8, 11.3) for DYS and OCD based on four studies, and 13.8 (12.3, 15.4) for DYS and GAD based on five studies. The only aOR (95% CI) was between DYS and social phobia: 4.1 (2.1, 8.2) based on five studies.

### 3.7 DYS and anxiety disorder: Period prevalence comorbidity

We identified 17 studies (eReference 8) for period prevalence comorbidity between any DYS and anxiety disorders (Table 4). The crude OR (95% CI) ranged between 6.9 (1.6, 29.3) for DYS and specific phobia based on four studies, and 17.2 (10.4, 28.4) for DYS and PTSD based on three studies. The aOR (95% CI) ranged between 2.3 (0.7, 7.8) for DYS and PTSD based on three studies, and 12.3 (9.8, 15.5) for DYS and GAD based on five studies.

### 3.8 BIPOLAR and anxiety disorder: Lifetime comorbidity

We identified 28 studies (eReference 9) for lifetime comorbidity between BIPOLAR and anxiety disorders (Table 5). The crude OR (95% CI) ranged between 4.4 (2.2, 9.0) for BIPOLAR and social phobia based on six studies, and 7.8 (6.5, 9.4) for BIPOLAR and ANX based on eight studies. The aOR (95% CI) ranged between 3.5 (1.9, 6.6) for BIPOLAR and specific phobia based on four studies, and 8.9 (5.2, 15.3) for BIPOLAR and GAD based on four studies.

### 3.9 BIPOLAR and anxiety disorder: Period prevalence comorbidity

We identified 14 studies (eReference 10) for period prevalence comorbidity between BIPOLAR and different anxiety disorders (Table 5). The crude OR (95% CI) ranged between 4.9 (for BIPOLAR and both ANX (2.0, 12.2) and also specific phobia (4.1, 5.8) based on three studies), and 11.6 (8.9, 15.0) for BIPOLAR and panic disorder based on three studies. The aOR (95% CI) ranged between 3.7 (2.1, 6.5) for BIPOLAR and social phobia based on four studies, and 7.4 (1.6, 34.0) for BIPOLAR and GAD based on three studies.

### 3.10 Temporally ordered associations between mood‐ and anxiety‐related disorders

Although the majority of these estimates could not be pooled because of low number of studies, there was an elevated risk of comorbidity between different MOOD (as a prior disorder) and different anxiety disorders (as a later disorder), and vice‐versa (etables 2 and 3). For example, those with DEP had 2‐3 times elevated risk of later ANX (aOR = 2.1: 95% CI 1.5, 2.8), those with ANX as a prior disorder had similar risk of developing DEP (aOR = 2.1: 95% CI 1.8, 2.5) (etable 3). The highest risk was observed between DEP as a prior disorder and social phobia as a later disorder (OR = 7.3: 95% CI 6.2, 8.7), while the corresponding risk was about one‐third for those with social phobia as a prior disorder and DEP as a later disorder (OR = 2.5: 95% CI 2.1, 3.1).

### 3.11 Overall findings, heterogeneity, quality scores and funnel plots

Most of the pooled estimates showed heterogeneity (the Q‐statistic and *I* ^2^ are presented with each forest plot). However, it is important to note that this heterogeneity does not detract from the main findings (i.e., there is strong comorbidity between the two disorders). The pooled estimates for all meta‐analyses were above one, and only one of the 76 uni‐directional meta‐analyses and two of the 14 bidirectional meta‐analyses had 95% confidence intervals that included 1 (Table 4: between DYS and PTSD; and etable 3: between OCD and DEP, and specific phobia and DEP respectively). The Eggers z (test) linked to respective funnel plots ranged between 0.04 (*p* = .97) for DEP and GAD (efigure 16), and 1.82 (*p* = .07) for DEP and panic disorder (efigure 21), thus not providing evidence of substantial publication bias for the various pooled estimates. The 14 funnel plots (with studies > 10) are presented in efigures. The quality score between studies ranged between 8 and 16 (Table 1) with the majority having quality scores in the upper range of the scale (median = 13, interquartile range: 12–15). As most of the meta‐analyses were based on small numbers of contributing studies, and because of the relative lack of variation in the quality scores, additional investigation of the impact of this score on the findings was not undertaken.

## 4 DISCUSSION

Our systematic review identified 171 articles from 37 countries over the last 38 years. Based on estimates from these studies, our systematic review generated 90 separate meta‐analyses that included 76 unidirectional and 14 temporally ordered bidirectional risk estimates. The median of these pooled estimates (OR) was 6.1 (range 1.5–18.7). Regardless of the specific nature of the mood‐ and anxiety‐related disorders, and regardless of the study design (e.g., diagnostic criteria), the pooled risk estimates of all the 90 meta‐analyses were above one. Of these estimates, 87 were significant (i.e., the 95% CI did not include 1), and 14 of the 90 pooled estimates had ORs that were greater than 10. Based on temporally ordered pooled estimates, we also found that the relationship was bidirectional. Regardless of which of the two disorders arose first, there was an increased risk of subsequently developing the other disorder. To the best of our knowledge, this is the most comprehensive systematic review and meta‐analysis of comorbidity between mood and anxiety disorders.

This study builds on previous systematic reviews by including a comprehensive range of mood and anxiety disorders. In addition, these estimates were sorted by design features related to the period of observation (period, lifetime prevalence etc.), temporal order, and the use of adjustments for covariates. Regardless of the many different types of pairs of disorders, the pooled estimates were uniformly above 1, and often were large (i.e., greater than 10‐fold risk). Overall, we found very strong comorbidity between mood and anxiety disorders, often higher than previously reported pooled estimates. For example, Pavlova et al. (2015) in a recent review found a three‐fold increased lifetime comorbidity between bipolar and anxiety disorder, whereas we found an elevated odds of about eight‐fold between this pair of disorders. In some disorder pairs (e.g., MOOD and GAD), the pooled estimate was as high as 19 times (aOR = 18.7). However, our estimates were broadly consistent with two studies that were published after the completion of our data extraction: a large register‐based study from Denmark (*n* = 5 940 778) (Plana‐Ripoll et al., 2019), and a trans‐national analysis that combined individual data from 27 countries (*n* = 145 990 survey respondents) (McGrath et al., 2020). Overall, the consistent patterns identified by our systematic review, and the findings from these two recent studies, provide convergent evidence of the strong comorbidity between these disorders.

With respect to the bidirectional nature of comorbidity between temporally ordered mood‐ and anxiety‐related disorders, we found that there was a two‐fold risk of developing ANX among those with DEP and vice‐versa. However, these estimates were based on a limited number of studies. Although this bidirectional association was broadly consistent with our recently conducted population‐based studies (McGrath et al., 2020; Plana‐Ripoll et al., 2019), there was a lack of consistency between these studies regarding the symmetry between the size of the reciprocal risk estimates (i.e., did the effect sizes differ according to order, regardless of the fact that both estimates were substantially above 1 and significant). Bi‐directionality within pairs of comorbid disorders suggests that these disorders may result from shared underlying risk factors (e.g., genetic; Levey et al., 2020; Purves et al., 2019; and/or shared early life exposures; Kessler et al., 1997).

With respect to study design features related to the temporal time‐frame, we found significant associations between MOOD and ANX when we pooled estimates from (a) lifetime, (b) period prevalence, and (c) temporally ordered estimates. The overall pattern of strong comorbidity between mood‐ and anxiety‐ disorders was found in both crude and adjusted estimates, consistent with findings from the recently published WHO World Mental Health Survey study (McGrath et al., 2020). We meta‐analyzed a large number of studies that presented a strong association between lifetime comorbidity between mood and anxiety disorders. The highest pooled estimate was found between BIPOLAR and GAD (aOR 8.9) followed by BIPOLAR and OCD (aOR 8.4), BIPOLAR and ANX (aOR 7.7), and DEP and panic disorder (aOR 7.4). Similarly, based on period prevalence estimates, the largest estimate was about 19‐fold between MOOD and GAD (aOR 18.7) followed by MOOD and ANX (aOR 11.8), DYS and GAD (aOR 12.3), and DEP and GAD (aOR 11.7).

### 4.1 Understanding heterogeneity

Despite parsing the estimates by a range of methodological features, the pooled estimates were largely heterogeneous according to Q‐statistics and *I* ^2^ test (although a recent report showed that *I* ^2^ is not an absolute measure of heterogeneity) (Borenstein et al., 2017). It is important to note that despite the heterogeneity underlying the pooled estimates, we found that the majority of the pooled estimates were large (14 estimates above 10‐fold), and only three had 95% confidence intervals that included 1. Given the imprecise nature of psychiatric epidemiology, some degree of heterogeneity is to be expected in our analyses, even when these studies are based on high quality standardized methods (Gureje, 2009; Nabavi et al., 2015). Trans‐national studies have found that the prevalence of anxiety and depression varies between sites and across time (related to conflict, natural disaster, access to substances of abuse, cultural issues, availability to treatment services etc.) (Scott et al., 2018). Thus, comorbidity based on prevalence estimates will also be expected to vary.

### 4.2 Understanding comorbidity, and clinical implications

This systematic review provided risk estimates (Odds ratios) as the evidence of comorbidity between mood and anxiety disorders. Although it has the appealing feature of summarizing two numbers (the risk in one group and the risk in the other), it provides no information about the underlying absolute risk (the number of events over number of people). Absolute risk estimates are more useful for clinicians to actively monitor comorbid disease development. For example, recent population based studies (McGrath et al., 2020) show that in the first 5 years of receiving a diagnosis of mood disorder more than a quarter of patients develop anxiety disorder. We encourage future studies to provide both relative and absolute risk estimates to better understand the epidemiological landscape of comorbidity.

### 4.3 Limitations

The systematic review has several important limitations. Firstly, for some studies we had to calculate the estimates based on raw data which may misrepresent true weighted estimates. Ideally, population based studies (e.g., survey data) use weightings that incorporates sample selection, nonresponse and post‐stratification and so forth for the presentation of frequencies and estimates (Kessler et al., 2011; Kessler & Ustun, 2004). We urge caution in the interpretation of unadjusted estimates. Secondly, to pool estimates, we combined similar disorders into broad categories which may create some heterogeneity between estimates as well as loss of fine‐grain data. For example, we used “broadly defined mood disorder” (MOOD) for disorders such as depression, dysthymia and bipolar disorders. Thirdly, estimates from population surveys were often based on interviews from lay interviewers which may inflate comorbidity. On the other hand, lifetime diagnoses were based on retrospective reports that may underestimate prevalence, and therefore deflate comorbidity structure. Pooled estimates usually minimize this discrepancy. Fourthly, surveys usually do not take into account treatment history, which may interrupt progression of comorbidity, and thus distort comorbidity estimates (Kessler et al., 2011). In addition, diagnostic overlap within disorders (e.g., bipolar and depression) also may contribute misleading comorbidity estimates. Finally, despite our endeavor to include as many articles as possible by searching important databases, grey literature, as well as snowballing for additional articles, it is possible that some studies may be missed.

## 5 CONCLUSIONS

This systematic review provides compelling evidence that there is substantial comorbidity between various mood‐ and anxiety‐related disorders. A recent commentary by Hyman (2019) emphasized the convergence of evidence from (a) comorbidity studies (such as the current meta‐analysis), and (b) twin and genome‐wide association studies. Different types of mental disorders often have a shared risk architecture, which could underpin the pathogenesis of mental disorders and the patterns of comorbidity seen in the current study (Anttila et al., 2018; Kendler et al., 2011; Kessler et al., 1996, 2011). From a clinical perspective, it is important to remain vigilant for the prompt identification and treatment of comorbidity between anxiety‐ and depression‐related disorders.

## CONFLICT OF INTERESTS

The authors declare that there are no conflict of interests.

## AUTHOR CONTRIBUTIONS

Sukanta Saha and John J. McGrath conceived the study. Carmen C. W. Lim, John J. McGrath and Sukanta Saha conducted the analysis. Sukanta Saha, Carmen C. W. Lim, and John J. McGrath drafted and edited the manuscript. All authors contributed to refinement of the study protocol and approved the final manuscript.

## Supporting information

Supporting information.

## Data Availability

All data underlying the meta‐analyses and code can be found at this URL: https://github.com/clim072/NB-SR_MOOD_ANX.
