# Supplementary material for: Co‐morbidity between mood and anxiety disorders: A systematic review and meta‐analysis
Source: Depress Anxiety. 2020 Nov 22;38(3):286–306. doi: 10.1002/da.23113 (PMC7984258; doi:10.1002/da.23113)

CO-MORBIDITY BETWEEN MOOD AND ANXIETY DISORDERS: A SYSTEMATIC REVIEW AND META-ANALYSIS

SUPPLEMENTARY MATERIAL

Contents

Supplementary Methods (eMethods) 7
eMethods 1 Search strings........ 7
eMethods 2. Selection criteria: inclusion and exclusion criteria 11
eMethods 3. Search and data extraction methodology 12
eMethods 4. Diagnostic instruments and diagnostic criteria for Mood and Anxiety disorders... 13
eMethods 5. Overlap assessment using *Study-level* and *Estimate-level* filters........ 14
eMethods 6. Quality Reporting Scale (QRS) 19
eMethods 7. Broad categories of names used for major disorders 21
Supplementary Tables (etable) 22
etable 1. Characteristics of the included studies presenting temporally-ordered estimates (n=36)........ 22
eTable 2. Pooled estimates for broadly-defined mood disorder (MOOD) and anxiety disorders 25
eTable 3. Pooled estimates for broadly-defined depressive disorder (DEP) and anxiety disorders 26
References (eReferences) 27
eReference 1. Table 1. Studies presenting lifetime and period prevalence estimates (147 studies)........ 27
eReference 2. eTable 1. Studies presenting temporally-ordered estimates (36 studies) 36
eReference 3. Mood and anxiety: lifetime comorbidity (9 studies) 38
eReference 4. MOOD and anxiety disorder: period prevalence comorbidity (20 studies) 39
eReference 5. DEP and anxiety disorder: lifetime comorbidity (48 studies)........ 40
eReference 6. DEP and anxiety disorder: period prevalence comorbidity (68 studies)........ 43
eReference 7. DYS and anxiety disorder: lifetime comorbidity (16 studies) 47
eReference 8. DYS and anxiety disorder: period prevalence comorbidity (17 studies) 47
eReference 9. BIPOLAR and anxiety disorder: lifetime comorbidity (28 studies) 49
eReference 10. BIPOLAR and anxiety disorder: period prevalence comorbidity (14 studies) 50
Supplementary Figures (efigure) 52
efigure 1 Studies by country 52
efigure 2 Forest plot of the random-effects meta-analysis of lifetime comorbidity between broadly-defined mood and broadly-defined anxiety disorders (unadjusted) 53

efigure 3 Funnel plot of the lifetime comorbidity between broadly-defined mood and broadly-defined anxiety disorders (unadjusted) 54

efigure 4 Forest plot of the random-effects meta-analysis of lifetime comorbidity between broadly-defined mood and broadly-defined anxiety disorders (adjusted) 55

efigure 5 Forest plot of the random-effects meta-analysis of lifetime comorbidity between broadly-defined mood and social phobia (unadjusted) 56

efigure 6 Forest plot of the random-effects meta-analysis of lifetime comorbidity between broadly-defined mood and specific phobia (unadjusted) 57

efigure 7 Forest plot of the random-effects meta-analysis of lifetime comorbidity between broadly-defined mood and broadly-defined anxiety disorder (unadjusted) 58

efigure 8 Forest plot of the random-effects meta-analysis of lifetime comorbidity between broadly-defined mood and social phobia (unadjusted) 59

efigure 9 Forest plot of the random-effects meta-analysis of lifetime comorbidity between broadly-defined mood and social phobia (adjusted) 60

efigure 10 Forest plot of the random-effects meta-analysis of lifetime comorbidity between broadly-defined depressive disorders and agoraphobia (unadjusted) 61

efigure 11 Funnel plot of the lifetime comorbidity between broadly-defined depressive disorders and agoraphobia (unadjusted) 62

efigure 12 Forest plot of the random-effects meta-analysis of lifetime comorbidity between broadly-defined depressive disorders and obsessive compulsive disorder (unadjusted) 63

efigure 13 Funnel plot of the lifetime comorbidity between broadly-defined depressive disorders and obsessive compulsive disorder (unadjusted) 64

efigure 14 Forest plot of the random-effects meta-analysis of lifetime comorbidity between broadly-defined depressive disorders and obsessive compulsive disorder (adjusted) 65

efigure 15 Forest plot of the random-effects meta-analysis of lifetime comorbidity between broadly-defined depressive disorders and generalized anxiety disorder (unadjusted) 66

efigure 16 Funnel plot of the lifetime comorbidity between broadly-defined depressive disorders and generalized anxiety disorder (unadjusted) 67

efigure 17 Forest plot of the random-effects meta-analysis of lifetime comorbidity between broadly-defined depressive disorders and generalized anxiety disorder (adjusted) 68

efigure 18 Forest plot of the random-effects meta-analysis of lifetime comorbidity between broadly-defined depressive disorders and panic disorder (unadjusted) 69

efigure 19 Funnel plot of the lifetime comorbidity between broadly-defined depressive disorders and panic disorder (unadjusted) 70

efigure 20 Forest plot of the random-effects meta-analysis of lifetime comorbidity between broadly-defined depressive disorders and panic disorder (adjusted) 71

efigure 21 Funnel plot of the lifetime comorbidity between broadly-defined depressive disorders and panic disorder (adjusted) 72

efigure 22 Forest plot of the random-effects meta-analysis of lifetime comorbidity between broadly-defined depressive disorders and post-traumatic stress disorder (unadjusted) 73

efigure 23 Forest plot of the random-effects meta-analysis of lifetime comorbidity between broadly-defined depressive disorders and social phobia (unadjusted) 74

efigure 24 Funnel plot of the lifetime comorbidity between broadly-defined depressive disorders and social phobia (unadjusted) 75

efigure 25 Forest plot of the random-effects meta-analysis of lifetime comorbidity between broadly-defined depressive disorders and social phobia (adjusted) 76

efigure 26 Forest plot of the random-effects meta-analysis of lifetime comorbidity between broadly-defined depressive disorders and specific phobia (unadjusted) 77

efigure 27 Funnel plot of the lifetime comorbidity between broadly-defined depressive disorders and specific phobia (unadjusted) 78

efigure 28 Forest plot of the random-effects meta-analysis of lifetime comorbidity between broadly-defined depressive disorders and specific phobia (adjusted) 79

efigure 29 Forest plot of the random-effects meta-analysis of lifetime comorbidity between broadly-defined depressive disorders and anxiety disorder (unadjusted) 80

efigure 30 Funnel plot of the lifetime comorbidity between broadly-defined depressive disorders and anxiety disorder (unadjusted) 81

efigure 31 Forrest plot of the lifetime comorbidity between broadly-defined depressive disorders and anxiety disorder (adjusted) 82

efigure 32 Forest plot of the random-effects meta-analysis of period prevalence comorbidity between broadly-defined depressive disorders and agoraphobia (unadjusted) 83

efigure 33 Funnel plot of the period prevalence comorbidity between broadly-defined depressive disorders and agoraphobia (unadjusted) 84

efigure 34 Forest plot of the random-effects meta-analysis of period prevalence comorbidity between broadly-defined depressive disorders and agoraphobia (adjusted) 85

efigure 35 Forest plot of the random-effects meta-analysis of period prevalence comorbidity between broadly-defined depressive disorders and obsessive compulsive disorder (unadjusted) 86

efigure 36 Funnel plot of the period prevalence comorbidity between broadly-defined depressive disorders and obsessive compulsive disorder (unadjusted) 87

efigure 37 Forest plot of the random-effects meta-analysis of period prevalence comorbidity between broadly-defined depressive disorders and obsessive compulsive disorder (adjusted). 88

efigure 38 Funnel plot of the period prevalence comorbidity between broadly-defined depressive disorders and generalized anxiety disorder (unadjusted) 89

efigure 39 Funnel plot of the period prevalence comorbidity between broadly-defined depressive disorders and generalized anxiety disorder (adjusted) 90

efigure 40 Forest plot of the random-effects meta-analysis of period prevalence comorbidity between broadly-defined depressive disorders and panic disorder (unadjusted) 91

efigure 41 Funnel plot of the period prevalence comorbidity between broadly-defined depressive disorders and panic disorder (unadjusted) 92

efigure 42 Forest plot of the random-effects meta-analysis of period prevalence comorbidity between broadly-defined depressive disorders and panic disorder (adjusted) 93

efigure 43 Forest plot of the random-effects meta-analysis of period prevalence comorbidity between broadly-defined depressive disorders and post-traumatic stress disorder (unadjusted) 94

efigure 44 Forest plot of the random-effects meta-analysis of period prevalence comorbidity between broadly-defined depressive disorders and post-traumatic stress disorder (adjusted) . 95

efigure 45 Forest plot of the random-effects meta-analysis of period prevalence comorbidity between broadly-defined depressive disorders and social phobia (unadjusted) 96

efigure 46 Forest plot of the random-effects meta-analysis of period prevalence comorbidity between broadly-defined depressive disorders and social phobia (adjusted)........ 97

efigure 47 Forest plot of the random-effects meta-analysis of period prevalence comorbidity between broadly-defined depressive disorders and specific phobia (unadjusted) 98

efigure 48 Forest plot of the random-effects meta-analysis of period prevalence comorbidity between broadly-defined depressive disorders and specific phobia (adjusted)........ 99

efigure 49 Forest plot of the random-effects meta-analysis of period prevalence comorbidity between broadly-defined depressive disorders and broadly-defined anxiety disorder (unadjusted)........ 100

efigure 50 Forest plot of the period prevalence comorbidity between broadly-defined depressive disorders and broadly-defined anxiety disorder (unadjusted)........ 101

efigure 51 Forest plot of the random-effects meta-analysis of period prevalence comorbidity between broadly-defined depressive disorders and broadly-defined anxiety disorder (adjusted) 102

efigure 52 Forest plot of the period prevalence comorbidity between broadly-defined depressive disorders and broadly-defined anxiety disorder (adjusted)........ 103

efigure 53 Forest plot of the random-effects meta-analysis of lifetime comorbidity between broadly-defined dysthymic disorders and obsessive compulsive disorder (unadjusted)........ 104

efigure 54 Forest plot of the random-effects meta-analysis of lifetime comorbidity between broadly-defined dysthymic disorders and generalized anxiety disorder (unadjusted)........ 105

efigure 55 Forest plot of the random-effects meta-analysis of lifetime comorbidity between broadly-defined dysthymic disorders and social phobia (unadjusted)........ 106

efigure 56 Forest plot of the random-effects meta-analysis of lifetime comorbidity between broadly-defined dysthymic disorders and social phobia (adjusted)........ 107

efigure 57 Forest plot of the random-effects meta-analysis of lifetime comorbidity between broadly-defined dysthymic disorders and specific phobia (unadjusted)........ 108

efigure 58 Forest plot of the random-effects meta-analysis of period prevalence comorbidity between broadly-defined dysthymic disorders and agoraphobia (unadjusted) 109

efigure 59 Forest plot of the random-effects meta-analysis of lifetime comorbidity between broadly-defined dysthymic disorders and obsessive compulsive disorder (unadjusted)........ 110

efigure 60 Forest plot of the random-effects meta-analysis of lifetime comorbidity between broadly-defined dysthymic disorders and generalised anxiety disorder (unadjusted) 111

efigure 61 Forest plot of the random-effects meta-analysis of lifetime comorbidity between broadly-defined dysthymic disorders and generalized anxiety disorder (adjusted) 112

efigure 62 Forest plot of the random-effects meta-analysis of lifetime comorbidity between broadly-defined dysthymic disorders and panic disorder (unadjusted)....................................113

efigure 63 Forest plot of the random-effects meta-analysis of lifetime comorbidity between broadly-defined dysthymic disorders and post-traumatic stress disorder (unadjusted)...........114

efigure 64 Forest plot of the random-effects meta-analysis of lifetime comorbidity between broadly-defined dysthymic disorders and post-traumatic stress disorder (adjusted) 115

efigure 65 Forest plot of the random-effects meta-analysis of lifetime comorbidity between broadly-defined dysthymic disorders and social phobia (unadjusted)......................................116

efigure 66 Forest plot of the random-effects meta-analysis of lifetime comorbidity between broadly-defined dysthymic disorders and social phobia (adjusted)..117

efigure 67 Forest plot of the random-effects meta-analysis of lifetime comorbidity between broadly-defined dysthymic disorders and specific phobia (unadjusted)...................................118

efigure 68 Forest plot of the random-effects meta-analysis of lifetime comorbidity between broadly-defined bipolar disorders and agoraphobia (unadjusted) 119

efigure 69 Forest plot of the random-effects meta-analysis of lifetime comorbidity between broadly-defined bipolar disorders and obsessive compulsive disorder (unadjusted)................120

efigure 70 Forest plot of the random-effects meta-analysis of lifetime comorbidity between broadly-defined bipolar disorders and obsessive compulsive disorder (adjusted)....................121

efigure 71 Forest plot of the random-effects meta-analysis of lifetime comorbidity between broadly-defined bipolar disorders and generalized anxiety disorder (unadjusted)..................122

efigure 72 Forest plot of the random-effects meta-analysis of lifetime comorbidity between broadly-defined bipolar disorders and generalized anxiety disorder (adjusted) 123

efigure 73 Forest plot of the random-effects meta-analysis of lifetime comorbidity between broadly-defined bipolar disorders and panic disorder (unadjusted)...124

efigure 74 Forest plot of the random-effects meta-analysis of lifetime comorbidity between broadly-defined bipolar disorders and panic disorder (adjusted)...125

efigure 75 Forest plot of the random-effects meta-analysis of lifetime comorbidity between broadly-defined bipolar disorders and post-traumatic stress disorder (adjusted) 126

efigure 76 Forest plot of the random-effects meta-analysis of lifetime comorbidity between broadly-defined bipolar disorders and social phobia (unadjusted)...127

efigure 77 Forest plot of the random-effects meta-analysis of lifetime comorbidity between broadly-defined bipolar disorders and social phobia (adjusted)..128

efigure 79 Forest plot of the random-effects meta-analysis of lifetime comorbidity between broadly-defined bipolar disorders and specific phobia (adjusted)..130

efigure 80 Forest plot of the random-effects meta-analysis of lifetime comorbidity between broadly-defined bipolar disorders and broadly-defined anxiety disorder (unadjusted)............131

efigure 81 Forest plot of the random-effects meta-analysis of lifetime comorbidity between broadly-defined bipolar disorders and broadly-defined anxiety disorder (adjusted) 132

efigure 82 Forest plot of the random-effects meta-analysis of period prevalence comorbidity between broadly-defined bipolar disorders and obsessive compulsive disorder (unadjusted) 133

efigure 83 Forest plot of the random-effects meta-analysis of period prevalence comorbidity between broadly-defined bipolar disorders and obsessive compulsive disorder (adjusted)..... 134

efigure 84 Forest plot of the random-effects meta-analysis of period prevalence comorbidity between broadly-defined bipolar disorders and generalized anxiety disorder (unadjusted).... 135

efigure 85 Forest plot of the random-effects meta-analysis of period prevalence comorbidity between broadly-defined bipolar disorders and generalized anxiety disorder (adjusted)........ 136

efigure 86 Forest plot of the random-effects meta-analysis of period prevalence comorbidity between broadly-defined bipolar disorders and panic disorder (unadjusted) 137

efigure 87 Forest plot of the random-effects meta-analysis of period prevalence comorbidity between broadly-defined bipolar disorders and social phobia (unadjusted) 138

efigure 88 Forest plot of the random-effects meta-analysis of period prevalence comorbidity between broadly-defined bipolar disorders and social phobia (adjusted)................................ 139

efigure 89 Forest plot of the random-effects meta-analysis of period prevalence comorbidity between broadly-defined bipolar disorders and specific phobia (unadjusted) 140

efigure 90 Forest plot of the random-effects meta-analysis of period prevalence comorbidity between broadly-defined bipolar disorders and broadly-defined anxiety disorder (unadjusted) 141

Supplementary Methods (eMethods)

eMethods 1 Search strings

Medline (PubMed)

#1

comorbidity OR comorbidities OR co-morbidity OR co-morbidities OR multiple condition OR multiple conditions OR multiple diagnosis OR multiple disease OR multiple diseases OR multiple health problem OR multiple illness OR multiple morbid* OR multiple pathology OR multiple pathologies OR multi disease OR multi-disease OR multi-diseases OR multimorbidity OR multimorbidities OR multi-morbidity OR multi-morbidities OR multipathology OR multipathologies OR multi-pathology OR multi-pathologies OR associated condition OR associated diagnos* OR associated disease OR associated health problem OR associated illness OR associated morbidity OR associated morbidities OR associated pathology OR associated pathologies OR coexisting condition OR co-existing condition OR coexisting diagnos* OR co-existing diagnos* OR coexisting disease OR co-existing disease* OR coexisting health problem OR co-existing health problem* OR coexisting illness OR co-existing illness OR existing morbid* OR co existing morbid* OR coexisting patholog* OR co-existing patholog* OR comorbid condition* OR co-morbid condition* OR comorbid diagnos* OR co-morbid diagnos* OR comorbid disease* OR co-morbid disease* OR comorbid health problem OR co-morbid health problem OR comorbid illness OR co-morbid illness OR comorbid patholog* OR co-morbid patholog* OR concurrent condition OR concurrent diagnos* OR concurrent disease OR concurrent health problem OR concurrent illness OR concurrent morbid* OR concurrent patholog* OR cooccurring condition OR co-occurring condition OR cooccurring diagnos* OR co-occurring diagnos* OR cooccurring disease OR co-occurring disease OR cooccurring health problem OR co-occurring health problem OR cooccurring illness OR co-occurring illness OR cooccurring morbid* OR co-occurring morbid* OR cooccurring patholog* OR co-occurring patholog* OR pluripathology OR pluripathologies OR polypathology OR polypathologies OR poly-pathology OR poly-pathologies

#2

((“1980/01/01” [PDAT] : “2017/12/31” [PDAT]) NOT (animal* [TIAB]))

#3

(mood disorder* [TIAB] OR depress* [TIAB] OR MDD [TIAB] OR dysthymi* [TIAB] OR cyclothymi* [TIAB] OR bipolar [TIAB] OR BP [TIAB] OR mania [TIAB] OR manic* [TIAB] OR affective disorder* [TIAB])

#3

(anxiety [TIAB] OR anxiety disorder* [TIAB] OR agoraphobia* [TIAB] OR generalized anxiety disorder* [TIAB] OR GAD [TIAB] OR obsessive compulsive disorder* [TIAB] OR OCD [TIAB] OR panic disorder* [TIAB] OR posttraumatic stress* [TIAB] OR post traumatic stress* [TIAB] OR PTSD [TIAB] OR social phobia* [TIAB] OR specific phobia* [TIAB] OR neurotic disorder* [TIAB] OR adjustment disorder* [TIAB] OR acute stress disorder* [TIAB] OR acute stress reaction [TIAB] OR dissociative disorder* [TIAB] OR neurotic disorder* [TIAB])

Final search string

(#1 AND #2 AND #3 AND #4)

Search results: 38,641

EMBASE

#1

(comorbidity OR comorbidities OR co-morbidity OR co-morbidities OR ‘multiple condition’ OR ‘multiple conditions’ OR ‘multiple diagnosis’ OR ‘multiple disease*’ OR ‘multiple health problem’ OR ‘multiple illness*’ OR ‘multiple morbid*’ OR ‘multiple pathology’ OR ‘multiple pathologies’ OR ‘multi disease’ OR ‘multi-disease’ OR multi-diseases OR multimorbidity OR multimorbidities OR multi-morbidity OR multi-morbidities OR multipathology OR multipathologies OR multi-pathology OR multi-pathologies OR ‘associated condition’ OR ‘associated diagnos*’ OR ‘associated disease’ OR ‘associated health problem’ OR ‘associated illness’ OR ‘associated morbidity’ OR ‘associated morbidities’ OR ‘associated pathology’ OR ‘associated pathologies’ OR ’coexisting condition’ OR ‘co-existing condition’ OR ‘coexisting diagnos*’ OR ‘co-existing diagnos*’ OR ‘coexisting disease’ OR ‘co-existing disease*’ OR ‘coexisting health problem’ OR ‘co-existing health problem*’ OR ‘coexisting illness’ OR ‘co-existing illness’ OR ‘existing morbid*’ OR ‘co existing morbid*’ OR ‘coexisting patholog*’ OR ‘co-existing patholog*’ OR ‘comorbid condition*’ OR ‘co-morbid condition*’ OR ‘comorbid diagnos*’ OR ‘co-morbid diagnos*’ OR ‘comorbid disease*’ OR ‘co-morbid disease*’ OR ‘comorbid health problem’ OR ‘co-morbid health problem’ OR ‘comorbid illness’ OR ‘co-morbid illness’ OR ‘comorbid patholog*’ OR ‘co-morbid patholog*’ OR ‘concurrent condition’ OR ‘concurrent diagnos*’ OR ‘concurrent disease’ OR ‘concurrent health problem’ OR ‘concurrent illness’ OR ‘comorbid patholog*’ OR ‘co-morbid patholog*’ OR ‘concurrent condition’ OR ‘concurrent diagnos*’ OR ‘concurrent disease’ OR ‘concurrent health problem’ OR ‘concurrent illness’ OR ‘concurrent morbid*’ OR ‘concurrent patholog*’ OR ‘cooccurring condition’ OR ‘co-occurring condition’ OR ‘cooccurring diagnos*’ OR ’co-occurring diagnos*’ OR ‘cooccurring disease’ OR ‘co-occurring disease’ OR ’cooccurring health problem’ OR ‘co-occurring health problem’ OR ‘cooccurring illness’ OR ‘co-occurring illness’ OR ‘cooccurring morbid*’ OR ‘co-occurring morbid*’ OR ‘cooccurring patholog*’ OR ‘co-occurring patholog*’ OR pluripathology OR pluripathologies OR polypathology OR polypathologies OR poly-pathology OR poly-pathologies)

#2

[1980-2017]/py NOT ('animal'/exp OR animal OR 'nonhuman'/exp OR nonhuman)

#3

(‘mood disorder*’ OR ‘depress*’ OR MDD OR ‘dysthymi*’ OR ‘cyclothymi*’ OR bipolar OR BP OR mania OR ‘manic*’ OR ‘affective disorder*’)

#4

(anxiety OR ‘anxiety disorder*’ OR ‘agoraphobia*’ OR ‘generalized anxiety disorder*’ OR GAD OR ‘obsessive compulsive disorder*’ OR OCD OR ‘panic disorder*’ OR ‘posttraumatic stress*’ OR ‘post traumatic stress*’ OR PTSD OR ‘social phobia*’ OR ‘specific phobia*’ OR ‘neurotic disorder*’ OR ‘adjustment disorder*’ OR ‘acute stress disorder*’ OR ‘acute stress reaction’ OR ‘dissociative disorder*’ OR ‘neurotic disorder*’)

(#1 AND #2 AND #3 AND #4)

Search results: 921

CINAHL (EBSCOhost)

#1

(comorbidity OR comorbidities OR co-morbidity OR co-morbidities OR 'multiple condition' OR 'multiple conditions' OR 'multiple diagnosis' OR 'multiple disease*' OR 'multiple health problem' OR 'multiple illness*' OR 'multiple morbid*' OR 'multiple pathology' OR 'multiple pathologies' OR 'multi disease' OR multi-disease OR multi-diseases OR multimorbidity OR multimorbidities OR multi-morbidity OR multi-morbidities OR multipathology OR multipathologies OR multi-pathology OR multi-pathologies OR 'associated condition' OR 'associated diagnos*' OR 'associated disease' OR 'associated health problem' OR 'associated illness' OR 'associated morbidity' OR 'associated morbidities' OR 'associated pathology' OR 'associated pathologies' OR 'coexisting condition' OR 'co-existing condition' OR 'coexisting diagnos*' OR 'co-existing diagnos*' OR 'coexisting disease' OR 'co-existing disease*' OR 'coexisting health problem' OR 'co-existing health problem*' OR 'coexisting illness' OR 'co-existing illness' OR 'existing morbid*' OR 'co existing morbid*' OR 'coexisting patholog*' OR 'co-existing patholog*' OR 'comorbid condition*' OR 'co-morbid condition*' OR 'comorbid diagnos*' OR 'co-morbid diagnos*' OR 'comorbid disease*' OR 'co-morbid disease*' OR 'comorbid health problem' OR 'co-morbid health problem' OR 'comorbid illness' OR 'co-morbid illness' OR 'comorbid patholog*' OR 'co-morbid patholog*' OR 'concurrent condition' OR 'concurrent diagnos*' OR 'concurrent disease' OR 'concurrent health problem' OR 'concurrent illness' OR 'comorbid patholog*' OR 'co-morbid patholog*' OR 'concurrent condition' OR 'concurrent diagnos*' OR 'concurrent disease' OR 'concurrent health problem' OR 'concurrent illness' OR 'concurrent morbid*' OR 'concurrent patholog*' OR 'cooccurring condition' OR 'co-occurring condition' OR 'cooccurring diagnos*' OR 'co-occurring diagnos*' OR 'cooccurring disease' OR 'co-occurring disease' OR 'cooccurring health problem' OR 'co-occurring health problem' OR 'cooccurring illness' OR 'co-occurring illness' OR 'cooccurring morbid*' OR 'co-occurring morbid*' OR 'cooccurring patholog*' OR 'co-occurring patholog*' OR pluripathology OR pluripathologies OR polypathology OR polypathologies OR poly-pathology OR poly-pathologies) NOT (animal OR nonhuman)

#2

('mood disorder*' OR 'depress*' OR MDD OR 'dysthymi*' OR 'cyclothymi*' OR bipolar OR BP OR mania OR 'manic*' OR 'affective disorder*')

#3

(anxiety OR 'anxiety disorder*' OR 'agoraphobia*' OR 'generalized anxiety disorder*' OR GAD OR 'obsessive compulsive disorder*' OR OCD OR 'panic disorder*' OR 'posttraumatic stress*' OR 'post traumatic stress*' OR PTSD OR 'social phobia*' OR 'specific phobia*' OR 'neurotic disorder*' OR 'adjustment disorder*' OR 'acute stress disorder*' OR 'acute stress reaction' OR 'dissociative disorder*' OR 'neurotic disorder*')

Final search string

(#1 AND #2 AND #3)

Search results: 3650

Web of Science

#1

((comorbidity OR comorbidities OR co-morbidity OR co-morbidities OR multiple condition' OR 'multiple conditions' OR 'multiple diagnosis' OR 'multiple disease*' OR 'multiple health problem' OR 'multiple illness*' OR 'multiple morbid*' OR 'multiple pathology' OR 'multiple pathologies' OR 'multi disease' OR multi-disease OR multi-diseases OR multimorbidity OR multimorbidities OR multi-morbidity OR multi-morbidities OR multipathology OR multipathologies OR multi-pathology OR multi-pathologies OR 'associated condition' OR 'associated diagnos*' OR 'associated disease' OR 'associated health problem' OR 'associated illness' OR 'associated morbidity' OR 'associated morbidities' OR 'associated pathology' OR 'associated pathologies' OR 'coexisting condition' OR 'co-existing condition' OR 'coexisting diagnos*' OR 'co-existing diagnos*' OR 'coexisting disease' OR 'co-existing disease*' OR 'coexisting health problem' OR 'co-existing health problem*' OR 'coexisting

illness' OR 'co-existing illness' OR 'existing morbid*' OR 'co existing morbid*' OR 'coexisting patholog*' OR 'co-existing patholog*' OR 'comorbid condition*' OR 'co-morbid condition*' OR 'comorbid diagnos*' OR 'co-morbid diagnos*' OR 'comorbid disease*' OR 'co-morbid disease*' OR 'comorbid health problem' OR 'co-morbid health problem' OR 'comorbid illness' OR 'co-morbid illness' OR 'comorbid patholog*' OR 'co-morbid patholog*' OR 'concurrent condition' OR 'concurrent diagnos*' OR 'concurrent disease' OR 'concurrent health problem' OR 'concurrent illness' OR 'comorbid patholog*' OR 'co-morbid patholog*' OR 'concurrent condition' OR 'concurrent diagnos*' OR 'concurrent disease' OR 'concurrent health problem' OR 'concurrent illness' OR 'concurrent morbid*' OR 'concurrent patholog*' OR 'cooccurring condition' OR 'co-occurring condition' OR cooccurring diagnos* OR co-occurring diagnos* OR 'cooccurring disease' OR 'co-occurring disease' OR 'cooccurring health problem' OR 'co-occurring health problem' OR 'cooccurring illness' OR 'co-occurring illness' OR 'cooccurring morbid*' OR 'co-occurring morbid*' OR 'cooccurring patholog*' OR 'co-occurring patholog*' OR pluripathology OR pluripathologies OR polypathology OR polypathologies OR poly-pathology OR poly-pathologies) NOT (animal OR nonhuman))
#2
TOPIC: (('mood disorder*' OR 'depress*' OR MDD OR 'dysthymi*' OR 'cyclothymi*' OR bipolar OR BP OR mania OR 'manic*' OR 'affective disorder*'))
#3
TOPIC: ((anxiety OR 'anxiety disorder*' OR 'agoraphobia*' OR 'generalized anxiety disorder*' OR GAD OR 'obsessive compulsive disorder*' OR OCD OR 'panic disorder*' OR 'posttraumatic stress*' OR 'post traumatic stress*' OR PTSD OR 'social phobia*' OR 'specific phobia*' OR 'neurotic disorder*' OR 'adjustment disorder*' OR 'acute stress disorder*' OR 'acute stress reaction' OR 'dissociative disorder*' OR 'neurotic disorder*'))
Refined by: [excluding] Databases: (MEDLINE) AND Databases: (WOS)
Databases= WOS, CCC, DRCI, DIIDW, KJD, MEDLINE, RSCI, SCIELO Timespan=1980-2017
Search language=Auto
Final search string
(#3 AND #2 AND #1)

Search results: 4,293

Final search string

(#1 AND #2 AND #3 AND #4)

Search results: 38,641

eMethods 2. Selection criteria: inclusion and exclusion criteria

Selection criteria

The inclusion and exclusion criteria are as follows:

Inclusion criteria

1. Human only studies
2. Study has to report an association in the form of risk ratio or equivalent (i.e. hazard ratio, relative risk, odds ratio) and corresponding variance or provide data for calculating the estimates
3. General population
4. Peer-reviewed articles using the following study designs:
    - Case-control (including nested case-control) studies
    - Cohort (retrospective, prospective) studies
    - Cross-sectional studies
5. Study clearly state the diagnostic criteria (ICD/DSM etc.)

Exclusion criteria

1. Sub- or ‘special’ populations (e.g., prison, homeless, illness group like cancer, cardiovascular, hypertension, Down Syndrome, Multiple Sclerosis, HIV etc.), and studies with caregiver, burden of disease, sleep, insomnia, suicide, subjective memory complaints, mortality, MRI studies
2. Experimental studies, clinical trials, case reports, letters and commentaries.
3. Reviews (scoping, narrative reviews etc.)
4. Systematic reviews
5. Genetic study
6. Comorbidity of mental and physical disorder

.

eMethods 3. Search and data extraction methodology

eMethods 3.1 Search strategy

For delineating discrete estimates when more than one estimate was presented within a paper, we used a set of ad hoc rules to select one estimates. For example, (a) for inclusion of one adjusted risk estimate among multiples, we selected adjusted estimates that used the most number of covariates; (b), among data on more than one follow-up period, the longest follow-up period point was chosen for the data extraction, and (c) for estimates without uncertainty intervals but when corresponding exact p-values were presented. For the p-values that presented to the nearest 2-3 decimal points, we imposed a conservative assumption for p-values. For example, if an estimate with p <.01 was provided, we used p = 0.01. Similarly, for p <.05, we used p= 0.049, for p > 0.5, p = 0.5 and so on.

eMethods 3.2 Sequential filters

For identifying discrete data from overlapping studies, we used two types of filters, a *'study-level filter'* and an *'estimate-level filter'*. For overlapping data in related studies, first, we used a 'most informative rule'. For example, if a study overlap by year of study from the same geographical area, we selected estimates based on longer epoch (that covered more study period). Similarly, if study participants overlapped by age with other study from the same population, we used the widest age range over the narrower age strata. In the second step, we used an 'estimate-level filter' for discrete estimates when estimates were derived from the same database (s) but overlap. We had to use several ad-hoc rules that were operationalised and mutually exclusive. First, we applied previously mentioned 'most informative rules' to override estimates that were overlapped within the same cohorts. If this rule could not be applied, we preferred estimates from a study that used 'specific' disorder over study that used general mental disorders. If estimates obtained from studies that used both 'specific' disorder from the same cohorts (but still overlap), we used several mutually exclusive rules when we preferred; (1) broad category disorder over narrowly-defined disorder, or (2) estimates from study that used statistical model as opposed to study that provided raw number, or (4) estimates with uncertainty estimates over those without, or (5) estimates with clearly defined 'adjustment' over those not very clear (how many covariates, how to define covariates).

eMethods 4.

Diagnostic instruments and diagnostic criteria for Mood and Anxiety disorders.

We used several diagnostic instruments those were initially symptom-based but the diagnosis was made using either DSM or ICD criteria or any published diagnostic criteria. For example, several studies used WHO CIDI (World Health Organisation Composite International Diagnostic Interview) (Kessler & Ustun, 2004), one of the most notable screening instruments in which dichotomous diagnosis were made according to DSM and/or ICD criteria.

Instruments Abbreviation	Full name	Reference
**ICD**	International Classification of Diseases	1
**DSM**	Diagnostic and Statistical Manual of Mental Disorders	2
**CAMDEX**	Cambridge Mental Disorders of the Elderly Examination	3
**CAPA**	The Child and Adolescent Psychiatric Assessment	4
**CIDI**	Composite International Diagnostic Interview	5
**DIGS**	Diagnostic Interview for Genetic Studies	6
**DIS**	Diagnostic Interview Schedule	7
**GMS-AGECAT**	The Geriatric Mental State-Automated Geriatric Examination for Computer Assisted Taxonomy package	8
**MINI**	Mini-International Neuropsychiatric Interview	9
**PDSQ**	Psychiatric Diagnostic Screening Questionnaire	10
**PHQ**	Patient Health Questionnaire	10
**SCAN**	Schedules for Clinical Assessment in Neuropsychiatry	11
**SCID-I**	Structured Clinical Interview for DSM-IV Axis I Disorders	12

1. World Health Organization. *The ICD-10. Classification of mental and behavioural disorders. Clinical descriptions and diagnostic guidelines.* Geneva: World Health Organization; 1992.
2. American Psychiatric Association. *DSM-IV: Diagnostic and Statistical Manual of Mental Disorders.* 4 ed. Washington,D.C.: The Association; 1994.
3. Logie SA, Murphy B, Brooks DN, Wylie S, Barron ET, Mcculloch J. The Diagnosis of Depression in Patients with Dementia: Use of the Cambridge Mental Disorders of the Elderly Examination (CAMDEX). *Int J Geriatr Psychiatry.* 1992;7:363-368.
4. Angold A, Costello EJ. The Child and Adolescent Psychiatric Assessment (CAPA). *J Am Acad Child Adolesc Psychiatry.* 2000;39(1):39-48.
5. Kessler RC, Ustun TB. The World Mental Health (WMH) Survey Initiative Version of the World Health Organization (WHO) Composite International Diagnostic Interview (CIDI). *Int J Methods Psychiatr Res.* 2004;12(2):93-120.
6. Nurnberger JI, Jr, Blehar MC, Kaufmann CA, et al. Diagnostic interview for genetic studies: Rationale, unique features, and training. *Arch Gen Psychiatry.* 1994;51(11):849-859.
7. Segal Daniel L. Diagnostic Interview Schedule for DSM-IV (DIS-IV). *The Corsini Encyclopedia of Psychology.* 2010.
8. Copeland JR, Dewey Me Fau - Henderson AS, Henderson As Fau - Kay DW, et al. The Geriatric Mental State (GMS) used in the community: replication studies of the computerized diagnosis AGECAT. *Psychol Med.* 1988;18(1):219-223.
9. Sheehan DV, Lecrubier Y, Sheehan KH, et al. The Mini-International Neuropsychiatric Interview (M.I.N.I.): the development and validation of a structured diagnostic psychiatric interview for DSM-IV and ICD-10. *J Clin Psychiatry.* 1998;59 Suppl 20:22-33;quiz 34-57.
10. Rush Jr AJ, First MB, Blacker D. *Handbook of psychiatric measures, 2nd ed.* Arlington, VA, US: American Psychiatric Publishing, Inc.; 2008.
11. World Health Organization. *Schedules for Clinical Assessment in Neuropsychiatry.* Geneva: World Health Organization; 1999.
12. First MB, Gibbon M. *The Structured Clinical Interview for DSM-IV Axis I Disorders (SCID-I) and the Structured Clinical Interview for DSM-IV Axis II Disorders (SCID-II).* Vol 2. Hoboken, NJ, US: John Wiley & Sons Inc; 2004.

eMethods 5. Overlap assessment using *Study-level* and *Estimate-level* filters

SL. No.	Cohort name	Disorder pair for potential overlap	Study ID†	Decision/comment		Filter used
1	BJS (Bremer Jugendstudie)	Mood : Anx	49 , 50 , 171	Keep 49 (50 and 171 to be discarded)<br>ID 49: 1st row label to be replaced with:<br>Any anxiety disorder-Mood disorder	Duplicate study	Study-level filter<br>(171 to go)
2	EDSP (Early Developmental Stages of Psychopathology)	MDD : SoP	6 , 19 , 150	All accepted.<br>ID: 6 & 19 data are prospective but different time period, and<br>ID: 150 CS		None
3		MDD: PD	19 , 164	Keep ID 19 is accepted (164 discarded)<br>All estimates for 164 to be re-entered from Table 1	Better adjustment for estimates	Estimate-level filter
4		MDD : GAD	19 , 141	Keep estimate for ID 19, better adj (141 discarded)	Better adjustment for estimates	Estimate-level filter
5		MDD : SP	19 , 74	Keep estimate for ID 19, better adj (74 discarded)	Better adjustment for estimates	Estimate-level filter
6	AMSTEL (The Amsterdam study of the elderly)	MDE : GAD	85 , 87	Keep 85 (87 to be discarded)	Duplicate study	Study-level filter<br>(87 to go)
7	CCHS 1.2 (The Canadian	MDD : PD	12 , 45	Keep 45, 45 provided ORs	45 provided ORs	Estimate-level filter
8	Community Health Survey)	MDD : AGO	12 , 130	Keep 130, 12 did not provide ORs	130 provided ORs	Estimate-level filter
9	ECA (Epidemiological Catchment Area)	MDD : PD	56 , 96 , 97	Keep 56, better adj (ID 96, 97 discarded)<br>ECA studies: no other overlap so far known	Better adjustment for estimates	Estimate-level filter
10	NSMHWB (National survey of Mental Health and Well-being)	MDD : GAD	36 , 135	135 preferred as estimate is adj as well, against raw number in 36	Better adjustment for estimates against raw number	Estimate-level filter
11		MDD : SoP	63 , 135	63 is preferred as estimates are adjusted with CIs, 135 with p values	Estimates are adjusted with Cis (135 with p values)	Estimate-level filter
12		Dysthymia : SoP	63 , 135	63 is preferred, ditto No. 11	Estimates are adjusted with	Estimate-level filter

					Cis (135 with p values)	
14	CPES (The Christchurch Psychiatry Epidemiology Study)	MDD : PD	96 , 97	Keep 96, 7 country data (97 study to be discarded: same data, same adj, same sample size etc)	Duplicate study	Study-level filter (97 to go)
15	The Korean Epidemiologic Study of Mental Disorders	MDD : PD	96 , 97	Ditto 14, consider ALL (yellow highlighted country data, Nos 28, 29 ditto..)	Estimates are adjusted with Cis (135 with p values)	Study-level filter (97 to go)
16	OHS/MHS (Ontario health survey – mental health supplement)	MDD : GAD	61 , 147	Keep 61 (147 GAD_MDD CANADA rate to be discarded- 1 row only) Yes, OHS/MHS is same	?	Estimate-level filter
17	NCS(National Comorbidity Survey)	SO : MDD (temporally ordered)	105 , 107	Keep 105 over 107 (for SP and MDD-temporal adjusted rates only, 107 did not mention any adj)	No mention of any adjustments in 107	Estimate-level filter
18	NCS	PD : MDD (study 107) PD : MDE (study 104) (temporally ordered)	104 , 107	Keep 104 over 107 (no hierarchy mentioned)	?	Estimate-level filter
19	NCS	GAD : MDE (temporally ordered)	107 , 133	Keep 133 only for GAD and MDD overall, over 107	No mention of any adjustments in 107	Estimate-level filter
20	NCS	MDD : PTSD (LT)	107 , 200	Study 200 reports by sex Study 107 reports total We do not need both, just 1 will do OK	?	Estimate-level filter
21	NCS	MDD: PD (LT) 12m:	107 , 140	Keep 140 over 107 (140-specialized study)	Specialized study on PD is preferred	Estimate-level filter
22	NCS	MDD : GAD (LT)	107 , 174	Keep 174 over 107 (174-specialized study on GAD)	Specialized study on GAD is preferred	Estimate-level filter
23	NCS	SO : MDD SO : DYS SO : Mood (LT)	105 , 132, 107, 163	Keep 132 over 105(132-specialized study)	Specialized study is preferred	Estimate-level filter

24	NCSR (national comorbidity survey replication)	BP : OCD	67 , 119	Keep 67 over 119 (both are specialized studies but 119 BP definition includes subthreshold category, no good)	Specialized study rule applied together with better diagnostic criteria in 67	Estimate-level filter
25		BP : SoP	119 , 163	Keep 163 (ditto….24…same estimate-coin tossing!!)	Specialized study rule applied together with better diagnostic criteria in 24	Estimate-level filter
26	OADP (The Oregon adolescent depression project)	ANX : MDD (temporally ordered)	70 , 175	Keep 70 (bigger sample with both bivariate and multivariate rates available in both direction)<br>Add bivariate rates into the dataset:<br>MDD:ANX=1.81 (1.21-2.70)<br>ANX:MDD=2.32 (1.70-3.16)	Most informative rule (bigger sample with both bivariate and multivariate rates available in both direction)	Study-level filter<br>(175 to go?)
27	The Puerto Rico Study of Psychiatric disorders	MDD : PD	96 , 97	Ditto 14, consider ALL yellow highlighted country data		Study-level filter<br>(97 to go)
28	TPEP (The Taiwan Psychiatric Epidemiological project)	MDD : PD	96 , 97	Ditto 14, consider ALL yellow highlighted country data		Study-level filter<br>(97 to go)
29	NESARC (National epidemiologic survey on alcohol and related conditions)	BP I : ANX (LT)	9 , 58	Keep 58 (both adj and unadj, 12m/LT: bigger sample, complete set)	Most informative rule (bigger sample with both adjusted and unadjusted rates available, complete set)	Estimate-level filter
30	NESARC	BP I : GAD (LT)	28 , 58 , 98, 22	Keep 58 (both adj and unadj, 12m/LT: bigger sample, complete set)<br>ID:22 to be avoided for this as well	Most informative rule (bigger sample with both adjusted and unadjusted rates available, complete set)	Estimate-level filter
34	NESARC	BP II : GAD (LT)	28 , 98	Keep 28, and 98 is by gender M:F.	Overall rates are preferred over sex specific	Estimate-level filter

	NESARC	BP : SpP	9, 58	Keep 58 (both adj and unadj, 12m/LT: bigger sample, complete set)	Most informative rule (bigger sample with both adjusted and unadjusted rates available, complete set)	Estimate-level filter
	NESARC	BP : PD	9, 22, 41, 58	Keep 58 (both adj and unadj, 12m/LT: bigger sample, complete set)	Most informative rule (bigger sample with both adjusted and unadjusted rates available, complete set)	Estimate-level filter
	NESARC	BP : SoP	9, 40, 57, 58	Keep 58 (both adj and unadj, 12m/LT: bigger sample, complete set)	Most informative rule (bigger sample with both adjusted and unadjusted rates available, complete set)	Estimate-level filter
**31**	NESARC	BP I : PTSD (LT)	9 , 136	Keep 9 bigger sample, bigger age range (9 is for 18+, and 136 is for old>60y)	Most informative rule (bigger sample with both adjusted and unadjusted rates available, complete set)	Estimate-level filter
**32**	NESARC	Mood : GAD (LT)	28 , 98	Estimates are very diff The reason is 28 is for BOTH, and 98 is by gender M:F. Keep 28, and discard 98 altogether	Overall rates are preferred over sex specific	Study-level filter (98 to go)
**33**	NESARC	Dysthymia : GAD (LT)	28 , 98	Keep 28, and 98 is by gender M:F.	Overall rates are preferred over sex specific	Estimate-level filter
**35**	NESARC	MDD : GAD (LT)	28 , 31 , 98	Keep 31 because complete set adj and unadj, please entry unadj data from the paper 31 (and 98 is by gender M:F.)	Most informative rule (bigger sample with both adjusted and unadjusted rates available, complete set)	Estimate-level filter, and Study-level filter (98 to go)
**36**	NESARC	MDD : SpP (LT)	31 , 181	Keep 31 (bigger age, complete set) please entry unadj data from	Most informative rule (bigger	Estimate-level filter

				the paper	sample with both adjusted and unadjusted rates available, complete set)	
**37**	NESARC	MDD : PD (12mo)	31,41	Keep 31 (bigger age, complete set) please entry unadj data from the paper	Most informative rule (bigger sample with both adjusted and unadjusted rates available, complete set)	Estimate-level filter

†IDs are generated for internal use; Abbreviations: MDD= Major depressive disorder, MDE= Major depressive episode, BP = Bipolar disorder, ANX=Any Anxiety, SoP = Social phobia, SpP= Specific phobia, PD= Panic disorder, AGO= Agoraphobia (with or without panic disorder), Mood = Any mood disorder, PTSD= Posttraumatic stress disorder, LT = Lifetime, 12mo= 12 months; M = Male, F = Female

eMethods 6. Quality Reporting Scale (QRS)

Broad characteristics of the quality reporting scale include background methodological features (source of data, case definition, sample size, statistical analysis), internal validity or risk of bias (how credible the findings based on the design and apparent conduct of a study), generalizability and external validity or applicability (how well a study addresses the topic under review), and ethical approval with an overall rating. The scale is as follows:

1. Study background described:
   a. Yes = 1
   b. No = 0
2. Study objectives/aims stated:
   a. Yes = 1
   b. No = 0
3. Study design and/or setting described:
   a. Yes = 1
   b. No = 0
4. Source of data (for cases and controls):
   a. Surveys = 2
   b. Registers (including inpatients, outpatient, emergency departments, general practice or other health services) = 1
   c. Others or not stated = 0
5. Diagnostic *criteria* used for case definition:
   a. DSM or ICD (for both conditions) = 2 (For dementia= or NINCDS-ADRDA)
   b. DSM or ICD (for one condition) = 1 (For dementia= or NINCDS-ADRDA)
   c. Other published diagnostic criteria =0
6. Diagnostic *method* for case definition:
   a. Face-to-face structured interview = 3
   b. Systematic review of case note = 2
   c. Diagnosis listed in chart or register = 1
   d. Not specified = 0
7. Representative of sample and generalisability:
   a. Any text about generalisability = 1
   b. No description/uncertain = 0
8. Presentation of comorbidity estimate: risk estimate presented: Derived estimate presented (HR, RR, OR etc):
   a. Yes = 1
   b. No = 0 (No derived estimate but raw data provided)
9. Adjustments for age and sex used:
   a. Yes = 1
   b. No = 0
10. Text about any other potential sample bias (i.e. in addition to age and sex, selection bias, conduct of the study; any text or description):
   a. Yes = 1
   b. No = 0
11. Statistical analysis: Description of statistical methods used to derived comorbidity estimate (any model used eg., Cox regression model):
   a. Any model used = 1
   b. No = 0

12. Ethics approval described:
    a. Yes = 1
    b. No = 0

eMethods 7. Broad categories of names used for major disorders

Broadly-defined disorders	Disorder terms used by different authors
**Mood Disorder (MOOD)**	Mood disorder, any mood disorder, affective disorder, any affective disorders, depression and dysthymia, depression and dysthymic disorder
**Bipolar Disorder (BIPOLAR)**	Bipolar I, bipolar II, any bipolar disorder, and bipolar affective disorder, bipolar disorder
**Depressive Disorder (DEP)**	Depression, major depressive disorder, major depressive episode, depressive episode, single depressive episode, depressive disorder, current depression/depressive disorder /major depression, unipolar depression, depressive syndrome
**Dysthymic Disorder (DYS)**	Any dysthymia, dysthymic disorder, dysthymia
**Anxiety Disorders (ANX)**	Any anxiety disorders, anxiety disorder, other anxiety disorder, clinical anxiety disorder, neurotic disorder, overanxious disorder

Supplementary Tables (etable)

etable 1. Characteristics of the included studies presenting temporally-ordered estimates (n=36)

EndNote ID	First author, year, study location	Sample size	Study design	Mood disorder type†	Anxiety disorder type‡	Diagnostic criteria	Risk estimate type	Control variables
**149**	Acarturk, 2009, the Netherlands	7076	PC	DEP, DYS, BIPOLAR	SO	DSM-III-R	IRR, aIRR	age, sex, marital status and urbanization
**7**	Beesdo, 2007, Germany§	3021	PC	MOOD	SO	DSM-IV	aOR	age,sex
**150**	Beesdo, 2010, Germany	3021	PC	DEP	GAD, ANX	DSM-IV	HR, aHR	mental disorders
**152**	Bittner, 2004, Germany	3021	PC	DEP	GAD, PD, AGO, SO, SP, ANX	DSM-IV	aOR	age, sex, mental disorders
**151**	Bittner, 2007, USA	1008	PC	DEP	SAD, GAD, SO	DSM-IV	OR, aOR	childhood anxiety disorders
**153**	Bromet, 1998, USA	8098	CS	MOOD	PTSD	DSM-III-R	aOR	age, person-year, marital status, education status, interaction of marital status and education
**13**	Cederlof, 2015, Sweden§	19814	CC	BIPOLAR	OCD	ICD	RR	
**154**	Chang, 2017, Taiwan	76545	PC	DEP, BIPOLAR	PTSD	ICD-9	HR, aHR	age, sex
**155**	Chou, 2011, USA	34653	PC	BIPOLAR	GAD	DSM-IV	aOR	age, time
**134**	Costello, 2003, USA§	6674	PC	DEP	ANX	DSM-IV	OR, aOR	mental disorders
**156**	de Graaf, 2004, the Netherlands	7076	PC	MOOD	ANX	DSM-III-R	OR€	-
**33**	Goodwin, 2002, USA	15849	PC	DEP	AGO, OCD, SP	DSM-III	aOR	age, sex, race, marital status, education, mental disorders
**174**	Goodwin, 2004, Germany§	3021	PC	DYS, BIPOLAR, MOOD	PD	DSM-IV	aOR	age, sex

**158**	Grant, 2009, USA	34653	PC	DEP, DYS, BIPOLAR	GAD, PTSD, PD, SO, SP,	DSM-IV	aOR	sociodemographic characteristics, mental disorders
**140**	Jaffee, 2002, New Zealand[§]	1037	PC	DEP	ANX	DSM	aOR	sex
**159**	Johnson, 2000, USA	717	PC	BIPOLAR	ANX	DSM-IV	OR	-
**46**	Kang, 2016, South Korea[§]	1204	PC	DEP	ANX	AGECAT	OR, aOR	age, sex, depression, insomnia
**145**	Kessler, 1996, USA[§]	8098	CS	DEP	GAD, AGO, SP, SO, PD, PTSD, ANX	DSM-III-R	OR, aOR	mental disorders, number of years since onset
**161**	Kessler, 1998, USA	8098	CS	DEP	PD	DSM-III-R	aOR	age, sex, race, person-year
**48**	Kessler, 1999, USA[§]	8098	CS	DEP, DYS, BIPOLAR, MOOD	SO	DSM-III-R	aOR	person-year, age, sex, race
**47**	Kessler, 2002a, Multiple countries[§]	20189	CS	DEP, DYS, MANIA, MOOD	GAD	DSM-III-R	aOR	person-year, sex, cohort, country
**160**	Kessler, 2008, USA	5001	PC	DEP	GAD	DSM-IV	aOR	age, sex, race, person-year
**162**	Keyl, 1990, USA	12823	CC	DEP	PD	DSM-III	OR, aOR	age, sex, race, marital status, occupational prestige
**163**	Kim-Cohen, 2003, New Zealand	1037	PC	DEP	ANX	DSM	aOR	sex
**164**	Lieb, 2016, Germany	2210	PC	BIPOLAR, DYS	SP	DSM-IV	aRR	age, mental disorders
**165**	Mathew, 2011, USA	1709	PC	DEP	ANX	DSM-III-R	HR, aHR	sex, family social support, friend social support, worry, loneliness, emotional reliance
**166**	Meier, 2015, Denmark	3380059	PC	DEP	GAD, OCD, AGO, PD, PTSD, SO, SP	ICD-8	aIRR	calendar year, age, maternal and paternal age, sex, psychiatric family history, first psychiatric hospital contact due to any specific ANX, place of residence

								at time of birth,the interaction of age with sex
**167**	Olfson, 2017, USA	34653	PC	DEP	GAD, PD, SO, SP, ANX	DSM-IV	OR, aOR	age, sex, race, marital status, employment, education, mental component summary score, physical component summary score, lifetime psychiatric disorders at Wave 1.
**168**	Pine, 1998, USA	1141	PC	DEP	GAD, SO, SP, OVRANX	DSM-III-R	OR, aOR	age, race, social class, sex
**122**	Ritchie, 2013, France§	1968	PC	DEP	AGO	DSM-IV	aOR	age, time
**169**	Ruscio, 2010, USA§	9282	CS	DEP, DYS, BIPOLAR, MOOD	OCD	DSM-IV	aOR	age, sex, race
**170**	Schoevers, 2000, the Netherlands	4051	PC	DEP	ANX	GMS-AGECAT	OR	-
**171**	Stein, 2001, Germany	3021	PC	MOOD	SO	DSM-IV	aOR	age, sex
**91**	Tsuchiya, 2009, Japan§	2437	CS	DEP	SO, AGO, SP	DSM-IV	aHR	sex, birth-cohort, number of other ANXs, marital status, education
**172**	Woodward, 2001, New Zealand	1265	PC	DEP	ANX	DSM-III-R	OR€	-
**101**	Zhang, 2015, France	1711	PC	DEP	GAD	DSM-IV	aHR	age, sex, past GAD

Abbreviations: study design (CS = cross-sectional studies; PC = prospective cohort studies; CC = case-control studies), MOOD (MOOD = broadly-defined mood disorder; DEP = broadly-defined depressive disorder; BIPOLAR = broadly-defined bipolar disorder; DYS = broadly-defined dysthymic disorder), ANX (GAD = generalized anxiety disorder; PTSD = post-traumatic stress disorder; OCD = obsessive compulsive disorder; AGO = AGO with or without PD; ANX = broadly-defined anxiety disorder; PD = PD; SO = SO or social ANX; SP = SP or SP; SAD= separation anxiety disorder; OVRANX = overanxious disorder), Risk type (OR = Odds Ratio; RR = Risk Ratio; aRR = Adjusted Risk Ratio; aOR = Adjusted Odds Ratio; IRR = Incidence Rate Ratio; aIRR = Adjusted Incidence Rate Ratio; HR = Hazard Ratio; aHR = Adjusted Hazard Ratio)

†, ‡Disorders assessed in the study. We did not extract comorbidity estimates within the same disorder class.

§These studies have both temporally and non-temporally ordered estimates; €These are extracted from 2 X 2 table

eTable 2. Bi-directional pooled estimates for broadly-defined mood disorder (MOOD) and anxiety disorders

Prior disorder type	Later disorder type	Study reporting crude estimates				Study reporting adjusted estimates			
		**n**	**Pooled OR (95% CI)**	**I^2**	**P-value**	**n**	**Pooled AOR (95% CI)**	**I^2**	**P-value**
MOOD	Agoraphobia	0	-†	-	-	0	-†	-	-
Agoraphobia	MOOD	0	-†	-	-	1	-†	-	-
MOOD	OCD	0	-†	-	-	0	-†	-	-
OCD	MOOD	0	-†	-	-	2	-†	-	-
MOOD	GAD	0	-†	-	-	0	-†	-	-
GAD	MOOD	0	-†	-	-	1	-†	-	-
MOOD	Panic disorder	0	-†	-	-	0	-†	-	-
Panic disorder	MOOD	0	-†	-	-	2	-†	-	-
MOOD	PTSD	0	-†	-	-	1	-†	-	-
PTSD	MOOD	0	-†	-	-	1	-†	-	-
MOOD	Social phobia	0	-†	-	-	0	-†	-	-
Social phobia	MOOD	0	-†	-	-	4	2.4 (1.6-3.5)	92.5%	<.001
MOOD	Specific phobia	0	-†	-	-	0	-†	-	-
Specific phobia	MOOD	0	-†	-	-	1	-†	-	-
MOOD	ANX	1	-†	-	-	0	-†	-	-
ANX	MOOD	0	-†	-	-	0	-†	-	-

†,‡Estimates were not pooled if the number of studies are less than 3 for that disorder pair; BIPOLAR = broadly-defined bipolar disorder; GAD = generalized anxiety disorder; ANX = broadly-defined anxiety disorder; PTSD = post-traumatic stress disorder; OCD = obsessive compulsive disorder

eTable 3. Bi-directional pooled estimates for broadly-defined depressive disorder (DEP) and anxiety disorders

Prior disorder type	Later disorder type	Study reporting crude estimates				Study reporting adjusted estimates			
		n	Pooled OR (95% CI)	I^2	P-value	n	Pooled AOR (95% CI)	I^2	P-value
DEP	Agoraphobia	0	-†	-	-	1	-†	-	-
Agoraphobia	DEP	1	-†	-	-	5	1.8 (1.0-3.3)	97.0%	<.001
DEP	OCD	0	-†	-	-	0	-†	-	-
OCD	DEP	0	-†	-	-	3	2.3 (0.7-7.3)	99.5%	<.001
DEP	GAD	2	-†	-	-	6	2.6 (2.2-3.1)	26.2%	0.18
GAD	DEP	1	-†	-	-	6	2.0 (1.2-3.2)	83.2%	<.001
DEP	Panic disorder	0	-†	-	-	0	-†	-	-
Panic disorder	DEP	0	-†	-	-	4	1.5 (1.0-2.4)	57.5%	0.07
DEP	PTSD	0	-†	-	-	0	-†	-	-
PTSD	DEP	0	-†	-	-	0	-†	-	-
DEP	Social phobia	3	7.3 (6.2-8.7)	0.0%	0.13	2	-†	-	-
Social phobia	DEP	3	2.5 (2.1-3.1)	0.0%	0.57	6	1.7 (1.1-2.7)	88.8%	<.001
DEP	Specific phobia	2	-†	-	-	1	-†	-	-
Specific phobia	DEP	2	-†	-	-	6	1.6 (0.9-2.6)	96.0%	<.001
DEP	ANX	5	3.1 (2.0-4.7)	81.3%	<.001	6	2.1 (1.5-2.8)	67.0%	0.03
ANX	DEP	6	2.4 (2.0-2.9)	46.7%	0.11	7	2.1 (1.8-2.5)	28.5%	0.24

†Estimates were not pooled if the number of studies are less than 3 for that disorder pair; BIPOLAR = broadly-defined bipolar disorder; GAD = generalized anxiety disorder; ANX = broadly-defined anxiety disorder; PTSD = post-traumatic stress disorder; OCD = obsessive compulsive disorder.

References (eReferences)

eReference 1. Table 1. Studies presenting lifetime and period prevalence estimates (147 studies)

Adam, Y., Meinlschmidt, G., Gloster, A. T., & Lieb, R. (2012). Obsessive-compulsive disorder in the community: 12-month prevalence, comorbidity and impairment. *Social Psychiatry and Psychiatric Epidemiology, 47*, 339-349. doi:10.1007/s00127-010-0337-5

Alonso, J., Angermeyer, M. C., Bernert, S., Bruffaerts, R., Brugha, T. S., Bryson, H., . . . Vollebergh, W. A. M. (2004). 12-Month comorbidity patterns and associated factors in Europe: results from the European Study of the Epidemiology of Mental Disorders (ESEMeD) project. *Acta Psychiatrica Scandinavica, 109*, 28-37. doi:10.1111/j.1600-0047.2004.00328.x

Alvarenga, P. G., do Rosario, M. C., Cesar, R. C., Manfro, G. G., Moriyama, T. S., Bloch, M. H., . . . Miguel, E. C. (2016). Obsessive-compulsive symptoms are associated with psychiatric comorbidities, behavioral and clinical problems: a population-based study of Brazilian school children. *European Child and Adolescent Psychiatry, 25*, 175-182. doi:10.1007/s00787-015-0723-3

Andrews, G., Slade, T., & Issakidis, C. (2002). Deconstructing current comorbidity: data from the Australian National Survey of Mental Health and Well-Being. *British Journal of Psychiatry, 181*, 306-314. doi:10.1192/bjp.181.4.306

Angst, J., Gamma, A., Endrass, J., Hantouche, E., Goodwin, R., Ajdacic, V., . . . Rossler, W. (2005). Obsessive-compulsive syndromes and disorders: significance of comorbidity with bipolar and anxiety syndromes. *European Archives of Psychiatry and Clinical Neuroscience, 255*, 65-71. doi:10.1007/s00406-005-0576-8

Arillo Crespo, A., Aguinaga Ontoso, I., & Guillen Grima, F. (1998). [Prevalence of mental diseases in women of an urban area]. *Atencion Primarie, 21*, 265-269.

Autonell, J., Vila, F., Pinto-Meza, A., Vilagut, G., Codony, M., Almansa, J., . . . Haro, J. M. (2007). [One year prevalence of mental disorders comorbidity and associated socio-demographic risk factors in the general population of Spain. Results of the ESEMeD-Spain study]. *Actas Españolas de Psiquiatría, 35 Suppl 2*, 4-11. Retrieved from https://translate.googleusercontent.com/translate_f

Beekman, A. T., de Beurs, E., van Balkom, A. J., Deeg, D. J., van Dyck, R., & van Tilburg, W. (2000). Anxiety and depression in later life: Co-occurrence and communality of risk factors. *American Journal of Psychiatry, 157*, 89-95. doi:10.1176/ajp.157.1.89

Beesdo, K., Bittner, A., Pine, D. S., Stein, M. B., Hofler, M., Lieb, R., & Wittchen, H. U. (2007). Incidence of social anxiety disorder and the consistent risk for secondary depression in the first three decades of life. *Archives of General Psychiatry, 64*, 903-912. doi:10.1001/archpsyc.64.8.903

Biederman, J., Petty, C., Faraone, S. V., Hirshfeld-Becker, D. R., Henin, A., Pollack, M. H., & Rosenbaum, J. F. (2005). Patterns of comorbidity in panic disorder and major depression: findings from a nonreferred sample. *Depression and Anxiety, 21*, 55-60. doi:10.1002/da.20055

Blanco, C., Compton, W. M., Saha, T. D., Goldstein, B. I., Ruan, W. J., Huang, B., & Grant, B. F. (2017). Epidemiology of DSM-5 bipolar I disorder: Results from the National Epidemiologic Survey on Alcohol and Related Conditions - III. *Journal of Psychiatric Research, 84*, 310-317. doi:10.1016/j.jpsychires.2016.10.003

Boyd, J. H., Burke, J. D., Jr., Gruenberg, E., Holzer, C. E., III, Rae, D. S., George, L. K., . . . Nestadt, G. (1984). Exclusion Criteria of DSM-III: A Study of Co-occurrence of Hierarchy-Free Syndromes. *Archives of General Psychiatry, 41*, 983-989. doi:10.1001/archpsyc.1984.01790210065008

Bromet, E. J., Gluzman, S. F., Paniotto, V. I., Webb, C. P. M., Tintle, N. L., Zakhozha, V., . . . Schwartz, J. E. (2005). Epidemiology of psychiatric and alcohol disorders in Ukraine. *Social Psychiatry and Psychiatric Epidemiology, 40*, 681-690. doi:10.1007/s00127-005-0927-9

Bruce, S. E., Weisberg, R. B., Dolan, R. T., Machan, J. T., Kessler, R. C., Manchester, G., . . . Keller, M. B. (2001). Trauma and Posttraumatic Stress Disorder in Primary Care Patients. *Primary Care Companion to the*

Journal of Clinical Psychiatry, 3, 211-217. Retrieved from https://www.ncbi.nlm.nih.gov/pubmed/15014575

https://www.ncbi.nlm.nih.gov/pmc/PMC181217/

Cairney, J., Corna, L. M., Veldhuizen, S., Herrmann, N., & Streiner, D. L. (2008). Comorbid depression and anxiety in later life: patterns of association, subjective well-being, and impairment. *American Journal of Geriatric Psychiatry, 16*, 201-208. doi:10.1097/JGP.0b013e3181602a4a

Cairney, J., McCabe, L., Veldhuizen, S., Corna, L. M., Streiner, D., & Herrmann, N. (2007). Epidemiology of Social Phobia in Later Life. *American Journal of Geriatric Psychiatry, 15*, 224-233. doi:https://doi.org/10.1097/01.JGP.0000235702.77245.46

Carter, R. M., Wittchen, H. U., Pfister, H., & Kessler, R. C. (2001). One-year prevalence of subthreshold and threshold DSM-IV generalized anxiety disorder in a nationally representative sample. *Depression and Anxiety, 13*, 78-88.

Cederlof, M., Lichtenstein, P., Larsson, H., Boman, M., Ruck, C., Landen, M., & Mataix-Cols, D. (2015). Obsessive-Compulsive Disorder, Psychosis, and Bipolarity: A Longitudinal Cohort and Multigenerational Family Study. *Schizophrenia Bulletin, 41*, 1076-1083. doi:10.1093/schbul/sbu169

Chartier, M. J., Walker, J. R., & Stein, M. B. (2003). Considering comorbidity in social phobia. *Social Psychiatry and Psychiatric Epidemiology, 38*, 728-734. doi:10.1007/s00127-003-0720-6

Chavira, D. A., Stein, M. B., Bailey, K., & Stein, M. T. (2004). Comorbidity of generalized social anxiety disorder and depression in a pediatric primary care sample. *Journal of Affective Disorders, 80*, 163-171. doi:10.1016/s0165-0327(03)00103-4

Chen, Y., Bennett, D., Clarke, R., Guo, Y., Yu, C., Bian, Z., . . . Chen, Z. (2017). Patterns and correlates of major depression in Chinese adults: a cross-sectional study of 0.5 million men and women. *Psychological Medicine, 47*, 958-970. doi:10.1017/s0033291716002889

Chen, Y. W., & Dilsaver, S. C. (1995). Comorbidity of panic disorder in bipolar illness: evidence from the Epidemiologic Catchment Area Survey. *American Journal of Psychiatry, 152*, 280-282. doi:10.1176/ajp.152.2.280

Chou, K.-L. (2009). Specific Phobia in Older Adults: Evidence From the National Epidemiologic Survey on Alcohol and Related Conditions. *American Journal of Geriatric Psychiatry, 17*, 376-386. doi:https://doi.org/10.1097/JGP.0b013e3181943214

Chou, K. L. (2009). Social anxiety disorder in older adults: evidence from the National Epidemiologic Survey on alcohol and related conditions. *Journal of Affective Disorders, 119*, 76-83. doi:10.1016/j.jad.2009.04.002

Chou, K. L. (2010). Panic disorder in older adults: evidence from the national epidemiologic survey on alcohol and related conditions. *International Journal of Geriatric Psychiatry, 25*, 822-832. doi:10.1002/gps.2424

Choy, Y., Fyer, A. J., & Goodwin, R. D. (2007). Specific phobia and comorbid depression: a closer look at the National Comorbidity Survey data. *Comprehensive Psychiatry, 48*, 132-136. Retrieved from http://search.ebscohost.com/login.aspx?direct=true&db=cin20&AN=106200452&site=ehost-live

https://ac.els-cdn.com/S0010440X06001349/1-s2.0-S0010440X06001349-main.pdf?_tid=1fd90ceb-466c-4530-bf21-01a1131da900&acdnat=1540420573_3254cd4850537cc2473196cfbdad5e7e

Chuan, S. K., Kumar, R., Matthew, N., Heok, K. E., & Pin, N. T. (2008). Subsyndromal depression in old age: clinical significance and impact in a multi-ethnic community sample of elderly Singaporeans. *International Psychogeriatrics, 20*, 188-200. doi:10.1017/s1041610207006187

Copeland, W. E., Shanahan, L., Erkanli, A., Costello, E. J., & Angold, A. (2013). Indirect comorbidity in childhood and adolescence. *Frontiers in Psychiatry, 4*, 144. doi:10.3389/fpsyt.2013.00144

Corna, L. M., Cairney, J., Herrmann, N., Veldhuizen, S., McCabe, L., & Streiner, D. (2007). Panic disorder in later life: results from a national survey of Canadians. *International Psychogeriatrics, 19*, 1084-1096. doi:10.1017/s1041610207004978

Costello, E. J., Mustillo, S., Erkanli, A., Keeler, G., & Angold, A. (2003). Prevalence and Development of Psychiatric Disorders in Childhood and Adolescence. *Archives of General Psychiatry, 60*, 837-844. doi:10.1001/archpsyc.60.8.837

Coyne, J. C., Fechner-Bates, S., & Schwenk, T. L. (1994). Prevalence, nature, and comorbidity of depressive disorders in primary care. *General Hospital Psychiatry, 16*, 267-276. Retrieved from https://deepblue.lib.umich.edu/bitstream/handle/2027.42/31474/0000396.pdf?sequence=1

Cyranowski, J. M., Schott, L. L., Kravitz, H. M., Brown, C., Thurston, R. C., Joffe, H., . . . Bromberger, J. T. (2012). Psychosocial features associated with lifetime comorbidity of major depression and anxiety disorders among a community sample of mid-life women: the SWAN mental health study. *Depression and Anxiety, 29*, 1050-1057. doi:10.1002/da.21990

Davidson, J. R. T., Hughes, D., Blazer, D. G., & George, L. K. (1991). Post-traumatic stress disorder in the community: an epidemiological study. *Psychological Medicine, 21*, 713-721. doi:10.1017/S0033291700022352

de Graaf, R., Bijl, R. V., Smit, F., Vollebergh, W. A. M., & Spijker, J. (2002). Risk Factors for 12-Month Comorbidity of Mood, Anxiety, and Substance Use Disorders: Findings From the Netherlands Mental Health Survey and Incidence Study. *American Journal of Psychiatry, 159*, 620-629. doi:10.1176/appi.ajp.159.4.620

de Graaf, R., Bijl, R. V., Spijker, J., Beekman, A. T., & Vollebergh, W. A. (2003). Temporal sequencing of lifetime mood disorders in relation to comorbid anxiety and substance use disorders--findings from the Netherlands Mental Health Survey and Incidence Study. *Social Psychiatry and Psychiatric Epidemiology, 38*, 1-11. doi:10.1007/s00127-003-0597-4

Depla, M. F. I. A., ten Have, M. L., van Balkom, A. J. L. M., & de Graaf, R. (2008). Specific fears and phobias in the general population: Results from the Netherlands Mental Health Survey and Incidence Study (NEMESIS). *Social Psychiatry and Psychiatric Epidemiology, 43*, 200-208. doi:10.1007/s00127-007-0291-z

Douglass, H. M., Moffitt, T. E., Dar, R., McGee, R., & Silva, P. (1995). Obsessive-compulsive disorder in a birth cohort of 18-year-olds: prevalence and predictors. *Journal of the American Academy of Child and Adolescent Psychiatry, 34*, 1424-1431. doi:10.1097/00004583-199511000-00008

Essau, C. A. (2003). Comorbidity of anxiety disorders in adolescents. *Depression and Anxiety, 18*, 1-6. doi:10.1002/da.10107

Essau, C. A., Conradt, J., & Petermann, F. (1999). Frequency of panic attacks and panic disorder in adolescents. *Depression and Anxiety, 9*, 19-26. doi:10.1002/(SICI)1520-6394(1999)9:1<19::AID-DA3>3.0.CO;2-#

Faravelli, C., Abrardi, L., Bartolozzi, D., Cecchi, C., Cosci, F., D'Adamo, D., . . . Rosi, S. (2004). The Sesto Fiorentino Study: Background, Methods and Preliminary Results. *Psychotherapy and Psychosomatics, 73*, 216-225. doi:10.1159/000077740

Faravelli, C., Abrardi, L., Bartolozzi, D., Cecchi, C., Cosci, F., D'Adamo, D., . . . Rosi, S. (2004). The Sesto Fiorentino Study: Point and One-Year Prevalences of Psychiatric Disorders in an Italian Community Sample Using Clinical Interviewers. *Psychotherapy and Psychosomatics, 73*, 226-234. doi:10.1159/000077741

Fehm, L., Beesdo, K., Jacobi, F., & Fiedler, A. (2008). Social anxiety disorder above and below the diagnostic threshold: prevalence, comorbidity and impairment in the general population. *Social Psychiatry and Psychiatric Epidemiology, 43*, 257-265. doi:10.1007/s00127-007-0299-4

Fineberg, N. A., Hengartner, M. P., Bergbaum, C., Gale, T., Rossler, W., & Angst, J. (2013). Lifetime comorbidity of obsessive-compulsive disorder and sub-threshold obsessive-compulsive symptomatology in the community: impact, prevalence, socio-demographic and clinical characteristics. *International Journal of Psychiatry in Clinical Practice, 17*, 188-196. doi:10.3109/13651501.2013.777745

Fleitlich-Bilyk, B., & Goodman, R. (2004). Prevalence of Child and Adolescent Psychiatric Disorders in Southeast Brazil. *Journal of the American Academy of Child and Adolescent Psychiatry, 43*, 727-734. doi:https://doi.org/10.1097/01.chi.0000120021.14101.ca

Ford, T., Goodman, R., & Meltzer, H. (2003). The British Child and Adolescent Mental Health Survey 1999: The Prevalence of DSM-IV Disorders. *Journal of the American Academy of Child and Adolescent Psychiatry, 42*, 1203-1211. doi:https://doi.org/10.1097/00004583-200310000-00011

Fung, A. W., Chan, W. C., Wong, C. S., Chen, E. Y., Ng, R. M., Lee, E. H., . . . Lam, L. C. (2017). Prevalence of anxiety disorders in community dwelling older adults in Hong Kong. *International Psychogeriatrics, 29*, 259-267. doi:10.1017/s1041610216001617

Gabilondo, A., Rojas-Farreras, S., Vilagut, G., Haro, J. M., Fernandez, A., Pinto-Meza, A., & Alonso, J. (2010). Epidemiology of major depressive episode in a southern European country: results from the ESEMeD-Spain project. *Journal of Affective Disorders, 120*, 76-85. doi:10.1016/j.jad.2009.04.016

Glaesmer, H., Kaiser, M., Braehler, E., Freyberger, H. J., & Kuwert, P. (2012). Posttraumatic stress disorder and its comorbidity with depression and somatisation in the elderly - a German community-based study. *Aging & Mental Health, 16*, 403-412. doi:10.1080/13607863.2011.615740

Goncalves, D. C., Pachana, N. A., & Byrne, G. J. (2011). Prevalence and correlates of generalized anxiety disorder among older adults in the Australian National Survey of Mental Health and Well-Being. *Journal of Affective Disorders, 132*, 223-230. doi:10.1016/j.jad.2011.02.023

Goodwin, R. D., Lieb, R., Hoefler, M., Pfister, H., Bittner, A., Beesdo, K., & Wittchen, H. U. (2004). Panic attack as a risk factor for severe psychopathology. *American Journal of Psychiatry, 161*, 2207-2214. doi:10.1176/appi.ajp.161.12.2207

Grabe, H. J., Meyer, C., Hapke, U., Rumpf, H. J., Freyberger, H. J., Dilling, H., & John, U. (2001). Lifetime-comorbidity of obsessive-compulsive disorder and subclinical obsessive-compulsive disorder in Northern Germany. *European Archives of Psychiatry and Clinical Neuroscience, 251*, 130-135.

Grant, B. F., Hasin, D. S., Blanco, C., Stinson, F. S., Chou, S. P., Goldstein, R. B., . . . Huang, B. (2005). The epidemiology of social anxiety disorder in the United States: results from the National Epidemiologic Survey on Alcohol and Related Conditions. *Journal of Clinical Epidemiology, 66*, 1351-1361. Retrieved from http://www.psychiatrist.com/jcp/article/pages/2005/v66n11/v66n1102.aspx

Grant, B. F., Hasin, D. S., Stinson, F. S., Dawson, D. A., June Ruan, W., Goldstein, R. B., . . . Huang, B. (2005). Prevalence, correlates, co-morbidity, and comparative disability of DSM-IV generalized anxiety disorder in the USA: results from the National Epidemiologic Survey on Alcohol and Related Conditions. *Psychological Medicine, 35*, 1747-1759. doi:10.1017/s0033291705006069

Grant, B. F., Stinson, F. S., Hasin, D. S., Dawson, D. A., Chou, S. P., Ruan, W. J., & Huang, B. (2005). Prevalence, correlates, and comorbidity of bipolar I disorder and axis I and II disorders: results from the National Epidemiologic Survey on Alcohol and Related Conditions. *Journal of Clinical Epidemiology, 66*, 1205-1215. Retrieved from http://www.psychiatrist.com/jcp/article/pages/2005/v66n10/v66n1001.aspx

Gratzer, D., Levitan, R. D., Sheldon, T., Toneatto, T., Rector, N. A., & Goering, P. (2004). Lifetime rates of alcoholism in adults with anxiety, depression, or co-morbid depression/anxiety: a community survey of Ontario. *Journal of Affective Disorders, 79*, 209-215. doi:https://doi.org/10.1016/S0165-0327(02)00355-5

Grenier, S., Schuurmans, J., Goldfarb, M., Preville, M., Boyer, R., O'Connor, K., . . . Hudon, C. (2011). The epidemiology of specific phobia and subthreshold fear subtypes in a community-based sample of older adults. *Depression and Anxiety, 28*, 456-463. doi:10.1002/da.20812

Gureje, O., Uwakwe, R., Oladeji, B., Makanjuola, V. O., & Esan, O. (2010). Depression in adult Nigerians: results from the Nigerian Survey of Mental Health and Well-being. *Journal of Affective Disorders, 120*, 158-164. doi:10.1016/j.jad.2009.04.030

Hasin, D. S., Goodwin, R. D., Stinson, F. S., & Grant, B. F. (2005). Epidemiology of major depressive disorder: results from the National Epidemiologic Survey on Alcoholism and Related Conditions. *Archives of General Psychiatry, 62*, 1097-1106. doi:10.1001/archpsyc.62.10.1097

Hauffa, R., Rief, W., Brähler, E., Martin, A., Mewes, R., & Glaesmer, H. (2011). Lifetime traumatic experiences and posttraumatic stress disorder in the German population: results of a representative population survey. *Journal of Nervous and Mental Disease, 199*, 934-939. Retrieved from http://search.ebscohost.com/login.aspx?direct=true&db=cin20&AN=108208333&site=ehost-live

He, Y. L., Ma, H., Zhang, L., Liu, Z. N., Jia, F. J., & Zhang, M. Y. (2009). [A cross-sectional survey of the prevalence of depressive-anxiety disorders among general hospital outpatients in five cities in China]. *Chinese Journal of Internal Medicine, 48*, 748-751.

Hecht, H., von Zerssen, D., & Wittchen, H. U. (1990). Anxiety and depression in a community sample: the influence of comorbidity on social functioning. *Journal of Affective Disorders, 18*, 137-144. Retrieved from https://ac.els-cdn.com/016503279090070O/1-s2.0-016503279090070O-main.pdf?_tid=c9d38f12-1e00-4420-b2b8-8c170b8b6605&acdnat=1540420771_2843747c9059354354132ad03a408de7

Hek, K., Tiemeier, H., Newson, R. S., Luijendijk, H. J., Hofman, A., & Mulder, C. L. (2011). Anxiety disorders and comorbid depression in community dwelling older adults. *International Journal of Methods in Psychiatric Research, 20*, 157-168. doi:10.1002/mpr.344

Hunt, C., Issakidis, C., & Andrews, G. (2002). DSM-IV generalized anxiety disorder in the Australian National Survey of Mental Health and Well-Being. *Psychological Medicine, 32*, 649-659. doi:10.1017/S0033291702005512

Jaffee, S. R., Moffitt, T. E., Caspi, A., Fombonne, E., Poulton, R., & Martin, J. (2002). Differences in Early Childhood Risk Factors for Juvenile-Onset and Adult-Onset Depression. *Archives of General Psychiatry, 59*, 215-222. doi:10.1001/archpsyc.59.3.215

Jeon, H. J., Suh, T., Lee, H. J., Hahm, B. J., Lee, J. Y., Cho, S. J., . . . Cho, M. J. (2007). Partial versus full PTSD in the Korean community: prevalence, duration, correlates, comorbidity, and dysfunctions. *Depression and Anxiety, 24*, 577-585. doi:10.1002/da.20270

Kang, H. J., Bae, K. Y., Kim, S. W., Shin, I. S., Yoon, J. S., & Kim, J. M. (2016). Anxiety symptoms in Korean elderly individuals: a two-year longitudinal community study. *International Psychogeriatrics, 28*, 423-433. doi:10.1017/s1041610215001301

Kashani, J. H., & Orvaschel, H. (1988). Anxiety disorders in mid-adolescence: A community sample. *American Journal of Psychiatry, 145*, 960.

Katona, C. L. E., Manela, M. V., & Livingston, G. A. (1997). Comorbidity with depression in older people: The Islington Study. *Aging & Mental Health, 1*, 57-62. doi:10.1080/13607869757380

Kawakami, N., Shimizu, H., Haratani, T., Iwata, N., & Kitamura, T. (2004). Lifetime and 6-month prevalence of DSM-III-R psychiatric disorders in an urban community in Japan. *Psychiatry Research, 121*, 293-301. doi:https://doi.org/10.1016/S0165-1781(03)00239-7

Kessler, R. C., Andrade, L. H., Bijl, R. V., Offord, D. R., Demler, O. V., & Stein, D. J. (2002). The effects of co-morbidity on the onset and persistence of generalized anxiety disorder in the ICPE surveys. *Psychological Medicine, 32*, 1213-1225. doi:10.1017/s0033291702006104

Kessler, R. C., Berglund, P. A., Dewit, D. J., Bedirhan Üstün, T., Wang, P. S., & Wittchen, H.-U. (2002). Distinguishing generalized anxiety disorder from major depression: prevalence and impairment from current pure and comorbid disorders in the US and Ontario. *International Journal of Methods in Psychiatric Research, 11*, 99-111. doi:10.1002/mpr.128

Kessler, R. C., DuPont, R. L., Berglund, P., & Wittchen, H. U. (1999). Impairment in pure and comorbid generalized anxiety disorder and major depression at 12 months in two national surveys. *American Journal of Psychiatry, 156*, 1915-1923. doi:10.1176/ajp.156.12.1915

Kessler, R. C., Nelson, C. B., McGonagle, K. A., Liu, J., Swartz, M., & Blazer, D. G. (1996). Comorbidity of DSM-III-R major depressive disorder in the general population: results from the US National Comorbidity Survey. *British Journal of Psychiatry. Supplement, 30*, 17-30.

Kessler, R. C., Rubinow, D. R. H., C. Abelson, J. M., & Zhao, S. (1997). The epidemiology of DSM-III-R bipolar I disorder in a general population survey. *Psychological Medicine, 27*, 1079-1089. doi:10.1017/S0033291797005333

Kessler, R. C., Sonnega, A., Bromet, E., Hughes, M., & Nelson, C. B. (1995). Posttraumatic Stress Disorder in the National Comorbidity Survey. *Archives of General Psychiatry, 52*, 1048-1060. doi:10.1001/archpsyc.1995.03950240066012

Kessler, R. C., Stang, P., Wittchen, H. U., Stein, M., & Walters, E. E. (1999). Lifetime co-morbidities between social phobia and mood disorders in the US National Comorbidity Survey. *Psychological Medicine, 29*, 555-567.

Kim, J. H., Chang, S. M., Hong, J. P., Bae, J. N., Cho, S. J., Hahm, B. J., . . . Cho, M. J. (2016). Lifetime prevalence, sociodemographic correlates, and diagnostic overlaps of bipolar spectrum disorder in the general population of South Korea. *Journal of Affective Disorders, 203*, 248-255. doi:10.1016/j.jad.2016.06.017

Kolada, J. L., Bland, R. C., & Newman, S. C. (1994). Epidemiology of psychiatric disorders in Edmonton. Obsessive-compulsive disorder. *Acta Psychiatrica Scandinavica Suppl, 376*, 24-35.

Lampe, L., Slade, T., Issakidis, C., & Andrews, G. (2003). Social phobia in the Australian National Survey of Mental Health and Well-Being (NSMHWB). *Psychological Medicine, 33*, 637-646.

Leray, E., Camara, A., Drapier, D., Riou, F., Bougeant, N., Pelissolo, A., . . . Millet, B. (2011). Prevalence, characteristics and comorbidities of anxiety disorders in France: results from the "Mental Health in General Population" survey (MHGP). *European Psychiatry, 26*, 339-345. doi:10.1016/j.eurpsy.2009.12.001

Lewinsohn, P. M., Hops, H., Roberts, R. E., Seeley, J. R., & Andrews, J. A. (1993). Adolescent psychopathology: I. Prevalence and incidence of depression and other DSM-III-R disorders in high school students. *Journal of Abnormal Psychology, 102*, 133-144.

Lewinsohn, P. M., Klein, D. N., & Seeley, J. R. (1995). Bipolar disorders in a community sample of older adolescents: prevalence, phenomenology, comorbidity, and course. *Journal of the American Academy of Child and Adolescent Psychiatry, 34*, 454-463. Retrieved from https://ac.els-cdn.com/S089085670963731X/1-s2.0-S089085670963731X-main.pdf?_tid=9d3967d0-ec0a-4a4e-8e8b-b88f5a06bcdb&acdnat=1540420949_0deb824a36925a6b12090df788fdc075

Lewinsohn, P. M., Zinbarg, R., Seeley, J. R., Lewinsohn, M., & Sack, W. H. (1997). Lifetime comorbidity among anxiety disorders and between anxiety disorders and other mental disorders in adolescents. *Journal of Anxiety Disorders, 11*, 377-394. Retrieved from https://ac.els-cdn.com/S0887618597000170/1-s2.0-S0887618597000170-main.pdf?_tid=84cf75c4-313d-410e-8883-6ab196cc0506&acdnat=1540420945_937c326c71619979a4e3ca28059c3da1

Lim, L., Ng, T. P., Chua, H. C., Chiam, P. C., Won, V., Lee, T., . . . Kua, E. H. (2005). Generalised anxiety disorder in Singapore: prevalence, co-morbidity and risk factors in a multi-ethnic population. *Social Psychiatry and Psychiatric Epidemiology, 40*, 972-979. doi:10.1007/s00127-005-0978-y

Magee, W. J., Eaton, W. W., Wittchen, H.-U., McGonagle, K. A., & Kessler, R. C. (1996). Agoraphobia, Simple Phobia, and Social Phobia in the National Comorbidity Survey. *Archives of General Psychiatry, 53*, 159-168. doi:10.1001/archpsyc.1996.01830020077009

Magklara, K., Bellos, S., Niakas, D., Stylianidis, S., Kolaitis, G., Mavreas, V., & Skapinakis, P. (2015). Depression in late adolescence: a cross-sectional study in senior high schools in Greece. *BMC Psychiatry, 15*, 199. doi:10.1186/s12888-015-0584-9

Martin-Merino, E., Ruigomez, A., Johansson, S., Wallander, M. A., & Garcia-Rodriguez, L. A. (2010). Study of a cohort of patients newly diagnosed with depression in general practice: prevalence, incidence, comorbidity, and treatment patterns. *Primary Care Companion to the Journal of Clinical Psychiatry, 12*, PCC.08m00764. doi:10.4088/PCC.08m00764blu

McCabe, L., Cairney, J., Veldhuizen, S., Herrmann, N., & Streiner, D. L. (2006). Prevalence and Correlates of Agoraphobia in Older Adults. *American Journal of Geriatric Psychiatry, 14*, 515-522. doi:https://doi.org/10.1097/01.JGP.0000203177.54242.14

McEvoy, P. M., Grove, R., & Slade, T. (2011). Epidemiology of anxiety disorders in the Australian general population: findings of the 2007 Australian National Survey of Mental Health and Wellbeing. *Australian and New Zealand Journal of Psychiatry, 45*, 957-967. doi:10.3109/00048674.2011.624083

Mergl, R., Seidscheck, I., Allgaier, A. K., Moller, H. J., Hegerl, U., & Henkel, V. (2007). Depressive, anxiety, and somatoform disorders in primary care: prevalence and recognition. *Depression and Anxiety, 24*, 185-195. doi:10.1002/da.20192

Merikangas, K. R., Angst, J., Eaton, W., Canino, G., Rubio-Stipec, M., Wacker, H., . . . Kupfer, D. J. (1996). Comorbidity and Boundaries of Affective Disorders with Anxiety Disorders and Substance Misuse: Results of an International Task Force. *British Journal of Psychiatry, 168*, 58-67. doi:10.1192/S0007125000298425

Merikangas, K. R., Jin, R., He, J. P., Kessler, R. C., Lee, S., Sampson, N. A., . . . Zarkov, Z. (2011). Prevalence and correlates of bipolar spectrum disorder in the world mental health survey initiative. *Archives of General Psychiatry, 68*, 241-251. doi:10.1001/archgenpsychiatry.2011.12

Merikangas, K. R., Zhang, H., Avenevoli, S., Acharyya, S., Neuenschwander, M., & Angst, J. (2003). Longitudinal trajectories of depression and anxiety in a prospective community study: the Zurich Cohort Study. *Archives of General Psychiatry, 60*, 993-1000. doi:10.1001/archpsyc.60.9.993

Mitchell, P., Slade, T., & Andrews, G. (2004). Twelve-month prevalence and disability of DSM-IV bipolar disorder in an Australian general population survey. *International Journal of Neuropharmacology, 34*, 777-785. doi:10.1017/S1461145710000635

Moffitt, T. E., Harrington, H., Caspi, A., Kim-Cohen, J., Goldberg, D., Gregory, A. M., & Poulton, R. (2007). Depression and generalized anxiety disorder: cumulative and sequential comorbidity in a birth cohort followed prospectively to age 32 years. *Archives of General Psychiatry, 64*, 651-660. doi:10.1001/archpsyc.64.6.651

Mohammadi, M. R., Ghanizadeh, A., Mohammadi, M., & Mesgarpour, B. (2006). Prevalence of social phobia and its comorbidity with psychiatric disorders in Iran. *Depression and Anxiety, 23*, 405-411. doi:10.1002/da.20129

Mohammadi, M. R., Ghanizadeh, A., & Moini, R. (2007). Lifetime comorbidity of obsessive-compulsive disorder with psychiatric disorders in a community sample. *Depression and Anxiety, 24*, 602-607. doi:10.1002/da.20259

Munyandamutsa, N., Mahoro Nkubamugisha, P., Gex-Fabry, M., & Eytan, A. (2012). Mental and physical health in Rwanda 14 years after the genocide. *Social Psychiatry and Psychiatric Epidemiology, 47*, 1753-1761. doi:10.1007/s00127-012-0494-9

Murphy, J. M., Horton, N. J., Laird, N. M., Monson, R. R., Sobol, A. M., & Leighton, A. H. (2004). Anxiety and depression: a 40-year perspective on relationships regarding prevalence, distribution, and comorbidity. *Acta Psychiatrica Scandinavica, 109*, 355-375. doi:10.1111/j.1600-0447.2003.00286.x

Newman, D. L., Moffitt, T. E., Caspi, A., Magdol, L., Silva, P. A., & Stanton, W. R. (1996). Psychiatric disorder in a birth cohort of young adults: Prevalence, comorbidity, clinical significance, and new case incidence from ages 11 to 21. *Journal of Consulting and Clinical Psychology, 64*, 552-562. doi:10.1037/0022-006X.64.3.552

Ohayon, M. M., & Schatzberg, A. F. (2010). Social phobia and depression: prevalence and comorbidity. *Journal of Psychosomatic Research, 68*, 235-243. doi:10.1016/j.jpsychores.2009.07.018

Ohayon, M. M., Shapiro, C. M., & Kennedy, S. H. (2000). Differentiating DSM-IV anxiety and depressive disorders in the general population: comorbidity and treatment consequences. *Canadian Journal of Psychiatry, 45*, 166-172. doi:10.1177/070674370004500207

Pakriev, S., Vasar, V., Aluoja, A., Saarma, M., & Shlik, J. (1998). Prevalence of mood disorders in the rural population of Udmurtia. *Acta Psychiatrica Scandinavica, 97*, 169-174.

Pietrzak, R. H., Goldstein, R. B., Southwick, S. M., & Grant, B. F. (2012). Psychiatric Comorbidity of Full and Partial Posttraumatic Stress Disorder Among Older Adults in the United States: Results From Wave 2 of the National Epidemiologic Survey on Alcohol and Related Conditions. *American Journal of Geriatric Psychiatry, 20*, 380-390. doi:https://doi.org/10.1097/JGP.0b013e31820d92e7

Pirkola, S. P., Isometsa, E., Suvisaari, J., Aro, H., Joukamaa, M., Poikolainen, K., . . . Lonnqvist, J. K. (2005). DSM-IV mood-, anxiety- and alcohol use disorders and their comorbidity in the Finnish general population-results from the Health 2000 Study. *Social Psychiatry and Psychiatric Epidemiology, 40*, 1-10. doi:10.1007/s00127-005-0848-7

Preville, M., Boyer, R., Grenier, S., Dube, M., Voyer, P., Punti, R., . . . Brassard, J. (2008). The epidemiology of psychiatric disorders in Quebec's older adult population. *Canadian Journal of Psychiatry, 53*, 822-832. doi:10.1177/070674370805301208

Prina, A. M., Ferri, C. P., Guerra, M., Brayne, C., & Prince, M. (2011). Co-occurrence of anxiety and depression amongst older adults in low- and middle-income countries: findings from the 10/66 study. *Psychological Medicine, 41*, 2047-2056. doi:10.1017/s0033291711000444

Rihmer, Z., Szadoczky, E., Furedi, J., Kiss, K., & Papp, Z. (2001). Anxiety disorders comorbidity in bipolar I, bipolar II and unipolar major depression: results from a population-based study in Hungary. *Journal of Affective Disorders, 67*, 175-179.

Ritchie, K., Norton, J., Mann, A., Carrière, I., & Ancelin, M.-L. (2013). Late-Onset Agoraphobia: General Population Incidence and Evidence for a Clinical Subtype. *American Journal of Psychiatry, 170*, 790-798. doi:10.1176/appi.ajp.2013.12091235

Rohde, P., Lewinsohn, P. M., & Seeley, J. R. (1991). Comorbidity of unipolar depression: II. Comorbidity with other mental disorders in adolescents and adults. *Journal of Abnormal Psychology, 100*, 214-222. doi:10.1037/0021-843X.100.2.214

Romano, E., Tremblay, R. E., Vitaro, F., Zoccolillo, M., & Pagani, L. (2005). Sex and informant effects on diagnostic comorbidity in an adolescent community sample. *Canadian Journal of Psychiatry, 50*, 479-489. doi:10.1177/070674370505000808

Roy-Byrne, P. P., Stang, P., Wittchen, H.-U., Ustun, B., Walters, E. E., & Kessler, R. C. (2000). Lifetime panic–depression comorbidity in the National Comorbidity Survey: Association with symptoms, impairment, course and help-seeking. *British Journal of Psychiatry, 176*, 229-235. doi:10.1192/bjp.176.3.229

Rueda-Jaimes, G. E., Camacho Lopez, P. A., & Navarro-Mancilla, A. A. (2008). [Prevalence of Obsessive Compulsive Disorder and its comorbidity with Major Depressive Disorder in adolescent students]. *Vertex, 19*, 5-9.

Ruscio, A. M., Brown, T. A., Chiu, W. T., Sareen, J., Stein, M. B., & Kessler, R. C. (2008). Social fears and social phobia in the USA: results from the National Comorbidity Survey Replication. *Psychological Medicine, 38*, 15-28. doi:10.1017/S0033291707001699

Ruscio, A. M., Stein, D. J., Chiu, W. T., & Kessler, R. C. (2010). The epidemiology of obsessive-compulsive disorder in the National Comorbidity Survey Replication. *Molecular Psychiatry, 15*, 53-63. doi:10.1038/mp.2008.94

Sahoo, S., & Khess, C. R. (2010). Prevalence of depression, anxiety, and stress among young male adults in India: a dimensional and categorical diagnoses-based study. *Journal of Nervous and Mental Disease, 198*, 901-904. doi:10.1097/NMD.0b013e3181fe75dc

Sartorius, N., Ustun, T. B., Lecrubier, Y., & Wittchen, H. U. (1996). Depression comorbid with anxiety: results from the WHO study on psychological disorders in primary health care. *British Journal of Psychiatry. Supplement*, 38-43.

Schaffer, A., Cairney, J., Cheung, A., Veldhuizen, S., & Levitt, A. (2006). Community survey of bipolar disorder in Canada: lifetime prevalence and illness characteristics. *Canadian Journal of Psychiatry, 51*, 9-16. doi:10.1177/070674370605100104

Schaub, R. T., & Linden, M. (2000). Anxiety and anxiety disorders in the old and very old—Results from the Berlin aging study (BASE). *Comprehensive Psychiatry, 41*, 48-54. doi:https://doi.org/10.1016/S0010-440X(00)80008-5

Schneier, F. R., Johnson, J., Hornig, C. D., Liebowitz, M. R., & Weissman, M. M. (1992). Social Phobia: Comorbidity and Morbidity in an Epidemiologic Sample. *Archives of General Psychiatry, 49*, 282-288. doi:10.1001/archpsyc.1992.01820040034004

Schoevers, R. A., Beekman, A. T., Deeg, D. J., Jonker, C., & van Tilburg, W. (2003). Comorbidity and risk-patterns of depression, generalised anxiety disorder and mixed anxiety-depression in later life: results from the AMSTEL study. *International Journal of Geriatric Psychiatry, 18*, 994-1001. doi:10.1002/gps.1001

Schrier, A. C., de Wit, M. A., Coupe, V. M., Fassaert, T., Verhoeff, A. P., Kupka, R. W., . . . Beekman, A. T. (2012). Comorbidity of anxiety and depressive disorders: a comparative population study in Western and non-Western inhabitants in the Netherlands. *International Journal of Social Psychiatry, 58*, 186-194. doi:10.1177/0020764010390433

Scott, K. M., McGee, M. A., Oakley Browne, M. A., & Wells, J. E. (2006). Mental disorder comorbidity in Te Rau Hinengaro: the New Zealand Mental Health Survey. *Australian and New Zealand Journal of Psychiatry, 40*, 875-881. doi:10.1080/j.1440-1614.2006.01906.x

Serrano-Blanco, A., Palao, D. J., Luciano, J. V., Pinto-Meza, A., Lujan, L., Fernandez, A., . . . Haro, J. M. (2010). Prevalence of mental disorders in primary care: results from the diagnosis and treatment of mental disorders in primary care study (DASMAP). *Social Psychiatry and Psychiatric Epidemiology, 45*, 201-210. doi:10.1007/s00127-009-0056-y

Sicras-Mainar, A., Blanca-Tamayo, M., Navarro-Artieda, R., Pizarro-Paixa, I., & Gomez-Lus Centelles, S. (2008). [Influence of morbidity and the use of health resources in patients who require care for generalised anxiety disorder in the primary health care setting]. *Atencion Primarie, 40*, 603-610.

Spaner, D., Bland, R. C., & Newman, S. C. (1994). Epidemiology of psychiatric disorders in Edmonton. Major depressive disorder. *Acta Psychiatrica Scandinavica Suppl, 376*, 7-15.

Stein, M. B., & Heimberg, R. G. (2004). Well-being and life satisfaction in generalized anxiety disorder: comparison to major depressive disorder in a community sample. *Journal of Affective Disorders, 79*, 161-166. doi:10.1016/s0165-0327(02)00457-3

Stylianidis, S., Pantelidou, S., Chondros, P., Roelandt, J. L., & Barbato, A. (2014). Prevalence of mental disorders in a Greek island. *Psychiatriki, 25*, 19-26.

Subramaniam, M., Abdin, E., Vaingankar, J. A., & Chong, S. A. (2013). Prevalence, correlates, comorbidity and severity of bipolar disorder: Results from the Singapore Mental Health Study. *Journal of Affective Disorders, 146*, 189-196. doi:https://doi.org/10.1016/j.jad.2012.09.002

Szadoczky, E., Rihmer, Z., Papp, Z. S., Vitrai, J., & Furedi, J. (2002). Gender differences in major depressive disorder in a Hungarian community survey. *International Journal of Psychiatry in Clinical Practice, 6*, 31-37. doi:10.1080/136515002753489399

Thompson, A. H., Bland, R. C., & Orn, H. T. (1989). Relationship and chronology of depression, agoraphobia, and panic disorder in the general population. *Journal of Nervous and Mental Disease, 177*, 456-463. doi:10.1097/00005053-198908000-00002

Trumpf, J., Margraf, J., Vriends, N., Meyer, A. H., & Becker, E. S. (2010). Specific phobia predicts psychopathology in young women. *Social Psychiatry and Psychiatric Epidemiology, 45*, 1161-1166. doi:10.1007/s00127-009-0159-5

Tsuchiya, M., Kawakami, N., Ono, Y., Nakane, Y., Nakamura, Y., Tachimori, H., . . . Kessler, R. C. (2009). Lifetime comorbidities between phobic disorders and major depression in Japan: results from the World Mental Health Japan 2002-2004 Survey. *Depression and Anxiety, 26*, 949-955. doi:10.1002/da.20508

Vaiva, G., Jehel, L., Cottencin, O., Ducrocq, F., Duchet, C., Omnes, C., . . . Roelandt, J. L. (2008). [Prevalence of trauma-related disorders in the French WHO study: Sante mentale en population generale (SMPG)]. *Encephale, 34*, 577-583. doi:10.1016/j.encep.2007.11.006

Van Ameringen, M., Mancini, C., Patterson, B., & Boyle, M. H. (2008). Post-traumatic stress disorder in Canada. *CNS Neuroscience & Therapeutics, 14*, 171-181. doi:10.1111/j.1755-5949.2008.00049.x

van Balkom, A. J., Beekman, A. T., de Beurs, E., Deeg, D. J., van Dyck, R., & van Tilburg, W. (2000). Comorbidity of the anxiety disorders in a community-based older population in The Netherlands. *Acta Psychiatrica Scandinavica, 101*, 37-45.

van Loo, H. M., Schoevers, R. A., Kendler, K. S., de Jonge, P., & Romeijn, J. W. (2016). Psychiatric comorbidity does not only depend on diagnostic thresholds: an illustration with major depressive disorder and generalized anxiety disorder. *Depression and Anxiety, 33*, 143-152. doi:10.1002/da.22453

Weissman, M. M., Bland, R. C., Canino, G. J., Faravelli, C., Greenwald, S., Hwu, H. G., . . . Yeh, E. K. (1997). The cross-national epidemiology of panic disorder. *Archives of General Psychiatry, 54*, 305-309.

Weissman, M. M., Bland, R. C., Canino, G. J., Faravelli, C., Greenwald, S., Hwu, H. G., . . . Yeh, E. K. (1996). Cross-national epidemiology of major depression and bipolar disorder. *JAMA, 276*, 293-299.

Weissman, M. M., Bland, R. C., Canino, G. J., Greenwald, S., Hwu, H. G., Lee, C. K., . . . et al. (1994). The cross national epidemiology of obsessive compulsive disorder. The Cross National Collaborative Group. *Journal of Clinical Epidemiology, 55 Suppl*, 5-10.

Wichstrom, L., Berg-Nielsen, T. S., Angold, A., Egger, H. L., Solheim, E., & Sveen, T. H. (2012). Prevalence of psychiatric disorders in preschoolers. *Journal of Child Psychology and Psychiatry and Allied Disciplines, 53*, 695-705. doi:10.1111/j.1469-7610.2011.02514.x

Wittchen, H.-U., Carter, R. M., Pfister, H., Montgomery, S. A., & Kessler, R. C. (2000). Disabilities and quality of life in pure and comorbid generalized anxiety disorder and major depression in a national survey. *International Clinical Psychopharmacology, 15*, 319-328. doi:10.1097/00004850-200015060-00002

Wittchen, H.-U., Zhao, S., Kessler, R. C., & Eaton, W. W. (1994). DSM-III-R Generalized Anxiety Disorder in the National Comorbidity Survey. *Archives of General Psychiatry, 51*, 355-364. doi:10.1001/archpsyc.1994.03950050015002

Wittchen, H. U., Essau, C. A., von Zerssen, D., Krieg, J. C., & Zaudig, M. (1992). Lifetime and six-month prevalence of mental disorders in the Munich Follow-Up Study. *European Archives of Psychiatry and Clinical Neuroscience, 241*, 247-258.

Wittchen, H. U., Stein, M. B., & Kessler, R. C. (1999). Social fears and social phobia in a community sample of adolescents and young adults: prevalence, risk factors and co-morbidity. *Psychological Medicine, 29*, 309-323. doi:undefined

Zhang, X., Norton, J., Carriere, I., Ritchie, K., Chaudieu, I., & Ancelin, M. L. (2015). Risk factors for late-onset generalized anxiety disorder: results from a 12-year prospective cohort (the ESPRIT study). *Translational Psychiatry, 5*, e536. doi:10.1038/tp.2015.31

Zutshi, A., Reddy, Y. C., Thennarasu, K., & Chandrashekhar, C. R. (2006). Comorbidity of anxiety disorders in patients with remitted bipolar disorder. *European Archives of Psychiatry and Clinical Neuroscience, 256*, 428-436. doi:10.1007/s00406-006-0658-2

eReference 2. eTable 1. Studies presenting temporally-ordered estimates (36 studies)

Acarturk, C., Smit, F., de Graaf, R., van Straten, A., ten Have, M., & Cuijpers, P. (2009). Incidence of social phobia and identification of its risk indicators: a model for prevention. *Acta Psychiatrica Scandinavica, 119*, 62-70. doi:10.1111/j.1600-0447.2008.01275.x

Beesdo, K., Bittner, A., Pine, D. S., Stein, M. B., Hofler, M., Lieb, R., & Wittchen, H. U. (2007). Incidence of social anxiety disorder and the consistent risk for secondary depression in the first three decades of life. *Archives of General Psychiatry, 64*, 903-912. doi:10.1001/archpsyc.64.8.903

Beesdo, K., Pine, D. S., Lieb, R., & Wittchen, H.-U. (2010). Incidence and Risk Patterns of Anxiety and Depressive Disorders and Categorization of Generalized Anxiety Disorder. . *Archives of General Psychiatry, 67*, 47-57. doi:10.1001/archgenpsychiatry.2009.177

Bittner, A., Egger, H. L., Erkanli, A., Jane Costello, E., Foley, D. L., & Angold, A. (2007). What do childhood anxiety disorders predict? *Journal of Child Psychology and Psychiatry and Allied Disciplines, 48*, 1174-1183. doi:10.1111/j.1469-7610.2007.01812.x

Bittner, A., Goodwin, R. D., Wittchen, H. U., Beesdo, K., Hofler, M., & Lieb, R. (2004). What characteristics of primary anxiety disorders predict subsequent major depressive disorder? *Journal of Clinical Epidemiology, 65*, 618-626, quiz 730. Retrieved from http://www.psychiatrist.com/jcp/article/pages/2004/v65n05/v65n0505.aspx

Bromet, E., Sonnega, A., & Kessler, R. C. (1998). Risk Factors for DSM-III-R Posttraumatic Stress Disorder: Findings from the National Comorbidity Survey. *American Journal of Epidemiology, 147*, 353-361. doi:10.1093/oxfordjournals.aje.a009457

Cederlof, M., Lichtenstein, P., Larsson, H., Boman, M., Ruck, C., Landen, M., & Mataix-Cols, D. (2015). Obsessive-Compulsive Disorder, Psychosis, and Bipolarity: A Longitudinal Cohort and Multigenerational Family Study. *Schizophrenia Bulletin, 41*, 1076-1083. doi:10.1093/schbul/sbu169

Chang, J. C., Yen, A. M., Chen, H. H., Chen, S. L., Chiu, S. Y., Fann, J. C., & Lee, C. S. (2017). Comorbid diseases as risk factors for incident posttraumatic stress disorder (PTSD) in a large community cohort (KCIS no.PSY4). *Science reporter, 7*, 41276. doi:10.1038/srep41276

Chou, K.-L., Mackenzie, C. S., Liang, K., & Sareen, J. (2011). Three-year incidence and predictors of first-onset of DSM-IV mood, anxiety, and substance use disorders in older adults: Results from wave 2 of the National Epidemiologic Survey on Alcohol and Related Conditions. *Journal of Clinical Epidemiology, 72*, 144-155. doi:10.4088/JCP.09m05618gry

Costello, E. J., Mustillo, S., Erkanli, A., Keeler, G., & Angold, A. (2003). Prevalence and Development of Psychiatric Disorders in Childhood and Adolescence. *Archives of General Psychiatry, 60*, 837-844. doi:10.1001/archpsyc.60.8.837

De Graaf, R., Bijl, R. V., Ten Have, M., Beekman, A. T. F., & Vollebergh, W. A. M. (2004). Rapid onset of comorbidity of common mental disorders: findings from the Netherlands Mental Health Survey and Incidence Study (NEMESIS). *Acta Psychiatrica Scandinavica, 109*, 55-63. doi:10.1046/j.0001-690X.2003.00222.x

Goodwin, R. D. (2002). Anxiety disorders and the onset of depression among adults in the community. *Psychological Medicine, 32*, 1121-1124.

Goodwin, R. D., Lieb, R., Hoefler, M., Pfister, H., Bittner, A., Beesdo, K., & Wittchen, H. U. (2004). Panic attack as a risk factor for severe psychopathology. *American Journal of Psychiatry, 161*, 2207-2214. doi:10.1176/appi.ajp.161.12.2207

Grant, B. F., Goldstein, R. B., Chou, S. P., Huang, B., Stinson, F. S., Dawson, D. A., . . . Compton, W. M. (2009). Sociodemographic and psychopathologic predictors of first incidence of DSM-IV substance use, mood and anxiety disorders: results from the Wave 2 National Epidemiologic Survey on Alcohol and Related Conditions. *Molecular Psychiatry, 14*, 1051-1066. doi:10.1038/mp.2008.41

Jaffee, S. R., Moffitt, T. E., Caspi, A., Fombonne, E., Poulton, R., & Martin, J. (2002). Differences in Early Childhood Risk Factors for Juvenile-Onset and Adult-Onset Depression. *Archives of General Psychiatry, 59*, 215-222. doi:10.1001/archpsyc.59.3.215

Johnson, J. G., Cohen, P., & Brook, J. S. (2000). Associations between bipolar disorder and other psychiatric disorders during adolescence and early adulthood: a community-based longitudinal investigation. *American Journal of Psychiatry, 157*, 1679-1681. doi:10.1176/appi.ajp.157.10.1679

Kang, H. J., Bae, K. Y., Kim, S. W., Shin, I. S., Yoon, J. S., & Kim, J. M. (2016). Anxiety symptoms in Korean elderly individuals: a two-year longitudinal community study. *International Psychogeriatrics, 28*, 423-433. doi:10.1017/s1041610215001301

Kessler, R. C., Andrade, L. H., Bijl, R. V., Offord, D. R., Demler, O. V., & Stein, D. J. (2002). The effects of co-morbidity on the onset and persistence of generalized anxiety disorder in the ICPE surveys. *Psychological Medicine, 32*, 1213-1225. doi:10.1017/s0033291702006104

Kessler, R. C., DuPont, R. L., Berglund, P., & Wittchen, H. U. (1999). Impairment in pure and comorbid generalized anxiety disorder and major depression at 12 months in two national surveys. *American Journal of Psychiatry, 156*, 1915-1923. doi:10.1176/ajp.156.12.1915

Kessler, R. C., Gruber, M., Hettema, J. M., Hwang, I., Sampson, N., & Yonkers, K. A. (2008). Co-morbid major depression and generalized anxiety disorders in the National Comorbidity Survey follow-up. *Psychological Medicine, 38*, 365-374. doi:10.1017/S0033291707002012

Kessler, R. C., Nelson, C. B., McGonagle, K. A., Liu, J., Swartz, M., & Blazer, D. G. (1996). Comorbidity of DSM-III-R major depressive disorder in the general population: results from the US National Comorbidity Survey. *British Journal of Psychiatry. Supplement, 30*, 17-30.

Kessler, R. C., Stang, P. E., Wittchen, H. U., Ustun, T. B., Roy-Burne, P. P., & Walters, E. E. (1998). Lifetime panic-depression comorbidity in the National Comorbidity Survey. *Archives of General Psychiatry, 55*, 801-808. Retrieved from https://jamanetwork.com/journals/jamapsychiatry/articlepdf/204221/yoa7184.pdf

Keyl, P. M., & Eaton, W. W. (1990). Risk factors for the onset of panic disorder and other panic attacks in a prospective, population-based study. *American Journal of Epidemiology, 131*, 301-311. doi:10.1093/oxfordjournals.aje.a115499

Kim-Cohen, J., Caspi, A., Moffitt, T. E., Harrington, H., Milne, B. J., & Poulton, R. (2003). Prior Juvenile Diagnoses in Adults With Mental Disorder: Developmental Follow-Back of a Prospective-Longitudinal Cohort. *Archives of General Psychiatry, 60*, 709-717. doi:10.1001/archpsyc.60.7.709

Lieb, R., Miche, M., Gloster, A. T., Beesdo-Baum, K., Meyer, A. H., & Wittchen, H. U. (2016). Impact of specific phobia on the risk of onset of mental disorders: a 10-year prospective-longitudinal community study of adolescents and young adults. *Depression and Anxiety, 33*, 667-675. doi:10.1002/da.22487

Mathew, A. R., Pettit, J. W., Lewinsohn, P. M., Seeley, J. R., & Roberts, R. E. (2011). Co-morbidity between major depressive disorder and anxiety disorders: shared etiology or direct causation? *Psychological Medicine, 41*, 2023-2034. doi:10.1017/s0033291711000407

Meier, S. M., Petersen, L., Mattheisen, M., Mors, O., Mortensen, P. B., & Laursen, T. M. (2015). Secondary depression in severe anxiety disorders: a population-based cohort study in Denmark. *Lancet Psychiatry, 2*, 515-523. doi:10.1016/s2215-0366(15)00092-9

Olfson, M., Mojtabai, R., Merikangas, K. R., Compton, W. M., Wang, S., Grant, B. F., & Blanco, C. (2017). Reexamining associations between mania, depression, anxiety and substance use disorders: results from a prospective national cohort. *Molecular Psychiatry, 22*, 235-241. doi:10.1038/mp.2016.64

Pine, D. S., Cohen, P., Gurley, D., Brook, J., & Ma, Y. (1998). The Risk for Early-Adulthood Anxiety and Depressive Disorders in Adolescents With Anxiety and Depressive Disorders. *Archives of General Psychiatry, 55*, 56-64. doi:10.1001/archpsyc.55.1.56

Ritchie, K., Norton, J., Mann, A., Carrière, I., & Ancelin, M.-L. (2013). Late-Onset Agoraphobia: General Population Incidence and Evidence for a Clinical Subtype. *American Journal of Psychiatry, 170*, 790-798. doi:10.1176/appi.ajp.2013.12091235

Ruscio, A. M., Stein, D. J., Chiu, W. T., & Kessler, R. C. (2010). The epidemiology of obsessive-compulsive disorder in the National Comorbidity Survey Replication. *Molecular Psychiatry, 15*, 53-63. doi:10.1038/mp.2008.94

Schoevers, R. A., Beekman, A. T. F., Deeg, D. J. H., Geerlings, M. I., Jonker, C., & Van Tilburg, W. (2000). Risk factors for depression in later life; results of a prospective community based study (AMSTEL). *Journal of Affective Disorders, 59*, 127-137. doi:https://doi.org/10.1016/S0165-0327(99)00124-X

Stein, M. B., Fuetsch, M., Müller, N., Höfler, M., Lieb, R., & Wittchen, H.-U. (2001). Social Anxiety Disorder and the Risk of Depression: A Prospective Community Study of Adolescents and Young Adults. *Archives of General Psychiatry, 58*, 251-256. doi:10.1001/archpsyc.58.3.251

Tsuchiya, M., Kawakami, N., Ono, Y., Nakane, Y., Nakamura, Y., Tachimori, H., . . . Kessler, R. C. (2009). Lifetime comorbidities between phobic disorders and major depression in Japan: results from the World Mental Health Japan 2002-2004 Survey. *Depression and Anxiety, 26*, 949-955. doi:10.1002/da.20508

Woodward, L. J., & Fergusson, D. M. (2001). Life Course Outcomes of Young People With Anxiety Disorders in Adolescence. *Journal of the American Academy of Child and Adolescent Psychiatry, 40*, 1086-1093. doi:https://doi.org/10.1097/00004583-200109000-00018

Zhang, X., Norton, J., Carriere, I., Ritchie, K., Chaudieu, I., & Ancelin, M. L. (2015). Risk factors for late-onset generalized anxiety disorder: results from a 12-year prospective cohort (the ESPRIT study). *Translational Psychiatry, 5*, e536. doi:10.1038/tp.2015.31

eReference 3. Mood and anxiety: lifetime comorbidity (9 studies)

Beesdo, K., Bittner, A., Pine, D. S., Stein, M. B., Hofler, M., Lieb, R., & Wittchen, H. U. (2007). Incidence of social anxiety disorder and the consistent risk for secondary depression in the first three decades of life. *Archives of General Psychiatry, 64*, 903-912. doi:10.1001/archpsyc.64.8.903

Bromet, E. J., Gluzman, S. F., Paniotto, V. I., Webb, C. P. M., Tintle, N. L., Zakhozha, V., . . . Schwartz, J. E. (2005). Epidemiology of psychiatric and alcohol disorders in Ukraine. *Social Psychiatry and Psychiatric Epidemiology, 40*, 681-690. doi:10.1007/s00127-005-0927-9

Essau, C. A. (2003). Comorbidity of anxiety disorders in adolescents. *Depression and Anxiety, 18*, 1-6. doi:10.1002/da.10107

He, Y. L., Ma, H., Zhang, L., Liu, Z. N., Jia, F. J., & Zhang, M. Y. (2009). [A cross-sectional survey of the prevalence of depressive-anxiety disorders among general hospital outpatients in five cities in China]. *Chinese Journal of Internal Medicine, 48*, 748-751.

Kawakami, N., Shimizu, H., Haratani, T., Iwata, N., & Kitamura, T. (2004). Lifetime and 6-month prevalence of DSM-III-R psychiatric disorders in an urban community in Japan. *Psychiatry Research, 121*, 293-301. doi:https://doi.org/10.1016/S0165-1781(03)00239-7

Magee, W. J., Eaton, W. W., Wittchen, H.-U., McGonagle, K. A., & Kessler, R. C. (1996). Agoraphobia, Simple Phobia, and Social Phobia in the National Comorbidity Survey. *Archives of General Psychiatry, 53*, 159-168. doi:10.1001/archpsyc.1996.01830020077009

Pakriev, S., Vasar, V., Aluoja, A., Saarma, M., & Shlik, J. (1998). Prevalence of mood disorders in the rural population of Udmurtia. *Acta Psychiatrica Scandinavica, 97*, 169-174.

Trumpf, J., Margraf, J., Vriends, N., Meyer, A. H., & Becker, E. S. (2010). Specific phobia predicts psychopathology in young women. *Social Psychiatry and Psychiatric Epidemiology, 45*, 1161-1166. doi:10.1007/s00127-009-0159-5

Wittchen, H. U., Essau, C. A., von Zerssen, D., Krieg, J. C., & Zaudig, M. (1992). Lifetime and six-month prevalence of mental disorders in the Munich Follow-Up Study. *European Archives of Psychiatry and Clinical Neuroscience, 241*, 247-258.

eReference 4. MOOD and anxiety disorder: period prevalence comorbidity (20 studies)

Carter, R. M., Wittchen, H. U., Pfister, H., & Kessler, R. C. (2001). One-year prevalence of subthreshold and threshold DSM-IV generalized anxiety disorder in a nationally representative sample. *Depression and Anxiety, 13*, 78-88.

Copeland, W. E., Shanahan, L., Erkanli, A., Costello, E. J., & Angold, A. (2013). Indirect comorbidity in childhood and adolescence. *Frontiers in Psychiatry, 4*, 144. doi:10.3389/fpsyt.2013.00144

Costello, E. J., Mustillo, S., Erkanli, A., Keeler, G., & Angold, A. (2003). Prevalence and Development of Psychiatric Disorders in Childhood and Adolescence. *Archives of General Psychiatry, 60*, 837-844. doi:10.1001/archpsyc.60.8.837

de Graaf, R., Bijl, R. V., Smit, F., Vollebergh, W. A. M., & Spijker, J. (2002). Risk Factors for 12-Month Comorbidity of Mood, Anxiety, and Substance Use Disorders: Findings From the Netherlands Mental Health Survey and Incidence Study. *American Journal of Psychiatry, 159*, 620-629. doi:10.1176/appi.ajp.159.4.620

Fehm, L., Beesdo, K., Jacobi, F., & Fiedler, A. (2008). Social anxiety disorder above and below the diagnostic threshold: prevalence, comorbidity and impairment in the general population. *Social Psychiatry and Psychiatric Epidemiology, 43*, 257-265. doi:10.1007/s00127-007-0299-4

Fleitlich-Bilyk, B., & Goodman, R. (2004). Prevalence of Child and Adolescent Psychiatric Disorders in Southeast Brazil. *Journal of the American Academy of Child and Adolescent Psychiatry, 43*, 727-734. doi:https://doi.org/10.1097/01.chi.0000120021.14101.ca

Fung, A. W., Chan, W. C., Wong, C. S., Chen, E. Y., Ng, R. M., Lee, E. H., . . . Lam, L. C. (2017). Prevalence of anxiety disorders in community dwelling older adults in Hong Kong. *International Psychogeriatrics, 29*, 259-267. doi:10.1017/s1041610216001617

Grant, B. F., Hasin, D. S., Blanco, C., Stinson, F. S., Chou, S. P., Goldstein, R. B., . . . Huang, B. (2005). The epidemiology of social anxiety disorder in the United States: results from the National Epidemiologic Survey on Alcohol and Related Conditions. *Journal of Clinical Epidemiology, 66*, 1351-1361. Retrieved from http://www.psychiatrist.com/jcp/article/pages/2005/v66n11/v66n1102.aspx

Grant, B. F., Hasin, D. S., Stinson, F. S., Dawson, D. A., June Ruan, W., Goldstein, R. B., . . . Huang, B. (2005). Prevalence, correlates, co-morbidity, and comparative disability of DSM-IV generalized anxiety disorder in the USA: results from the National Epidemiologic Survey on Alcohol and Related Conditions. *Psychological Medicine, 35*, 1747-1759. doi:10.1017/s0033291705006069

Grant, B. F., Stinson, F. S., Hasin, D. S., Dawson, D. A., Chou, S. P., Ruan, W. J., & Huang, B. (2005). Prevalence, correlates, and comorbidity of bipolar I disorder and axis I and II disorders: results from the National Epidemiologic Survey on Alcohol and Related Conditions. *Journal of Clinical Epidemiology, 66*, 1205-1215. Retrieved from http://www.psychiatrist.com/jcp/article/pages/2005/v66n10/v66n1001.aspx

He, Y. L., Ma, H., Zhang, L., Liu, Z. N., Jia, F. J., & Zhang, M. Y. (2009). [A cross-sectional survey of the prevalence of depressive-anxiety disorders among general hospital outpatients in five cities in China]. *Chinese Journal of Internal Medicine, 48*, 748-751.

Hecht, H., von Zerssen, D., & Wittchen, H. U. (1990). Anxiety and depression in a community sample: the influence of comorbidity on social functioning. *Journal of Affective Disorders, 18*, 137-144. Retrieved from https://ac.els-cdn.com/016503279090070O/1-s2.0-016503279090070O-

main.pdf?_tid=c9d38f12-1e00-4420-b2b8-8c170b8b6605&acdnat=1540420771_2843747c9059354354132ad03a408de7

Hek, K., Tiemeier, H., Newson, R. S., Luijendijk, H. J., Hofman, A., & Mulder, C. L. (2011). Anxiety disorders and comorbid depression in community dwelling older adults. *International Journal of Methods in Psychiatric Research, 20*, 157-168. doi:10.1002/mpr.344

Hunt, C., Issakidis, C., & Andrews, G. (2002). DSM-IV generalized anxiety disorder in the Australian National Survey of Mental Health and Well-Being. *Psychological Medicine, 32*, 649-659. doi:10.1017/S0033291702005512

McEvoy, P. M., Grove, R., & Slade, T. (2011). Epidemiology of anxiety disorders in the Australian general population: findings of the 2007 Australian National Survey of Mental Health and Wellbeing. *Australian and New Zealand Journal of Psychiatry, 45*, 957-967. doi:10.3109/00048674.2011.624083

Mergl, R., Seidscheck, I., Allgaier, A. K., Moller, H. J., Hegerl, U., & Henkel, V. (2007). Depressive, anxiety, and somatoform disorders in primary care: prevalence and recognition. *Depression and Anxiety, 24*, 185-195. doi:10.1002/da.20192

Newman, D. L., Moffitt, T. E., Caspi, A., Magdol, L., Silva, P. A., & Stanton, W. R. (1996). Psychiatric disorder in a birth cohort of young adults: Prevalence, comorbidity, clinical significance, and new case incidence from ages 11 to 21. *Journal of Consulting and Clinical Psychology, 64*, 552-562. doi:10.1037/0022-006X.64.3.552

Ohayon, M. M., Shapiro, C. M., & Kennedy, S. H. (2000). Differentiating DSM-IV anxiety and depressive disorders in the general population: comorbidity and treatment consequences. *Canadian Journal of Psychiatry, 45*, 166-172. doi:10.1177/070674370004500207

Pirkola, S. P., Isometsa, E., Suvisaari, J., Aro, H., Joukamaa, M., Poikolainen, K., . . . Lonnqvist, J. K. (2005). DSM-IV mood-, anxiety- and alcohol use disorders and their comorbidity in the Finnish general population-results from the Health 2000 Study. *Social Psychiatry and Psychiatric Epidemiology, 40*, 1-10. doi:10.1007/s00127-005-0848-7

Wichstrom, L., Berg-Nielsen, T. S., Angold, A., Egger, H. L., Solheim, E., & Sveen, T. H. (2012). Prevalence of psychiatric disorders in preschoolers. *Journal of Child Psychology and Psychiatry and Allied Disciplines, 53*, 695-705. doi:10.1111/j.1469-7610.2011.02514.x

eReference 5. DEP and anxiety disorder: lifetime comorbidity (48 studies)

Arillo Crespo, A., Aguinaga Ontoso, I., & Guillen Grima, F. (1998). [Prevalence of mental diseases in women of an urban area]. *Atencion Primarie, 21*, 265-269.

Bromet, E. J., Gluzman, S. F., Paniotto, V. I., Webb, C. P. M., Tintle, N. L., Zakhozha, V., . . . Schwartz, J. E. (2005). Epidemiology of psychiatric and alcohol disorders in Ukraine. *Social Psychiatry and Psychiatric Epidemiology, 40*, 681-690. doi:10.1007/s00127-005-0927-9

Bruce, S. E., Weisberg, R. B., Dolan, R. T., Machan, J. T., Kessler, R. C., Manchester, G., . . . Keller, M. B. (2001). Trauma and Posttraumatic Stress Disorder in Primary Care Patients. *Primary Care Companion to the Journal of Clinical Psychiatry, 3*, 211-217. Retrieved from https://www.ncbi.nlm.nih.gov/pubmed/15014575
https://www.ncbi.nlm.nih.gov/pmc/PMC181217/

Chartier, M. J., Walker, J. R., & Stein, M. B. (2003). Considering comorbidity in social phobia. *Social Psychiatry and Psychiatric Epidemiology, 38*, 728-734. doi:10.1007/s00127-003-0720-6

Chavira, D. A., Stein, M. B., Bailey, K., & Stein, M. T. (2004). Comorbidity of generalized social anxiety disorder and depression in a pediatric primary care sample. *Journal of Affective Disorders, 80*, 163-171. doi:10.1016/s0165-0327(03)00103-4

Chen, Y. W., & Dilsaver, S. C. (1995). Comorbidity of panic disorder in bipolar illness: evidence from the Epidemiologic Catchment Area Survey. *American Journal of Psychiatry, 152*, 280-282. doi:10.1176/ajp.152.2.280

Choy, Y., Fyer, A. J., & Goodwin, R. D. (2007). Specific phobia and comorbid depression: a closer look at the National Comorbidity Survey data. *Comprehensive Psychiatry, 48*, 132-136. Retrieved from http://search.ebscohost.com/login.aspx?direct=true&db=cin20&AN=106200452&site=ehost-live

https://ac.els-cdn.com/S0010440X06001349/1-s2.0-S0010440X06001349-main.pdf?_tid=1fd90ceb-466c-4530-bf21-01a1131da900&acdnat=1540420573_3254cd4850537cc2473196cfbdad5e7e

Coyne, J. C., Fechner-Bates, S., & Schwenk, T. L. (1994). Prevalence, nature, and comorbidity of depressive disorders in primary care. *General Hospital Psychiatry, 16*, 267-276. Retrieved from https://deepblue.lib.umich.edu/bitstream/handle/2027.42/31474/0000396.pdf?sequence=1

Cyranowski, J. M., Schott, L. L., Kravitz, H. M., Brown, C., Thurston, R. C., Joffe, H., . . . Bromberger, J. T. (2012). Psychosocial features associated with lifetime comorbidity of major depression and anxiety disorders among a community sample of mid-life women: the SWAN mental health study. *Depression and Anxiety, 29*, 1050-1057. doi:10.1002/da.21990

Davidson, J. R. T., Hughes, D., Blazer, D. G., & George, L. K. (1991). Post-traumatic stress disorder in the community: an epidemiological study. *Psychological Medicine, 21*, 713-721. doi:10.1017/S0033291700022352

de Graaf, R., Bijl, R. V., Spijker, J., Beekman, A. T., & Vollebergh, W. A. (2003). Temporal sequencing of lifetime mood disorders in relation to comorbid anxiety and substance use disorders--findings from the Netherlands Mental Health Survey and Incidence Study. *Social Psychiatry and Psychiatric Epidemiology, 38*, 1-11. doi:10.1007/s00127-003-0597-4

Depla, M. F. I. A., ten Have, M. L., van Balkom, A. J. L. M., & de Graaf, R. (2008). Specific fears and phobias in the general population: Results from the Netherlands Mental Health Survey and Incidence Study (NEMESIS). *Social Psychiatry and Psychiatric Epidemiology, 43*, 200-208. doi:10.1007/s00127-007-0291-z

Essau, C. A. (2003). Comorbidity of anxiety disorders in adolescents. *Depression and Anxiety, 18*, 1-6. doi:10.1002/da.10107

Faravelli, C., Abrardi, L., Bartolozzi, D., Cecchi, C., Cosci, F., D'Adamo, D., . . . Rosi, S. (2004). The Sesto Fiorentino Study: Background, Methods and Preliminary Results. *Psychotherapy and Psychosomatics, 73*, 216-225. doi:10.1159/000077740

Goodwin, R. D., Lieb, R., Hoefler, M., Pfister, H., Bittner, A., Beesdo, K., & Wittchen, H. U. (2004). Panic attack as a risk factor for severe psychopathology. *American Journal of Psychiatry, 161*, 2207-2214. doi:10.1176/appi.ajp.161.12.2207

Grabe, H. J., Meyer, C., Hapke, U., Rumpf, H. J., Freyberger, H. J., Dilling, H., & John, U. (2001). Lifetime-comorbidity of obsessive-compulsive disorder and subclinical obsessive-compulsive disorder in Northern Germany. *European Archives of Psychiatry and Clinical Neuroscience, 251*, 130-135.

Gratzer, D., Levitan, R. D., Sheldon, T., Toneatto, T., Rector, N. A., & Goering, P. (2004). Lifetime rates of alcoholism in adults with anxiety, depression, or co-morbid depression/anxiety: a community survey of Ontario. *Journal of Affective Disorders, 79*, 209-215. doi:https://doi.org/10.1016/S0165-0327(02)00355-5

Hasin, D. S., Goodwin, R. D., Stinson, F. S., & Grant, B. F. (2005). Epidemiology of major depressive disorder: results from the National Epidemiologic Survey on Alcoholism and Related Conditions. *Archives of General Psychiatry, 62*, 1097-1106. doi:10.1001/archpsyc.62.10.1097

Hauffa, R., Rief, W., Brähler, E., Martin, A., Mewes, R., & Glaesmer, H. (2011). Lifetime traumatic experiences and posttraumatic stress disorder in the German population: results of a representative population survey. *Journal of Nervous and Mental Disease, 199*, 934-939. Retrieved from http://search.ebscohost.com/login.aspx?direct=true&db=cin20&AN=108208333&site=ehost-live

Kessler, R. C., Andrade, L. H., Bijl, R. V., Offord, D. R., Demler, O. V., & Stein, D. J. (2002). The effects of co-morbidity on the onset and persistence of generalized anxiety disorder in the ICPE surveys. *Psychological Medicine, 32*, 1213-1225. doi:10.1017/s0033291702006104

Kessler, R. C., Berglund, P. A., Dewit, D. J., Bedirhan Üstün, T., Wang, P. S., & Wittchen, H.-U. (2002). Distinguishing generalized anxiety disorder from major depression: prevalence and impairment from current pure and comorbid disorders in the US and Ontario. *International Journal of Methods in Psychiatric Research, 11*, 99-111. doi:10.1002/mpr.128

Kessler, R. C., Nelson, C. B., McGonagle, K. A., Liu, J., Swartz, M., & Blazer, D. G. (1996). Comorbidity of DSM-III-R major depressive disorder in the general population: results from the US National Comorbidity Survey. *British Journal of Psychiatry. Supplement, 30*, 17-30.

Kolada, J. L., Bland, R. C., & Newman, S. C. (1994). Epidemiology of psychiatric disorders in Edmonton. Obsessive-compulsive disorder. *Acta Psychiatrica Scandinavica Suppl, 376*, 24-35.

Lewinsohn, P. M., Zinbarg, R., Seeley, J. R., Lewinsohn, M., & Sack, W. H. (1997). Lifetime comorbidity among anxiety disorders and between anxiety disorders and other mental disorders in adolescents. *Journal of Anxiety Disorders, 11*, 377-394. Retrieved from https://ac.els-cdn.com/S0887618597000170/1-

s2.0-S0887618597000170-main.pdf?_tid=84cf75c4-313d-410e-8883-6ab196cc0506&acdnat=1540420945_937c326c71619979a4e3ca28059c3da1
Lim, L., Ng, T. P., Chua, H. C., Chiam, P. C., Won, V., Lee, T., . . . Kua, E. H. (2005). Generalised anxiety disorder in Singapore: prevalence, co-morbidity and risk factors in a multi-ethnic population. *Social Psychiatry and Psychiatric Epidemiology, 40*, 972-979. doi:10.1007/s00127-005-0978-y
Martin-Merino, E., Ruigomez, A., Johansson, S., Wallander, M. A., & Garcia-Rodriguez, L. A. (2010). Study of a cohort of patients newly diagnosed with depression in general practice: prevalence, incidence, comorbidity, and treatment patterns. *Primary Care Companion to the Journal of Clinical Psychiatry, 12*, PCC.08m00764. doi:10.4088/PCC.08m00764blu
Merikangas, K. R., Angst, J., Eaton, W., Canino, G., Rubio-Stipec, M., Wacker, H., . . . Kupfer, D. J. (1996). Comorbidity and Boundaries of Affective Disorders with Anxiety Disorders and Substance Misuse: Results of an International Task Force. *British Journal of Psychiatry, 168*, 58-67. doi:10.1192/S0007125000298425
Mohammadi, M. R., Ghanizadeh, A., Mohammadi, M., & Mesgarpour, B. (2006). Prevalence of social phobia and its comorbidity with psychiatric disorders in Iran. *Depression and Anxiety, 23*, 405-411. doi:10.1002/da.20129
Mohammadi, M. R., Ghanizadeh, A., & Moini, R. (2007). Lifetime comorbidity of obsessive-compulsive disorder with psychiatric disorders in a community sample. *Depression and Anxiety, 24*, 602-607. doi:10.1002/da.20259
Rihmer, Z., Szadoczky, E., Furedi, J., Kiss, K., & Papp, Z. (2001). Anxiety disorders comorbidity in bipolar I, bipolar II and unipolar major depression: results from a population-based study in Hungary. *Journal of Affective Disorders, 67*, 175-179.
Rohde, P., Lewinsohn, P. M., & Seeley, J. R. (1991). Comorbidity of unipolar depression: II. Comorbidity with other mental disorders in adolescents and adults. *Journal of Abnormal Psychology, 100*, 214-222. doi:10.1037/0021-843X.100.2.214
Roy-Byrne, P. P., Stang, P., Wittchen, H.-U., Ustun, B., Walters, E. E., & Kessler, R. C. (2000). Lifetime panic–depression comorbidity in the National Comorbidity Survey: Association with symptoms, impairment, course and help-seeking. *British Journal of Psychiatry, 176*, 229-235. doi:10.1192/bjp.176.3.229
Ruscio, A. M., Brown, T. A., Chiu, W. T., Sareen, J., Stein, M. B., & Kessler, R. C. (2008). Social fears and social phobia in the USA: results from the National Comorbidity Survey Replication. *Psychological Medicine, 38*, 15-28. doi:10.1017/S0033291707001699
Ruscio, A. M., Stein, D. J., Chiu, W. T., & Kessler, R. C. (2010). The epidemiology of obsessive-compulsive disorder in the National Comorbidity Survey Replication. *Molecular Psychiatry, 15*, 53-63. doi:10.1038/mp.2008.94
Sahoo, S., & Khess, C. R. (2010). Prevalence of depression, anxiety, and stress among young male adults in India: a dimensional and categorical diagnoses-based study. *Journal of Nervous and Mental Disease, 198*, 901-904. doi:10.1097/NMD.0b013e3181fe75dc
Schneier, F. R., Johnson, J., Hornig, C. D., Liebowitz, M. R., & Weissman, M. M. (1992). Social Phobia: Comorbidity and Morbidity in an Epidemiologic Sample. *Archives of General Psychiatry, 49*, 282-288. doi:10.1001/archpsyc.1992.01820040034004
Spaner, D., Bland, R. C., & Newman, S. C. (1994). Epidemiology of psychiatric disorders in Edmonton. Major depressive disorder. *Acta Psychiatrica Scandinavica Suppl, 376*, 7-15.
Stein, M. B., & Heimberg, R. G. (2004). Well-being and life satisfaction in generalized anxiety disorder: comparison to major depressive disorder in a community sample. *Journal of Affective Disorders, 79*, 161-166. doi:10.1016/s0165-0327(02)00457-3
Szadoczky, E., Rihmer, Z., Papp, Z. S., Vitrai, J., & Furedi, J. (2002). Gender differences in major depressive disorder in a Hungarian community survey. *International Journal of Psychiatry in Clinical Practice, 6*, 31-37. doi:10.1080/136515002753489399
Thompson, A. H., Bland, R. C., & Orn, H. T. (1989). Relationship and chronology of depression, agoraphobia, and panic disorder in the general population. *Journal of Nervous and Mental Disease, 177*, 456-463. doi:10.1097/00005053-198908000-00002
Trumpf, J., Margraf, J., Vriends, N., Meyer, A. H., & Becker, E. S. (2010). Specific phobia predicts psychopathology in young women. *Social Psychiatry and Psychiatric Epidemiology, 45*, 1161-1166. doi:10.1007/s00127-009-0159-5
Tsuchiya, M., Kawakami, N., Ono, Y., Nakane, Y., Nakamura, Y., Tachimori, H., . . . Kessler, R. C. (2009). Lifetime comorbidities between phobic disorders and major depression in Japan: results from the World Mental Health Japan 2002-2004 Survey. *Depression and Anxiety, 26*, 949-955. doi:10.1002/da.20508

Van Ameringen, M., Mancini, C., Patterson, B., & Boyle, M. H. (2008). Post-traumatic stress disorder in Canada. *CNS Neuroscience & Therapeutics, 14*, 171-181. doi:10.1111/j.1755-5949.2008.00049.x

Weissman, M. M., Bland, R. C., Canino, G. J., Faravelli, C., Greenwald, S., Hwu, H. G., . . . Yeh, E. K. (1997). The cross-national epidemiology of panic disorder. *Archives of General Psychiatry, 54*, 305-309.

Weissman, M. M., Bland, R. C., Canino, G. J., Faravelli, C., Greenwald, S., Hwu, H. G., . . . Yeh, E. K. (1996). Cross-national epidemiology of major depression and bipolar disorder. *JAMA, 276*, 293-299.

Weissman, M. M., Bland, R. C., Canino, G. J., Greenwald, S., Hwu, H. G., Lee, C. K., . . . et al. (1994). The cross national epidemiology of obsessive compulsive disorder. The Cross National Collaborative Group. *Journal of Clinical Epidemiology, 55 Suppl*, 5-10.

Wittchen, H.-U., Zhao, S., Kessler, R. C., & Eaton, W. W. (1994). DSM-III-R Generalized Anxiety Disorder in the National Comorbidity Survey. *Archives of General Psychiatry, 51*, 355-364. doi:10.1001/archpsyc.1994.03950050015002

Wittchen, H. U., Stein, M. B., & Kessler, R. C. (1999). Social fears and social phobia in a community sample of adolescents and young adults: prevalence, risk factors and co-morbidity. *Psychological Medicine, 29*, 309-323. doi:undefined

eReference 6. DEP and anxiety disorder: period prevalence comorbidity (68 studies)

Adam, Y., Meinlschmidt, G., Gloster, A. T., & Lieb, R. (2012). Obsessive-compulsive disorder in the community: 12-month prevalence, comorbidity and impairment. *Social Psychiatry and Psychiatric Epidemiology, 47*, 339-349. doi:10.1007/s00127-010-0337-5

Alonso, J., Angermeyer, M. C., Bernert, S., Bruffaerts, R., Brugha, T. S., Bryson, H., . . . Vollebergh, W. A. M. (2004). 12-Month comorbidity patterns and associated factors in Europe: results from the European Study of the Epidemiology of Mental Disorders (ESEMeD) project. *Acta Psychiatrica Scandinavica, 109*, 28-37. doi:10.1111/j.1600-0047.2004.00328.x

Alvarenga, P. G., do Rosario, M. C., Cesar, R. C., Manfro, G. G., Moriyama, T. S., Bloch, M. H., . . . Miguel, E. C. (2016). Obsessive-compulsive symptoms are associated with psychiatric comorbidities, behavioral and clinical problems: a population-based study of Brazilian school children. *European Child and Adolescent Psychiatry, 25*, 175-182. doi:10.1007/s00787-015-0723-3

Andrews, G., Slade, T., & Issakidis, C. (2002). Deconstructing current comorbidity: data from the Australian National Survey of Mental Health and Well-Being. *British Journal of Psychiatry, 181*, 306-314. doi:10.1192/bjp.181.4.306

Angst, J., Gamma, A., Endrass, J., Hantouche, E., Goodwin, R., Ajdacic, V., . . . Rossler, W. (2005). Obsessive-compulsive syndromes and disorders: significance of comorbidity with bipolar and anxiety syndromes. *European Archives of Psychiatry and Clinical Neuroscience, 255*, 65-71. doi:10.1007/s00406-005-0576-8

Beekman, A. T., de Beurs, E., van Balkom, A. J., Deeg, D. J., van Dyck, R., & van Tilburg, W. (2000). Anxiety and depression in later life: Co-occurrence and communality of risk factors. *American Journal of Psychiatry, 157*, 89-95. doi:10.1176/ajp.157.1.89

Biederman, J., Petty, C., Faraone, S. V., Hirshfeld-Becker, D. R., Henin, A., Pollack, M. H., & Rosenbaum, J. F. (2005). Patterns of comorbidity in panic disorder and major depression: findings from a nonreferred sample. *Depression and Anxiety, 21*, 55-60. doi:10.1002/da.20055

Boyd, J. H., Burke, J. D., Jr., Gruenberg, E., Holzer, C. E., III, Rae, D. S., George, L. K., . . . Nestadt, G. (1984). Exclusion Criteria of DSM-III: A Study of Co-occurrence of Hierarchy-Free Syndromes. *Archives of General Psychiatry, 41*, 983-989. doi:10.1001/archpsyc.1984.01790210065008

Cairney, J., Corna, L. M., Veldhuizen, S., Herrmann, N., & Streiner, D. L. (2008). Comorbid depression and anxiety in later life: patterns of association, subjective well-being, and impairment. *American Journal of Geriatric Psychiatry, 16*, 201-208. doi:10.1097/JGP.0b013e3181602a4a

Cairney, J., McCabe, L., Veldhuizen, S., Corna, L. M., Streiner, D., & Herrmann, N. (2007). Epidemiology of Social Phobia in Later Life. *American Journal of Geriatric Psychiatry, 15*, 224-233. doi:https://doi.org/10.1097/01.JGP.0000235702.77245.46

Carter, R. M., Wittchen, H. U., Pfister, H., & Kessler, R. C. (2001). One-year prevalence of subthreshold and threshold DSM-IV generalized anxiety disorder in a nationally representative sample. *Depression and Anxiety, 13*, 78-88.

Chen, Y., Bennett, D., Clarke, R., Guo, Y., Yu, C., Bian, Z., . . . Chen, Z. (2017). Patterns and correlates of major depression in Chinese adults: a cross-sectional study of 0.5 million men and women. *Psychological Medicine, 47*, 958-970. doi:10.1017/s0033291716002889

Chuan, S. K., Kumar, R., Matthew, N., Heok, K. E., & Pin, N. T. (2008). Subsyndromal depression in old age: clinical significance and impact in a multi-ethnic community sample of elderly Singaporeans. *International Psychogeriatrics, 20*, 188-200. doi:10.1017/s1041610207006187

Corna, L. M., Cairney, J., Herrmann, N., Veldhuizen, S., McCabe, L., & Streiner, D. (2007). Panic disorder in later life: results from a national survey of Canadians. *International Psychogeriatrics, 19*, 1084-1096. doi:10.1017/s1041610207004978

Douglass, H. M., Moffitt, T. E., Dar, R., McGee, R., & Silva, P. (1995). Obsessive-compulsive disorder in a birth cohort of 18-year-olds: prevalence and predictors. *Journal of the American Academy of Child and Adolescent Psychiatry, 34*, 1424-1431. doi:10.1097/00004583-199511000-00008

Faravelli, C., Abrardi, L., Bartolozzi, D., Cecchi, C., Cosci, F., D'Adamo, D., . . . Rosi, S. (2004). The Sesto Fiorentino Study: Background, Methods and Preliminary Results. *Psychotherapy and Psychosomatics, 73*, 216-225. doi:10.1159/000077740

Fehm, L., Beesdo, K., Jacobi, F., & Fiedler, A. (2008). Social anxiety disorder above and below the diagnostic threshold: prevalence, comorbidity and impairment in the general population. *Social Psychiatry and Psychiatric Epidemiology, 43*, 257-265. doi:10.1007/s00127-007-0299-4

Fineberg, N. A., Hengartner, M. P., Bergbaum, C., Gale, T., Rossler, W., & Angst, J. (2013). Lifetime comorbidity of obsessive-compulsive disorder and sub-threshold obsessive-compulsive symptomatology in the community: impact, prevalence, socio-demographic and clinical characteristics. *International Journal of Psychiatry in Clinical Practice, 17*, 188-196. doi:10.3109/13651501.2013.777745

Ford, T., Goodman, R., & Meltzer, H. (2003). The British Child and Adolescent Mental Health Survey 1999: The Prevalence of DSM-IV Disorders. *Journal of the American Academy of Child and Adolescent Psychiatry, 42*, 1203-1211. doi:https://doi.org/10.1097/00004583-200310000-00011

Gabilondo, A., Rojas-Farreras, S., Vilagut, G., Haro, J. M., Fernandez, A., Pinto-Meza, A., & Alonso, J. (2010). Epidemiology of major depressive episode in a southern European country: results from the ESEMeD-Spain project. *Journal of Affective Disorders, 120*, 76-85. doi:10.1016/j.jad.2009.04.016

Glaesmer, H., Kaiser, M., Braehler, E., Freyberger, H. J., & Kuwert, P. (2012). Posttraumatic stress disorder and its comorbidity with depression and somatisation in the elderly - a German community-based study. *Aging & Mental Health, 16*, 403-412. doi:10.1080/13607863.2011.615740

Goncalves, D. C., Pachana, N. A., & Byrne, G. J. (2011). Prevalence and correlates of generalized anxiety disorder among older adults in the Australian National Survey of Mental Health and Well-Being. *Journal of Affective Disorders, 132*, 223-230. doi:10.1016/j.jad.2011.02.023

Gureje, O., Uwakwe, R., Oladeji, B., Makanjuola, V. O., & Esan, O. (2010). Depression in adult Nigerians: results from the Nigerian Survey of Mental Health and Well-being. *Journal of Affective Disorders, 120*, 158-164. doi:10.1016/j.jad.2009.04.030

Hasin, D. S., Goodwin, R. D., Stinson, F. S., & Grant, B. F. (2005). Epidemiology of major depressive disorder: results from the National Epidemiologic Survey on Alcoholism and Related Conditions. *Archives of General Psychiatry, 62*, 1097-1106. doi:10.1001/archpsyc.62.10.1097

Hunt, C., Issakidis, C., & Andrews, G. (2002). DSM-IV generalized anxiety disorder in the Australian National Survey of Mental Health and Well-Being. *Psychological Medicine, 32*, 649-659. doi:10.1017/S0033291702005512

Jaffee, S. R., Moffitt, T. E., Caspi, A., Fombonne, E., Poulton, R., & Martin, J. (2002). Differences in Early Childhood Risk Factors for Juvenile-Onset and Adult-Onset Depression. *Archives of General Psychiatry, 59*, 215-222. doi:10.1001/archpsyc.59.3.215

Jeon, H. J., Suh, T., Lee, H. J., Hahm, B. J., Lee, J. Y., Cho, S. J., . . . Cho, M. J. (2007). Partial versus full PTSD in the Korean community: prevalence, duration, correlates, comorbidity, and dysfunctions. *Depression and Anxiety, 24*, 577-585. doi:10.1002/da.20270

Kang, H. J., Bae, K. Y., Kim, S. W., Shin, I. S., Yoon, J. S., & Kim, J. M. (2016). Anxiety symptoms in Korean elderly individuals: a two-year longitudinal community study. *International Psychogeriatrics, 28*, 423-433. doi:10.1017/s1041610215001301

Kashani, J. H., & Orvaschel, H. (1988). Anxiety disorders in mid-adolescence: A community sample. *American Journal of Psychiatry, 145*, 960.

Katona, C. L. E., Manela, M. V., & Livingston, G. A. (1997). Comorbidity with depression in older people: The Islington Study. *Aging & Mental Health, 1*, 57-62. doi:10.1080/13607869757380

Kessler, R. C., Andrade, L. H., Bijl, R. V., Offord, D. R., Demler, O. V., & Stein, D. J. (2002). The effects of co-morbidity on the onset and persistence of generalized anxiety disorder in the ICPE surveys. *Psychological Medicine, 32*, 1213-1225. doi:10.1017/s0033291702006104

Kessler, R. C., Berglund, P. A., Dewit, D. J., Bedirhan Üstün, T., Wang, P. S., & Wïttchen, H.-U. (2002).

Distinguishing generalized anxiety disorder from major depression: prevalence and impairment from current pure and comorbid disorders in the US and Ontario. *International Journal of Methods in Psychiatric Research, 11*, 99-111. doi:10.1002/mpr.128

Kessler, R. C., DuPont, R. L., Berglund, P., & Wittchen, H. U. (1999). Impairment in pure and comorbid generalized anxiety disorder and major depression at 12 months in two national surveys. *American Journal of Psychiatry, 156*, 1915-1923. doi:10.1176/ajp.156.12.1915

Kessler, R. C., Nelson, C. B., McGonagle, K. A., Liu, J., Swartz, M., & Blazer, D. G. (1996). Comorbidity of DSM-III-R major depressive disorder in the general population: results from the US National Comorbidity Survey. *British Journal of Psychiatry. Supplement, 30*, 17-30.

Lampe, L., Slade, T., Issakidis, C., & Andrews, G. (2003). Social phobia in the Australian National Survey of Mental Health and Well-Being (NSMHWB). *Psychological Medicine, 33*, 637-646.

Leray, E., Camara, A., Drapier, D., Riou, F., Bougeant, N., Pelissolo, A., . . . Millet, B. (2011). Prevalence, characteristics and comorbidities of anxiety disorders in France: results from the "Mental Health in General Population" survey (MHGP). *European Psychiatry, 26*, 339-345. doi:10.1016/j.eurpsy.2009.12.001

Lim, L., Ng, T. P., Chua, H. C., Chiam, P. C., Won, V., Lee, T., . . . Kua, E. H. (2005). Generalised anxiety disorder in Singapore: prevalence, co-morbidity and risk factors in a multi-ethnic population. *Social Psychiatry and Psychiatric Epidemiology, 40*, 972-979. doi:10.1007/s00127-005-0978-y

Magklara, K., Bellos, S., Niakas, D., Stylianidis, S., Kolaitis, G., Mavreas, V., & Skapinakis, P. (2015). Depression in late adolescence: a cross-sectional study in senior high schools in Greece. *BMC Psychiatry, 15*, 199. doi:10.1186/s12888-015-0584-9

McCabe, L., Cairney, J., Veldhuizen, S., Herrmann, N., & Streiner, D. L. (2006). Prevalence and Correlates of Agoraphobia in Older Adults. *American Journal of Geriatric Psychiatry, 14*, 515-522. doi:https://doi.org/10.1097/01.JGP.0000203177.54242.14

McEvoy, P. M., Grove, R., & Slade, T. (2011). Epidemiology of anxiety disorders in the Australian general population: findings of the 2007 Australian National Survey of Mental Health and Wellbeing. *Australian and New Zealand Journal of Psychiatry, 45*, 957-967. doi:10.3109/00048674.2011.624083

Mergl, R., Seidscheck, I., Allgaier, A. K., Moller, H. J., Hegerl, U., & Henkel, V. (2007). Depressive, anxiety, and somatoform disorders in primary care: prevalence and recognition. *Depression and Anxiety, 24*, 185-195. doi:10.1002/da.20192

Merikangas, K. R., Zhang, H., Avenevoli, S., Acharyya, S., Neuenschwander, M., & Angst, J. (2003). Longitudinal trajectories of depression and anxiety in a prospective community study: the Zurich Cohort Study. *Archives of General Psychiatry, 60*, 993-1000. doi:10.1001/archpsyc.60.9.993

Moffitt, T. E., Harrington, H., Caspi, A., Kim-Cohen, J., Goldberg, D., Gregory, A. M., & Poulton, R. (2007). Depression and generalized anxiety disorder: cumulative and sequential comorbidity in a birth cohort followed prospectively to age 32 years. *Archives of General Psychiatry, 64*, 651-660. doi:10.1001/archpsyc.64.6.651

Munyandamutsa, N., Mahoro Nkubamugisha, P., Gex-Fabry, M., & Eytan, A. (2012). Mental and physical health in Rwanda 14 years after the genocide. *Social Psychiatry and Psychiatric Epidemiology, 47*, 1753-1761. doi:10.1007/s00127-012-0494-9

Murphy, J. M., Horton, N. J., Laird, N. M., Monson, R. R., Sobol, A. M., & Leighton, A. H. (2004). Anxiety and depression: a 40-year perspective on relationships regarding prevalence, distribution, and comorbidity. *Acta Psychiatrica Scandinavica, 109*, 355-375. doi:10.1111/j.1600-0447.2003.00286.x

Ohayon, M. M., & Schatzberg, A. F. (2010). Social phobia and depression: prevalence and comorbidity. *Journal of Psychosomatic Research, 68*, 235-243. doi:10.1016/j.jpsychores.2009.07.018

Preville, M., Boyer, R., Grenier, S., Dube, M., Voyer, P., Punti, R., . . . Brassard, J. (2008). The epidemiology of psychiatric disorders in Quebec's older adult population. *Canadian Journal of Psychiatry, 53*, 822-832. doi:10.1177/070674370805301208

Prina, A. M., Ferri, C. P., Guerra, M., Brayne, C., & Prince, M. (2011). Co-occurrence of anxiety and depression amongst older adults in low- and middle-income countries: findings from the 10/66 study. *Psychological Medicine, 41*, 2047-2056. doi:10.1017/s0033291711000444

Ritchie, K., Norton, J., Mann, A., Carrière, I., & Ancelin, M.-L. (2013). Late-Onset Agoraphobia: General Population Incidence and Evidence for a Clinical Subtype. *American Journal of Psychiatry, 170*, 790-798. doi:10.1176/appi.ajp.2013.12091235

Rohde, P., Lewinsohn, P. M., & Seeley, J. R. (1991). Comorbidity of unipolar depression: II. Comorbidity with other mental disorders in adolescents and adults. *Journal of Abnormal Psychology, 100*, 214-222. doi:10.1037/0021-843X.100.2.214

Romano, E., Tremblay, R. E., Vitaro, F., Zoccolillo, M., & Pagani, L. (2005). Sex and informant effects on diagnostic comorbidity in an adolescent community sample. *Canadian Journal of Psychiatry, 50*, 479-489. doi:10.1177/070674370505000808

Roy-Byrne, P. P., Stang, P., Wittchen, H.-U., Ustun, B., Walters, E. E., & Kessler, R. C. (2000). Lifetime panic–depression comorbidity in the National Comorbidity Survey: Association with symptoms, impairment, course and help-seeking. *British Journal of Psychiatry, 176*, 229-235. doi:10.1192/bjp.176.3.229

Rueda-Jaimes, G. E., Camacho Lopez, P. A., & Navarro-Mancilla, A. A. (2008). [Prevalence of Obsessive Compulsive Disorder and its comorbidity with Major Depressive Disorder in adolescent students]. *Vertex, 19*, 5-9.

Sartorius, N., Ustun, T. B., Lecrubier, Y., & Wittchen, H. U. (1996). Depression comorbid with anxiety: results from the WHO study on psychological disorders in primary health care. *British Journal of Psychiatry. Supplement*, 38-43.

Schaub, R. T., & Linden, M. (2000). Anxiety and anxiety disorders in the old and very old—Results from the Berlin aging study (BASE). *Comprehensive Psychiatry, 41*, 48-54. doi:https://doi.org/10.1016/S0010-440X(00)80008-5

Schoevers, R. A., Beekman, A. T., Deeg, D. J., Jonker, C., & van Tilburg, W. (2003). Comorbidity and risk-patterns of depression, generalised anxiety disorder and mixed anxiety-depression in later life: results from the AMSTEL study. *International Journal of Geriatric Psychiatry, 18*, 994-1001. doi:10.1002/gps.1001

Schrier, A. C., de Wit, M. A., Coupe, V. M., Fassaert, T., Verhoeff, A. P., Kupka, R. W., . . . Beekman, A. T. (2012). Comorbidity of anxiety and depressive disorders: a comparative population study in Western and non-Western inhabitants in the Netherlands. *International Journal of Social Psychiatry, 58*, 186-194. doi:10.1177/0020764010390433

Scott, K. M., McGee, M. A., Oakley Browne, M. A., & Wells, J. E. (2006). Mental disorder comorbidity in Te Rau Hinengaro: the New Zealand Mental Health Survey. *Australian and New Zealand Journal of Psychiatry, 40*, 875-881. doi:10.1080/j.1440-1614.2006.01906.x

Serrano-Blanco, A., Palao, D. J., Luciano, J. V., Pinto-Meza, A., Lujan, L., Fernandez, A., . . . Haro, J. M. (2010). Prevalence of mental disorders in primary care: results from the diagnosis and treatment of mental disorders in primary care study (DASMAP). *Social Psychiatry and Psychiatric Epidemiology, 45*, 201-210. doi:10.1007/s00127-009-0056-y

Sicras-Mainar, A., Blanca-Tamayo, M., Navarro-Artieda, R., Pizarro-Paixa, I., & Gomez-Lus Centelles, S. (2008). [Influence of morbidity and the use of health resources in patients who require care for generalised anxiety disorder in the primary health care setting]. *Atencion Primarie, 40*, 603-610.

Stein, M. B., & Heimberg, R. G. (2004). Well-being and life satisfaction in generalized anxiety disorder: comparison to major depressive disorder in a community sample. *Journal of Affective Disorders, 79*, 161-166. doi:10.1016/s0165-0327(02)00457-3

Stylianidis, S., Pantelidou, S., Chondros, P., Roelandt, J. L., & Barbato, A. (2014). Prevalence of mental disorders in a Greek island. *Psychiatriki, 25*, 19-26.

Vaiva, G., Jehel, L., Cottencin, O., Ducrocq, F., Duchet, C., Omnes, C., . . . Roelandt, J. L. (2008). [Prevalence of trauma-related disorders in the French WHO study: Sante mentale en population generale (SMPG)]. *Encephale, 34*, 577-583. doi:10.1016/j.encep.2007.11.006

van Balkom, A. J., Beekman, A. T., de Beurs, E., Deeg, D. J., van Dyck, R., & van Tilburg, W. (2000). Comorbidity of the anxiety disorders in a community-based older population in The Netherlands. *Acta Psychiatrica Scandinavica, 101*, 37-45.

van Loo, H. M., Schoevers, R. A., Kendler, K. S., de Jonge, P., & Romeijn, J. W. (2016). Psychiatric comorbidity does not only depend on diagnostic thresholds: an illustration with major depressive disorder and generalized anxiety disorder. *Depression and Anxiety, 33*, 143-152. doi:10.1002/da.22453

Wittchen, H.-U., Carter, R. M., Pfister, H., Montgomery, S. A., & Kessler, R. C. (2000). Disabilities and quality of life in pure and comorbid generalized anxiety disorder and major depression in a national survey. *International Clinical Psychopharmacology, 15*, 319-328. doi:10.1097/00004850-200015060-00002

Wittchen, H.-U., Zhao, S., Kessler, R. C., & Eaton, W. W. (1994). DSM-III-R Generalized Anxiety Disorder in the National Comorbidity Survey. *Archives of General Psychiatry, 51*, 355-364. doi:10.1001/archpsyc.1994.03950050015002

Zhang, X., Norton, J., Carriere, I., Ritchie, K., Chaudieu, I., & Ancelin, M. L. (2015). Risk factors for late-onset generalized anxiety disorder: results from a 12-year prospective cohort (the ESPRIT study). *Translational Psychiatry, 5*, e536. doi:10.1038/tp.2015.31

eReference 7. DYS and anxiety disorder: lifetime comorbidity (16 studies)

Chartier, M. J., Walker, J. R., & Stein, M. B. (2003). Considering comorbidity in social phobia. *Social Psychiatry and Psychiatric Epidemiology, 38*, 728-734. doi:10.1007/s00127-003-0720-6

Chou, K.-L. (2009). Specific Phobia in Older Adults: Evidence From the National Epidemiologic Survey on Alcohol and Related Conditions. *American Journal of Geriatric Psychiatry, 17*, 376-386. doi:https://doi.org/10.1097/JGP.0b013e3181943214

Chou, K. L. (2009). Social anxiety disorder in older adults: evidence from the National Epidemiologic Survey on alcohol and related conditions. *Journal of Affective Disorders, 119*, 76-83. doi:10.1016/j.jad.2009.04.002

de Graaf, R., Bijl, R. V., Spijker, J., Beekman, A. T., & Vollebergh, W. A. (2003). Temporal sequencing of lifetime mood disorders in relation to comorbid anxiety and substance use disorders--findings from the Netherlands Mental Health Survey and Incidence Study. *Social Psychiatry and Psychiatric Epidemiology, 38*, 1-11. doi:10.1007/s00127-003-0597-4

Faravelli, C., Abrardi, L., Bartolozzi, D., Cecchi, C., Cosci, F., D'Adamo, D., . . . Rosi, S. (2004). The Sesto Fiorentino Study: Background, Methods and Preliminary Results. *Psychotherapy and Psychosomatics, 73*, 216-225. doi:10.1159/000077740

Faravelli, C., Abrardi, L., Bartolozzi, D., Cecchi, C., Cosci, F., D'Adamo, D., . . . Rosi, S. (2004). The Sesto Fiorentino Study: Point and One-Year Prevalences of Psychiatric Disorders in an Italian Community Sample Using Clinical Interviewers. *Psychotherapy and Psychosomatics, 73*, 226-234. doi:10.1159/000077741

Grabe, H. J., Meyer, C., Hapke, U., Rumpf, H. J., Freyberger, H. J., Dilling, H., & John, U. (2001). Lifetime-comorbidity of obsessive-compulsive disorder and subclinical obsessive-compulsive disorder in Northern Germany. *European Archives of Psychiatry and Clinical Neuroscience, 251*, 130-135.

Kessler, R. C., Andrade, L. H., Bijl, R. V., Offord, D. R., Demler, O. V., & Stein, D. J. (2002). The effects of co-morbidity on the onset and persistence of generalized anxiety disorder in the ICPE surveys. *Psychological Medicine, 32*, 1213-1225. doi:10.1017/s0033291702006104

Kessler, R. C., Berglund, P. A., Dewit, D. J., Bedirhan Üstün, T., Wang, P. S., & Wïttchen, H.-U. (2002). Distinguishing generalized anxiety disorder from major depression: prevalence and impairment from current pure and comorbid disorders in the US and Ontario. *International Journal of Methods in Psychiatric Research, 11*, 99-111. doi:10.1002/mpr.128

Kolada, J. L., Bland, R. C., & Newman, S. C. (1994). Epidemiology of psychiatric disorders in Edmonton. Obsessive-compulsive disorder. *Acta Psychiatrica Scandinavica Suppl, 376*, 24-35.

Lim, L., Ng, T. P., Chua, H. C., Chiam, P. C., Won, V., Lee, T., . . . Kua, E. H. (2005). Generalised anxiety disorder in Singapore: prevalence, co-morbidity and risk factors in a multi-ethnic population. *Social Psychiatry and Psychiatric Epidemiology, 40*, 972-979. doi:10.1007/s00127-005-0978-y

Magee, W. J., Eaton, W. W., Wittchen, H.-U., McGonagle, K. A., & Kessler, R. C. (1996). Agoraphobia, Simple Phobia, and Social Phobia in the National Comorbidity Survey. *Archives of General Psychiatry, 53*, 159-168. doi:10.1001/archpsyc.1996.01830020077009

Ruscio, A. M., Brown, T. A., Chiu, W. T., Sareen, J., Stein, M. B., & Kessler, R. C. (2008). Social fears and social phobia in the USA: results from the National Comorbidity Survey Replication. *Psychological Medicine, 38*, 15-28. doi:10.1017/S0033291707001699

Schneier, F. R., Johnson, J., Hornig, C. D., Liebowitz, M. R., & Weissman, M. M. (1992). Social Phobia: Comorbidity and Morbidity in an Epidemiologic Sample. *Archives of General Psychiatry, 49*, 282-288. doi:10.1001/archpsyc.1992.01820040034004

Wittchen, H.-U., Zhao, S., Kessler, R. C., & Eaton, W. W. (1994). DSM-III-R Generalized Anxiety Disorder in the National Comorbidity Survey. *Archives of General Psychiatry, 51*, 355-364. doi:10.1001/archpsyc.1994.03950050015002

Wittchen, H. U., Stein, M. B., & Kessler, R. C. (1999). Social fears and social phobia in a community sample of adolescents and young adults: prevalence, risk factors and co-morbidity. *Psychological Medicine, 29*, 309-323. doi:undefined

eReference 8. DYS and anxiety disorder: period prevalence comorbidity (17 studies)

Alonso, J., Angermeyer, M. C., Bernert, S., Bruffaerts, R., Brugha, T. S., Bryson, H., . . . Vollebergh, W. A. M. (2004). 12-Month comorbidity patterns and associated factors in Europe: results from the European

Study of the Epidemiology of Mental Disorders (ESEMeD) project. *Acta Psychiatrica Scandinavica, 109*, 28-37. doi:10.1111/j.1600-0047.2004.00328.x

Andrews, G., Slade, T., & Issakidis, C. (2002). Deconstructing current comorbidity: data from the Australian National Survey of Mental Health and Well-Being. *British Journal of Psychiatry, 181*, 306-314. doi:10.1192/bjp.181.4.306

Autonell, J., Vila, F., Pinto-Meza, A., Vilagut, G., Codony, M., Almansa, J., . . . Haro, J. M. (2007). [One year prevalence of mental disorders comorbidity and associated socio-demographic risk factors in the general population of Spain. Results of the ESEMeD-Spain study]. *Actas Españolas de Psiquiatría, 35 Suppl 2*, 4-11. Retrieved from https://translate.googleusercontent.com/translate_f

Carter, R. M., Wittchen, H. U., Pfister, H., & Kessler, R. C. (2001). One-year prevalence of subthreshold and threshold DSM-IV generalized anxiety disorder in a nationally representative sample. *Depression and Anxiety, 13*, 78-88.

Douglass, H. M., Moffitt, T. E., Dar, R., McGee, R., & Silva, P. (1995). Obsessive-compulsive disorder in a birth cohort of 18-year-olds: prevalence and predictors. *Journal of the American Academy of Child and Adolescent Psychiatry, 34*, 1424-1431. doi:10.1097/00004583-199511000-00008

Faravelli, C., Abrardi, L., Bartolozzi, D., Cecchi, C., Cosci, F., D'Adamo, D., . . . Rosi, S. (2004). The Sesto Fiorentino Study: Background, Methods and Preliminary Results. *Psychotherapy and Psychosomatics, 73*, 216-225. doi:10.1159/000077740

Faravelli, C., Abrardi, L., Bartolozzi, D., Cecchi, C., Cosci, F., D'Adamo, D., . . . Rosi, S. (2004). The Sesto Fiorentino Study: Point and One-Year Prevalences of Psychiatric Disorders in an Italian Community Sample Using Clinical Interviewers. *Psychotherapy and Psychosomatics, 73*, 226-234. doi:10.1159/000077741

Fehm, L., Beesdo, K., Jacobi, F., & Fiedler, A. (2008). Social anxiety disorder above and below the diagnostic threshold: prevalence, comorbidity and impairment in the general population. *Social Psychiatry and Psychiatric Epidemiology, 43*, 257-265. doi:10.1007/s00127-007-0299-4

Grant, B. F., Hasin, D. S., Blanco, C., Stinson, F. S., Chou, S. P., Goldstein, R. B., . . . Huang, B. (2005). The epidemiology of social anxiety disorder in the United States: results from the National Epidemiologic Survey on Alcohol and Related Conditions. *Journal of Clinical Epidemiology, 66*, 1351-1361. Retrieved from http://www.psychiatrist.com/jcp/article/pages/2005/v66n11/v66n1102.aspx

Grant, B. F., Hasin, D. S., Stinson, F. S., Dawson, D. A., June Ruan, W., Goldstein, R. B., . . . Huang, B. (2005). Prevalence, correlates, co-morbidity, and comparative disability of DSM-IV generalized anxiety disorder in the USA: results from the National Epidemiologic Survey on Alcohol and Related Conditions. *Psychological Medicine, 35*, 1747-1759. doi:10.1017/s0033291705006069

Hunt, C., Issakidis, C., & Andrews, G. (2002). DSM-IV generalized anxiety disorder in the Australian National Survey of Mental Health and Well-Being. *Psychological Medicine, 32*, 649-659. doi:10.1017/S0033291702005512

Lampe, L., Slade, T., Issakidis, C., & Andrews, G. (2003). Social phobia in the Australian National Survey of Mental Health and Well-Being (NSMHWB). *Psychological Medicine, 33*, 637-646.

Lim, L., Ng, T. P., Chua, H. C., Chiam, P. C., Won, V., Lee, T., . . . Kua, E. H. (2005). Generalised anxiety disorder in Singapore: prevalence, co-morbidity and risk factors in a multi-ethnic population. *Social Psychiatry and Psychiatric Epidemiology, 40*, 972-979. doi:10.1007/s00127-005-0978-y

Scott, K. M., McGee, M. A., Oakley Browne, M. A., & Wells, J. E. (2006). Mental disorder comorbidity in Te Rau Hinengaro: the New Zealand Mental Health Survey. *Australian and New Zealand Journal of Psychiatry, 40*, 875-881. doi:10.1080/j.1440-1614.2006.01906.x

Serrano-Blanco, A., Palao, D. J., Luciano, J. V., Pinto-Meza, A., Lujan, L., Fernandez, A., . . . Haro, J. M. (2010). Prevalence of mental disorders in primary care: results from the diagnosis and treatment of mental disorders in primary care study (DASMAP). *Social Psychiatry and Psychiatric Epidemiology, 45*, 201-210. doi:10.1007/s00127-009-0056-y

Vaiva, G., Jehel, L., Cottencin, O., Ducrocq, F., Duchet, C., Omnes, C., . . . Roelandt, J. L. (2008). [Prevalence of trauma-related disorders in the French WHO study: Sante mentale en population generale (SMPG)]. *Encephale, 34*, 577-583. doi:10.1016/j.encep.2007.11.006

Wittchen, H.-U., Zhao, S., Kessler, R. C., & Eaton, W. W. (1994). DSM-III-R Generalized Anxiety Disorder in the National Comorbidity Survey. *Archives of General Psychiatry, 51*, 355-364. doi:10.1001/archpsyc.1994.03950050015002

eReference 9. BIPOLAR and anxiety disorder: lifetime comorbidity (28 studies)

Blanco, C., Compton, W. M., Saha, T. D., Goldstein, B. I., Ruan, W. J., Huang, B., & Grant, B. F. (2017). Epidemiology of DSM-5 bipolar I disorder: Results from the National Epidemiologic Survey on Alcohol and Related Conditions - III. *Journal of Psychiatric Research, 84*, 310-317. doi:10.1016/j.jpsychires.2016.10.003

Cederlof, M., Lichtenstein, P., Larsson, H., Boman, M., Ruck, C., Landen, M., & Mataix-Cols, D. (2015). Obsessive-Compulsive Disorder, Psychosis, and Bipolarity: A Longitudinal Cohort and Multigenerational Family Study. *Schizophrenia Bulletin, 41*, 1076-1083. doi:10.1093/schbul/sbu169

Chartier, M. J., Walker, J. R., & Stein, M. B. (2003). Considering comorbidity in social phobia. *Social Psychiatry and Psychiatric Epidemiology, 38*, 728-734. doi:10.1007/s00127-003-0720-6

Chen, Y. W., & Dilsaver, S. C. (1995). Comorbidity of panic disorder in bipolar illness: evidence from the Epidemiologic Catchment Area Survey. *American Journal of Psychiatry, 152*, 280-282. doi:10.1176/ajp.152.2.280

Chou, K.-L. (2009). Specific Phobia in Older Adults: Evidence From the National Epidemiologic Survey on Alcohol and Related Conditions. *American Journal of Geriatric Psychiatry, 17*, 376-386. doi:https://doi.org/10.1097/JGP.0b013e3181943214

de Graaf, R., Bijl, R. V., Spijker, J., Beekman, A. T., & Vollebergh, W. A. (2003). Temporal sequencing of lifetime mood disorders in relation to comorbid anxiety and substance use disorders--findings from the Netherlands Mental Health Survey and Incidence Study. *Social Psychiatry and Psychiatric Epidemiology, 38*, 1-11. doi:10.1007/s00127-003-0597-4

Depla, M. F. I. A., ten Have, M. L., van Balkom, A. J. L. M., & de Graaf, R. (2008). Specific fears and phobias in the general population: Results from the Netherlands Mental Health Survey and Incidence Study (NEMESIS). *Social Psychiatry and Psychiatric Epidemiology, 43*, 200-208. doi:10.1007/s00127-007-0291-z

Goodwin, R. D., Lieb, R., Hoefler, M., Pfister, H., Bittner, A., Beesdo, K., & Wittchen, H. U. (2004). Panic attack as a risk factor for severe psychopathology. *American Journal of Psychiatry, 161*, 2207-2214. doi:10.1176/appi.ajp.161.12.2207

Grabe, H. J., Meyer, C., Hapke, U., Rumpf, H. J., Freyberger, H. J., Dilling, H., & John, U. (2001). Lifetime-comorbidity of obsessive-compulsive disorder and subclinical obsessive-compulsive disorder in Northern Germany. *European Archives of Psychiatry and Clinical Neuroscience, 251*, 130-135.

Grant, B. F., Hasin, D. S., Stinson, F. S., Dawson, D. A., June Ruan, W., Goldstein, R. B., . . . Huang, B. (2005). Prevalence, correlates, co-morbidity, and comparative disability of DSM-IV generalized anxiety disorder in the USA: results from the National Epidemiologic Survey on Alcohol and Related Conditions. *Psychological Medicine, 35*, 1747-1759. doi:10.1017/s0033291705006069

Grant, B. F., Stinson, F. S., Hasin, D. S., Dawson, D. A., Chou, S. P., Ruan, W. J., & Huang, B. (2005). Prevalence, correlates, and comorbidity of bipolar I disorder and axis I and II disorders: results from the National Epidemiologic Survey on Alcohol and Related Conditions. *Journal of Clinical Epidemiology, 66*, 1205-1215. Retrieved from http://www.psychiatrist.com/jcp/article/pages/2005/v66n10/v66n1001.aspx

Kessler, R. C., Rubinow, D. R. H., C. Abelson, J. M., & Zhao, S. (1997). The epidemiology of DSM-III-R bipolar I disorder in a general population survey. *Psychological Medicine, 27*, 1079-1089. doi:10.1017/S0033291797005333

Kessler, R. C., Stang, P., Wittchen, H. U., Stein, M., & Walters, E. E. (1999). Lifetime co-morbidities between social phobia and mood disorders in the US National Comorbidity Survey. *Psychological Medicine, 29*, 555-567.

Kim, J. H., Chang, S. M., Hong, J. P., Bae, J. N., Cho, S. J., Hahm, B. J., . . . Cho, M. J. (2016). Lifetime prevalence, sociodemographic correlates, and diagnostic overlaps of bipolar spectrum disorder in the general population of South Korea. *Journal of Affective Disorders, 203*, 248-255. doi:10.1016/j.jad.2016.06.017

Lewinsohn, P. M., Hops, H., Roberts, R. E., Seeley, J. R., & Andrews, J. A. (1993). Adolescent psychopathology: I. Prevalence and incidence of depression and other DSM-III-R disorders in high school students. *Journal of Abnormal Psychology, 102*, 133-144.

Lewinsohn, P. M., Klein, D. N., & Seeley, J. R. (1995). Bipolar disorders in a community sample of older adolescents: prevalence, phenomenology, comorbidity, and course. *Journal of the American Academy of Child and Adolescent Psychiatry, 34*, 454-463. Retrieved from https://ac.els-cdn.com/S089085670963731X/1-s2.0-S089085670963731X-main.pdf?_tid=9d3967d0-ec0a-4a4e-8e8b-b88f5a06bcdb&acdnat=1540420949_0deb824a36925a6b12090df788fdc075

Lewinsohn, P. M., Zinbarg, R., Seeley, J. R., Lewinsohn, M., & Sack, W. H. (1997). Lifetime comorbidity among anxiety disorders and between anxiety disorders and other mental disorders in adolescents. *Journal of Anxiety Disorders, 11*, 377-394. Retrieved from https://ac.els-cdn.com/S0887618597000170/1-s2.0-S0887618597000170-main.pdf?_tid=84cf75c4-313d-410e-8883-6ab196cc0506&acdnat=1540420945_937c326c71619979a4e3ca28059c3da1

Merikangas, K. R., Jin, R., He, J. P., Kessler, R. C., Lee, S., Sampson, N. A., . . . Zarkov, Z. (2011). Prevalence and correlates of bipolar spectrum disorder in the world mental health survey initiative. *Archives of General Psychiatry, 68*, 241-251. doi:10.1001/archgenpsychiatry.2011.12

Mohammadi, M. R., Ghanizadeh, A., Mohammadi, M., & Mesgarpour, B. (2006). Prevalence of social phobia and its comorbidity with psychiatric disorders in Iran. *Depression and Anxiety, 23*, 405-411. doi:10.1002/da.20129

Mohammadi, M. R., Ghanizadeh, A., & Moini, R. (2007). Lifetime comorbidity of obsessive-compulsive disorder with psychiatric disorders in a community sample. *Depression and Anxiety, 24*, 602-607. doi:10.1002/da.20259

Pietrzak, R. H., Goldstein, R. B., Southwick, S. M., & Grant, B. F. (2012). Psychiatric Comorbidity of Full and Partial Posttraumatic Stress Disorder Among Older Adults in the United States: Results From Wave 2 of the National Epidemiologic Survey on Alcohol and Related Conditions. *American Journal of Geriatric Psychiatry, 20*, 380-390. doi:https://doi.org/10.1097/JGP.0b013e31820d92e7

Rihmer, Z., Szadoczky, E., Furedi, J., Kiss, K., & Papp, Z. (2001). Anxiety disorders comorbidity in bipolar I, bipolar II and unipolar major depression: results from a population-based study in Hungary. *Journal of Affective Disorders, 67*, 175-179.

Ruscio, A. M., Brown, T. A., Chiu, W. T., Sareen, J., Stein, M. B., & Kessler, R. C. (2008). Social fears and social phobia in the USA: results from the National Comorbidity Survey Replication. *Psychological Medicine, 38*, 15-28. doi:10.1017/S0033291707001699

Ruscio, A. M., Stein, D. J., Chiu, W. T., & Kessler, R. C. (2010). The epidemiology of obsessive-compulsive disorder in the National Comorbidity Survey Replication. *Molecular Psychiatry, 15*, 53-63. doi:10.1038/mp.2008.94

Schaffer, A., Cairney, J., Cheung, A., Veldhuizen, S., & Levitt, A. (2006). Community survey of bipolar disorder in Canada: lifetime prevalence and illness characteristics. *Canadian Journal of Psychiatry, 51*, 9-16. doi:10.1177/070674370605100104

Schneier, F. R., Johnson, J., Hornig, C. D., Liebowitz, M. R., & Weissman, M. M. (1992). Social Phobia: Comorbidity and Morbidity in an Epidemiologic Sample. *Archives of General Psychiatry, 49*, 282-288. doi:10.1001/archpsyc.1992.01820040034004

Subramaniam, M., Abdin, E., Vaingankar, J. A., & Chong, S. A. (2013). Prevalence, correlates, comorbidity and severity of bipolar disorder: Results from the Singapore Mental Health Study. *Journal of Affective Disorders, 146*, 189-196. doi:https://doi.org/10.1016/j.jad.2012.09.002

Zutshi, A., Reddy, Y. C., Thennarasu, K., & Chandrashekhar, C. R. (2006). Comorbidity of anxiety disorders in patients with remitted bipolar disorder. *European Archives of Psychiatry and Clinical Neuroscience, 256*, 428-436. doi:10.1007/s00406-006-0658-2

eReference 10. BIPOLAR and anxiety disorder: period prevalence comorbidity (14 studies)

Adam, Y., Meinlschmidt, G., Gloster, A. T., & Lieb, R. (2012). Obsessive-compulsive disorder in the community: 12-month prevalence, comorbidity and impairment. *Social Psychiatry and Psychiatric Epidemiology, 47*, 339-349. doi:10.1007/s00127-010-0337-5

Alvarenga, P. G., do Rosario, M. C., Cesar, R. C., Manfro, G. G., Moriyama, T. S., Bloch, M. H., . . . Miguel, E. C. (2016). Obsessive-compulsive symptoms are associated with psychiatric comorbidities, behavioral and clinical problems: a population-based study of Brazilian school children. *European Child and Adolescent Psychiatry, 25*, 175-182. doi:10.1007/s00787-015-0723-3

Angst, J., Gamma, A., Endrass, J., Hantouche, E., Goodwin, R., Ajdacic, V., . . . Rossler, W. (2005). Obsessive-compulsive syndromes and disorders: significance of comorbidity with bipolar and anxiety syndromes. *European Archives of Psychiatry and Clinical Neuroscience, 255*, 65-71. doi:10.1007/s00406-005-0576-8

Chou, K. L. (2009). Social anxiety disorder in older adults: evidence from the National Epidemiologic Survey on alcohol and related conditions. *Journal of Affective Disorders, 119*, 76-83. doi:10.1016/j.jad.2009.04.002

Fehm, L., Beesdo, K., Jacobi, F., & Fiedler, A. (2008). Social anxiety disorder above and below the diagnostic

threshold: prevalence, comorbidity and impairment in the general population. *Social Psychiatry and Psychiatric Epidemiology, 43*, 257-265. doi:10.1007/s00127-007-0299-4

Fineberg, N. A., Hengartner, M. P., Bergbaum, C., Gale, T., Rossler, W., & Angst, J. (2013). Lifetime comorbidity of obsessive-compulsive disorder and sub-threshold obsessive-compulsive symptomatology in the community: impact, prevalence, socio-demographic and clinical characteristics. *International Journal of Psychiatry in Clinical Practice, 17*, 188-196. doi:10.3109/13651501.2013.777745

Grant, B. F., Hasin, D. S., Blanco, C., Stinson, F. S., Chou, S. P., Goldstein, R. B., . . . Huang, B. (2005). The epidemiology of social anxiety disorder in the United States: results from the National Epidemiologic Survey on Alcohol and Related Conditions. *Journal of Clinical Epidemiology, 66*, 1351-1361. Retrieved from http://www.psychiatrist.com/jcp/article/pages/2005/v66n11/v66n1102.aspx

Grant, B. F., Hasin, D. S., Stinson, F. S., Dawson, D. A., June Ruan, W., Goldstein, R. B., . . . Huang, B. (2005). Prevalence, correlates, co-morbidity, and comparative disability of DSM-IV generalized anxiety disorder in the USA: results from the National Epidemiologic Survey on Alcohol and Related Conditions. *Psychological Medicine, 35*, 1747-1759. doi:10.1017/s0033291705006069

Grant, B. F., Stinson, F. S., Hasin, D. S., Dawson, D. A., Chou, S. P., Ruan, W. J., & Huang, B. (2005). Prevalence, correlates, and comorbidity of bipolar I disorder and axis I and II disorders: results from the National Epidemiologic Survey on Alcohol and Related Conditions. *Journal of Clinical Epidemiology, 66*, 1205-1215. Retrieved from http://www.psychiatrist.com/jcp/article/pages/2005/v66n10/v66n1001.aspx

McEvoy, P. M., Grove, R., & Slade, T. (2011). Epidemiology of anxiety disorders in the Australian general population: findings of the 2007 Australian National Survey of Mental Health and Wellbeing. *Australian and New Zealand Journal of Psychiatry, 45*, 957-967. doi:10.3109/00048674.2011.624083

Mitchell, P., Slade, T., & Andrews, G. (2004). Twelve-month prevalence and disability of DSM-IV bipolar disorder in an Australian general population survey. *International Journal of Neuropharmacology, 34*, 777-785. doi:10.1017/S1461145710000635

Ohayon, M. M., Shapiro, C. M., & Kennedy, S. H. (2000). Differentiating DSM-IV anxiety and depressive disorders in the general population: comorbidity and treatment consequences. *Canadian Journal of Psychiatry, 45*, 166-172. doi:10.1177/070674370004500207

Scott, K. M., McGee, M. A., Oakley Browne, M. A., & Wells, J. E. (2006). Mental disorder comorbidity in Te Rau Hinengaro: the New Zealand Mental Health Survey. *Australian and New Zealand Journal of Psychiatry, 40*, 875-881. doi:10.1080/j.1440-1614.2006.01906.x

Zutshi, A., Reddy, Y. C., Thennarasu, K., & Chandrashekhar, C. R. (2006). Comorbidity of anxiety disorders in patients with remitted bipolar disorder. *European Archives of Psychiatry and Clinical Neuroscience, 256*, 428-436. doi:10.1007/s00406-006-0658-2

Supplementary Figures (efigure)

efigure 1 Studies by country

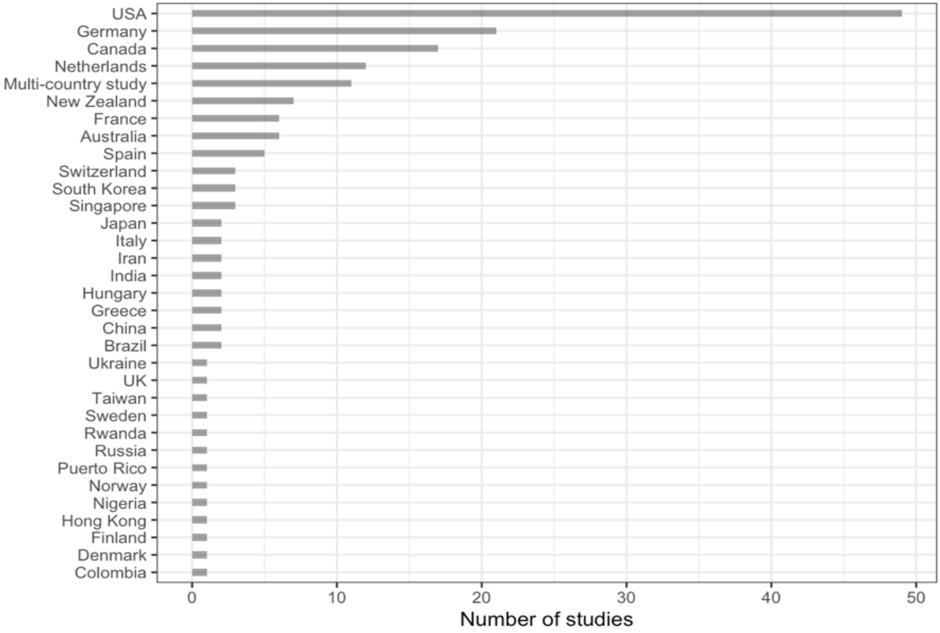

efigure 2 Forest plot of the random-effects meta-analysis of lifetime comorbidity between broadly-defined mood and broadly-defined anxiety disorders (unadjusted)

Study	Unadjusted Odds Ratio [95% CI]
Wichstrom et al, 2012 , Norway	5.10 [2.88, 9.03]
Newman et al, 1996 , New Zealand	5.71 [4.00, 8.15]
Mergl et al, 2007 , Germany	8.53 [4.99, 14.57]
Hecht et al, 1990 , Germany	11.71 [4.63, 29.57]
de Graaf et al, 2002 , Netherlands	12.23 [10.02, 14.92]
McEvoy et al, 2011 , Australia	13.40 [10.07, 17.84]
Fung et al, 2017 , Hong Kong	13.68 [7.32, 25.57]
He et al, 2009 , China	17.74 [14.38, 21.90]
Costello et al, 2003 , USA	27.21 [15.78, 46.93]
Pirkola et al, 2005 , Finland	54.28 [36.56, 80.60]
Heterogeneity (Q = 97.38, df = 9, p = 0.00; I^2 = 93.2%)	13.41 [8.61, 20.87]

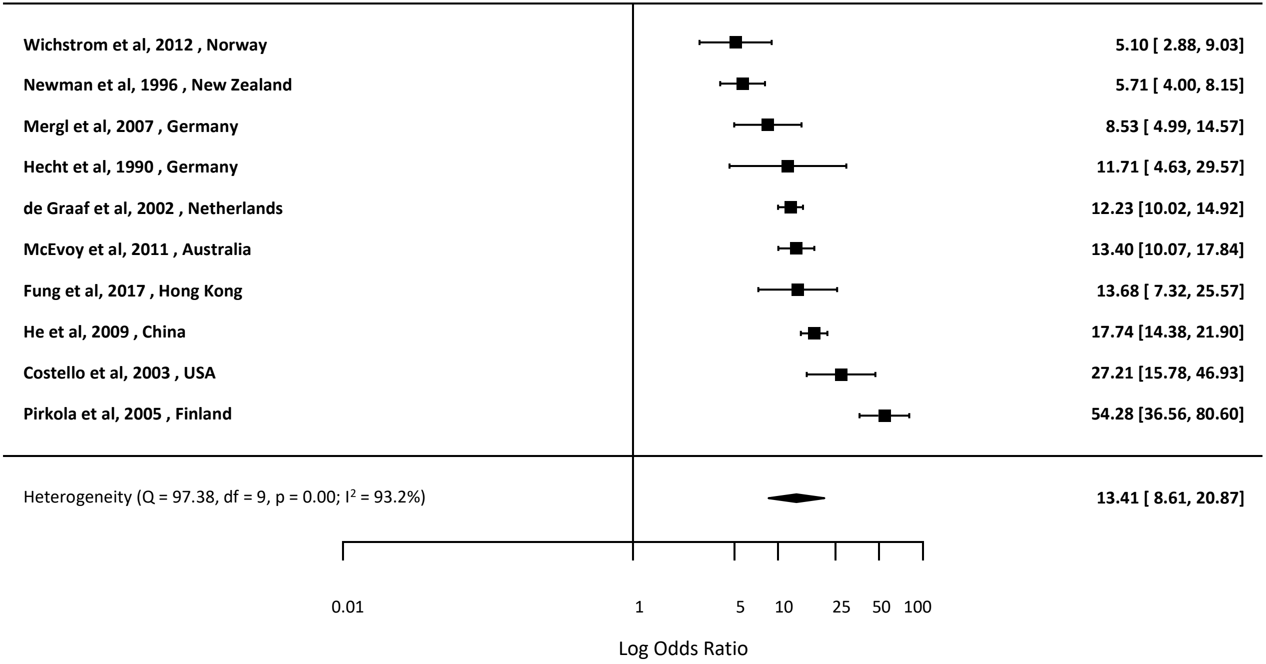

efigure 3 Funnel plot of the lifetime comorbidity between broadly-defined mood and broadly-defined anxiety disorders (unadjusted)

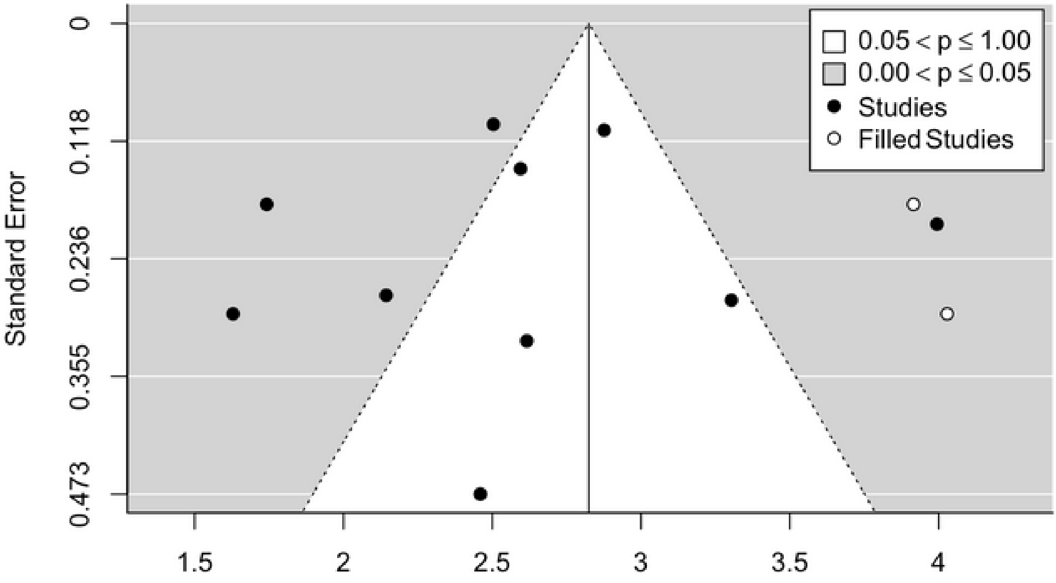

Log Odds Ratio

efigure 4 Forest plot of the random-effects meta-analysis of lifetime comorbidity between broadly-defined mood and broadly-defined anxiety disorders (adjusted)

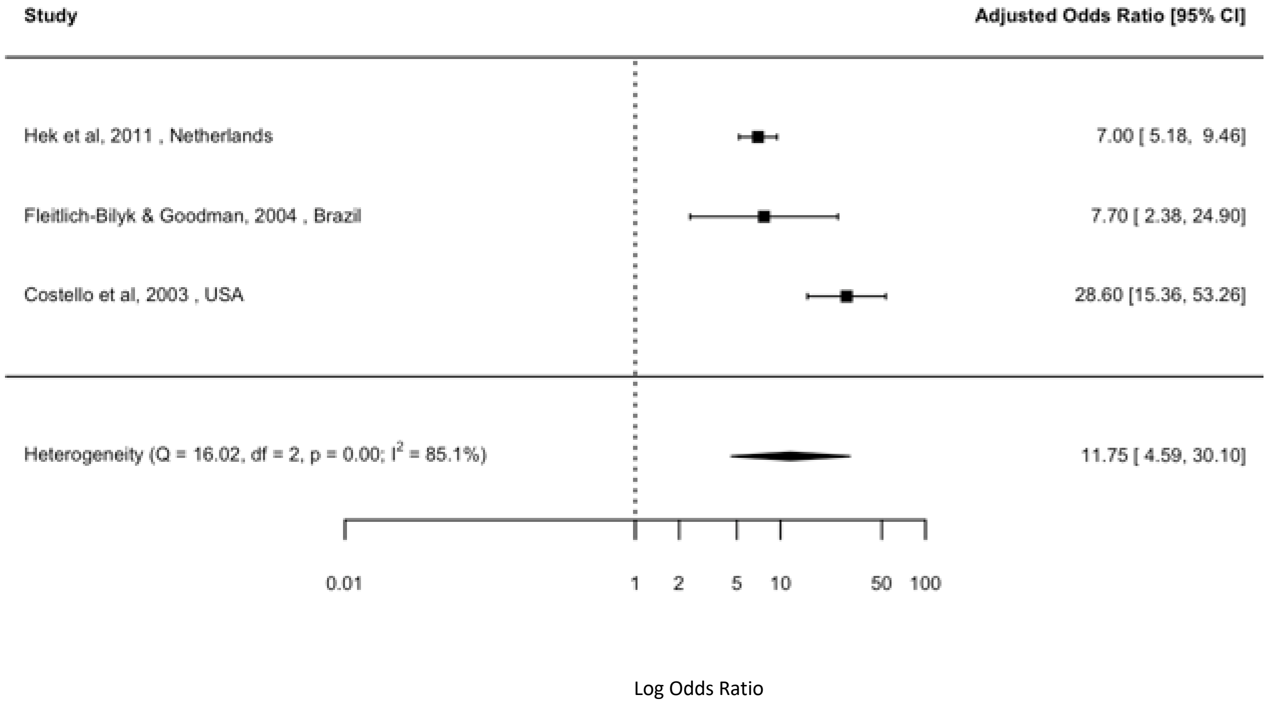

efigure 5 Forest plot of the random-effects meta-analysis of lifetime comorbidity between broadly-defined mood and social phobia (unadjusted)

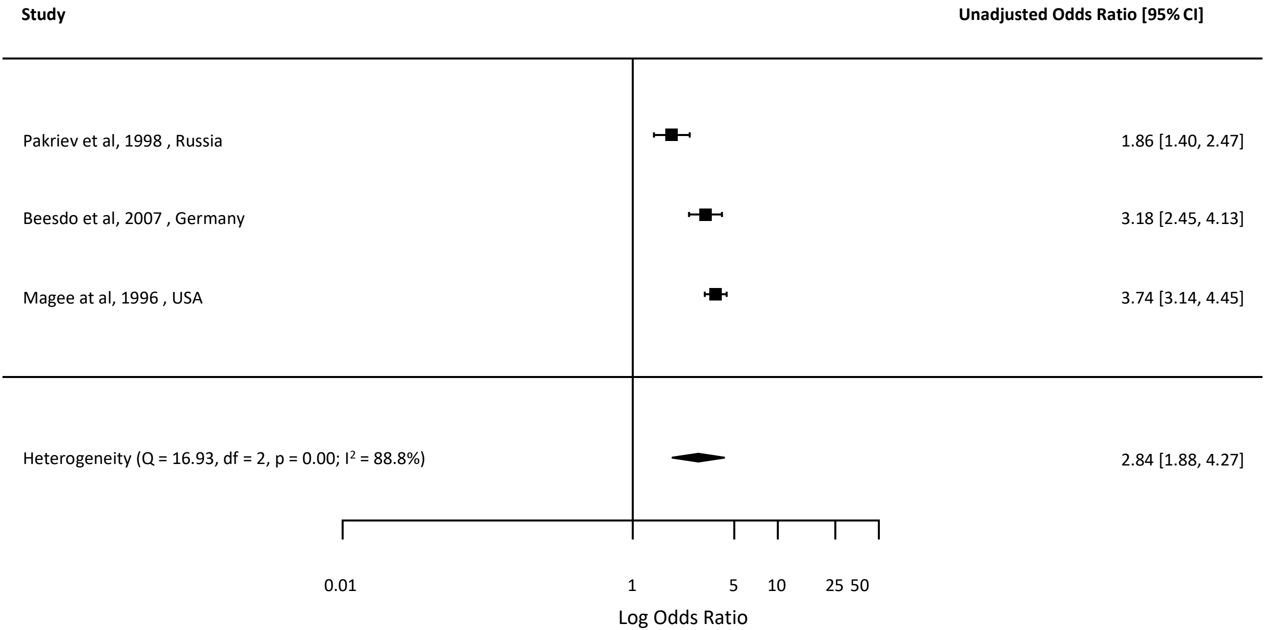

efigure 6 Forest plot of the random-effects meta-analysis of lifetime comorbidity between broadly-defined mood and specific phobia (unadjusted)

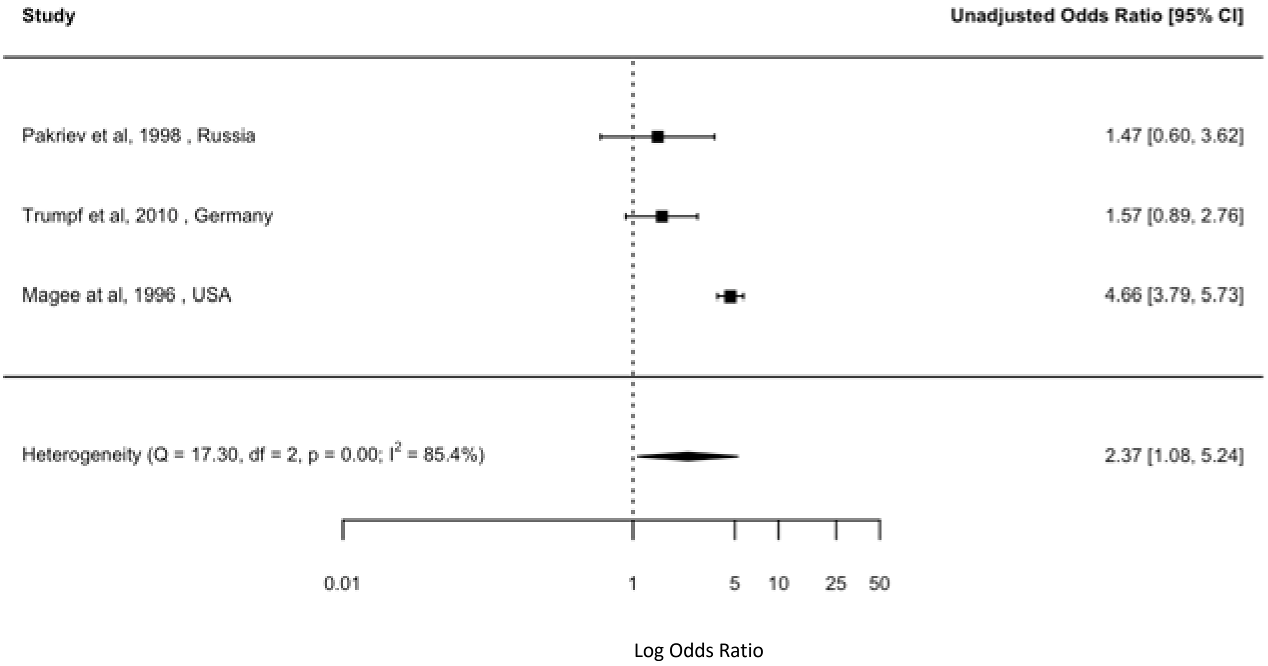

efigure 7 Forest plot of the random-effects meta-analysis of lifetime comorbidity between broadly-defined mood and broadly-defined anxiety disorder (unadjusted)

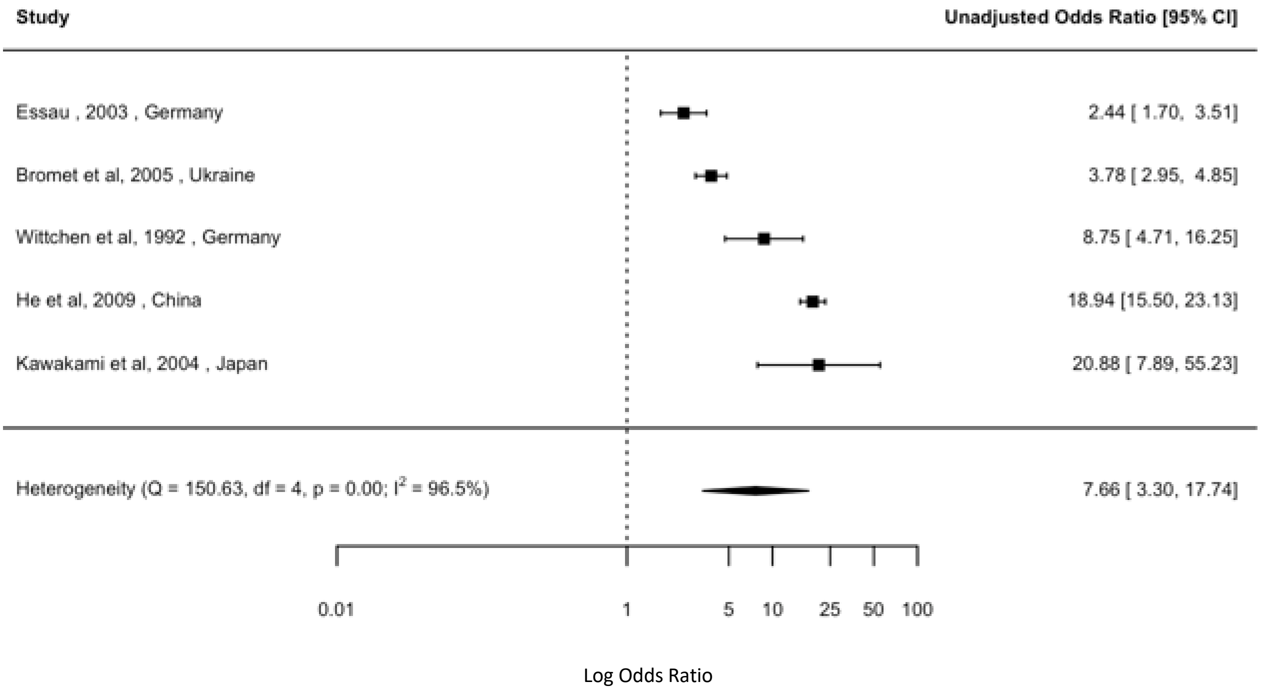

efigure 8 Forest plot of the random-effects meta-analysis of lifetime comorbidity between broadly-defined mood and social phobia (unadjusted)

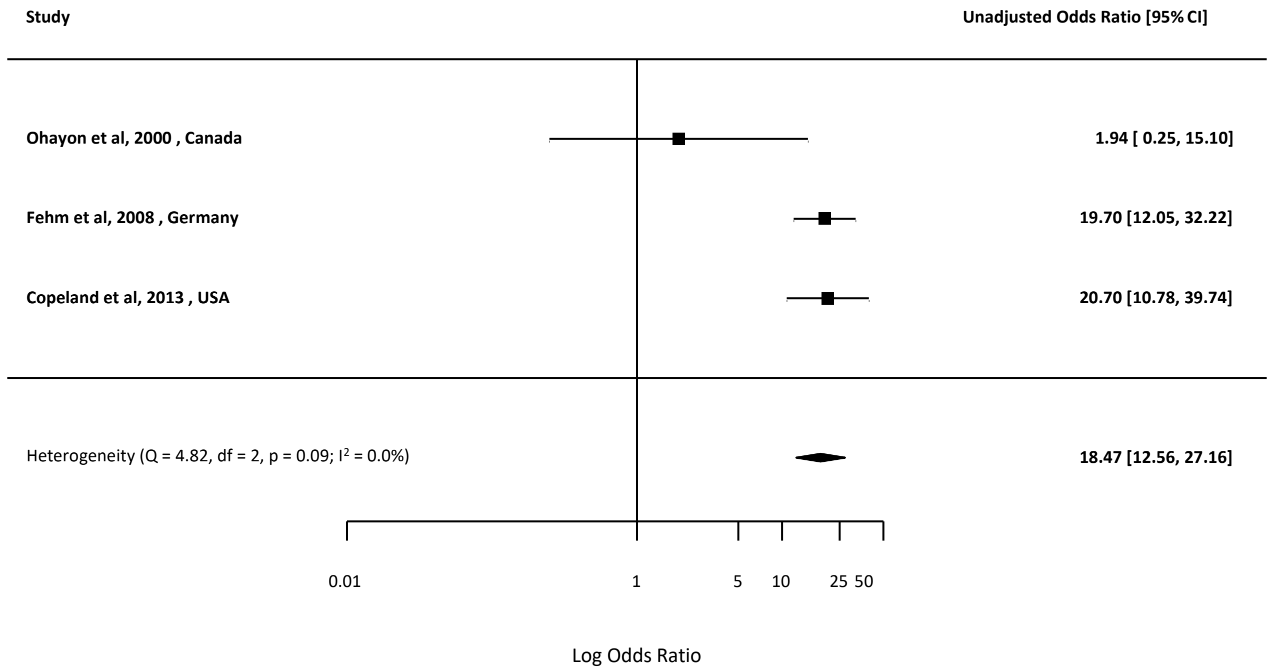

efigure 9 Forest plot of the random-effects meta-analysis of lifetime comorbidity between broadly-defined mood and social phobia (adjusted)

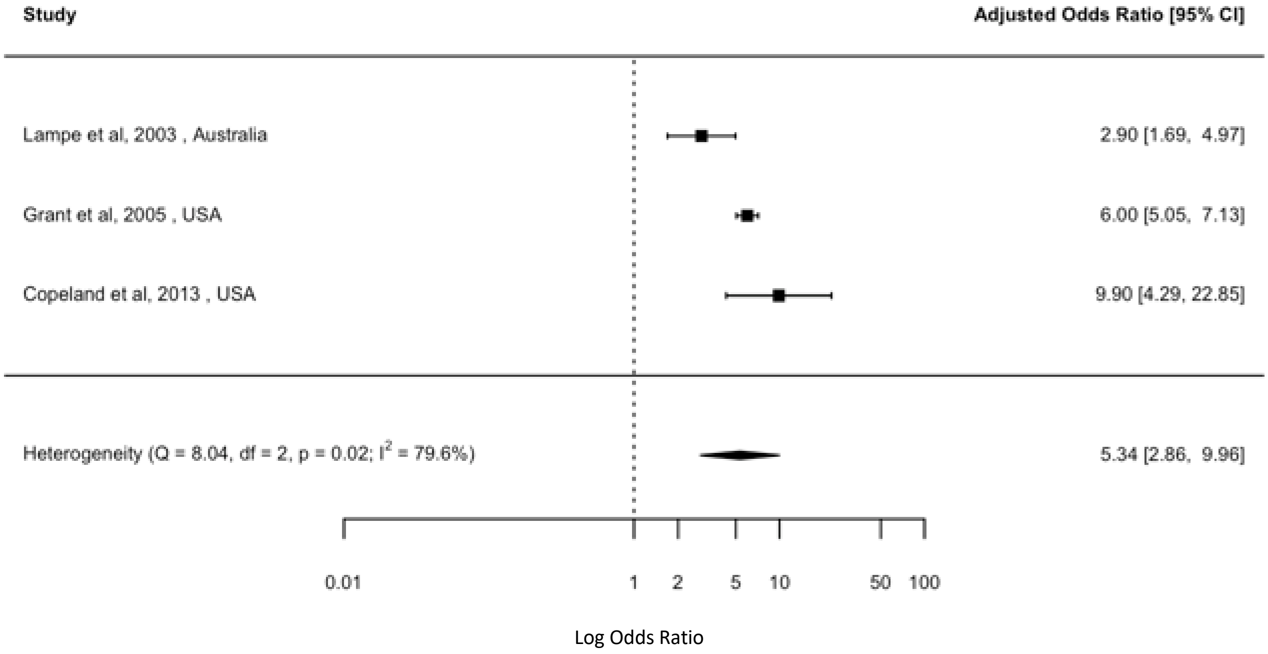

efigure 10 Forest plot of the random-effects meta-analysis of lifetime comorbidity between broadly-defined depressive disorders and agoraphobia (unadjusted)

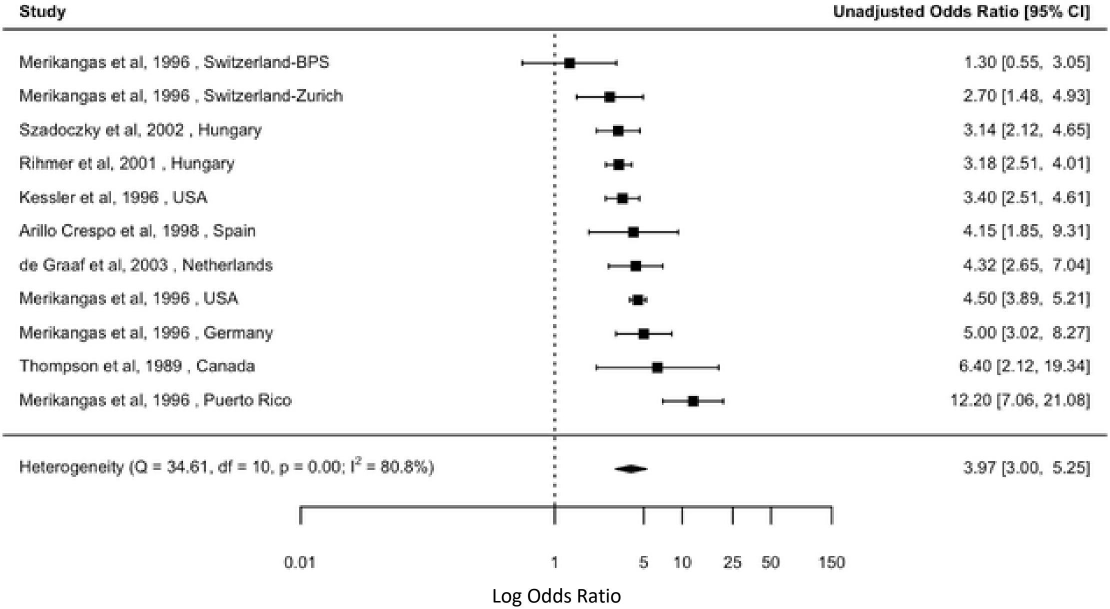

efigure 11 Funnel plot of the lifetime comorbidity between broadly-defined depressive disorders and agoraphobia (unadjusted)

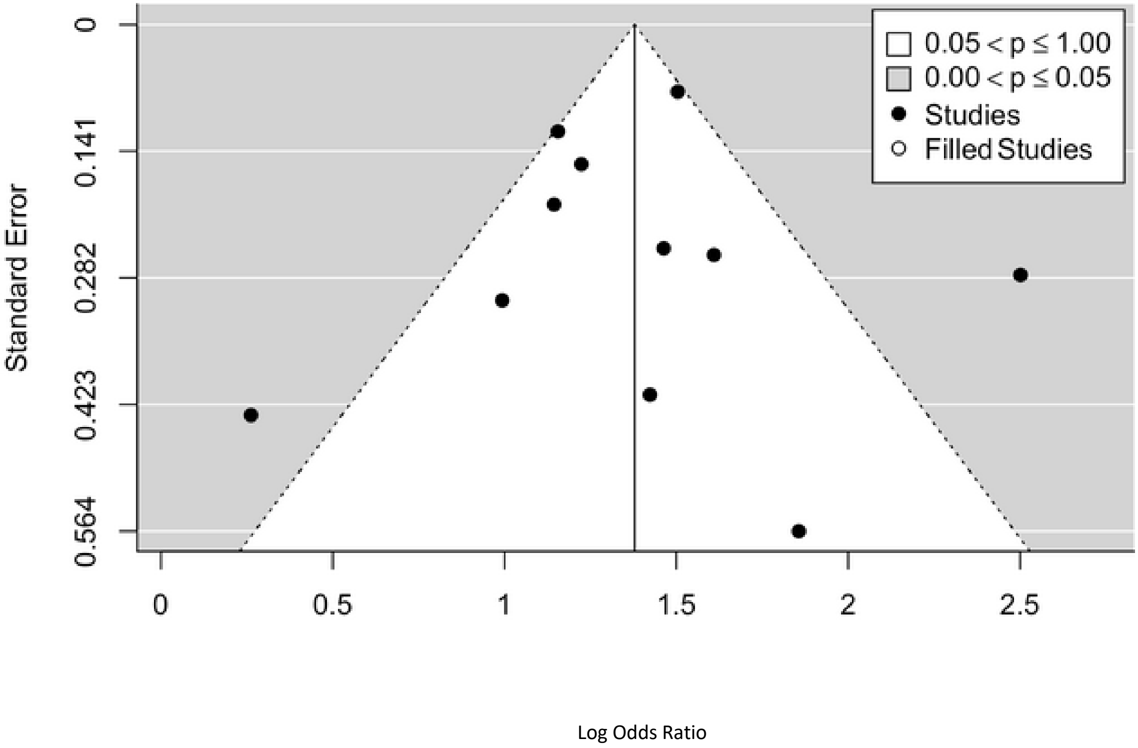

efigure 12 Forest plot of the random-effects meta-analysis of lifetime comorbidity between broadly-defined depressive disorders and obsessive compulsive disorder (unadjusted)

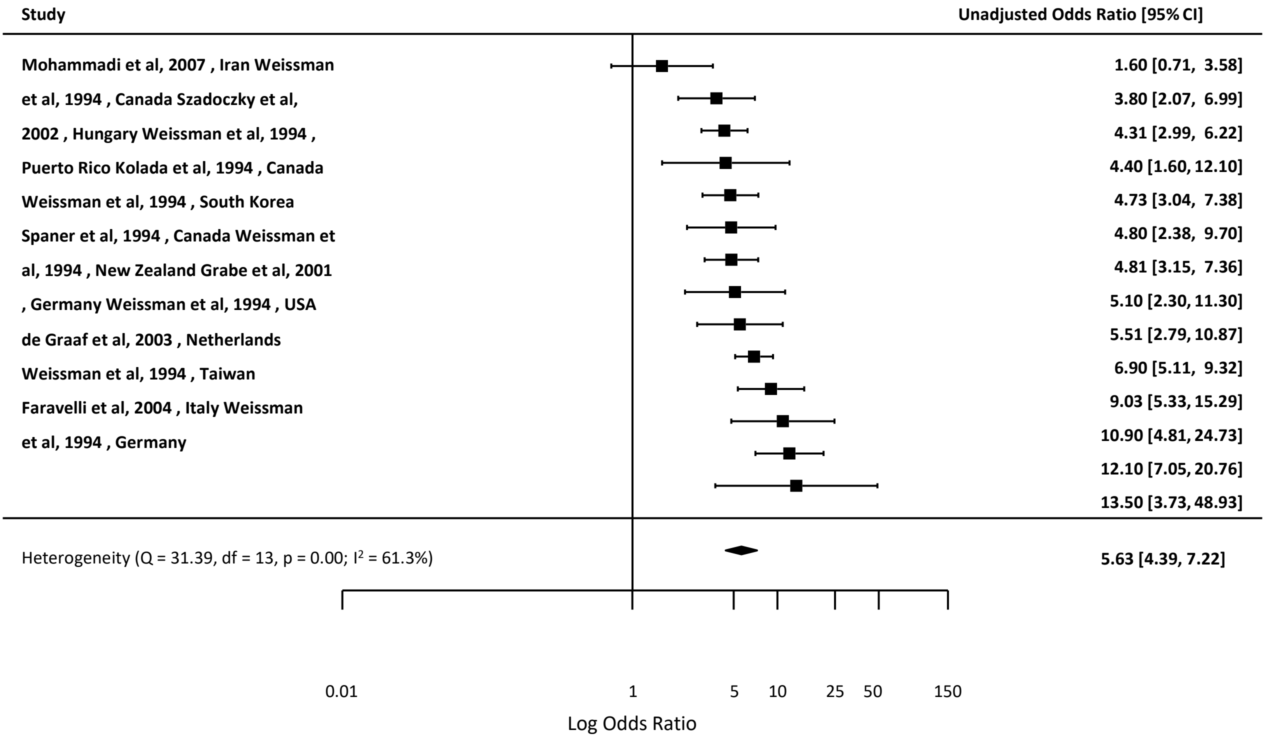

efigure 13 Funnel plot of the lifetime comorbidity between broadly-defined depressive disorders and obsessive compulsive disorder (unadjusted)

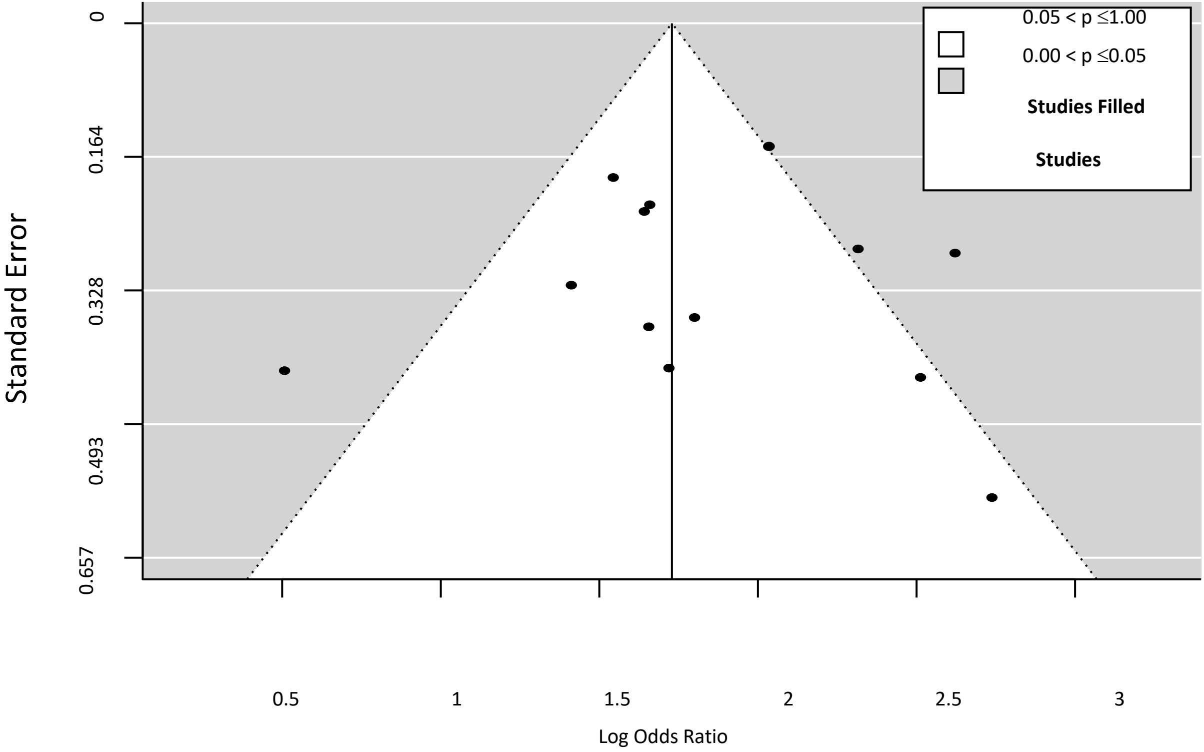

efigure 14 Forest plot of the random-effects meta-analysis of lifetime comorbidity between broadly-defined depressive disorders and obsessive compulsive disorder (adjusted)

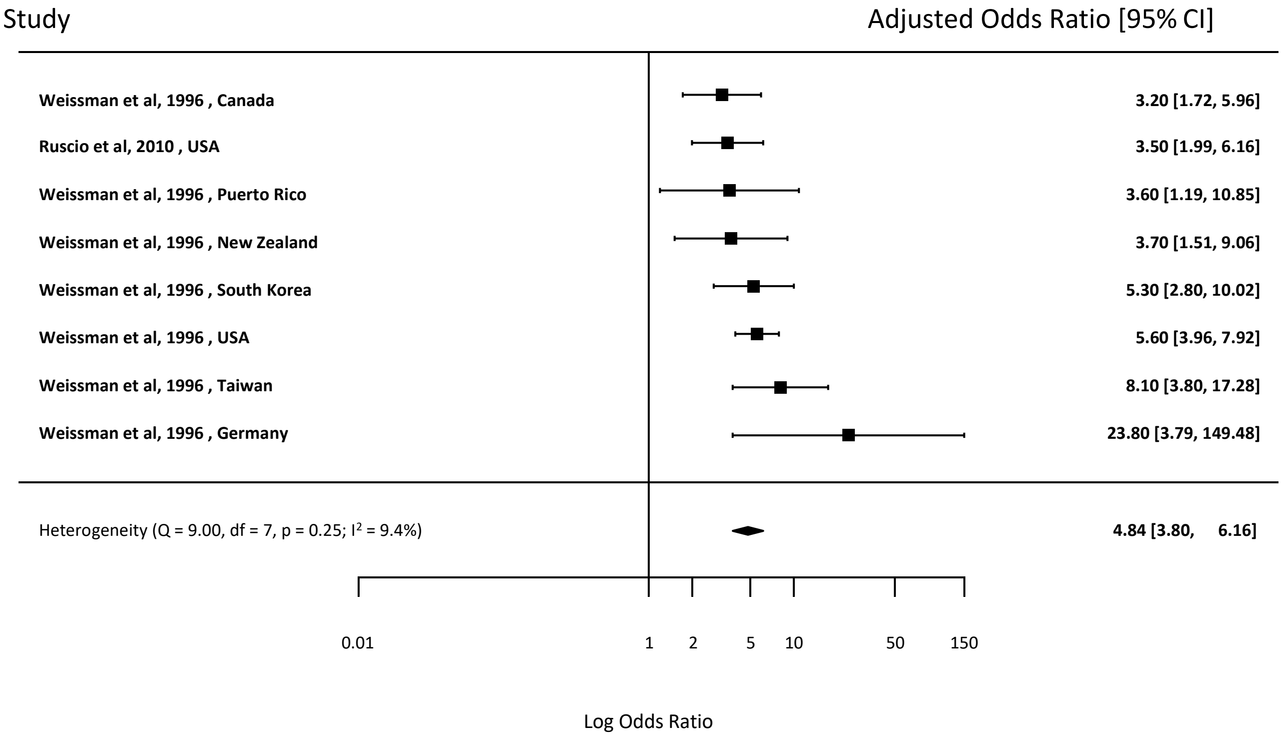

efigure 15 Forest plot of the random-effects meta-analysis of lifetime comorbidity between broadly-defined depressive disorders and generalized anxiety disorder (unadjusted)

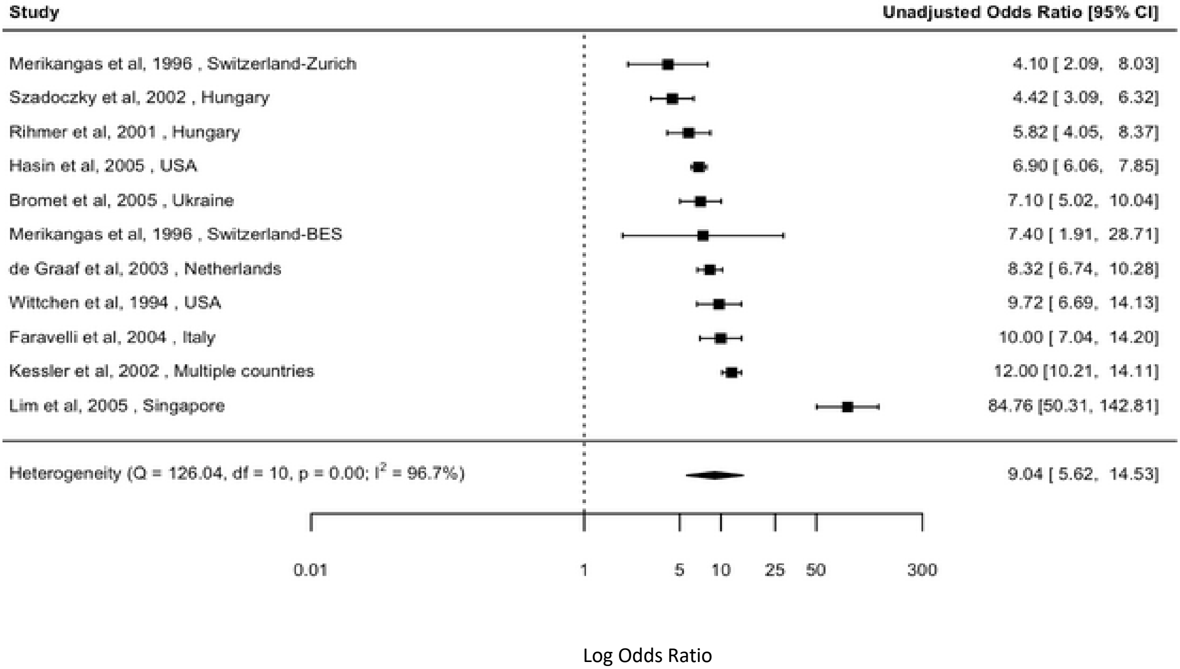

efigure 16 Funnel plot of the lifetime comorbidity between broadly-defined depressive disorders and generalized anxiety disorder (unadjusted)

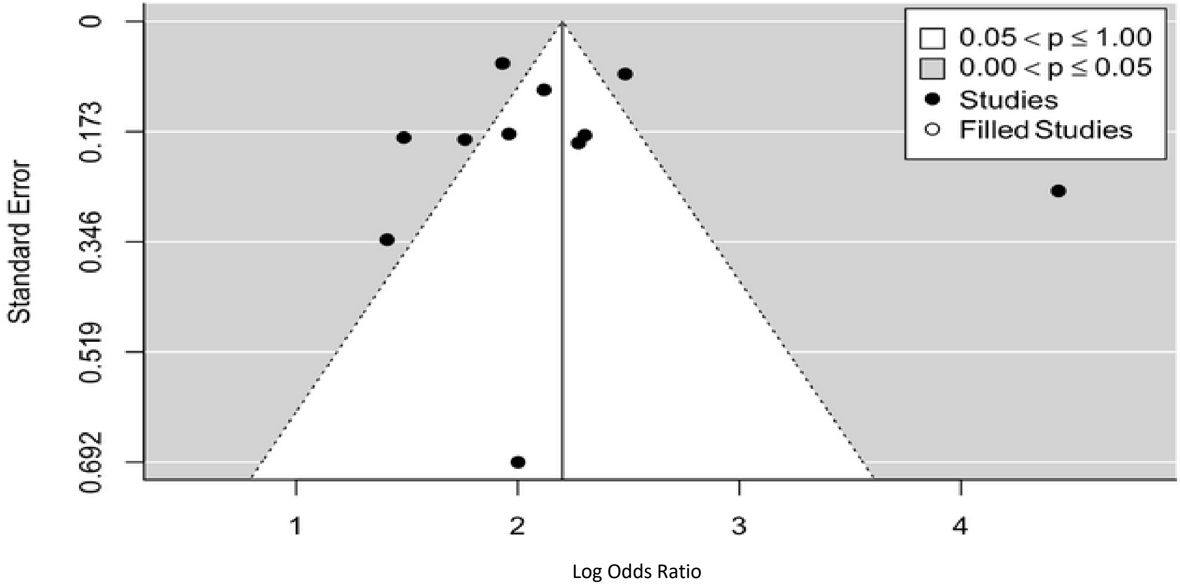

efigure 17 Forest plot of the random-effects meta-analysis of lifetime comorbidity between broadly-defined depressive disorders and generalized anxiety disorder (adjusted)

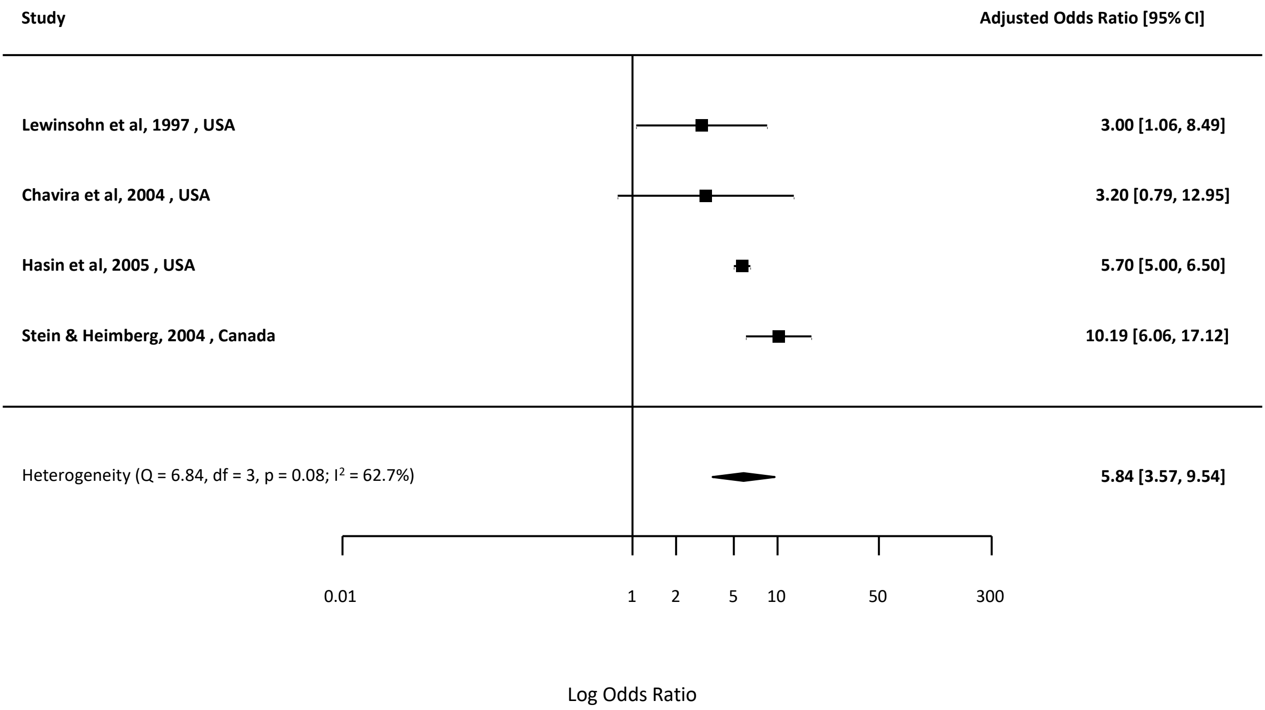

efigure 18 Forest plot of the random-effects meta-analysis of lifetime comorbidity between broadly-defined depressive disorders and panic disorder (unadjusted)

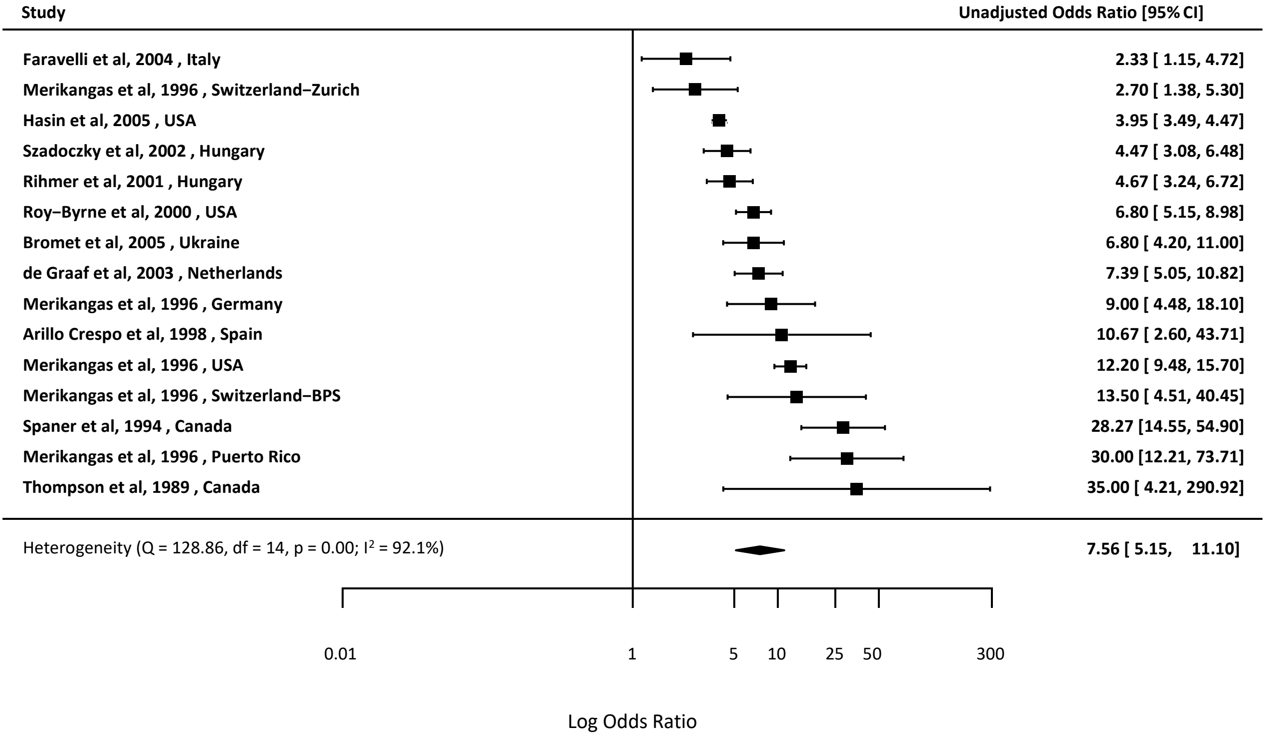

efigure 19 Funnel plot of the lifetime comorbidity between broadly-defined depressive disorders and panic disorder (unadjusted)

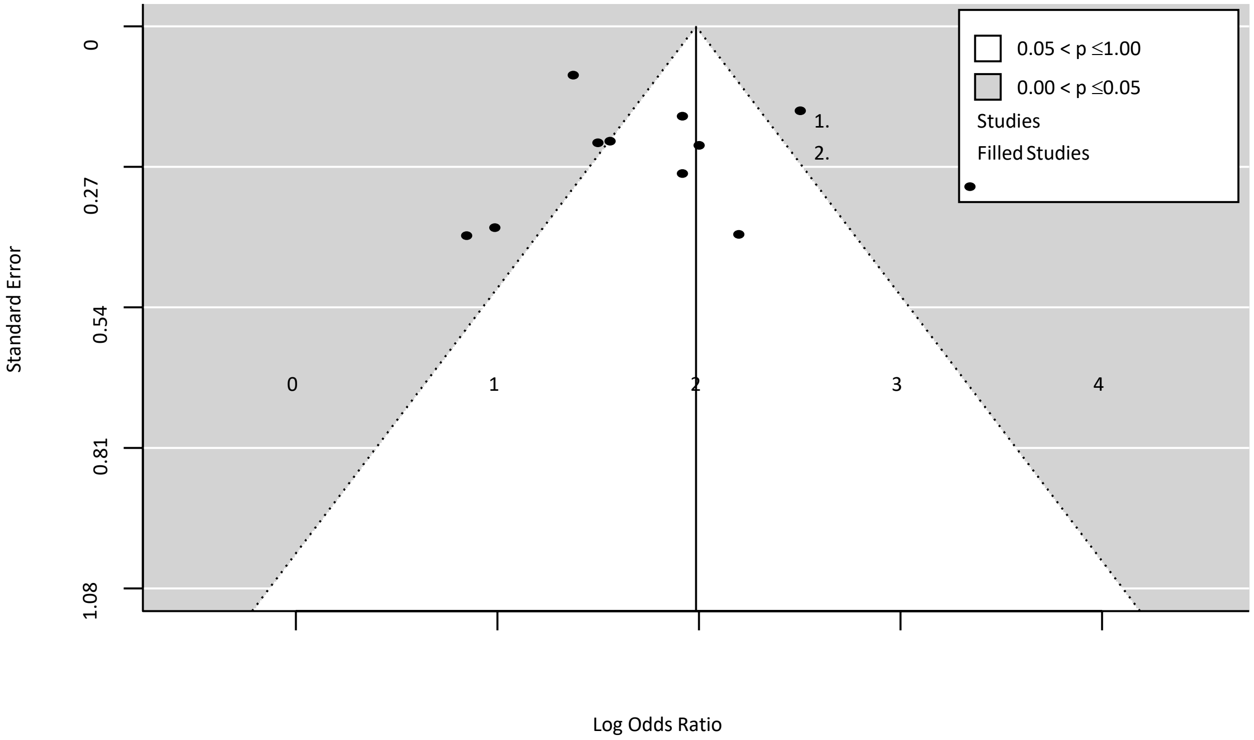

efigure 20 Forest plot of the random-effects meta-analysis of lifetime comorbidity between broadly-defined depressive disorders and panic disorder (adjusted)

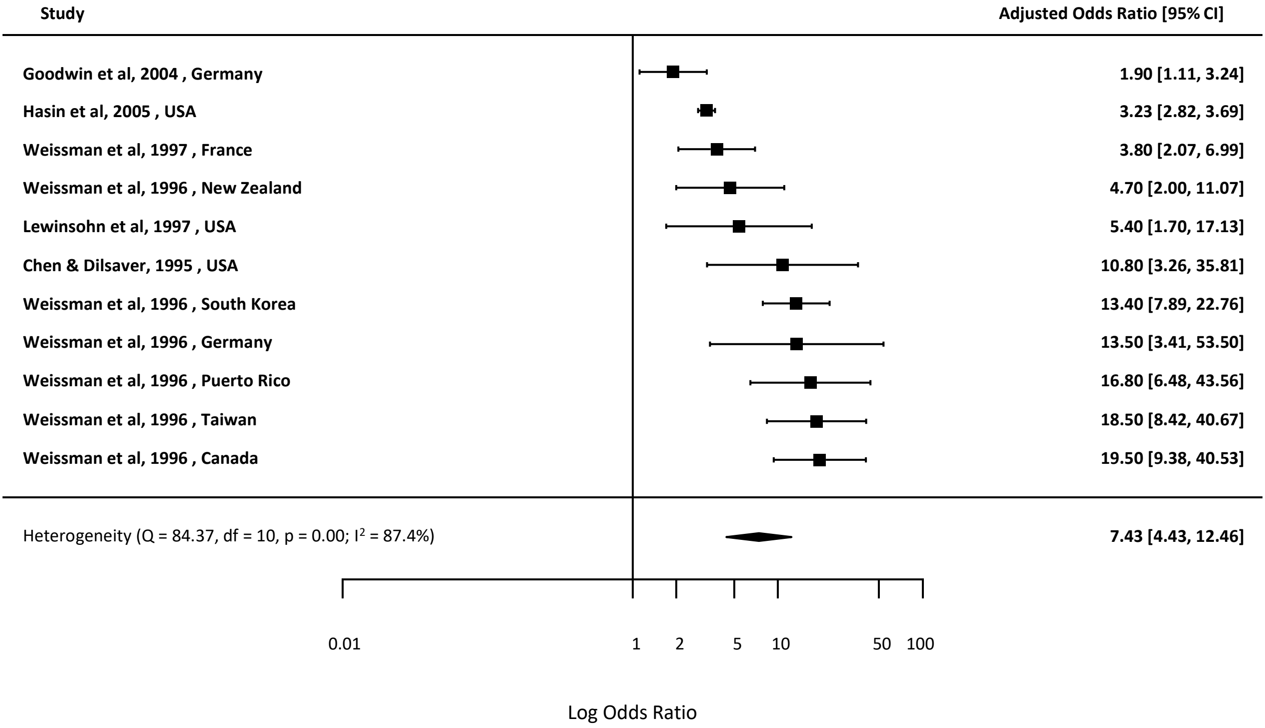

efigure 21 Funnel plot of the lifetime comorbidity between broadly-defined depressive disorders and panic disorder (adjusted)

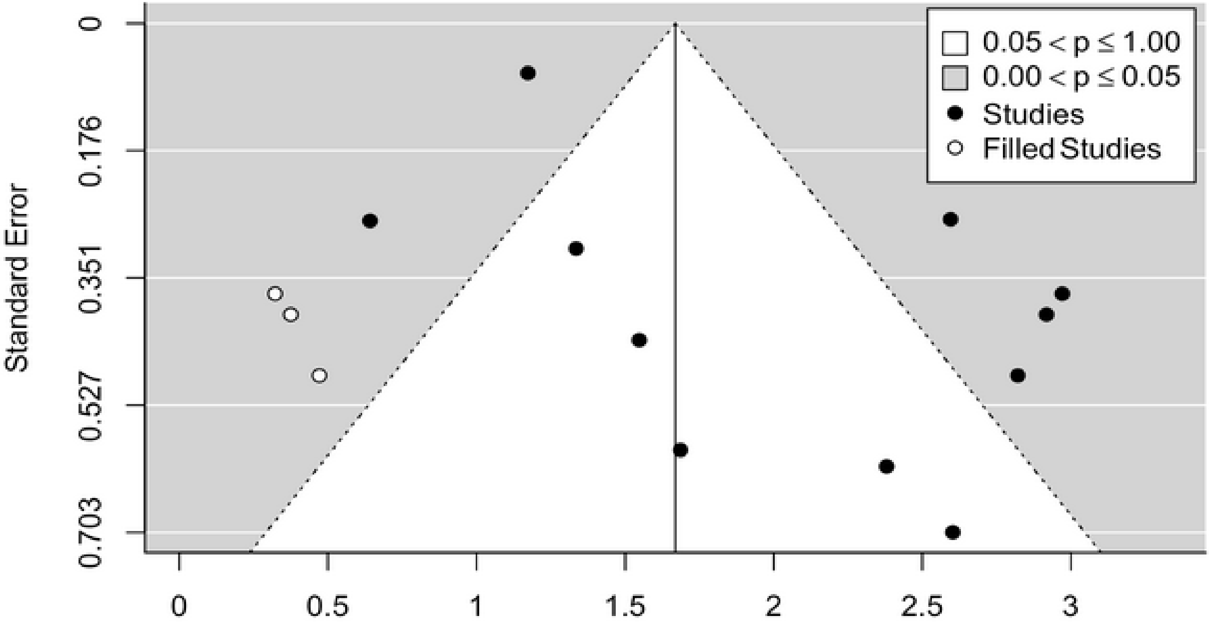

Log Odds Ratio

efigure 22 Forest plot of the random-effects meta-analysis of lifetime comorbidity between broadly-defined depressive disorders and post-traumatic stress disorder (unadjusted)

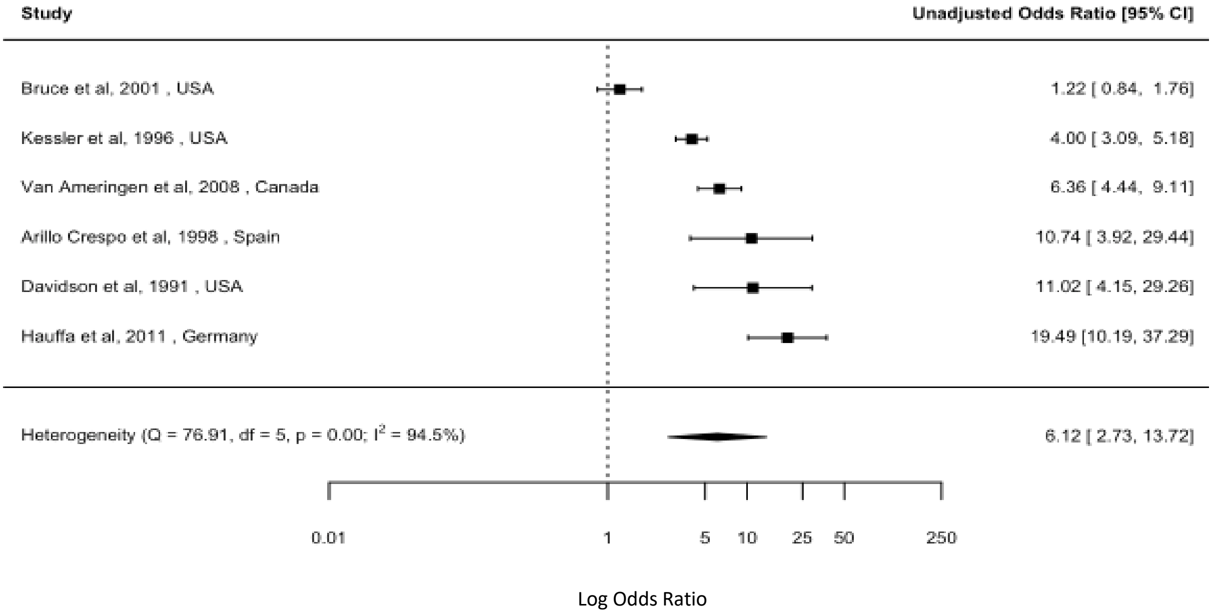

efigure 23 Forest plot of the random-effects meta-analysis of lifetime comorbidity between broadly-defined depressive disorders and social phobia (unadjusted)

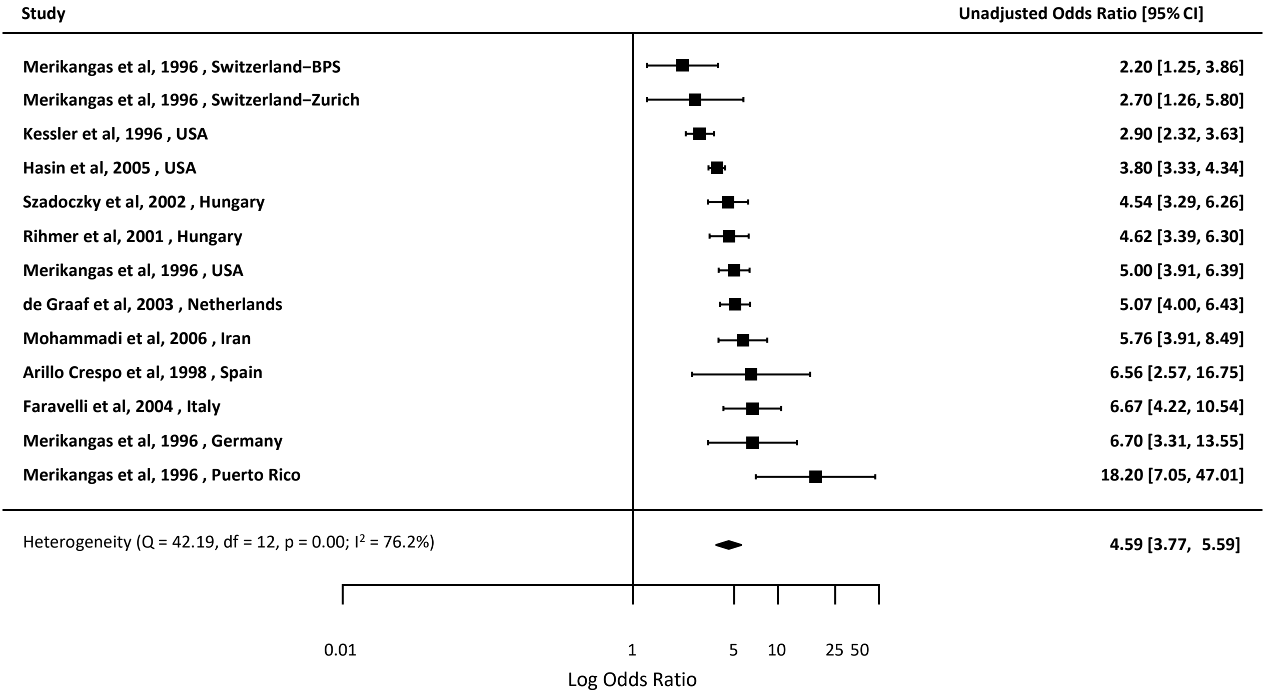

efigure 24 Funnel plot of the lifetime comorbidity between broadly-defined depressive disorders and social phobia (unadjusted)

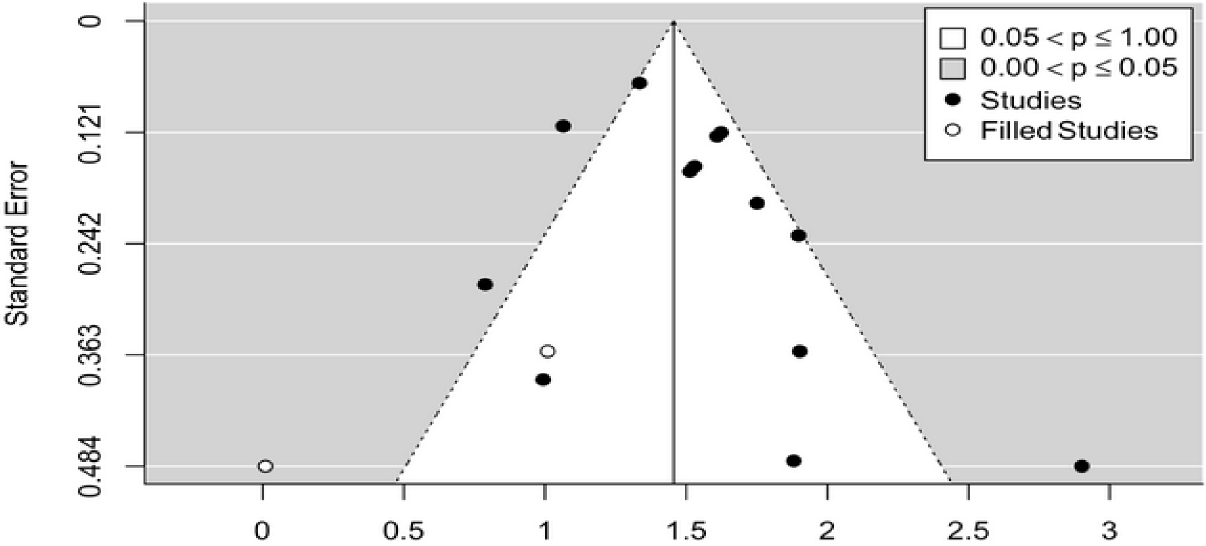

Log Odds Ratio

efigure 25 Forest plot of the random-effects meta-analysis of lifetime comorbidity between broadly-defined depressive disorders and social phobia (adjusted)

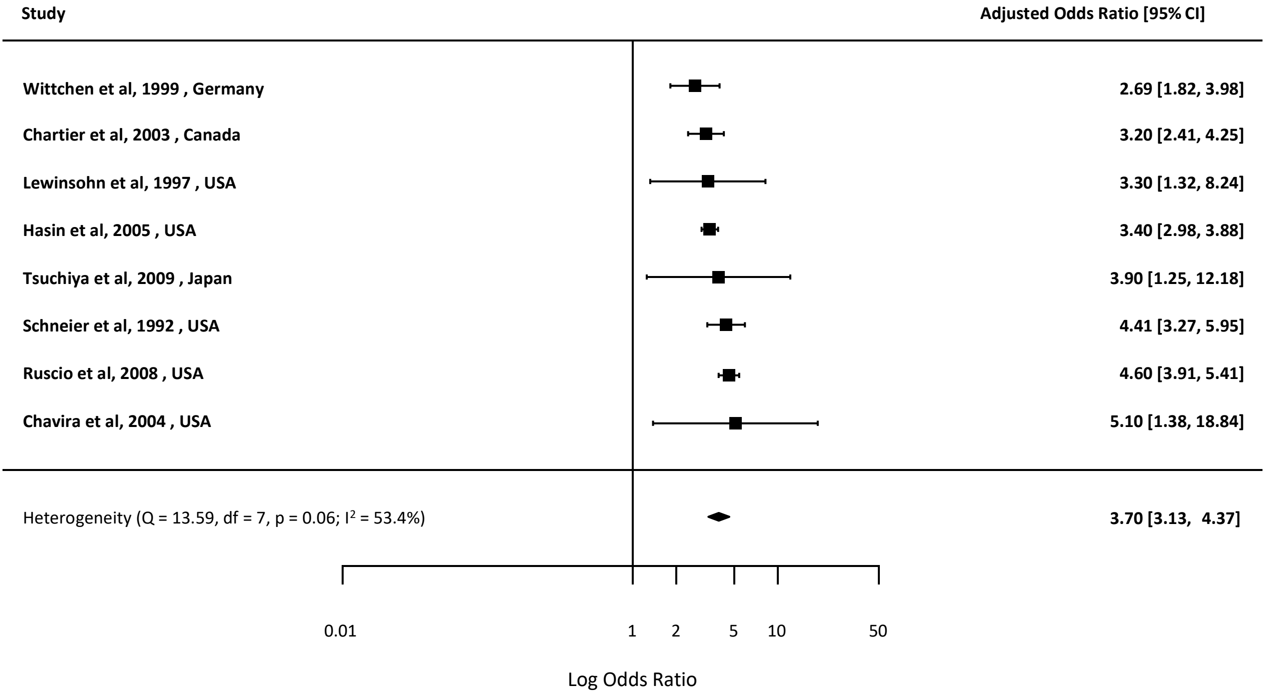

efigure 26 Forest plot of the random-effects meta-analysis of lifetime comorbidity between broadly-defined depressive disorders and specific phobia (unadjusted)

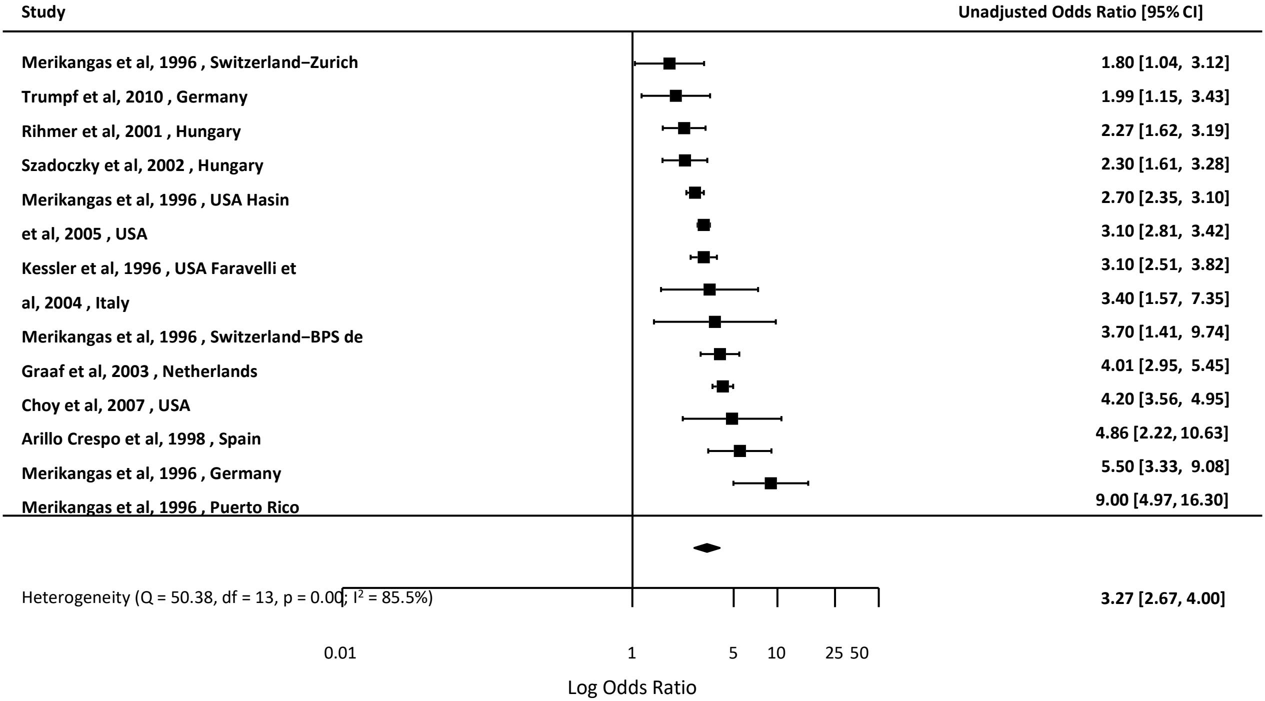

efigure 27 Funnel plot of the lifetime comorbidity between broadly-defined depressive disorders and specific phobia (unadjusted)

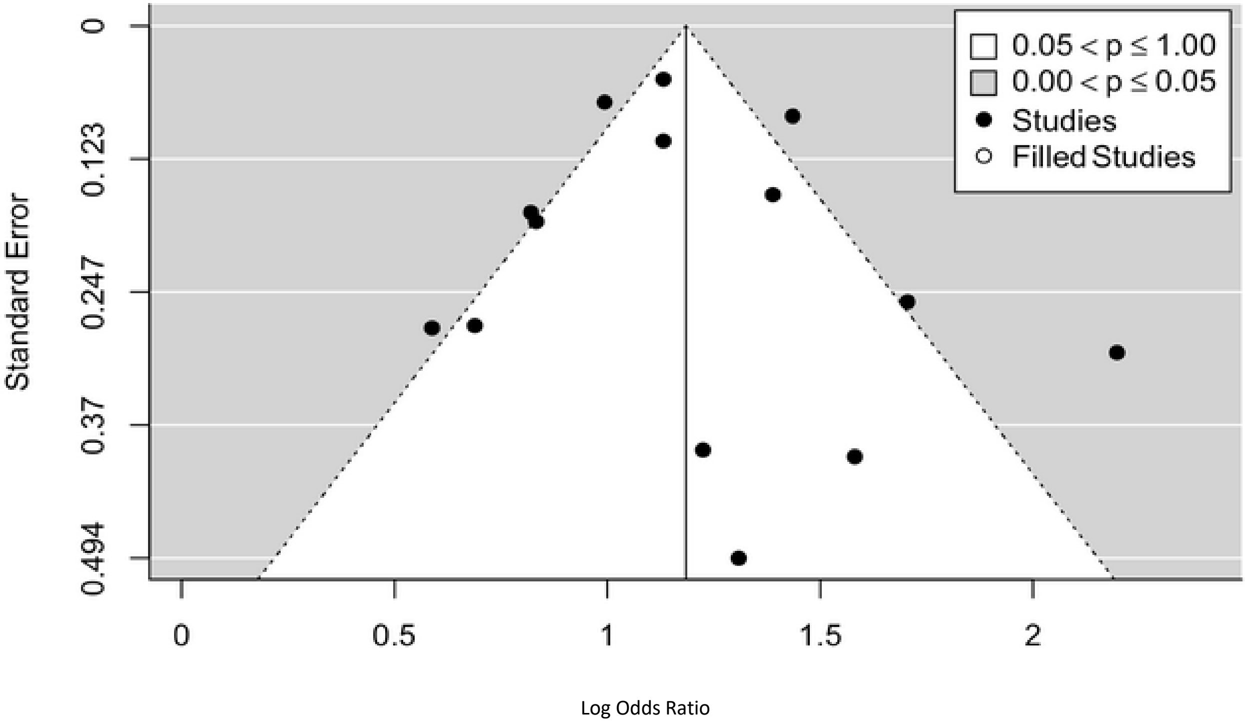

efigure 28 Forest plot of the random-effects meta-analysis of lifetime comorbidity between broadly-defined depressive disorders and specific phobia (adjusted)

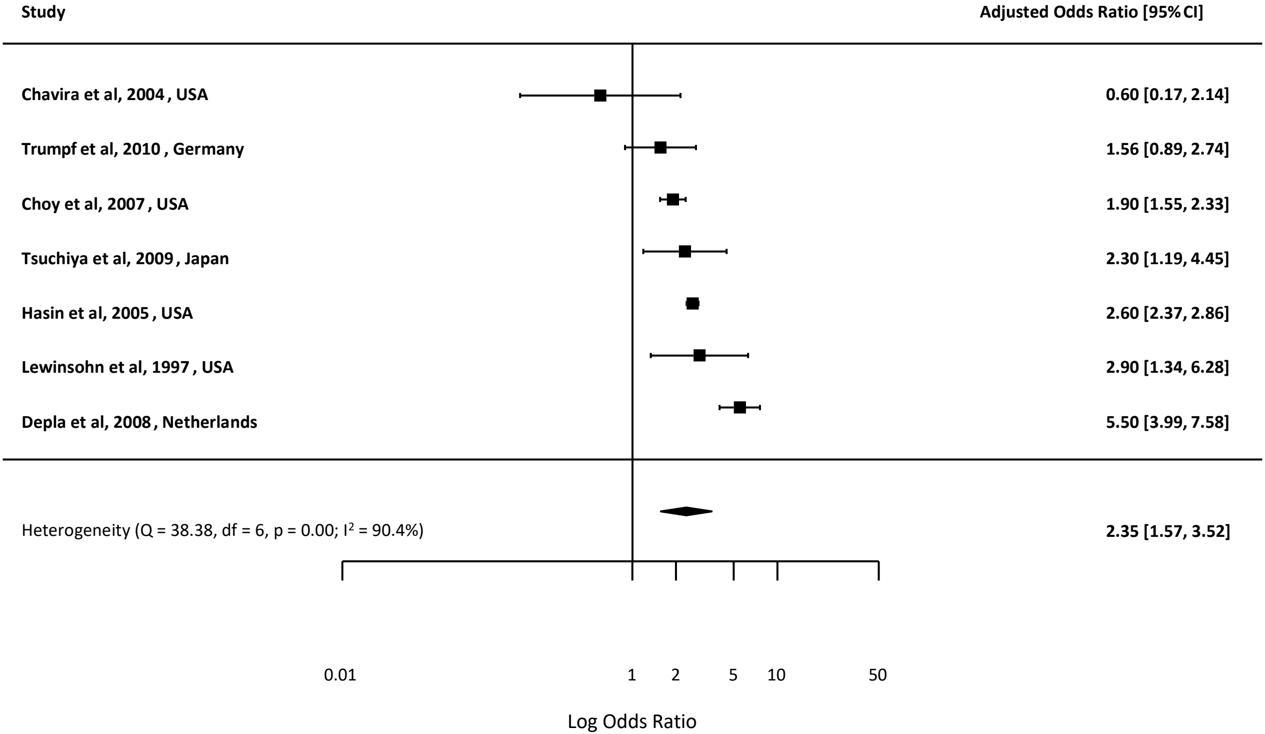

efigure 29 Forest plot of the random-effects meta-analysis of lifetime comorbidity between broadly-defined depressive disorders and anxiety disorder (unadjusted)

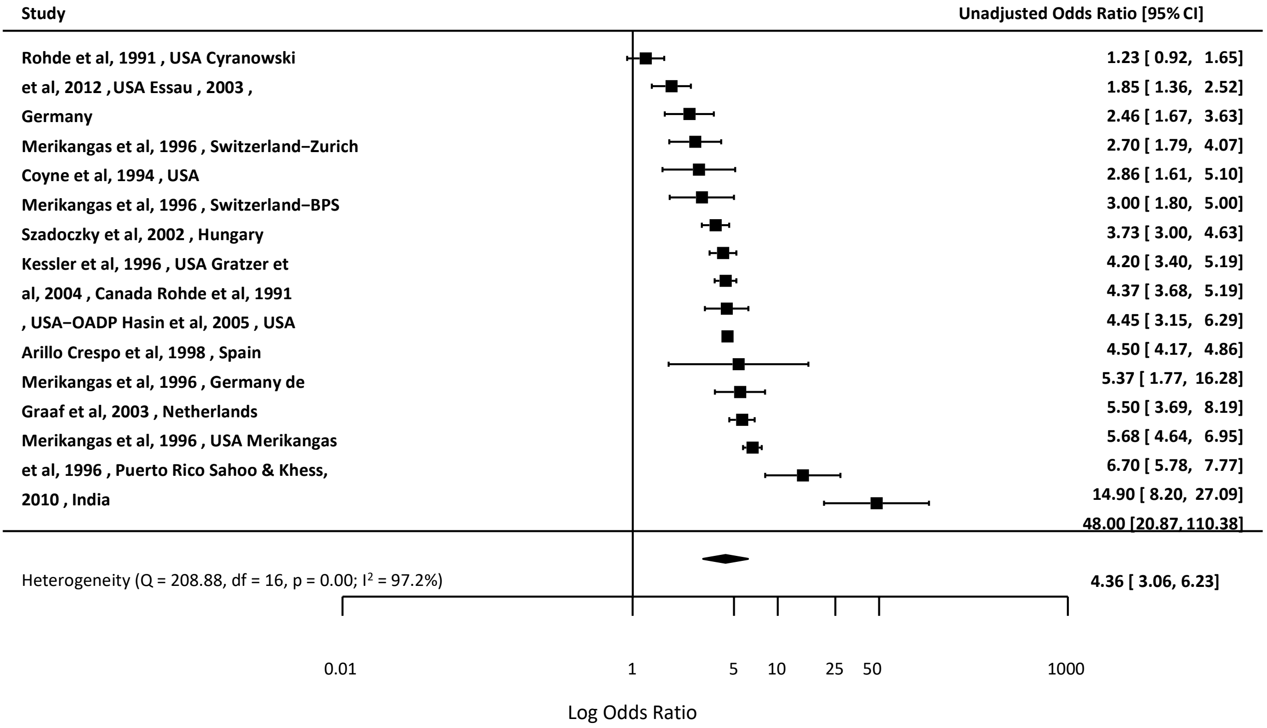

efigure 30 Funnel plot of the lifetime comorbidity between broadly-defined depressive disorders and anxiety disorder (unadjusted)

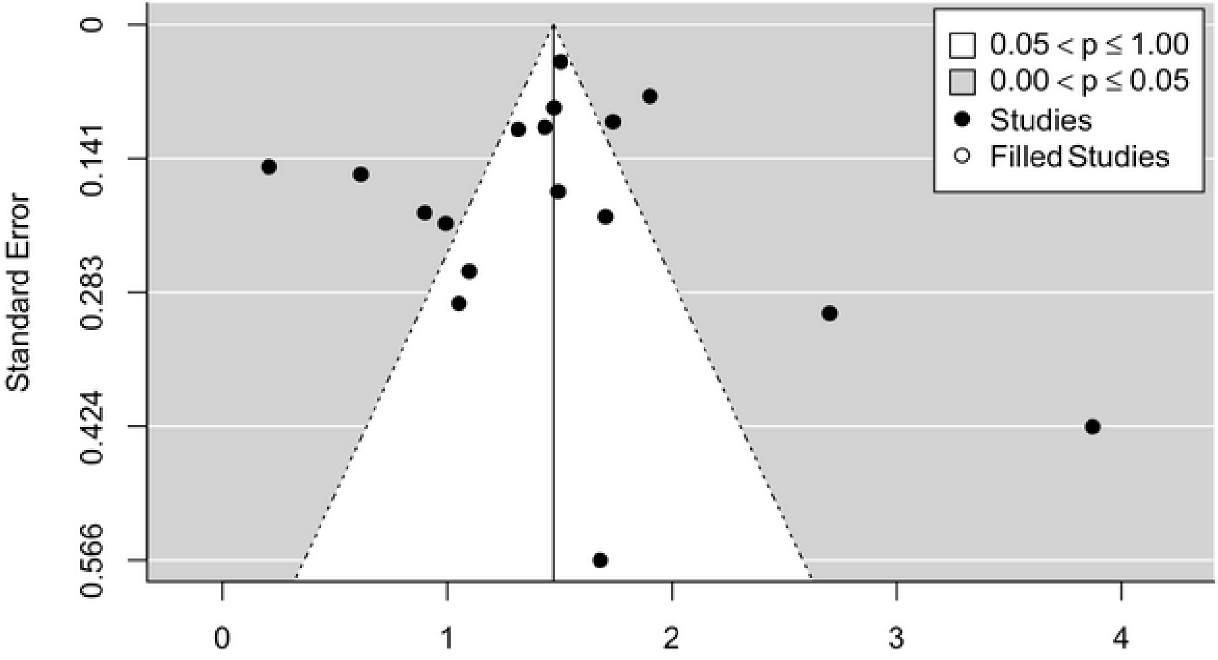

Log Odds Ratio

efigure 31 Forrest plot of the lifetime comorbidity between broadly-defined depressive disorders and anxiety disorder (adjusted)

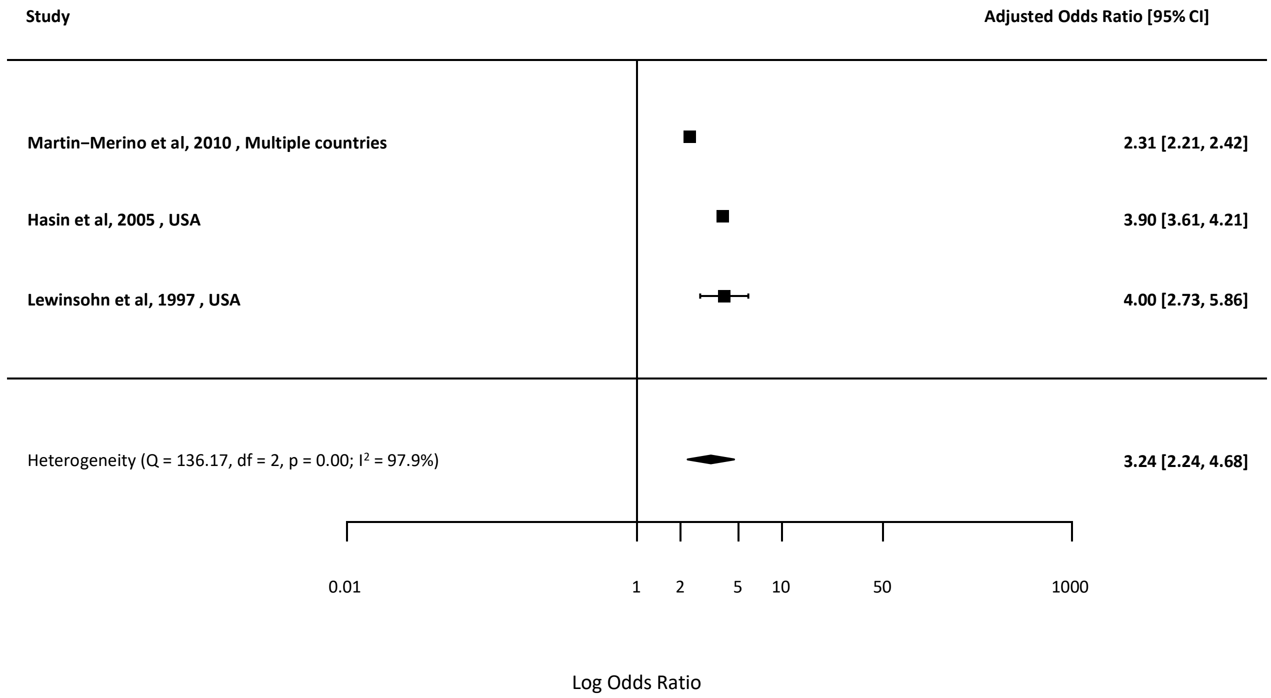

efigure 32 Forest plot of the random-effects meta-analysis of period prevalence comorbidity between broadly-defined depressive disorders and agoraphobia (unadjusted)

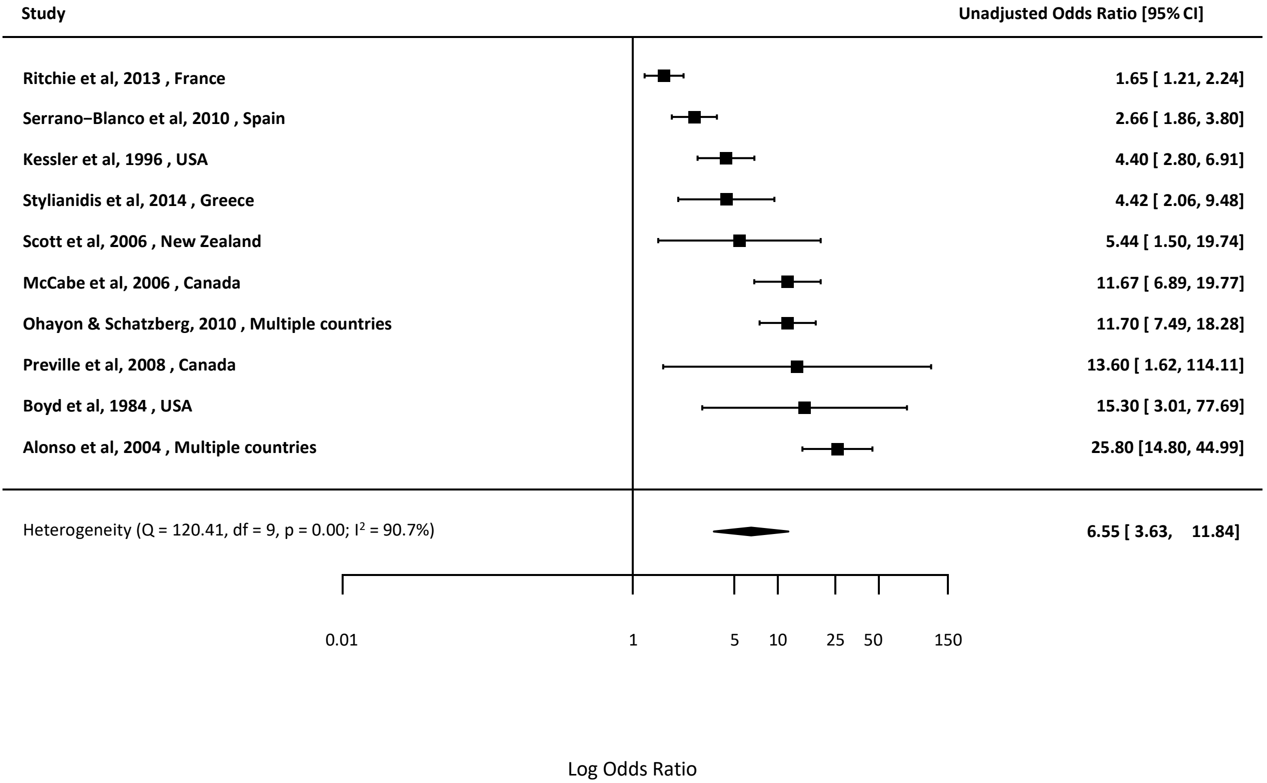

efigure 33 Funnel plot of the period prevalence comorbidity between broadly-defined depressive disorders and agoraphobia (unadjusted)

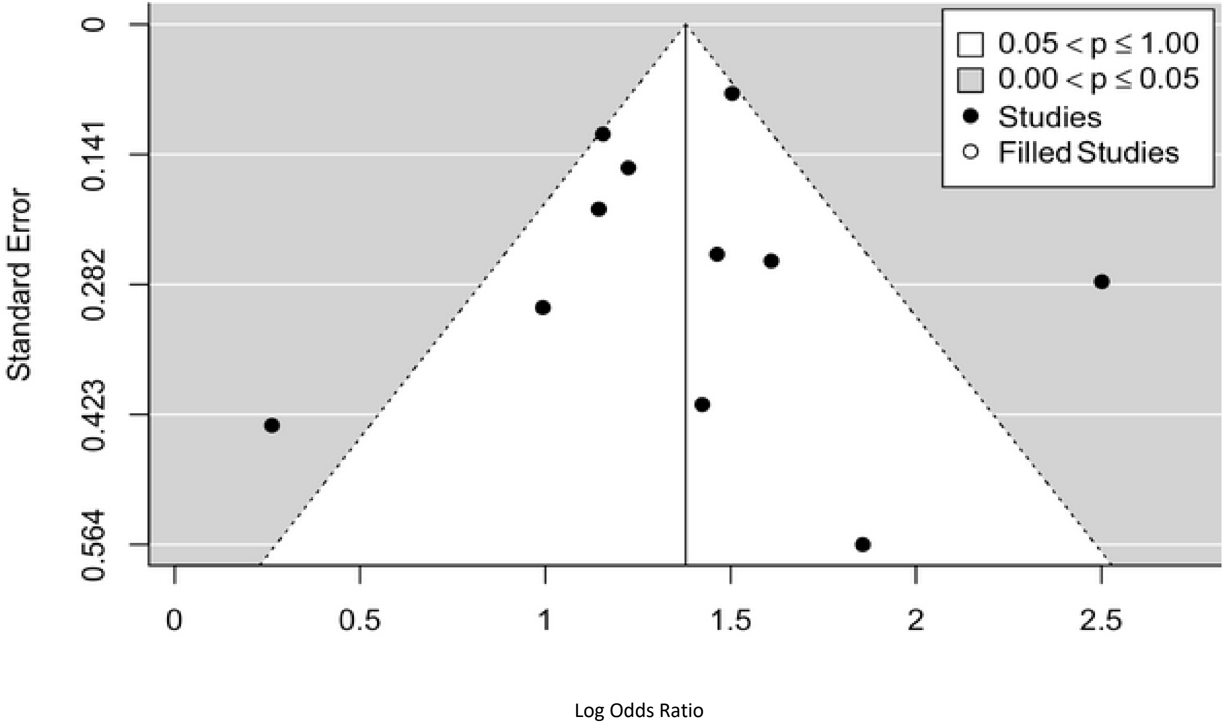

efigure 34 Forest plot of the random-effects meta-analysis of period prevalence comorbidity between broadly-defined depressive disorders and agoraphobia (adjusted)

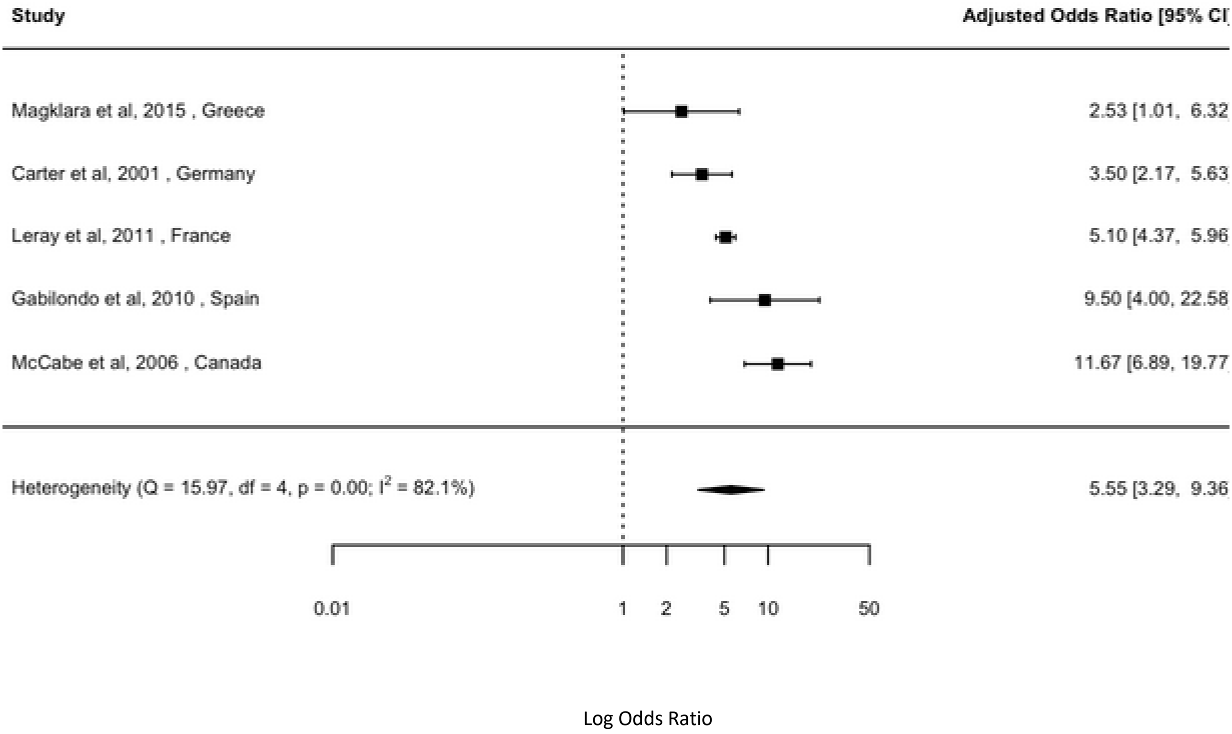

efigure 35 Forest plot of the random-effects meta-analysis of period prevalence comorbidity between broadly-defined depressive disorders and obsessive compulsive disorder (unadjusted)

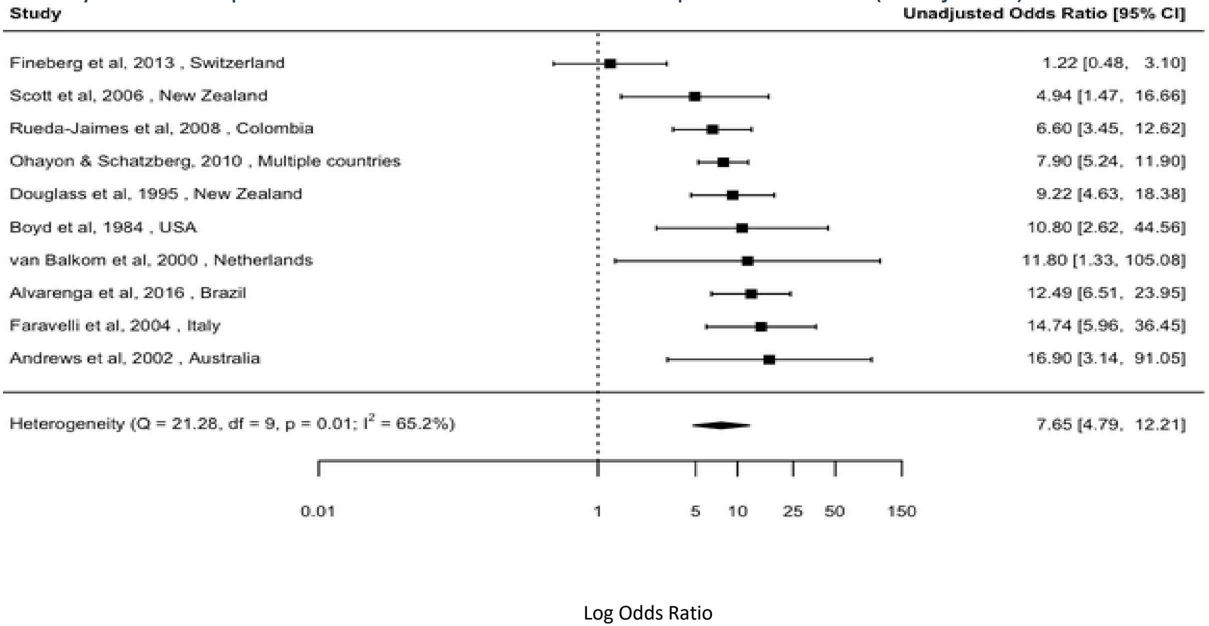

efigure 36 Funnel plot of the period prevalence comorbidity between broadly-defined depressive disorders and obsessive compulsive disorder (unadjusted)

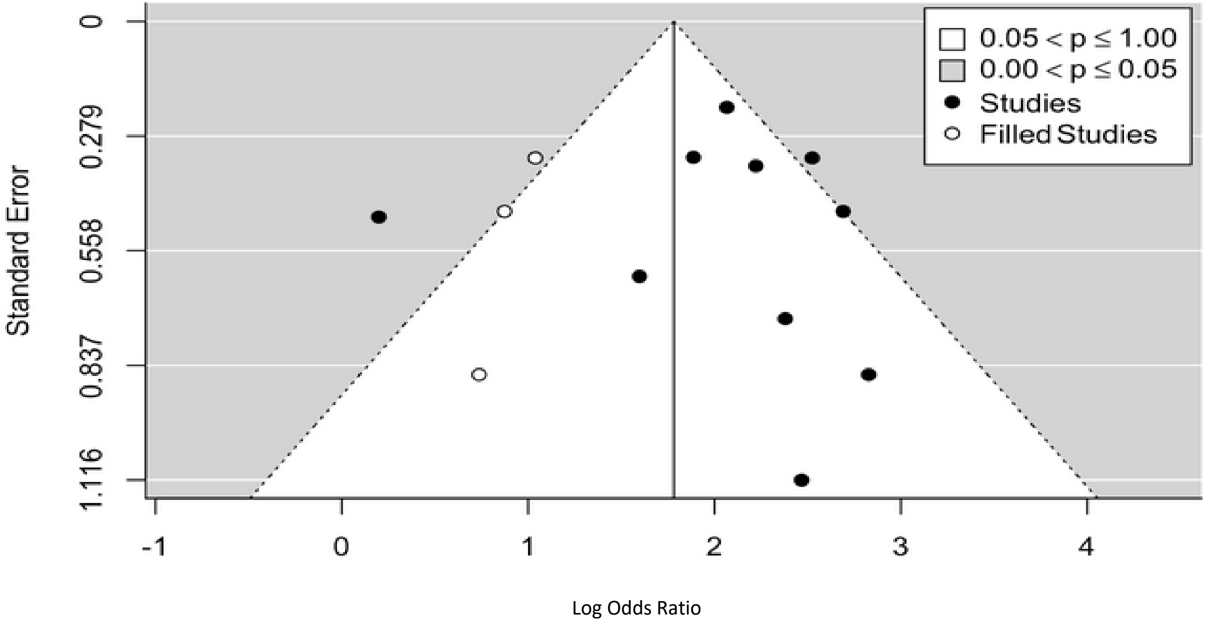

efigure 37 Forest plot of the random-effects meta-analysis of period prevalence comorbidity between broadly-defined depressive disorders and obsessive compulsive disorder (adjusted)

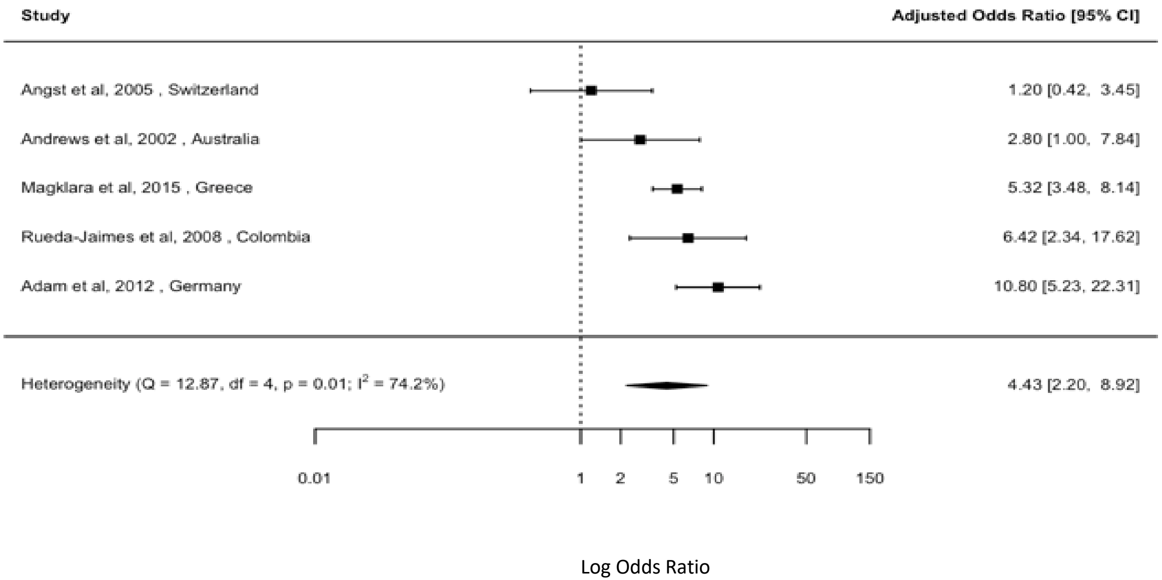

efigure 38 Funnel plot of the period prevalence comorbidity between broadly-defined depressive disorders and generalized anxiety disorder (unadjusted)

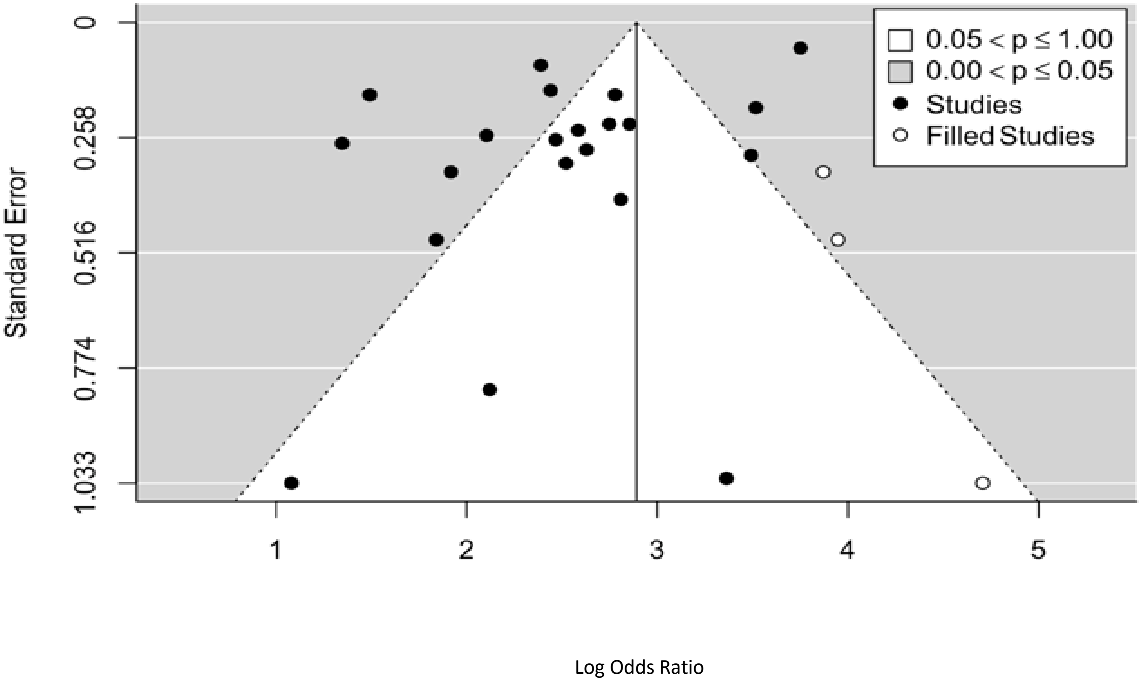

efigure 39 Funnel plot of the period prevalence comorbidity between broadly-defined depressive disorders and generalized anxiety disorder (adjusted)

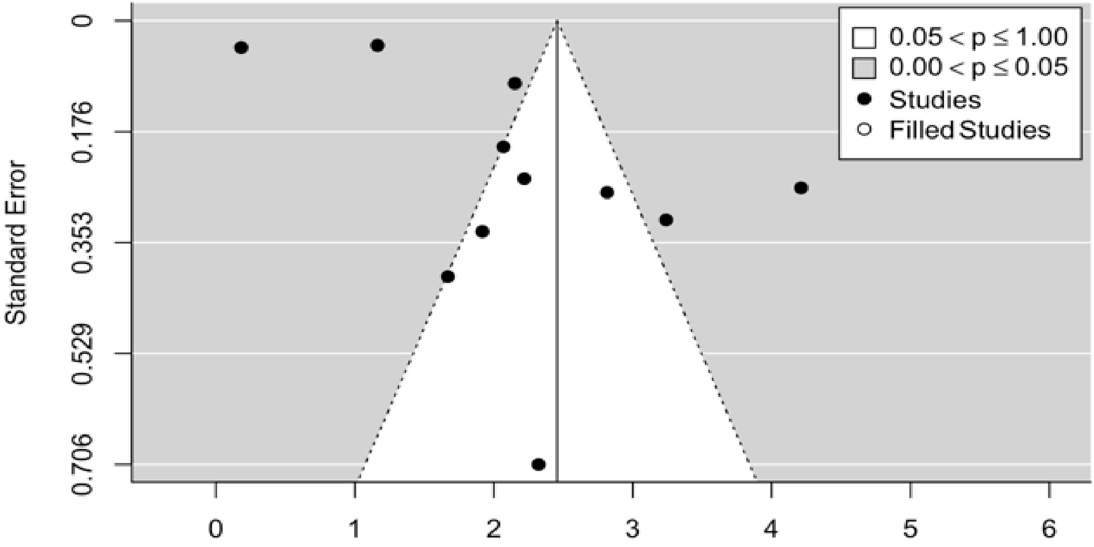

Log Odds Ratio

efigure 40 Forest plot of the random-effects meta-analysis of period prevalence comorbidity between broadly-defined depressive disorders and panic disorder (unadjusted)

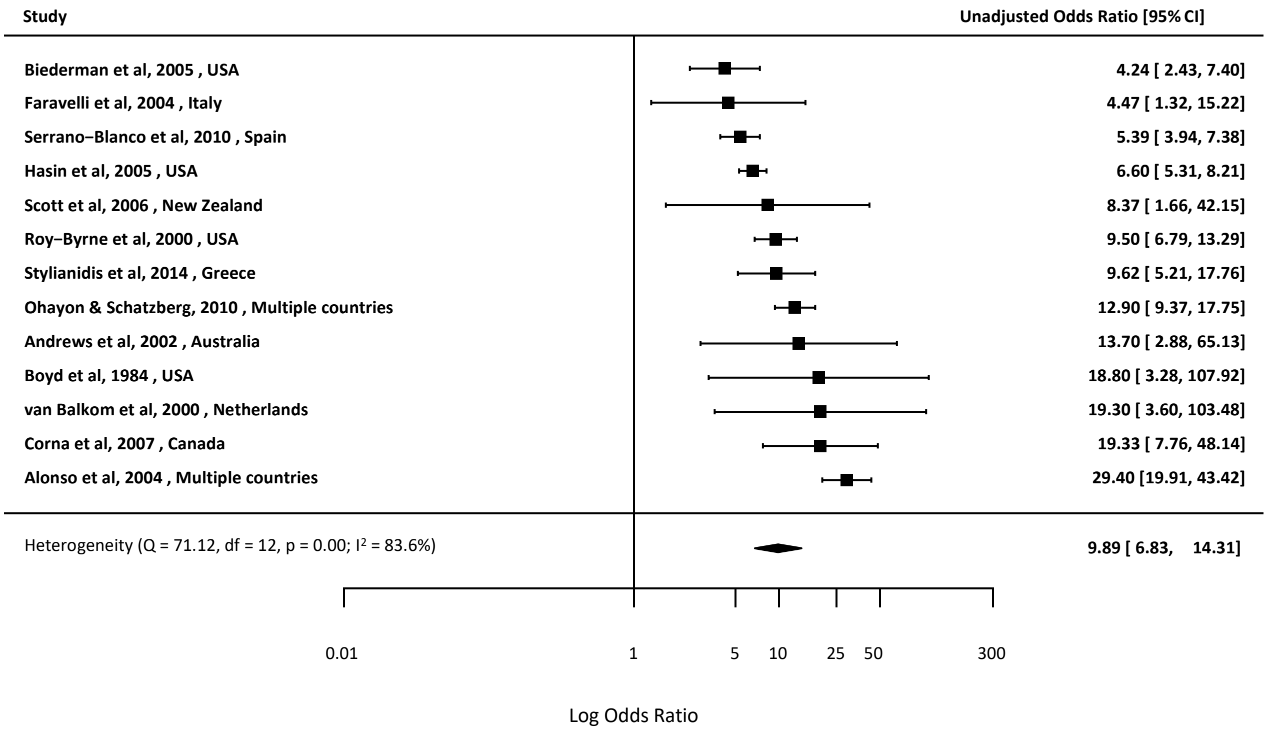

efigure 41 Funnel plot of the period prevalence comorbidity between broadly-defined depressive disorders and panic disorder (unadjusted)

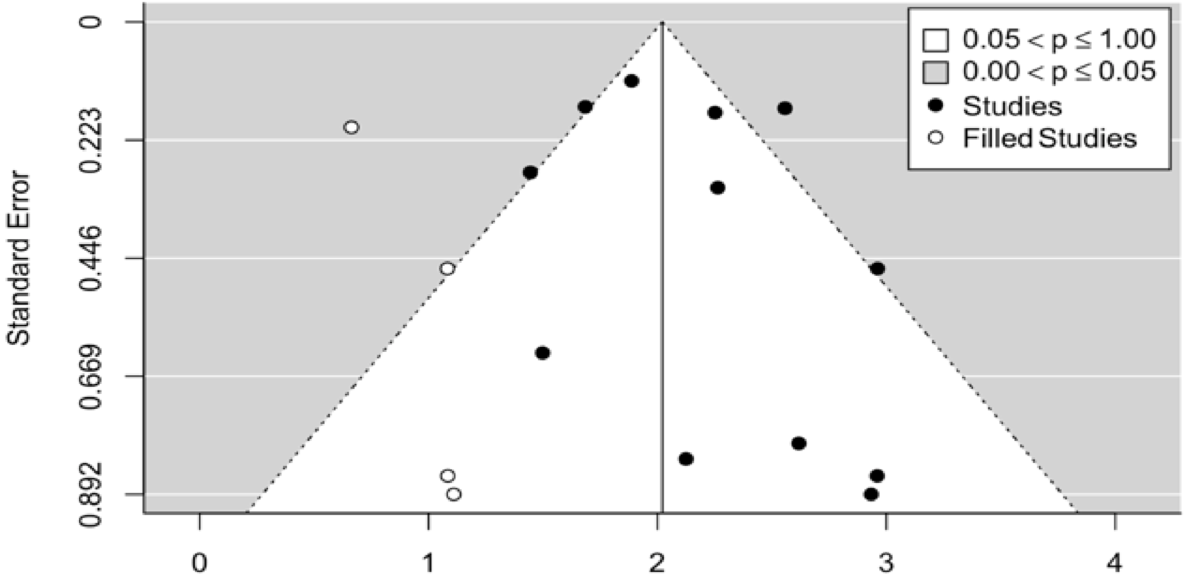

Log Odds Ratio

efigure 42 Forest plot of the random-effects meta-analysis of period prevalence comorbidity between broadly-defined depressive disorders and panic disorder (adjusted)

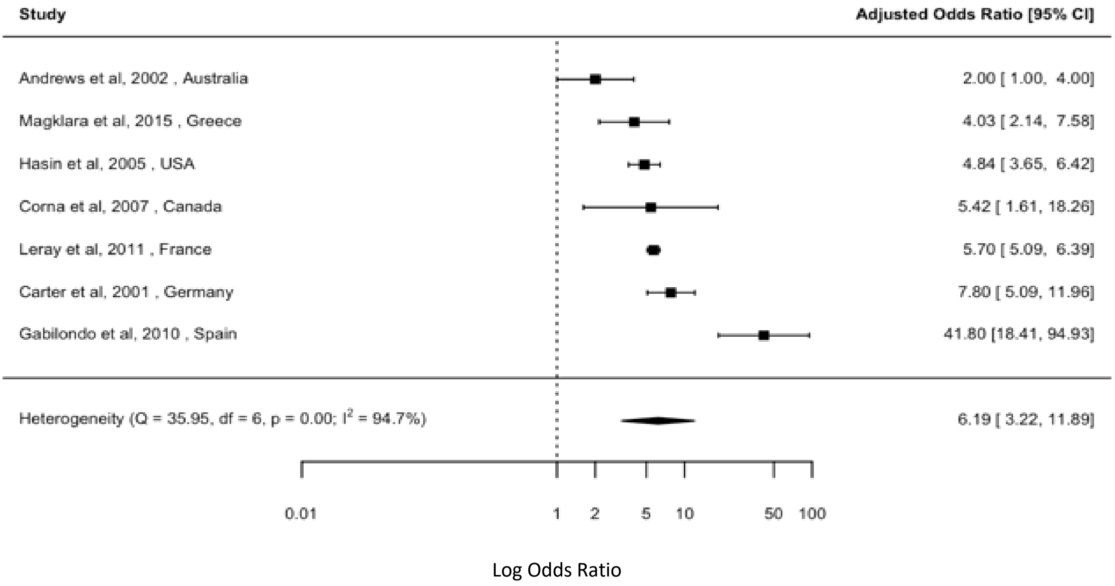

efigure 43 Forest plot of the random-effects meta-analysis of period prevalence comorbidity between broadly-defined depressive disorders and post-traumatic stress disorder (unadjusted)

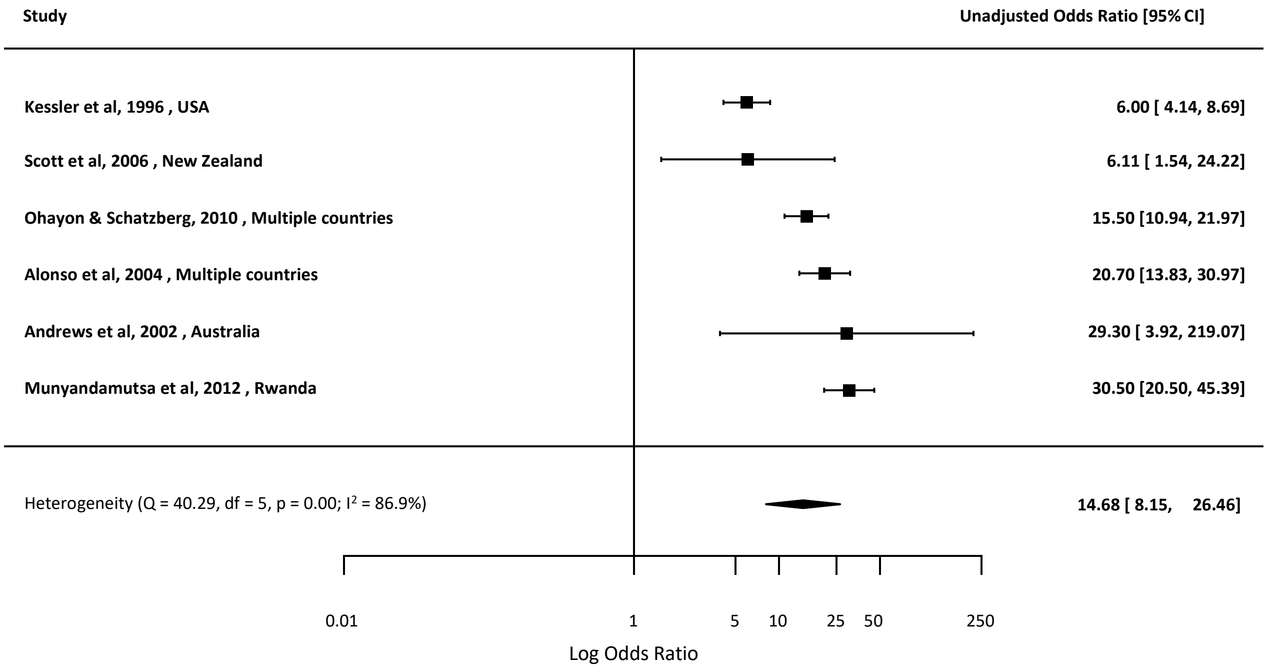

efigure 44 Forest plot of the random-effects meta-analysis of period prevalence comorbidity between broadly-defined depressive disorders and post-traumatic stress disorder (adjusted)

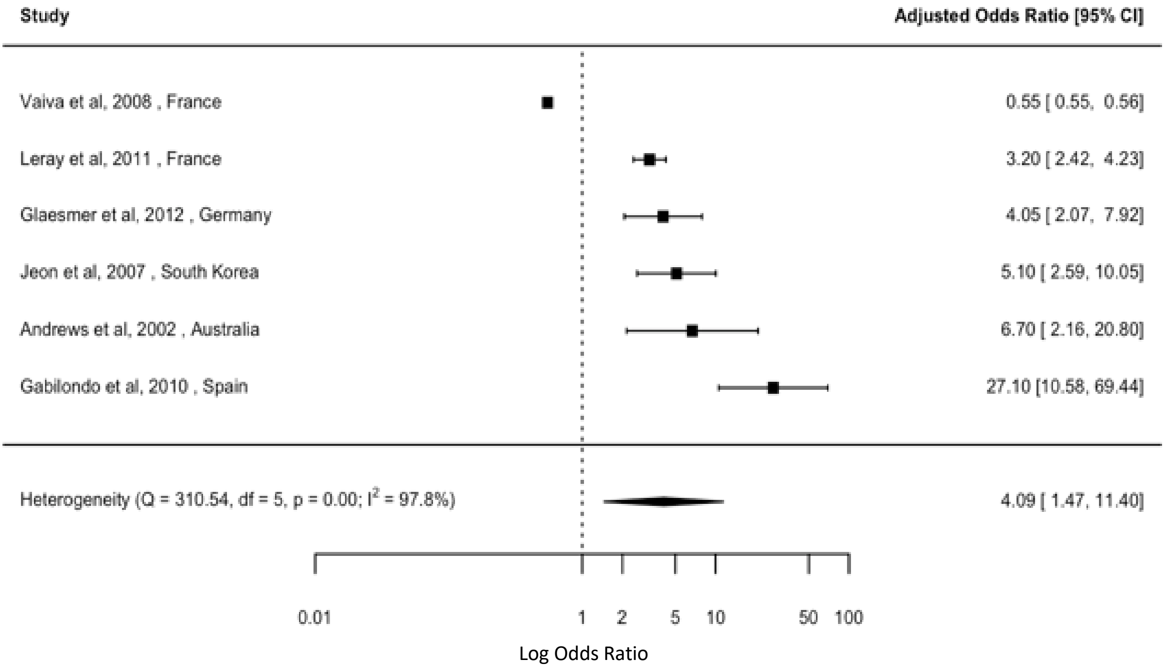

efigure 45 Forest plot of the random-effects meta-analysis of period prevalence comorbidity between broadly-defined depressive disorders and social phobia (unadjusted)

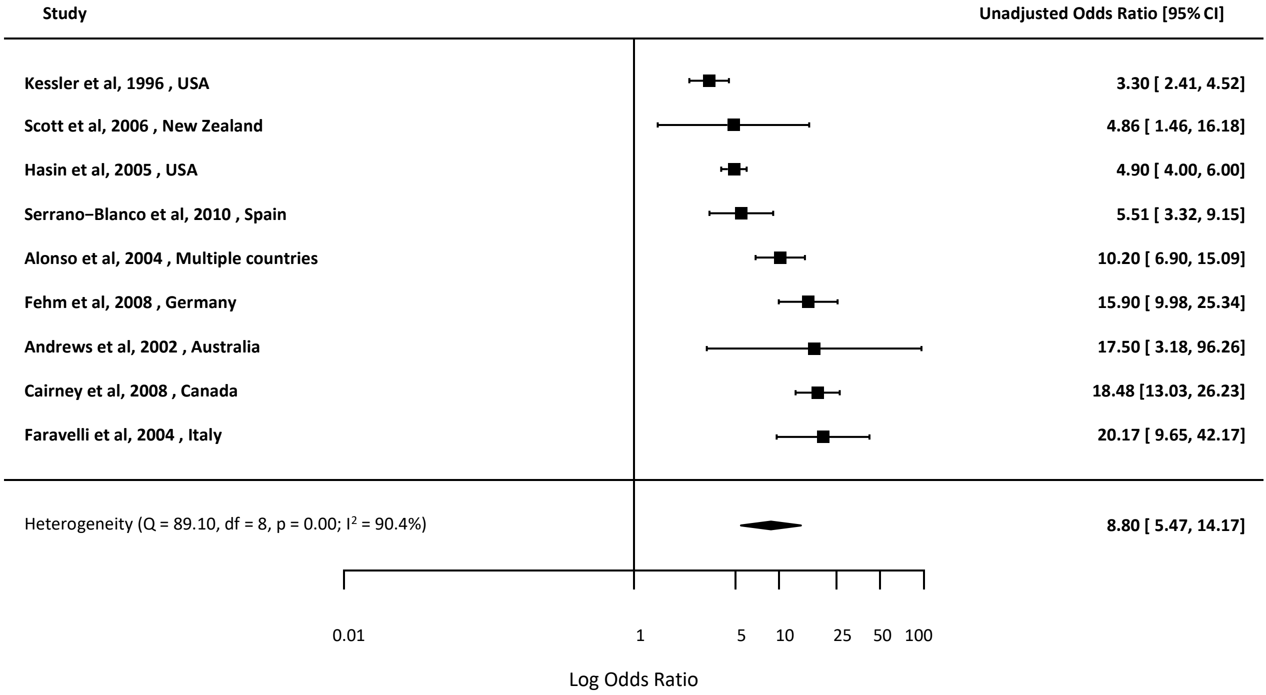

efigure 46 Forest plot of the random-effects meta-analysis of period prevalence comorbidity between broadly-defined depressive disorders and social phobia (adjusted)

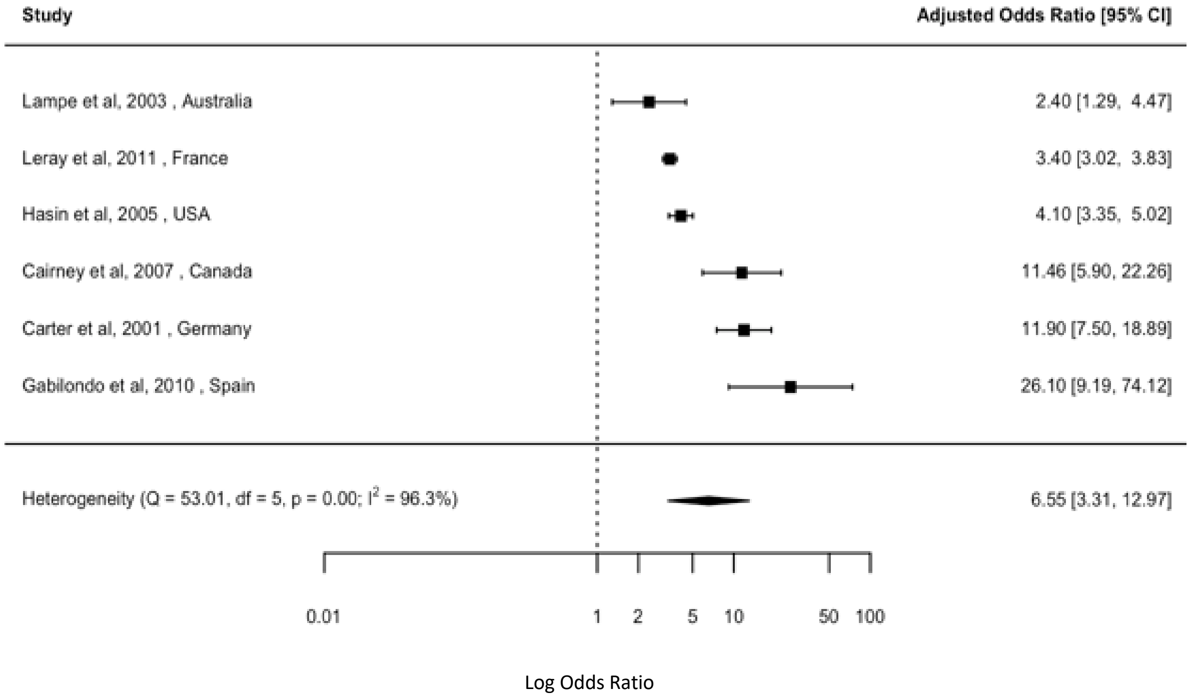

efigure 47 Forest plot of the random-effects meta-analysis of period prevalence comorbidity between broadly-defined depressive disorders and specific phobia (unadjusted)

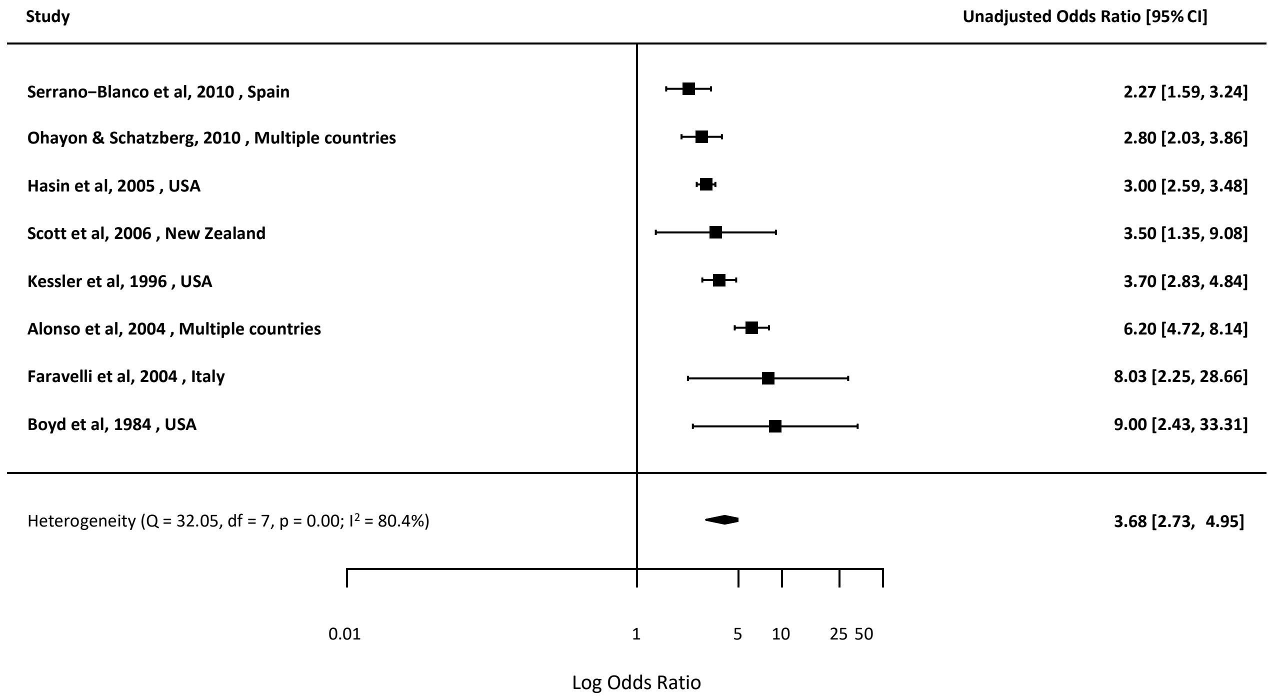

efigure 48 Forest plot of the random-effects meta-analysis of period prevalence comorbidity between broadly-defined depressive disorders and specific phobia (adjusted)

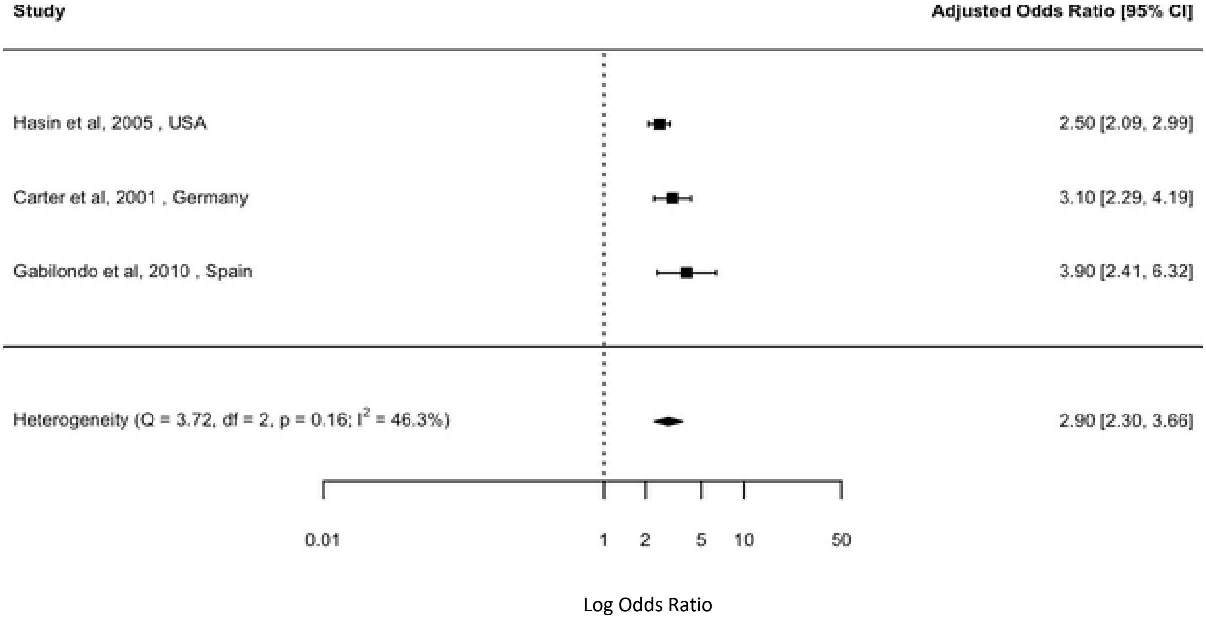

efigure 49 Forest plot of the random-effects meta-analysis of period prevalence comorbidity between broadly-defined depressive disorders and broadly-defined anxiety disorder (unadjusted)

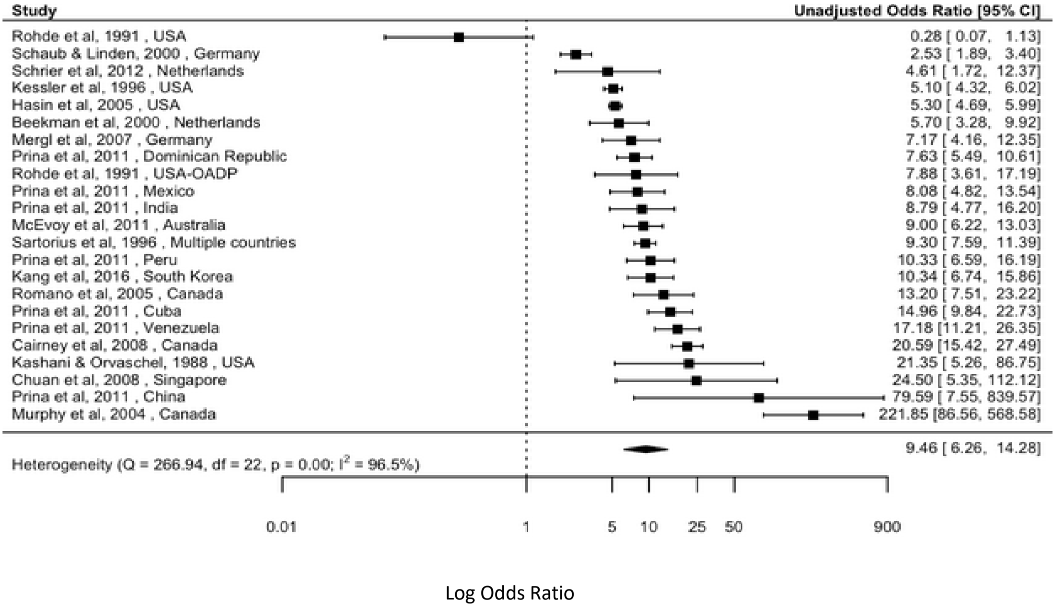

efigure 50 Forest plot of the period prevalence comorbidity between broadly-defined depressive disorders and broadly-defined anxiety disorder (unadjusted)

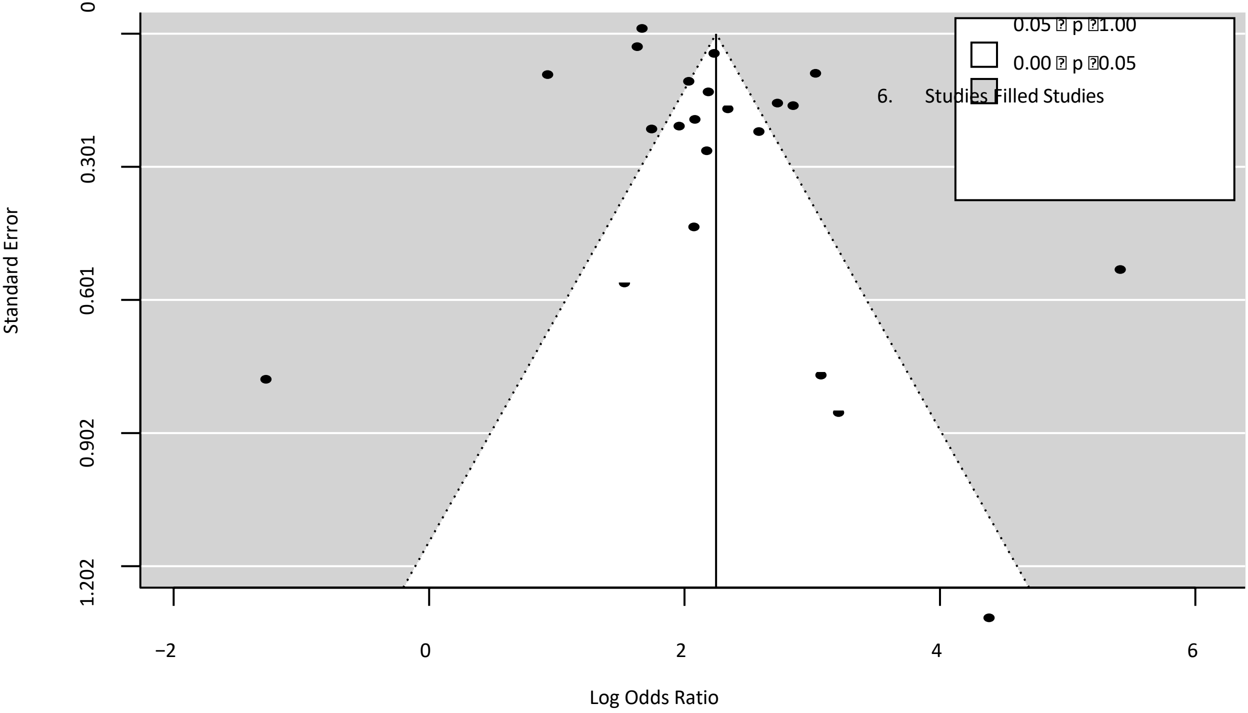

efigure 51 Forest plot of the random-effects meta-analysis of period prevalence comorbidity between broadly-defined depressive disorders and broadly-defined anxiety disorder (adjusted)

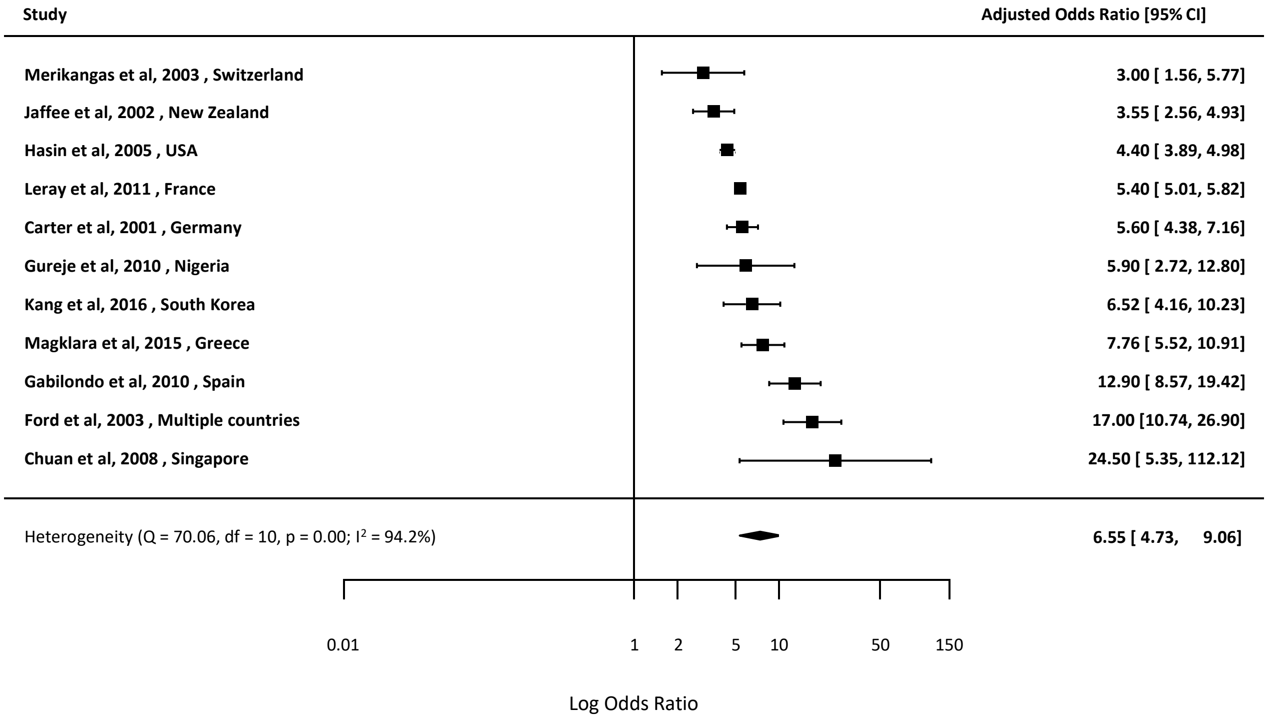

efigure 52 Forest plot of the period prevalence comorbidity between broadly-defined depressive disorders and broadly-defined anxiety disorder (adjusted)

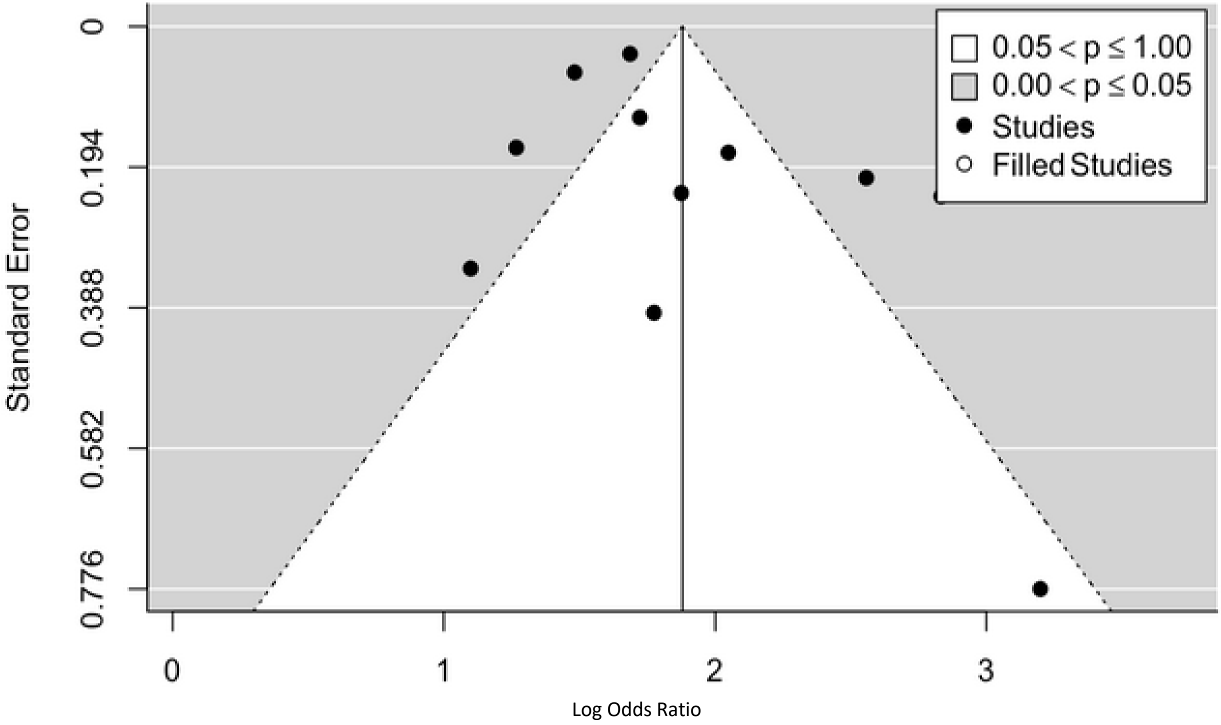

efigure 53 Forest plot of the random-effects meta-analysis of lifetime comorbidity between broadly-defined dysthymic disorders and obsessive compulsive disorder (unadjusted)

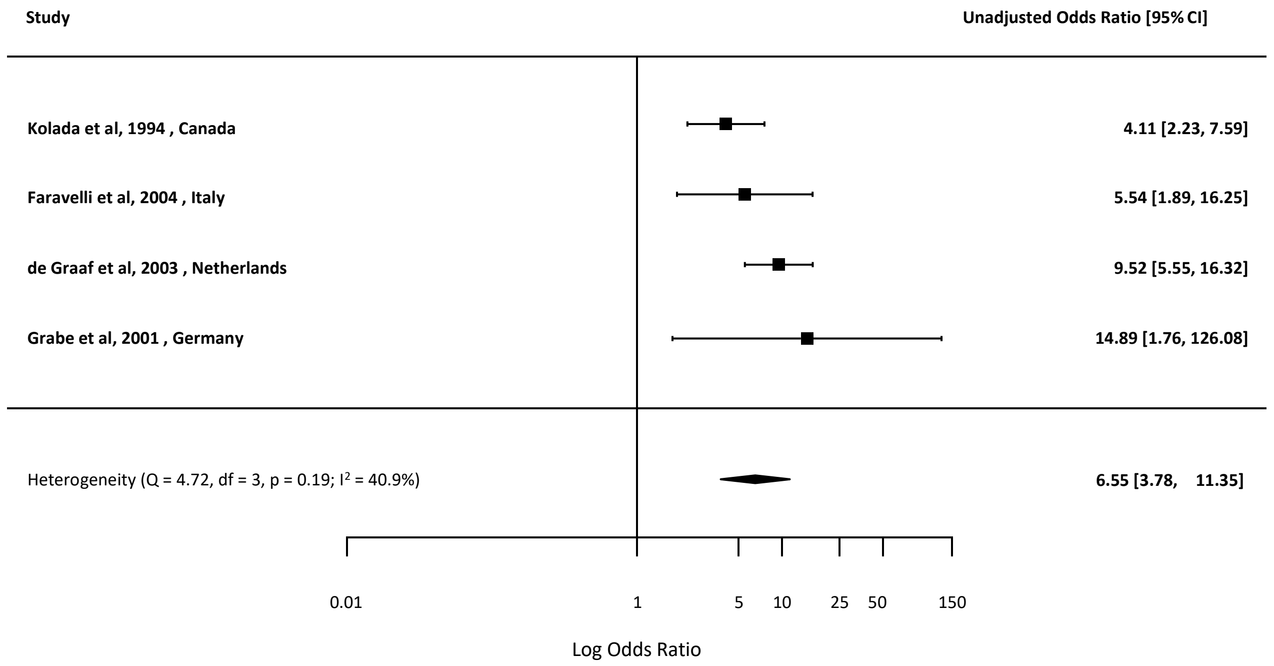

efigure 54 Forest plot of the random-effects meta-analysis of lifetime comorbidity between broadly-defined dysthymic disorders and generalized anxiety disorder (unadjusted)

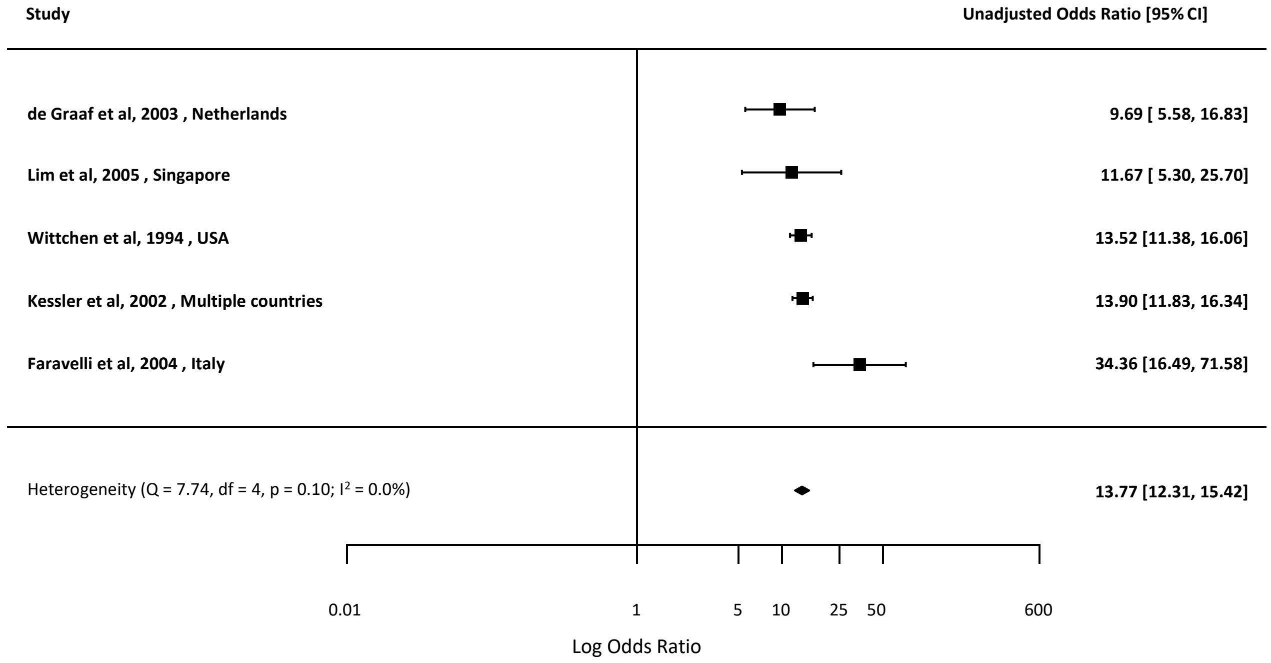

efigure 55 Forest plot of the random-effects meta-analysis of lifetime comorbidity between broadly-defined dysthymic disorders and social phobia (unadjusted)

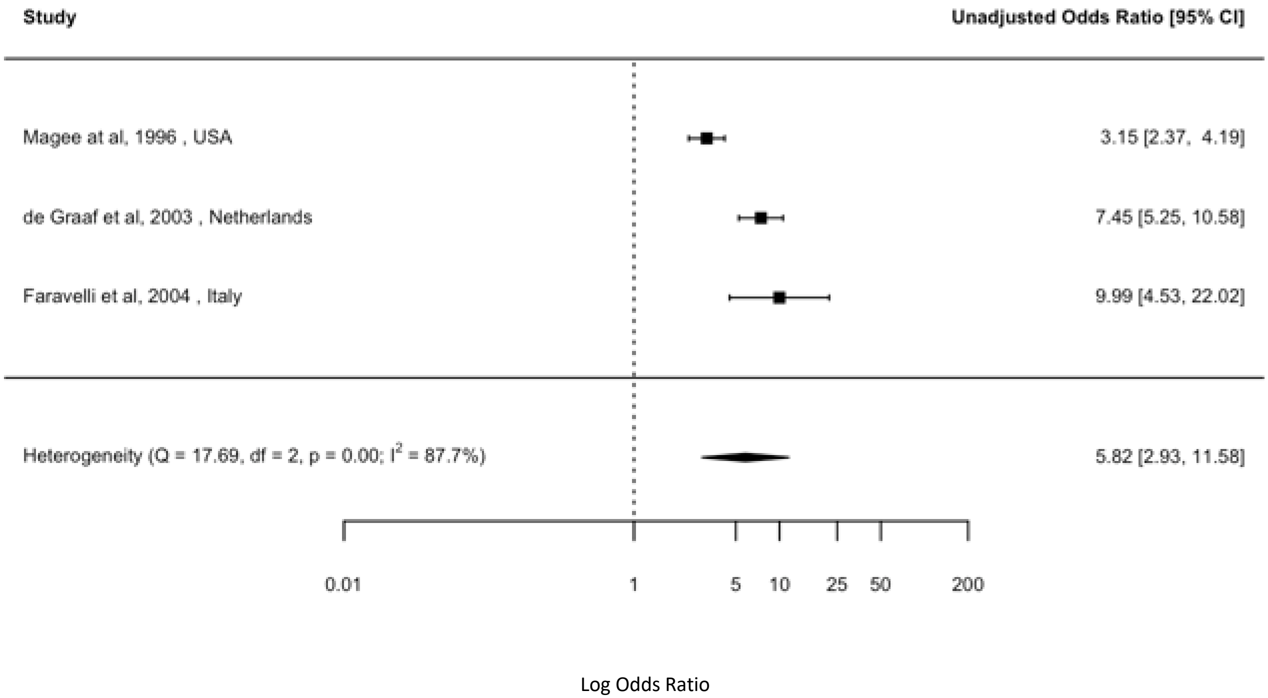

efigure 56 Forest plot of the random-effects meta-analysis of lifetime comorbidity between broadly-defined dysthymic disorders and social phobia (adjusted)

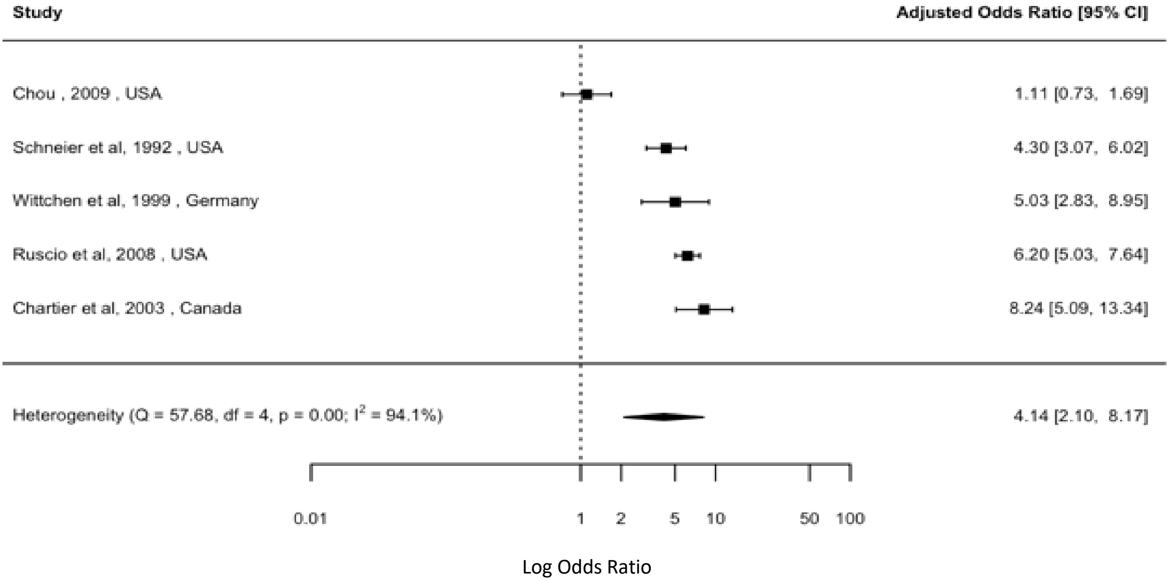

efigure 57 Forest plot of the random-effects meta-analysis of lifetime comorbidity between broadly-defined dysthymic disorders and specific phobia (unadjusted)

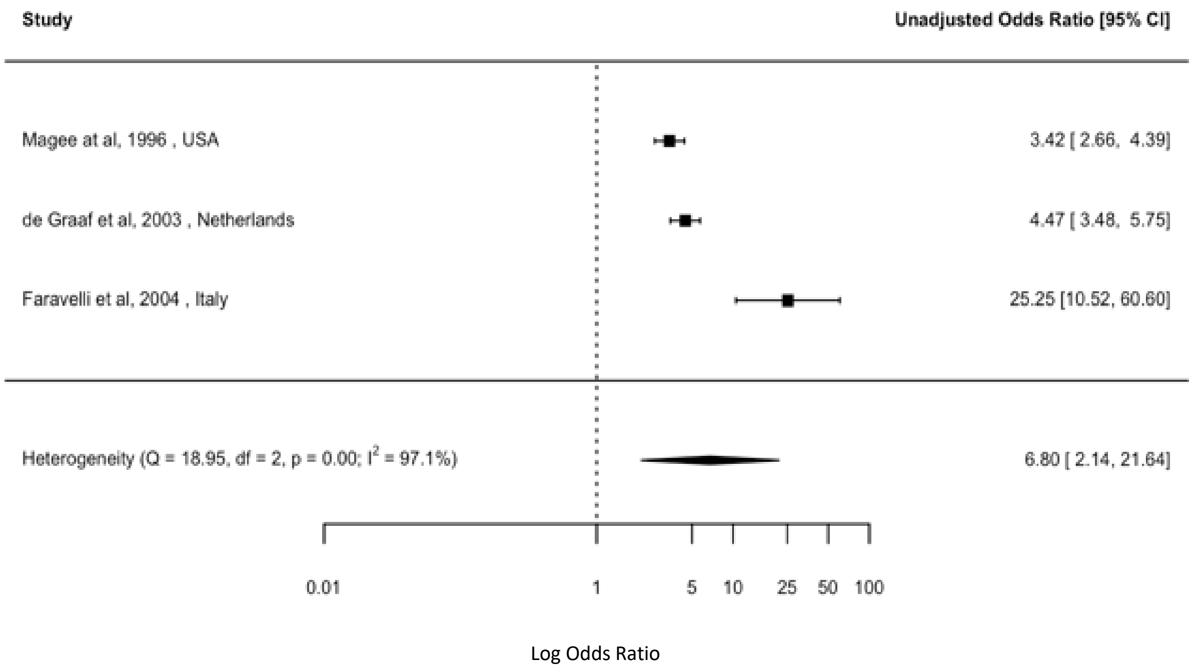

efigure 58 Forest plot of the random-effects meta-analysis of period prevalence comorbidity between broadly-defined dysthymic disorders and agoraphobia (unadjusted)

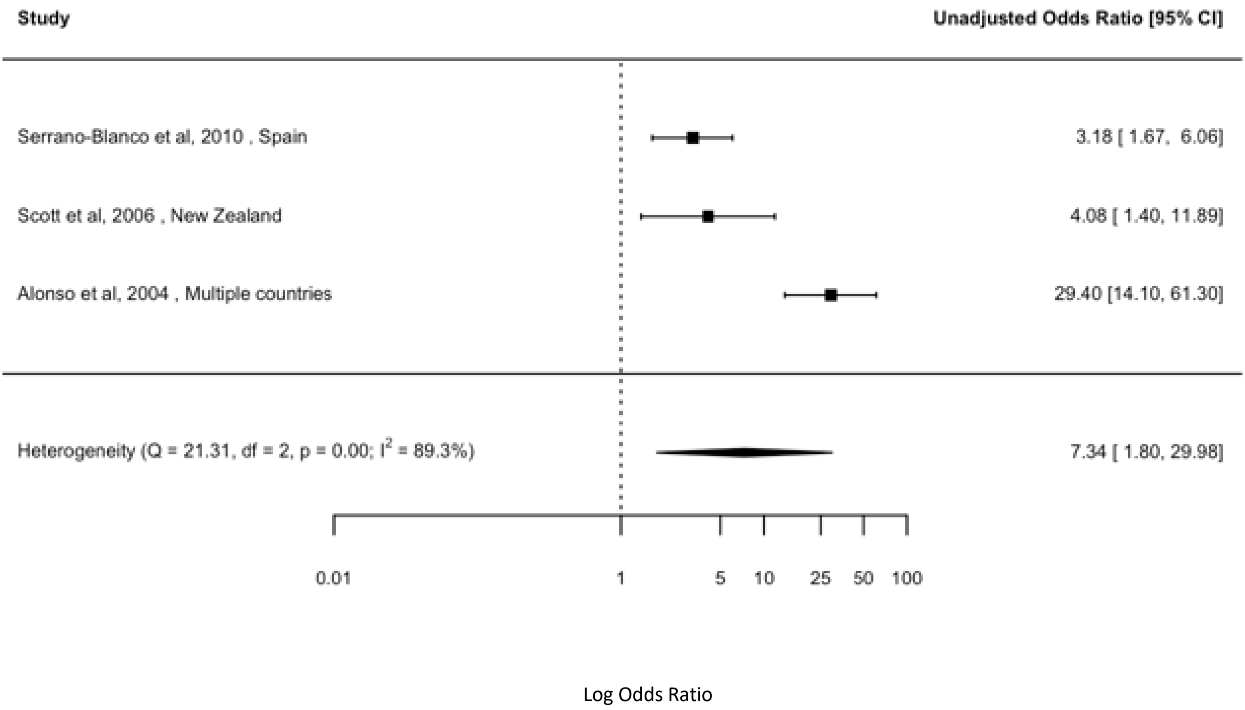

efigure 59 Forest plot of the random-effects meta-analysis of lifetime comorbidity between broadly-defined dysthymic disorders and obsessive compulsive disorder (unadjusted)

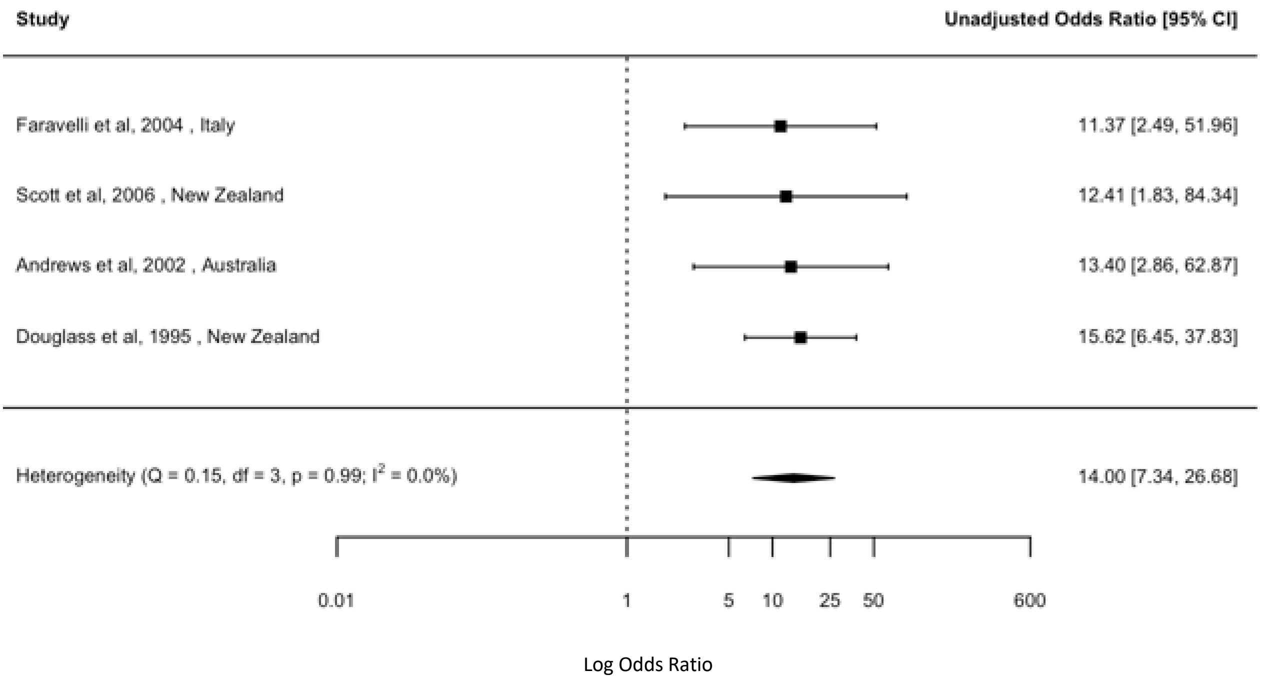

efigure 60 Forest plot of the random-effects meta-analysis of lifetime comorbidity between broadly-defined dysthymic disorders and generalised anxiety disorder (unadjusted)

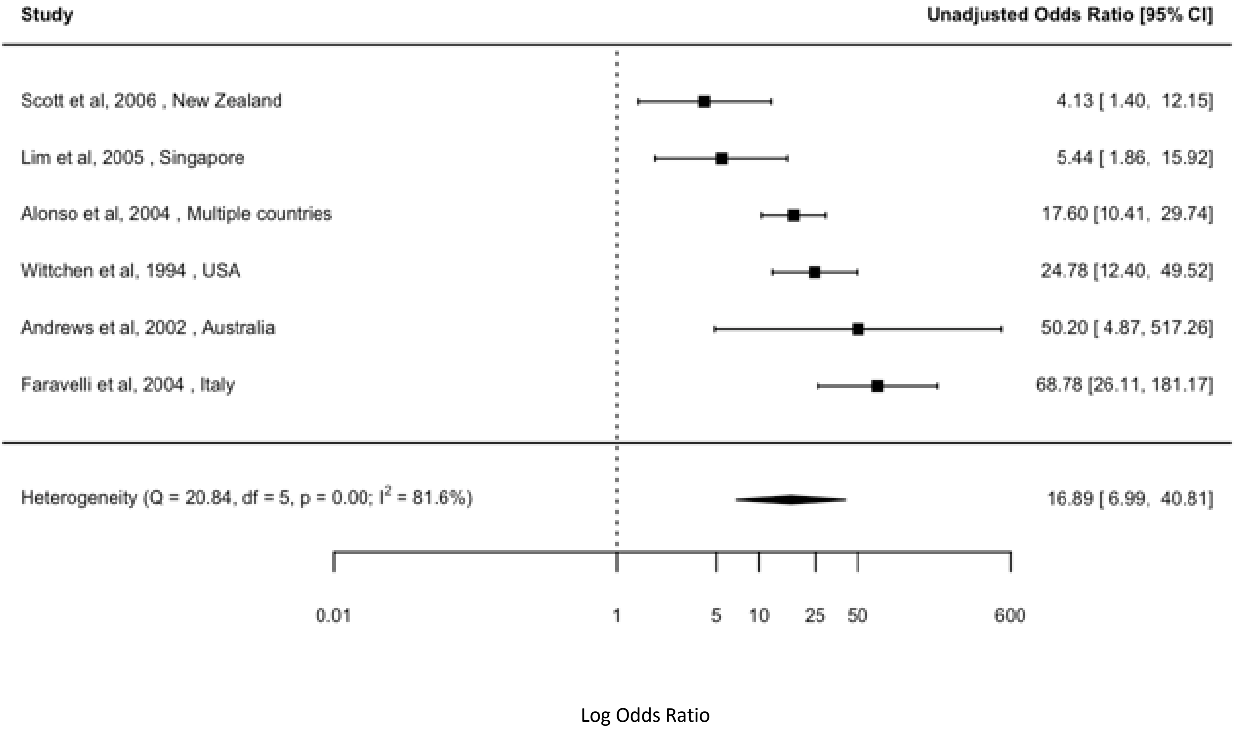

efigure 61 Forest plot of the random-effects meta-analysis of lifetime comorbidity between broadly-defined dysthymic disorders and generalized anxiety disorder (adjusted)

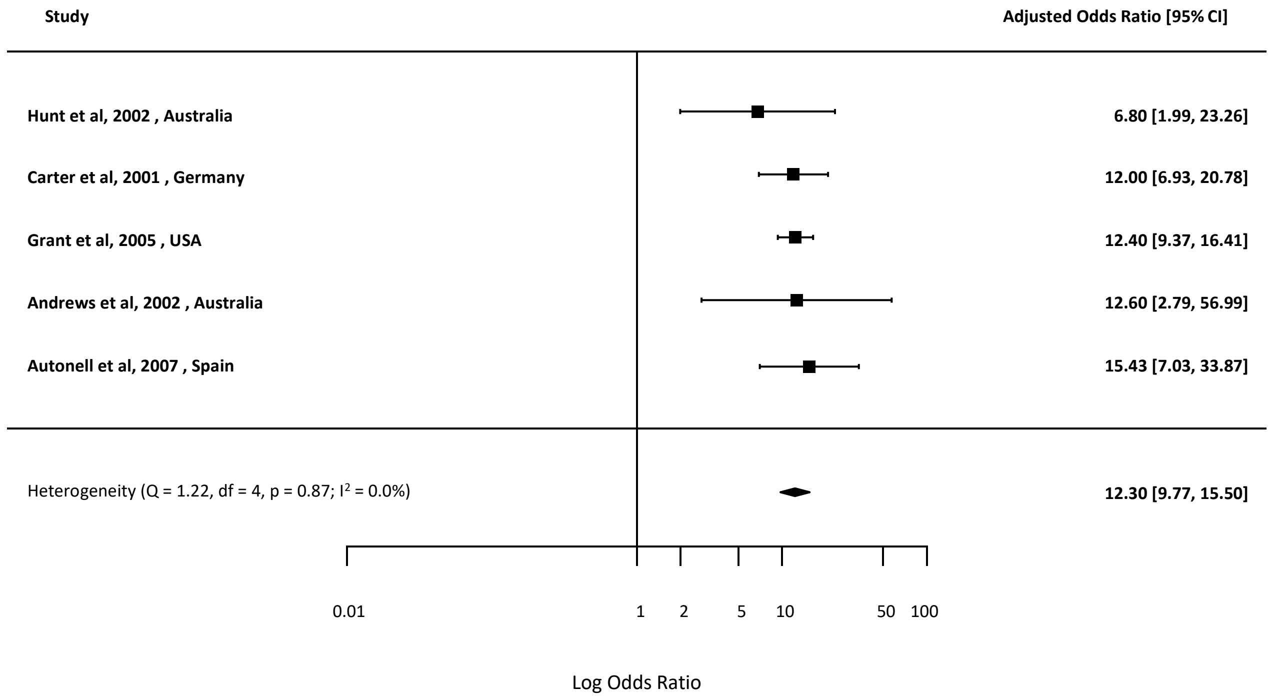

efigure 62 Forest plot of the random-effects meta-analysis of lifetime comorbidity between broadly-defined dysthymic disorders and panic disorder (unadjusted)

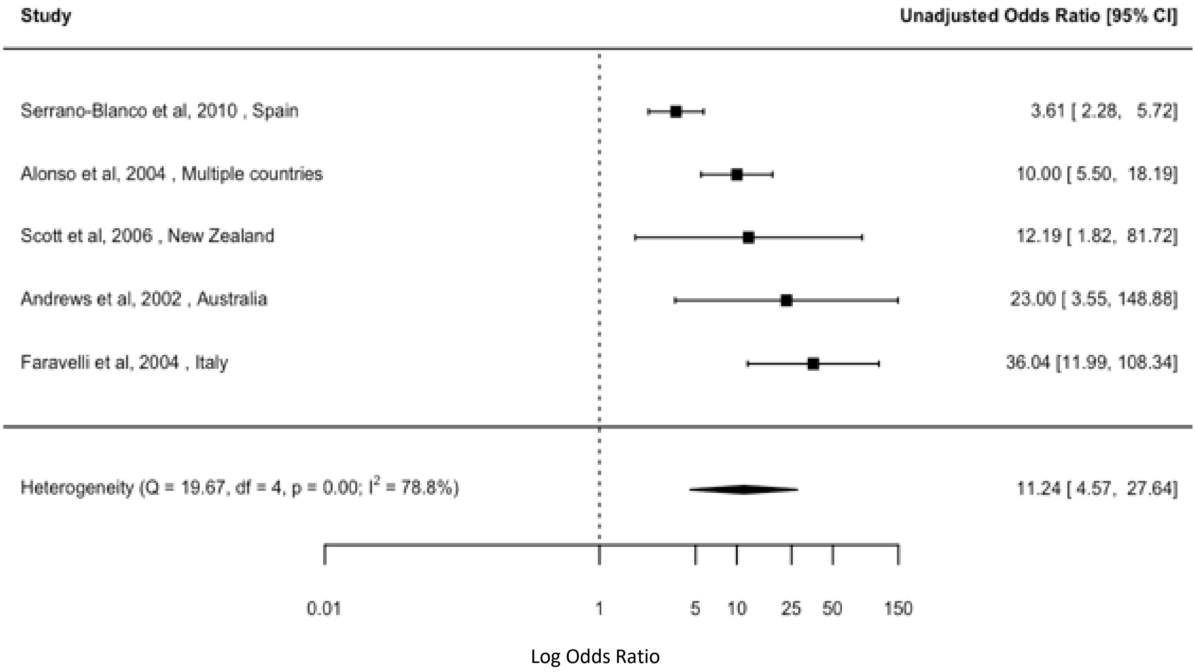

efigure 63 Forest plot of the random-effects meta-analysis of lifetime comorbidity between broadly-defined dysthymic disorders and post-traumatic stress disorder (unadjusted)

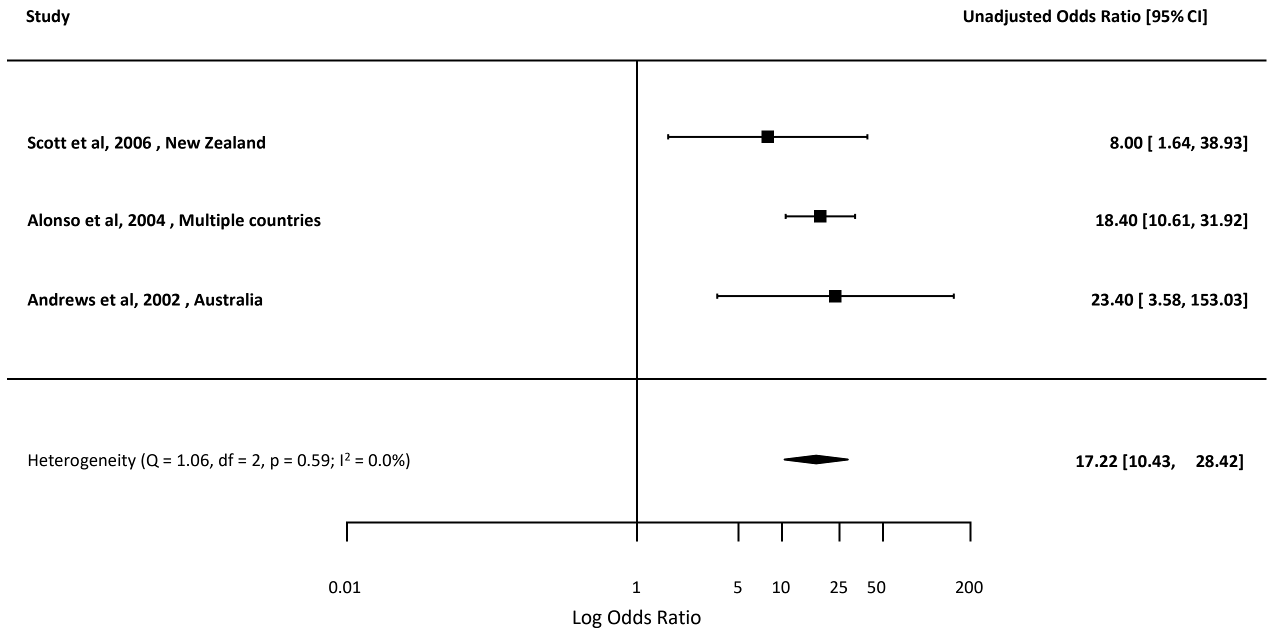

efigure 64 Forest plot of the random-effects meta-analysis of lifetime comorbidity between broadly-defined dysthymic disorders and post-traumatic stress disorder (adjusted)

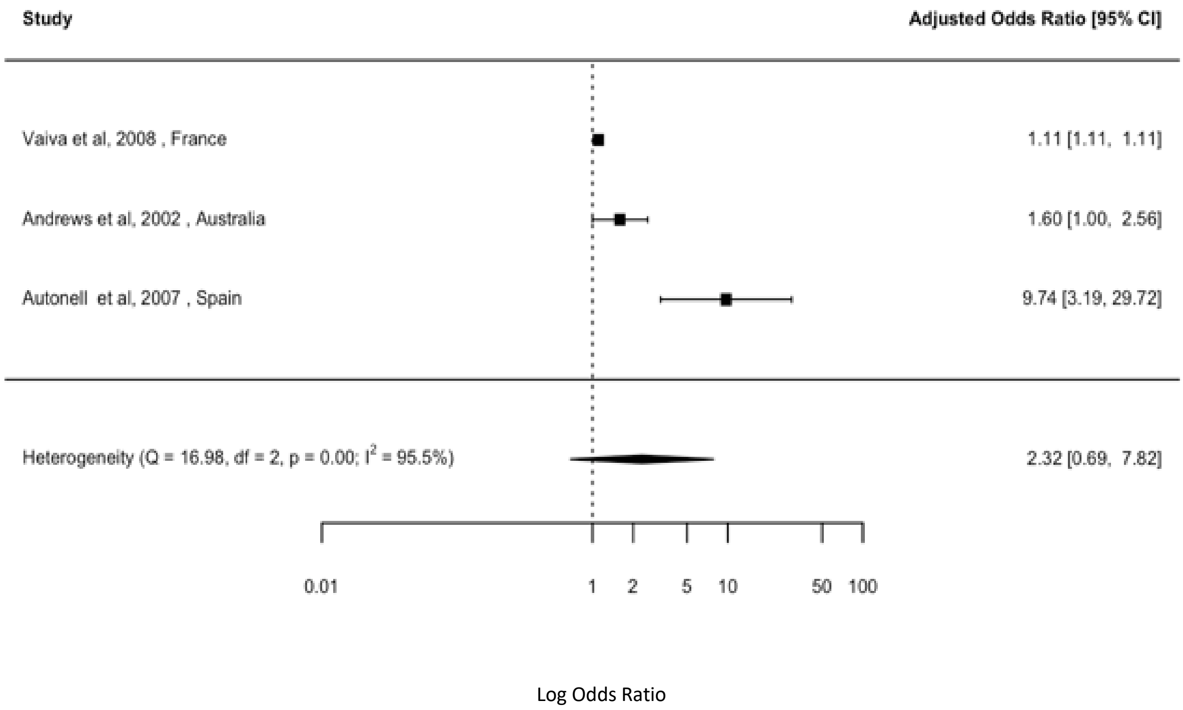

efigure 65 Forest plot of the random-effects meta-analysis of lifetime comorbidity between broadly-defined dysthymic disorders and social phobia (unadjusted)

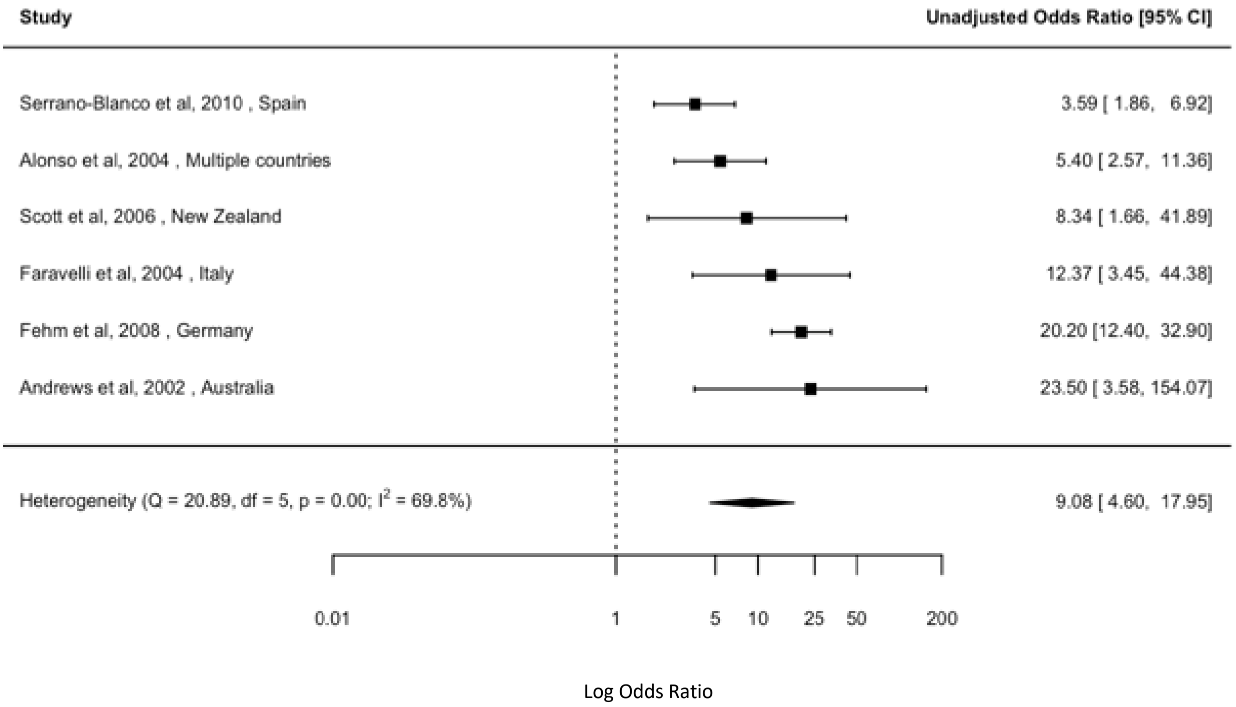

efigure 66 Forest plot of the random-effects meta-analysis of lifetime comorbidity between broadly-defined dysthymic disorders and social phobia (adjusted)

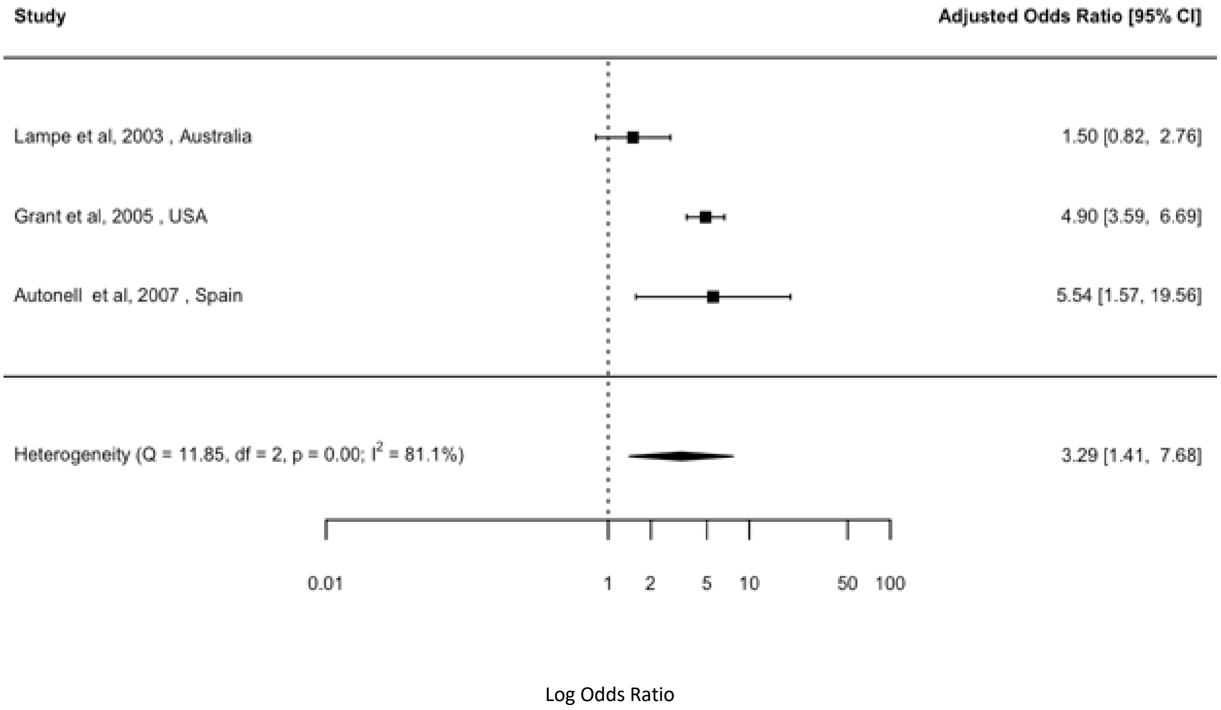

efigure 67 Forest plot of the random-effects meta-analysis of lifetime comorbidity between broadly-defined dysthymic disorders and specific phobia (unadjusted)

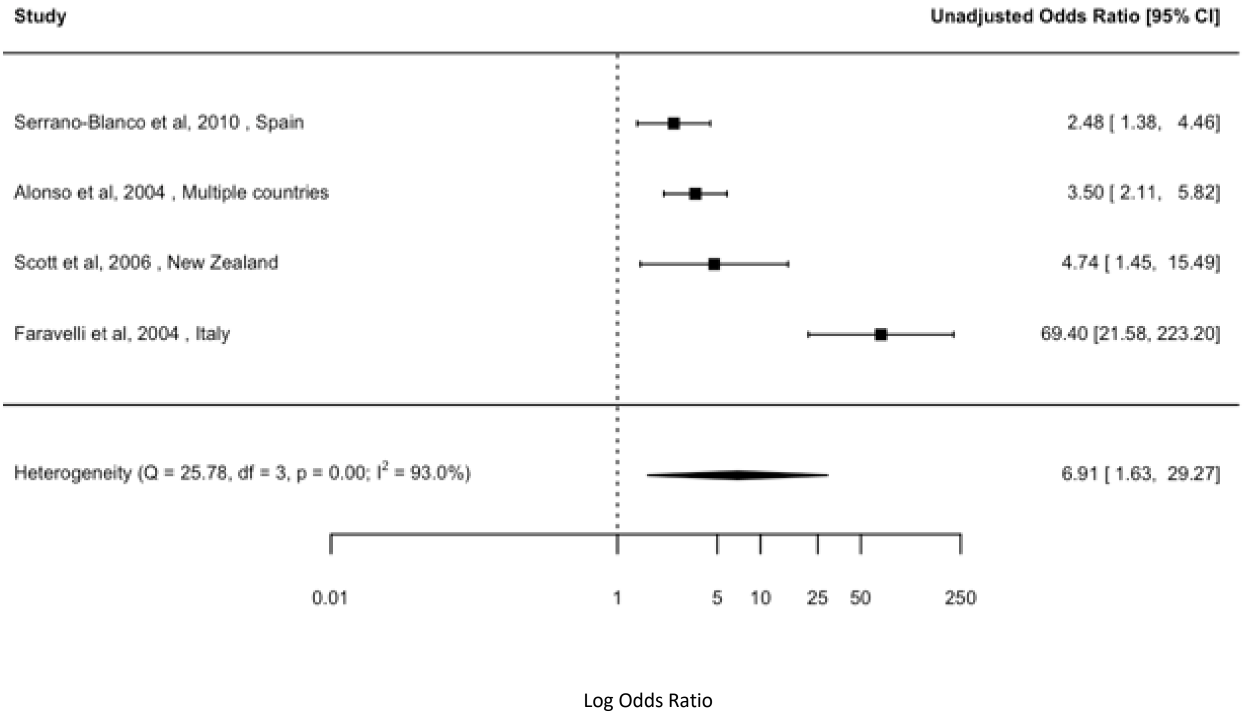

efigure 68 Forest plot of the random-effects meta-analysis of lifetime comorbidity between broadly-defined bipolar disorders and agoraphobia (unadjusted)

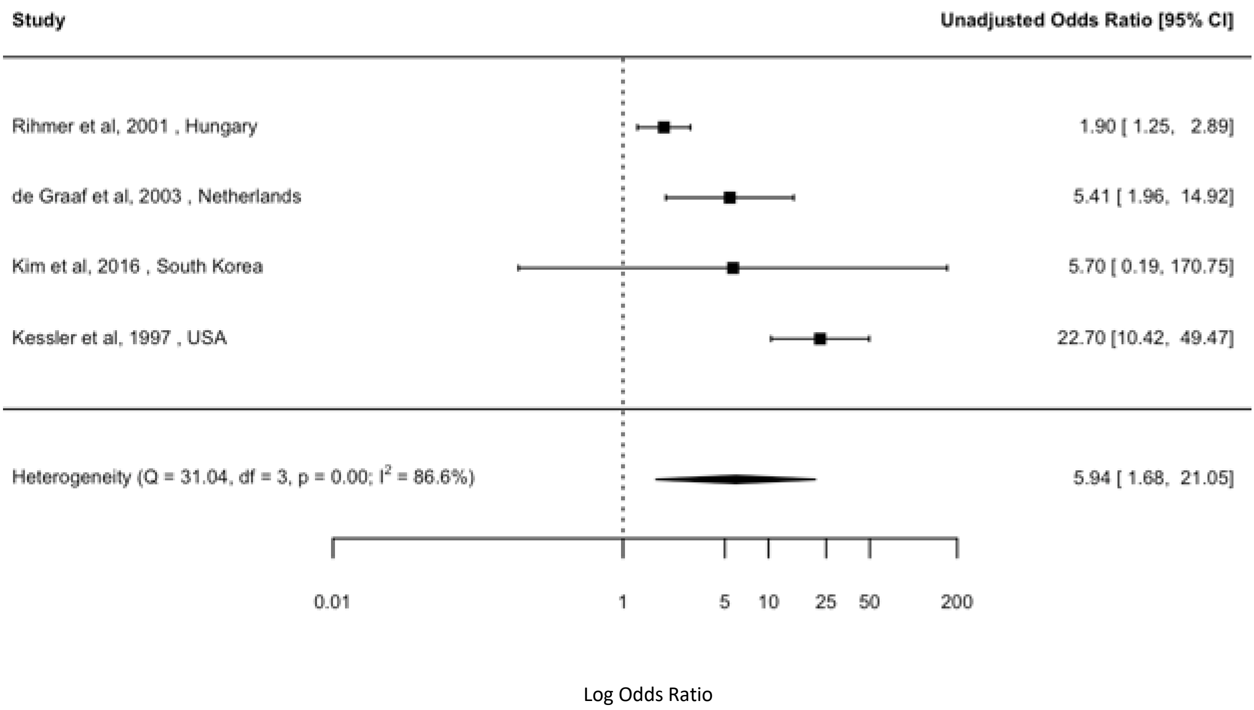

efigure 69 Forest plot of the random-effects meta-analysis of lifetime comorbidity between broadly-defined bipolar disorders and obsessive compulsive disorder (unadjusted)

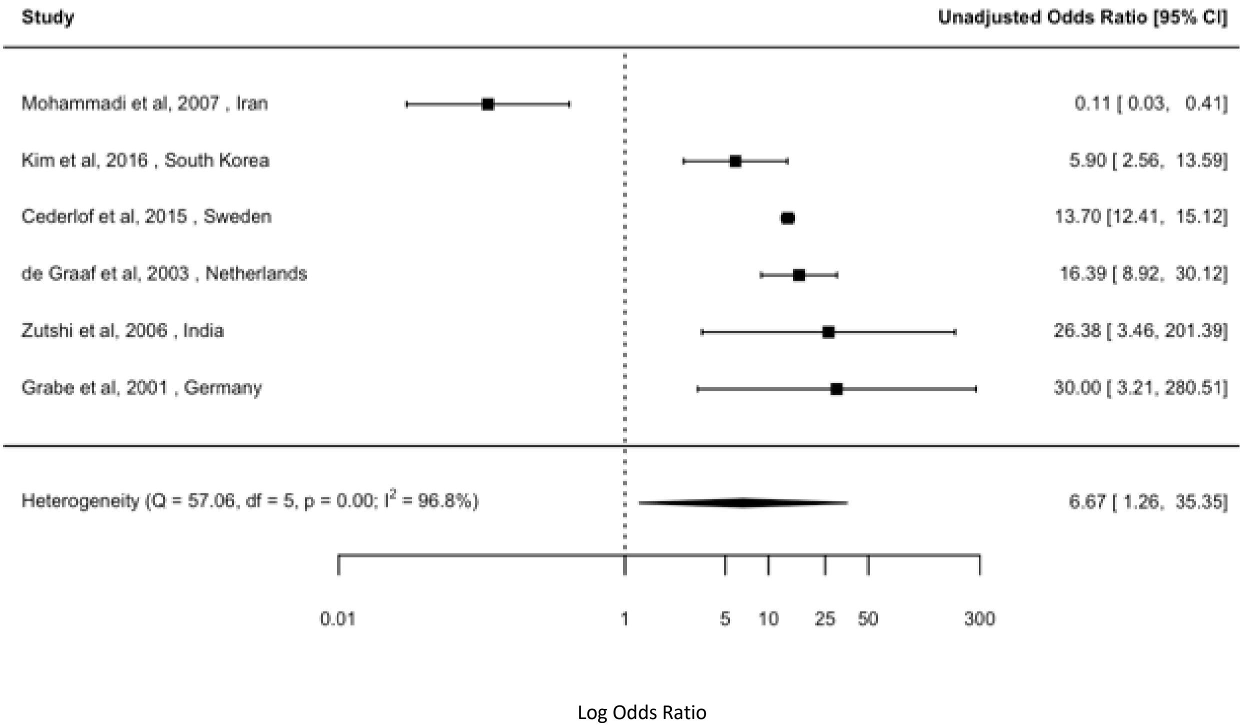

efigure 70 Forest plot of the random-effects meta-analysis of lifetime comorbidity between broadly-defined bipolar disorders and obsessive compulsive disorder (adjusted)

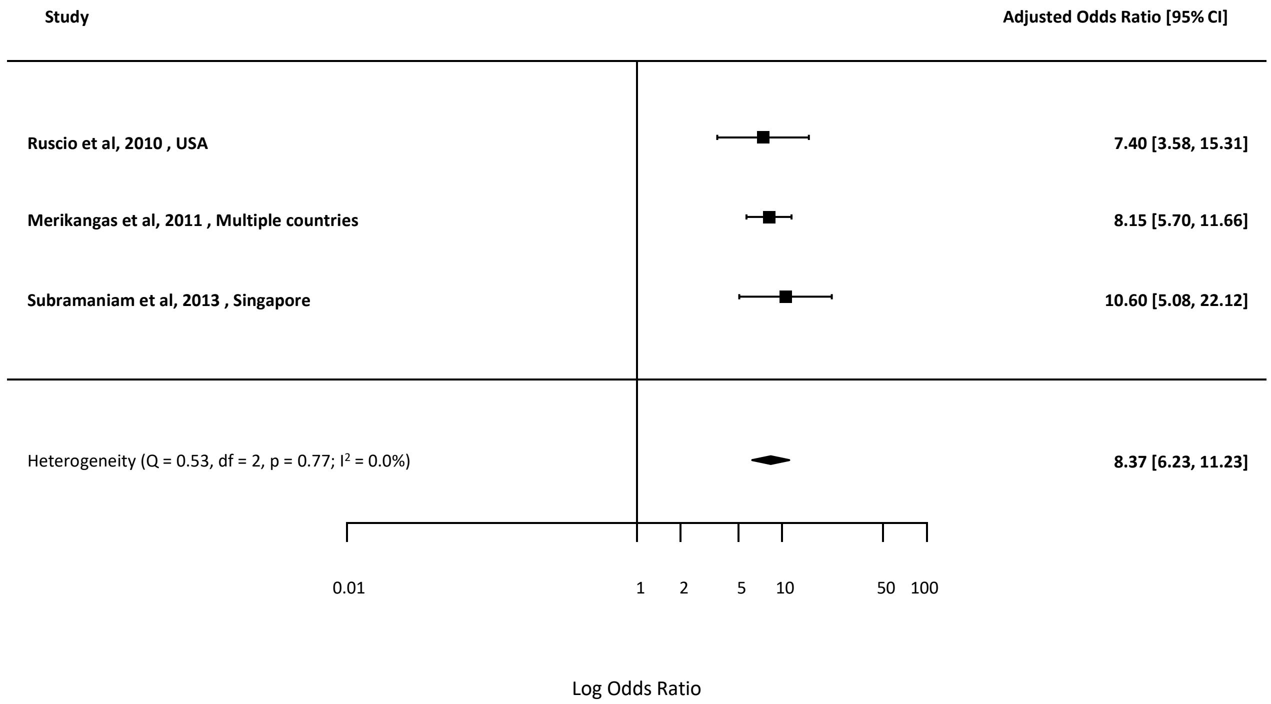

efigure 71 Forest plot of the random-effects meta-analysis of lifetime comorbidity between broadly-defined bipolar disorders and generalized anxiety disorder (unadjusted)

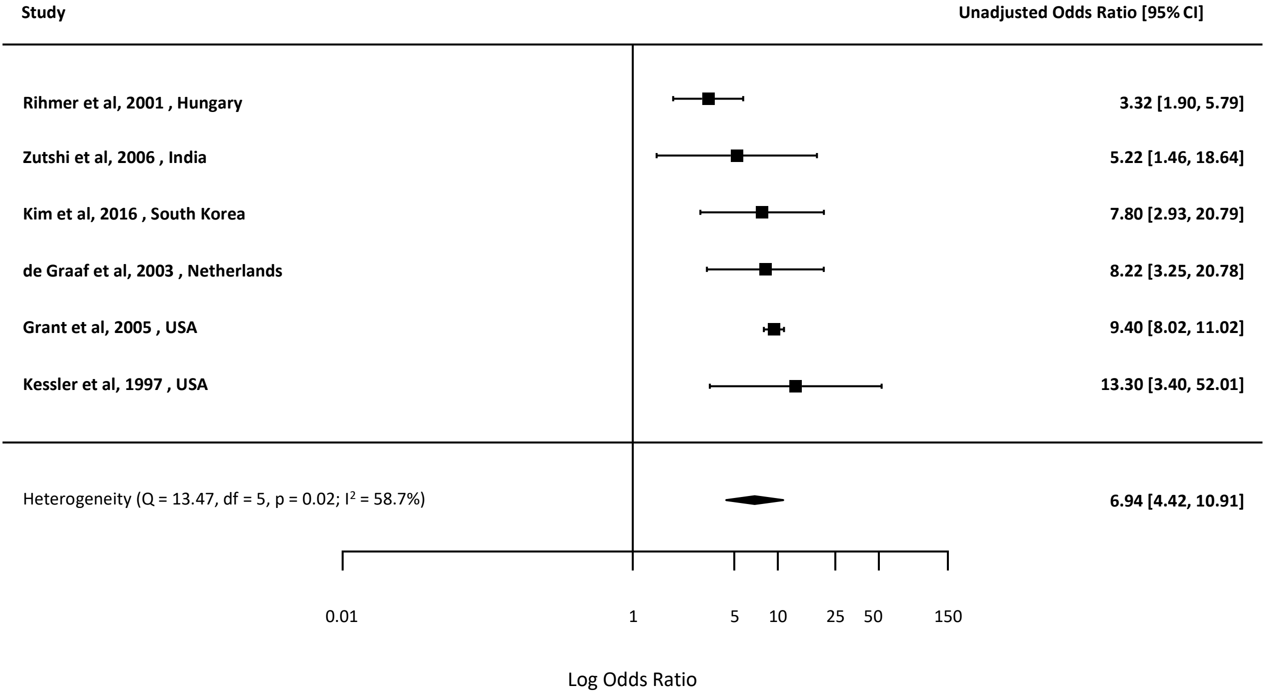

efigure 72 Forest plot of the random-effects meta-analysis of lifetime comorbidity between broadly-defined bipolar disorders and generalized anxiety disorder (adjusted)

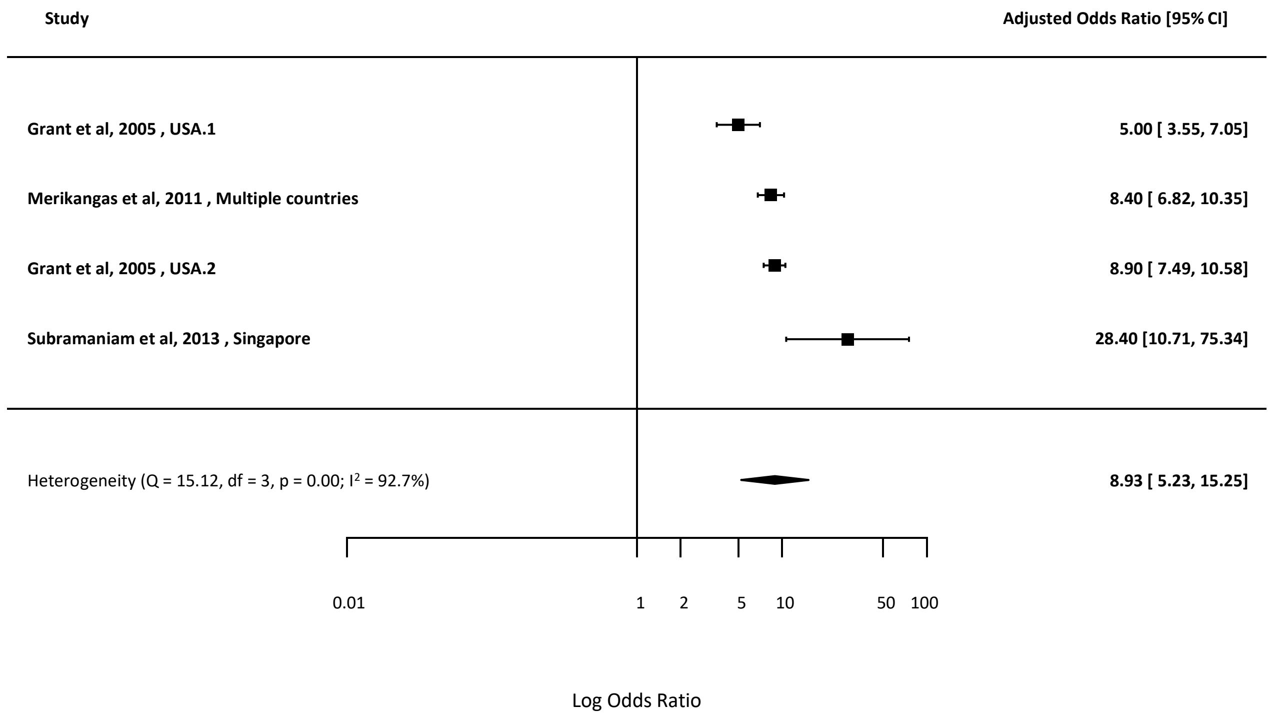

efigure 73 Forest plot of the random-effects meta-analysis of lifetime comorbidity between broadly-defined bipolar disorders and panic disorder (unadjusted)

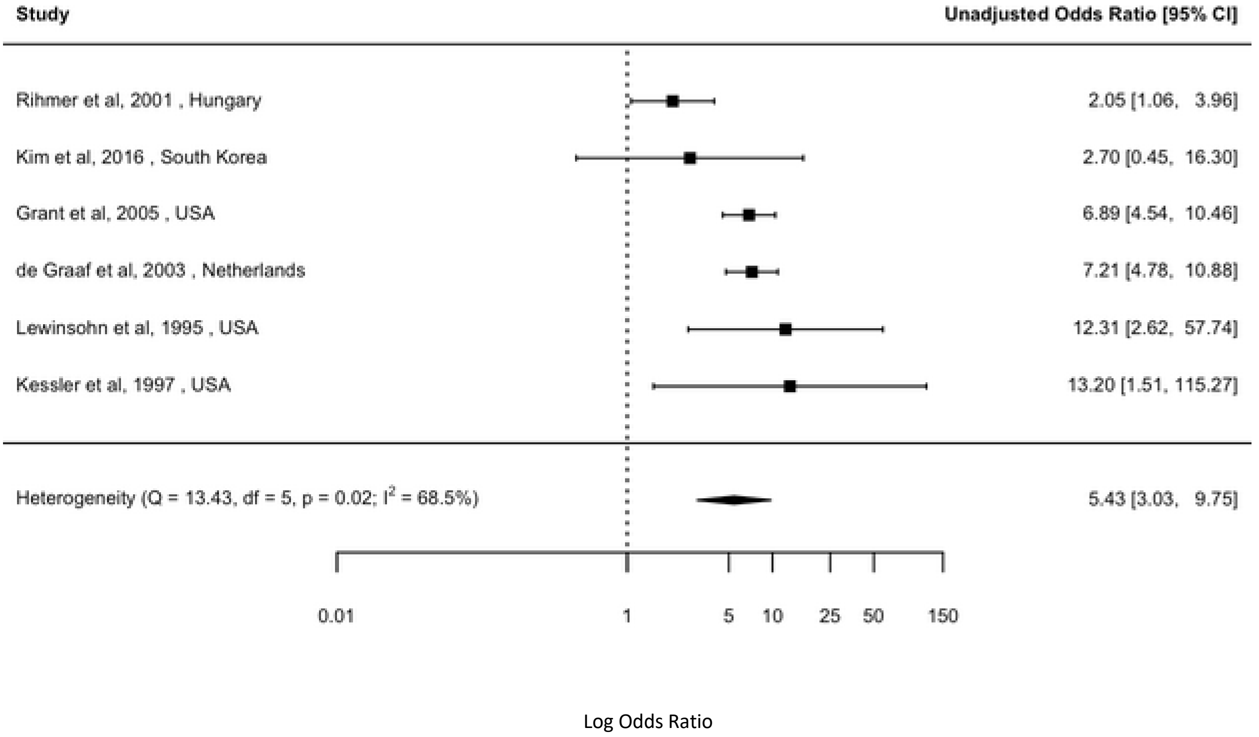

efigure 74 Forest plot of the random-effects meta-analysis of lifetime comorbidity between broadly-defined bipolar disorders and panic disorder (adjusted)

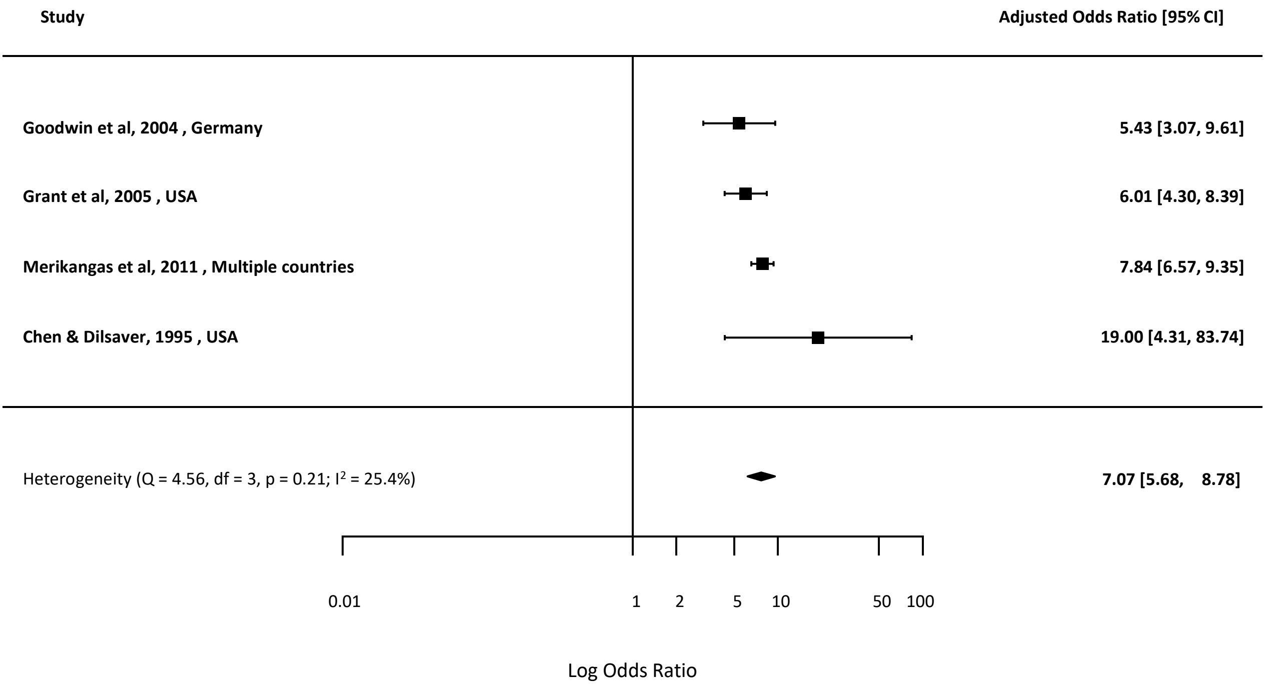

efigure 75 Forest plot of the random-effects meta-analysis of lifetime comorbidity between broadly-defined bipolar disorders and post-traumatic stress disorder (adjusted)

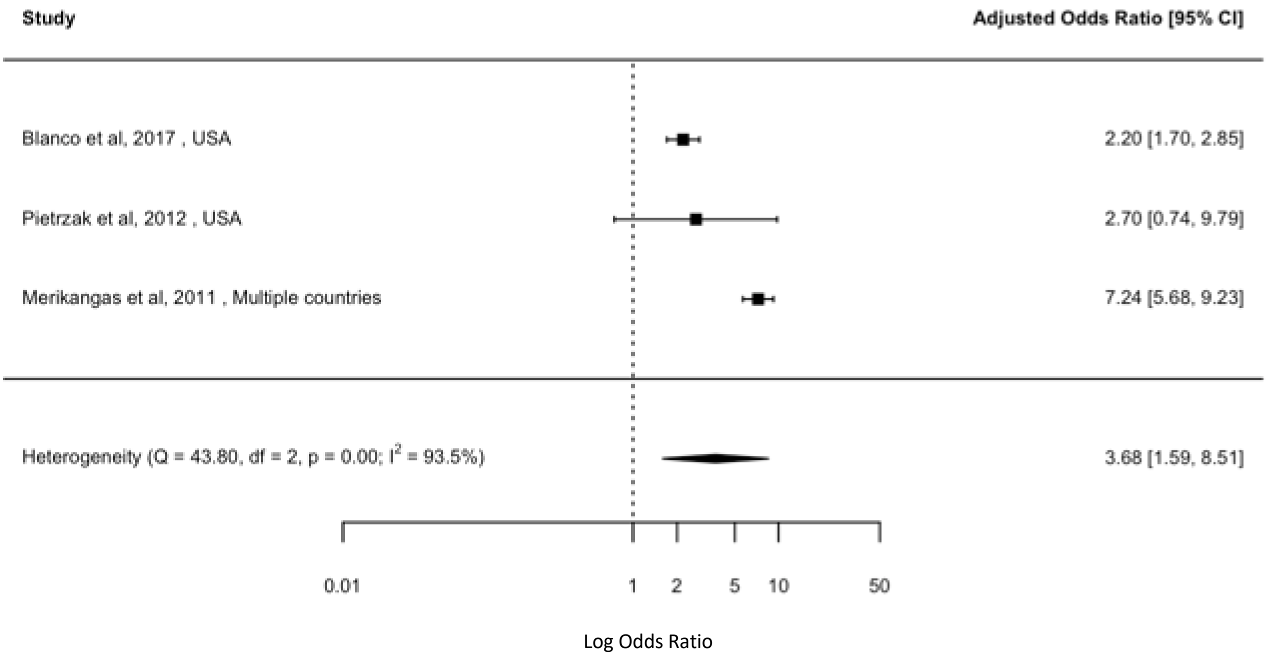

efigure 76 Forest plot of the random-effects meta-analysis of lifetime comorbidity between broadly-defined bipolar disorders and social phobia (unadjusted)

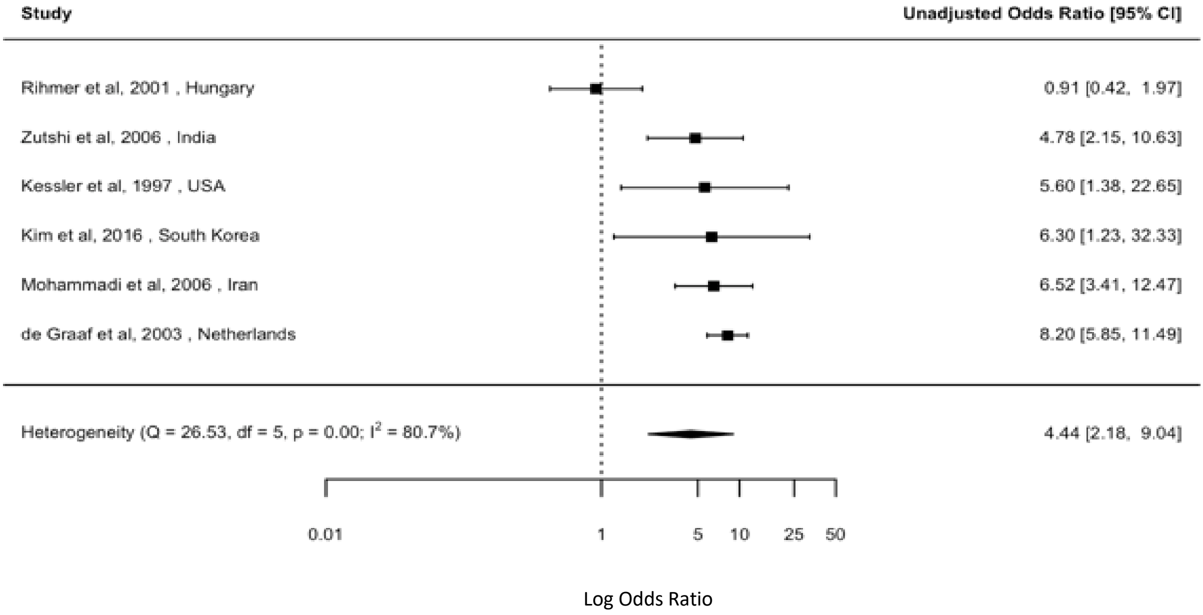

efigure 77 Forest plot of the random-effects meta-analysis of lifetime comorbidity between broadly-defined bipolar disorders and social phobia (adjusted)

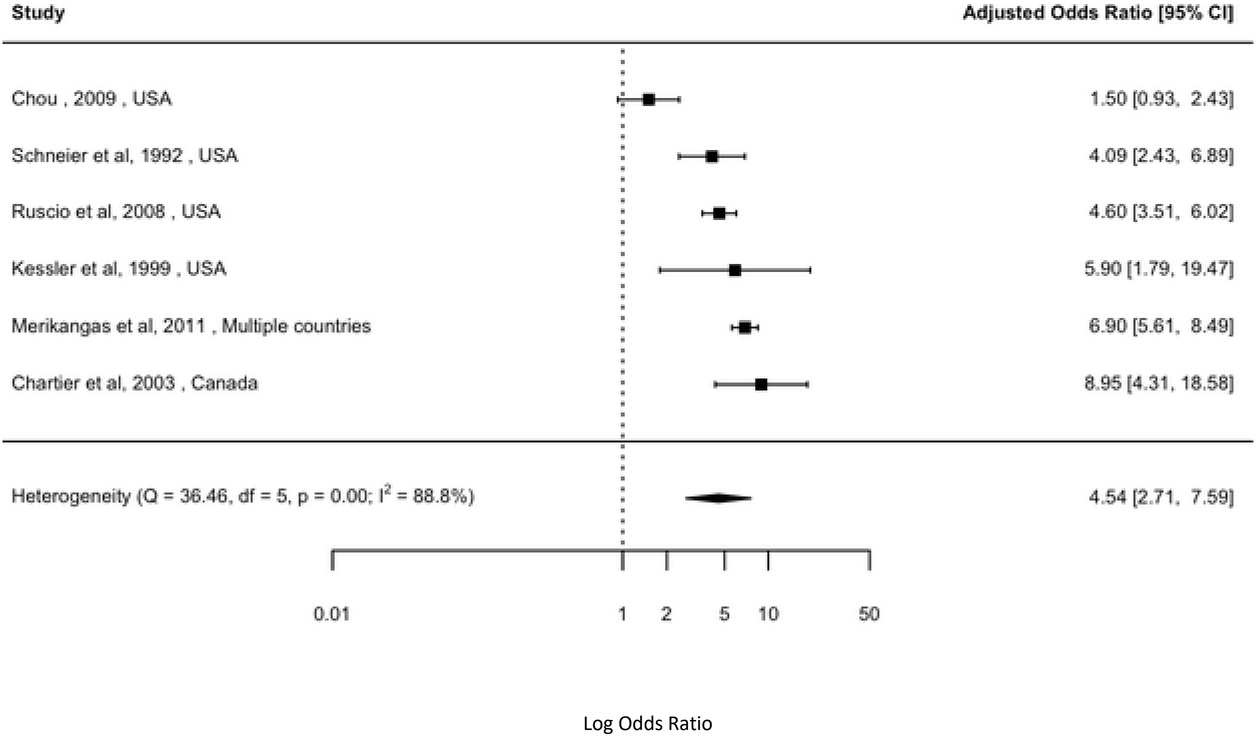

efigure 78 Forest plot of the random-effects meta-analysis of lifetime comorbidity between broadly-defined bipolar disorders and specific phobia (unadjusted)

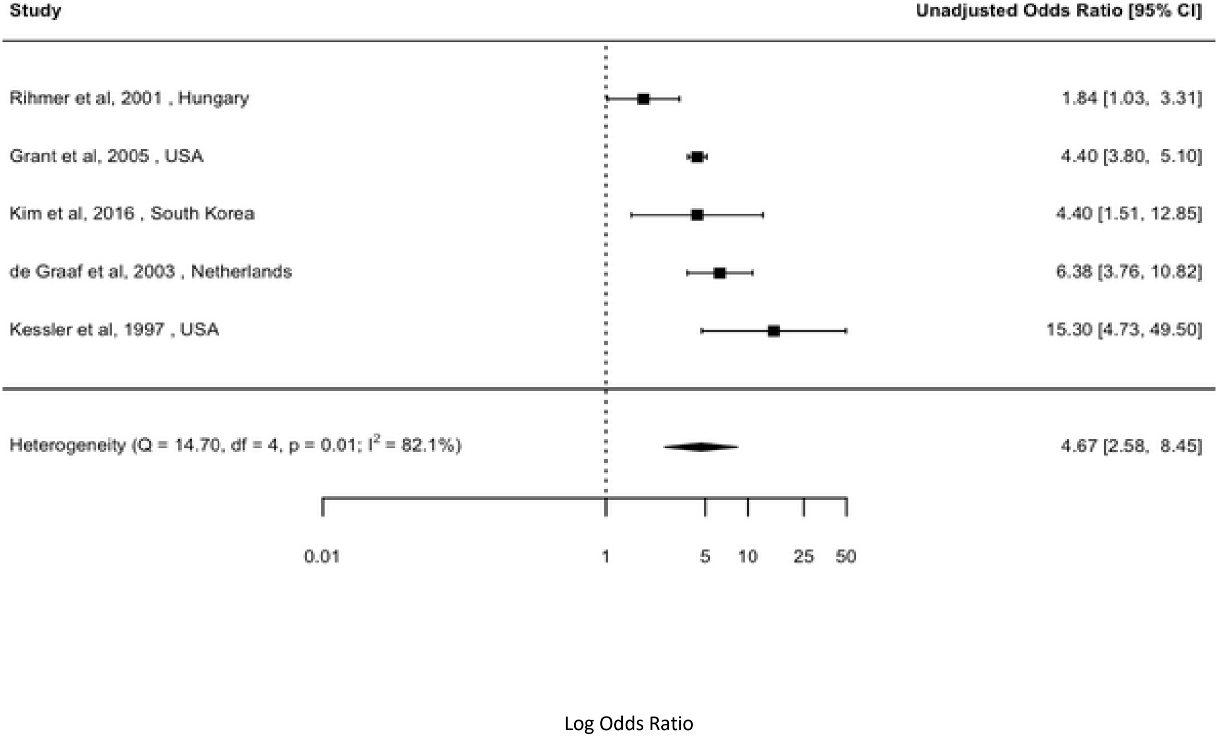

efigure 79 Forest plot of the random-effects meta-analysis of lifetime comorbidity between broadly-defined bipolar disorders and specific phobia (adjusted)

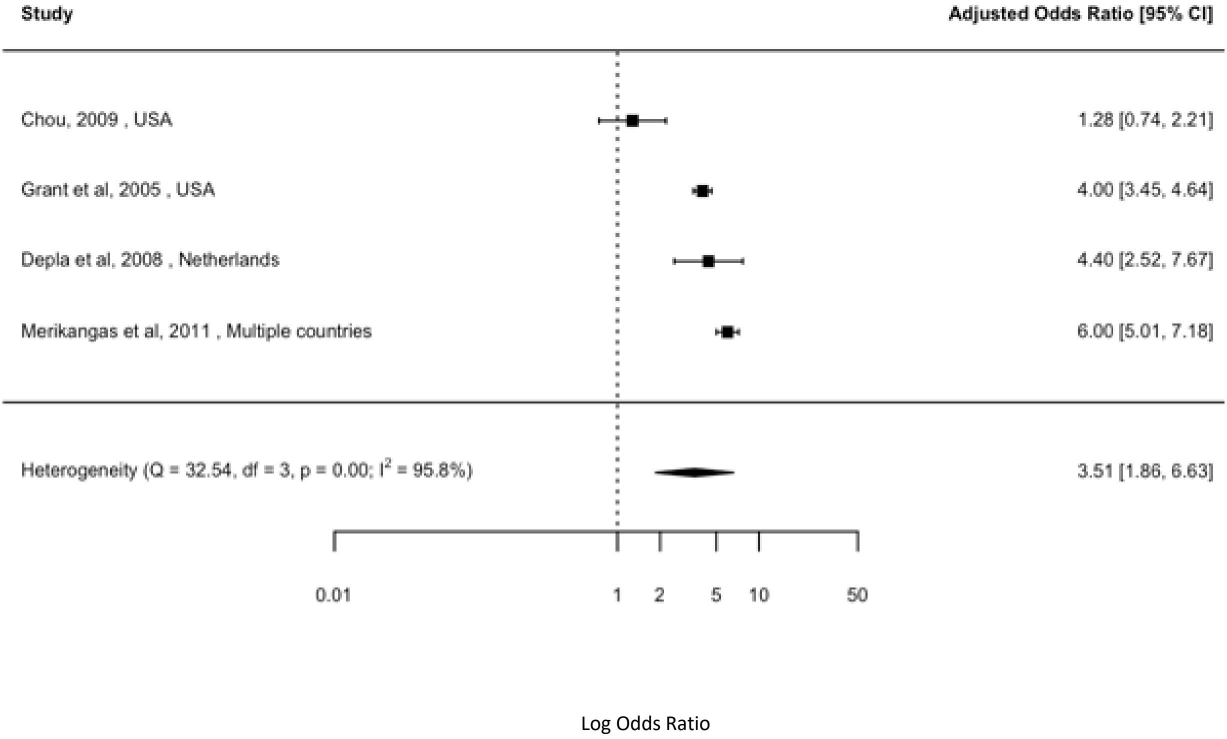

efigure 80 Forest plot of the random-effects meta-analysis of lifetime comorbidity between broadly-defined bipolar disorders and broadly-defined anxiety disorder (unadjusted)

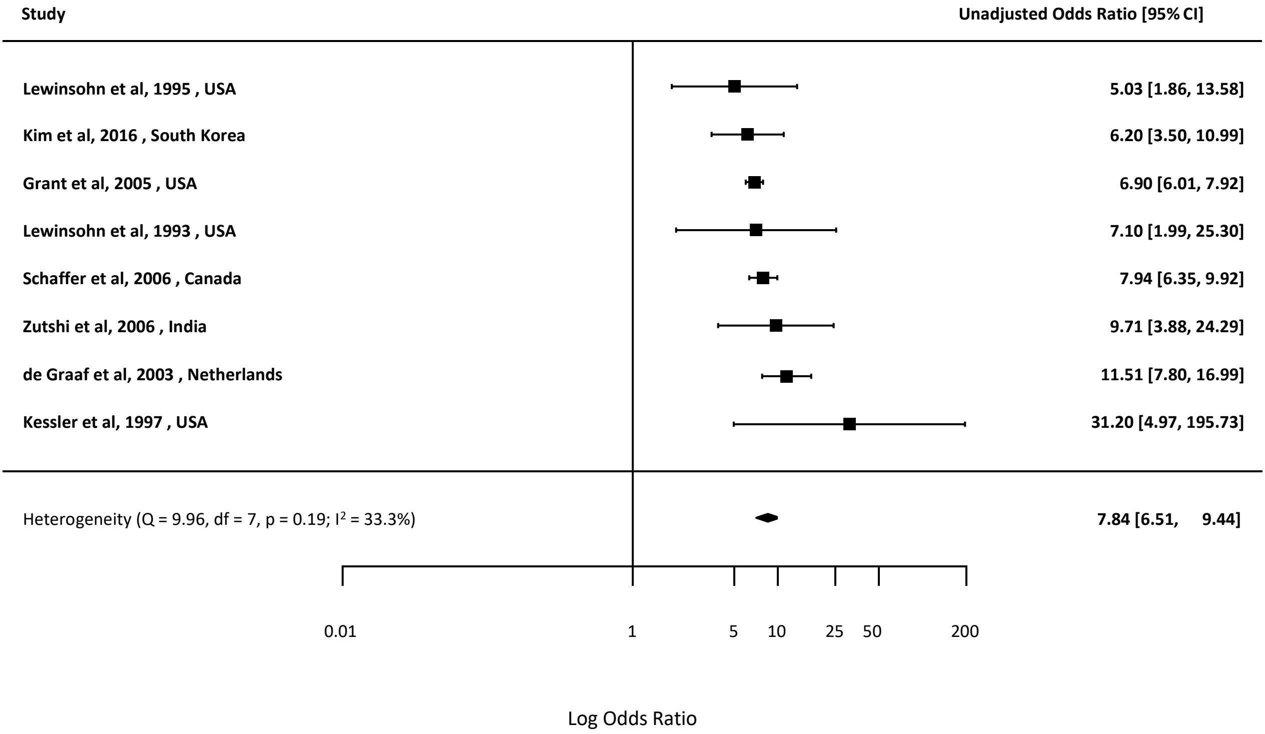

efigure 81 Forest plot of the random-effects meta-analysis of lifetime comorbidity between broadly-defined bipolar disorders and broadly-defined anxiety disorder (adjusted)

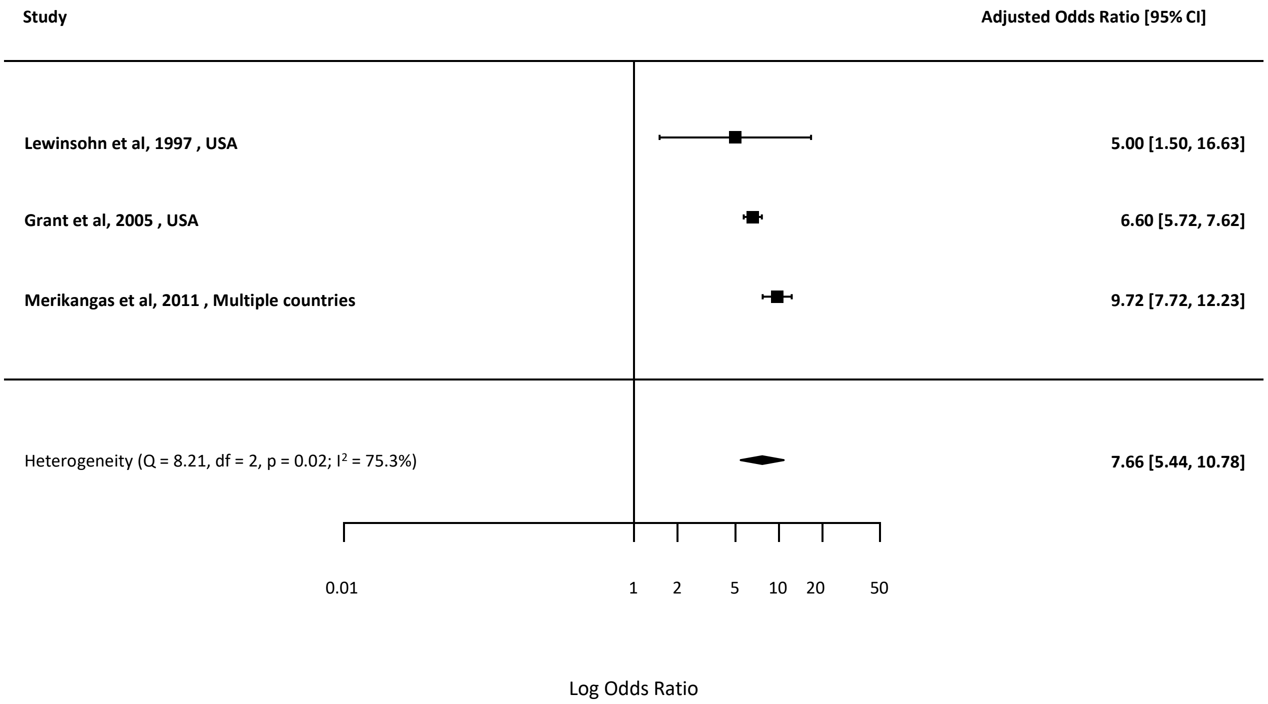

efigure 82 Forest plot of the random-effects meta-analysis of period prevalence comorbidity between broadly-defined bipolar disorders and obsessive compulsive disorder (unadjusted)

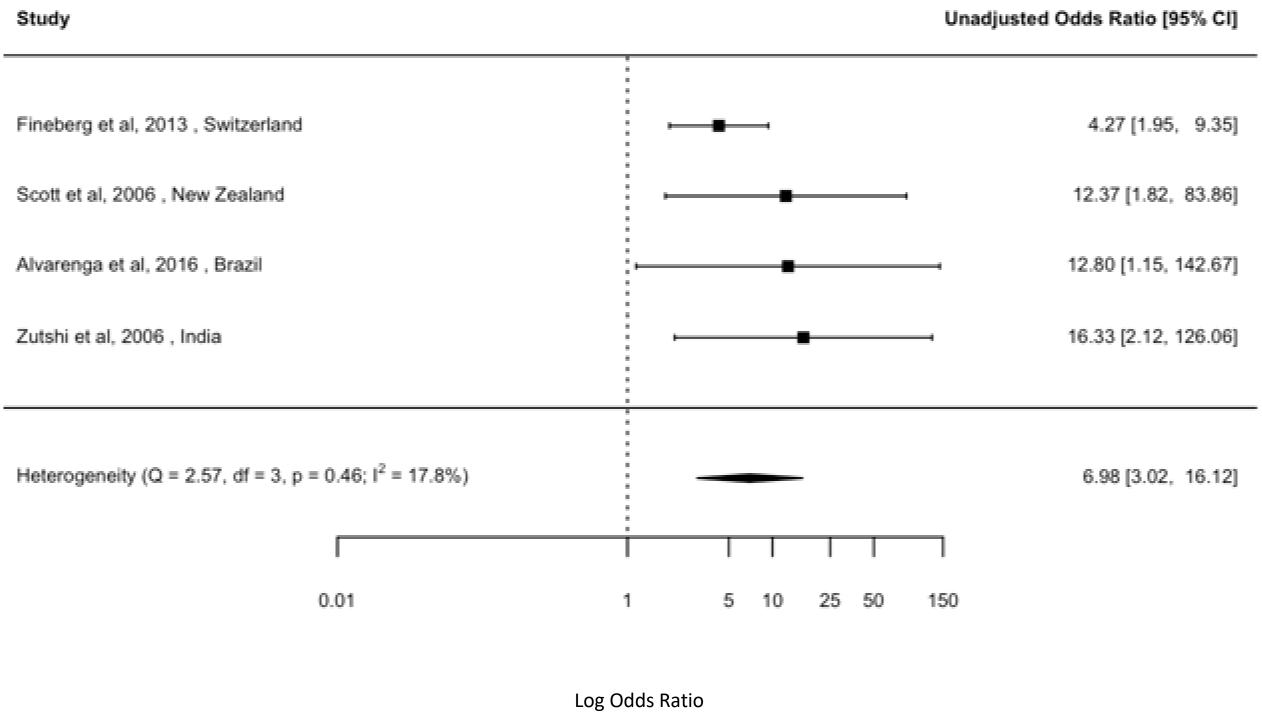

efigure 83 Forest plot of the random-effects meta-analysis of period prevalence comorbidity between broadly-defined bipolar disorders and obsessive compulsive disorder (adjusted)

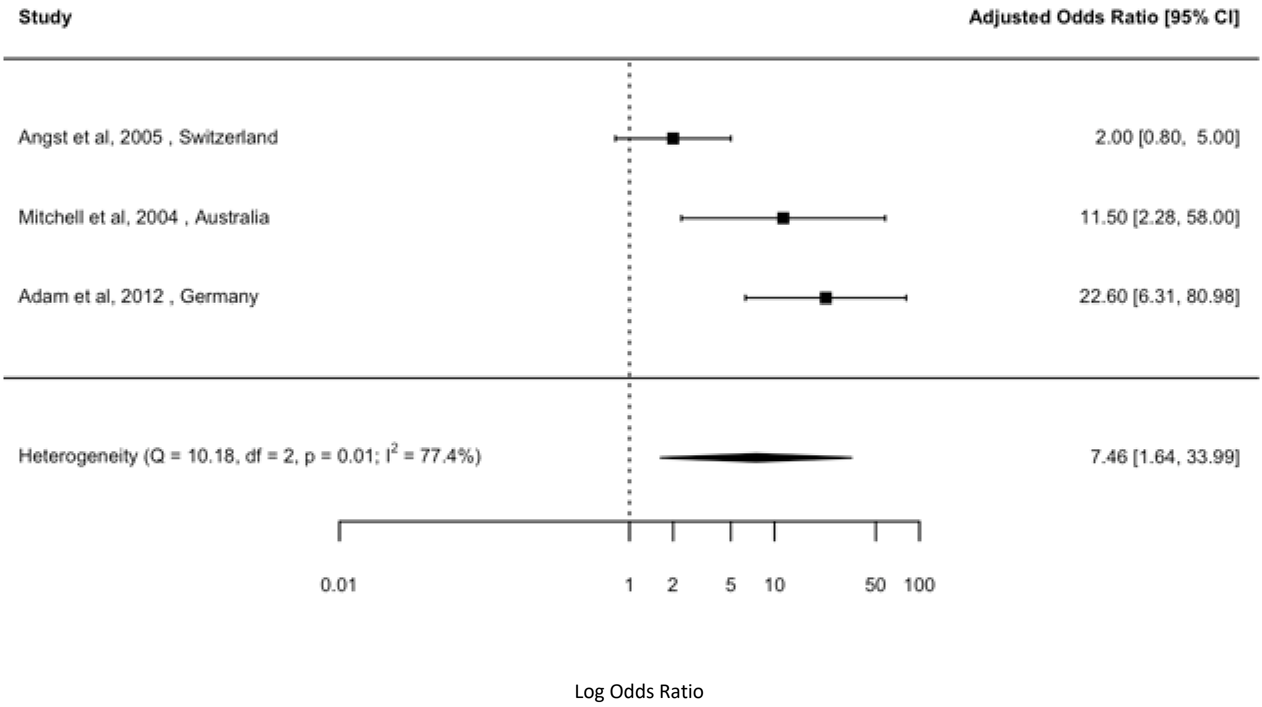

efigure 84 Forest plot of the random-effects meta-analysis of period prevalence comorbidity between broadly-defined bipolar disorders and generalized anxiety disorder (unadjusted)

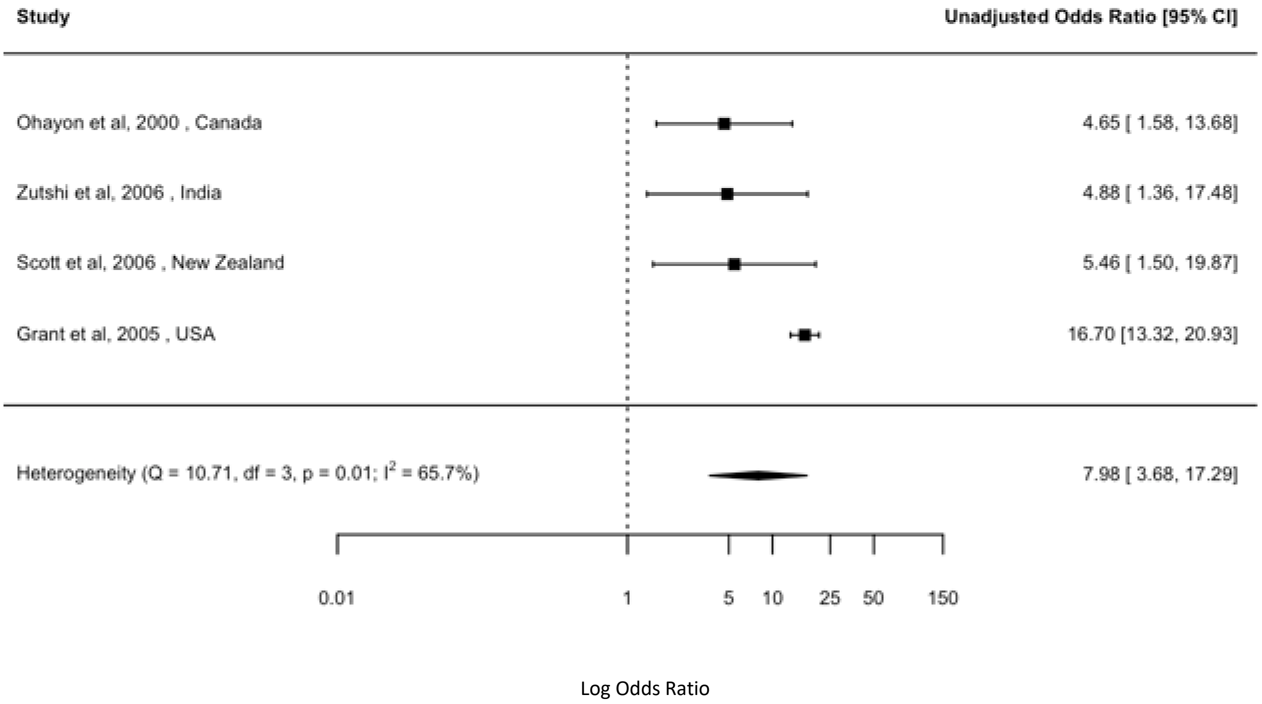

efigure 85 Forest plot of the random-effects meta-analysis of period prevalence comorbidity between broadly-defined bipolar disorders and generalized anxiety disorder (adjusted)

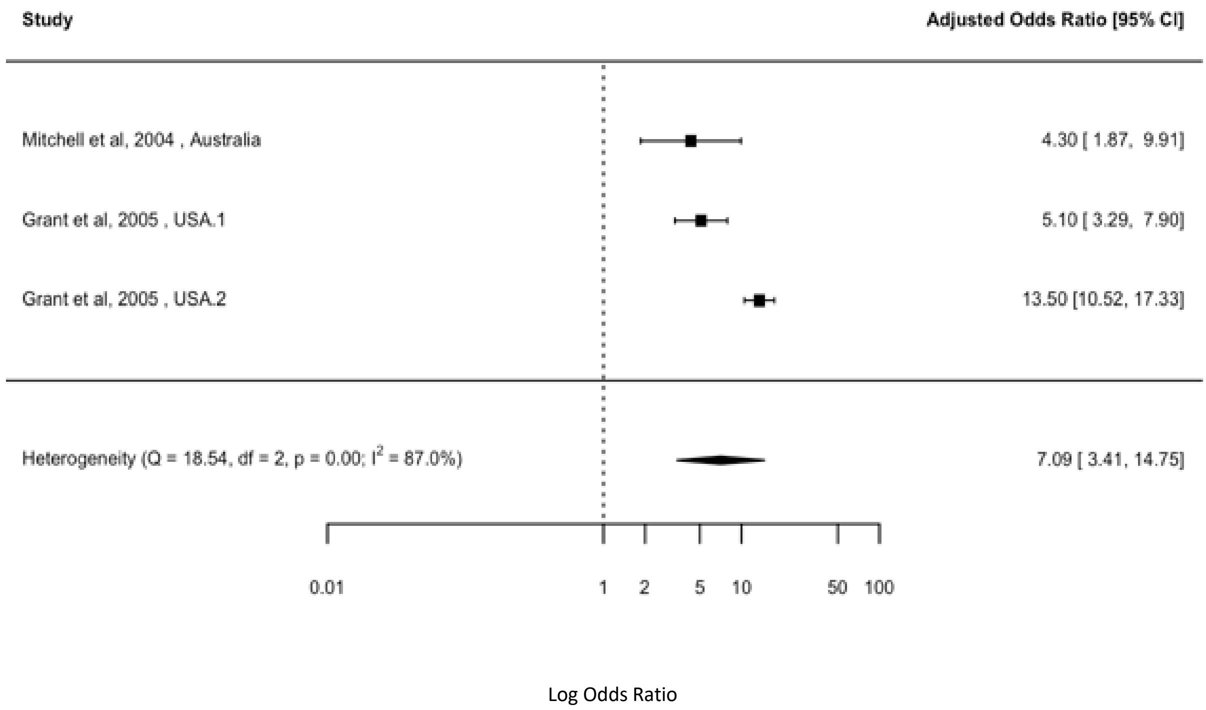

efigure 86 Forest plot of the random-effects meta-analysis of period prevalence comorbidity between broadly-defined bipolar disorders and panic disorder (unadjusted)

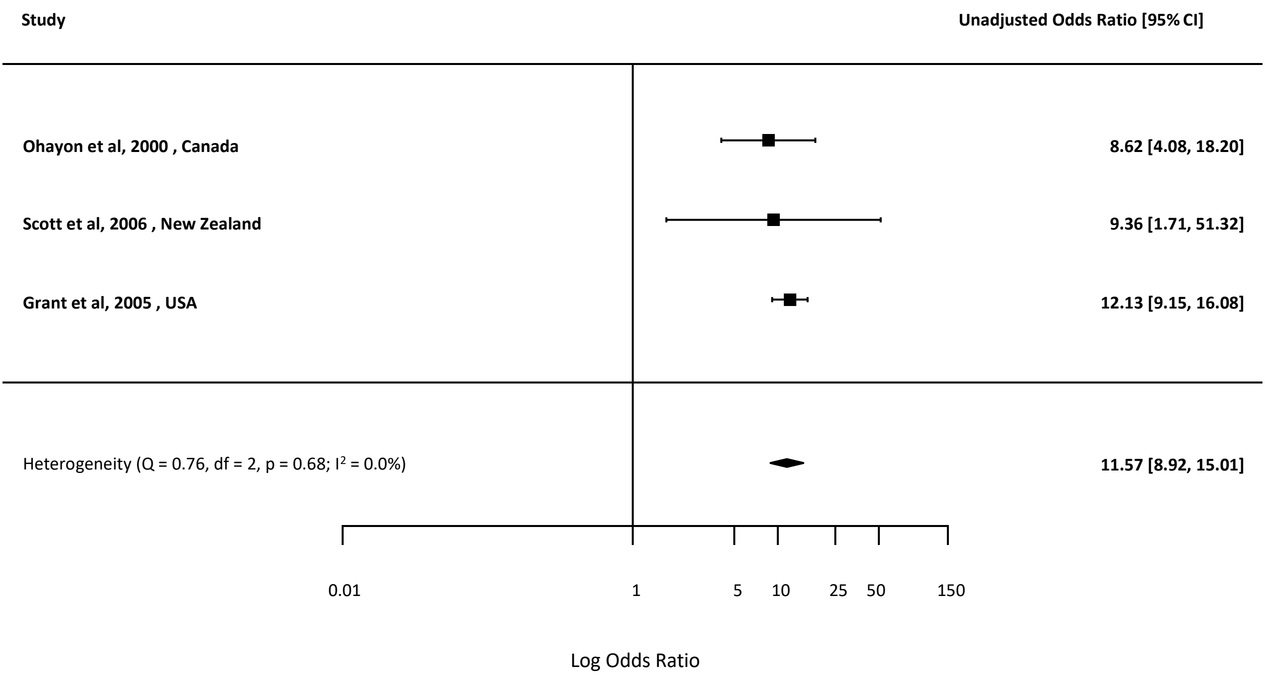

efigure 87 Forest plot of the random-effects meta-analysis of period prevalence comorbidity between broadly-defined bipolar disorders and social phobia (unadjusted)

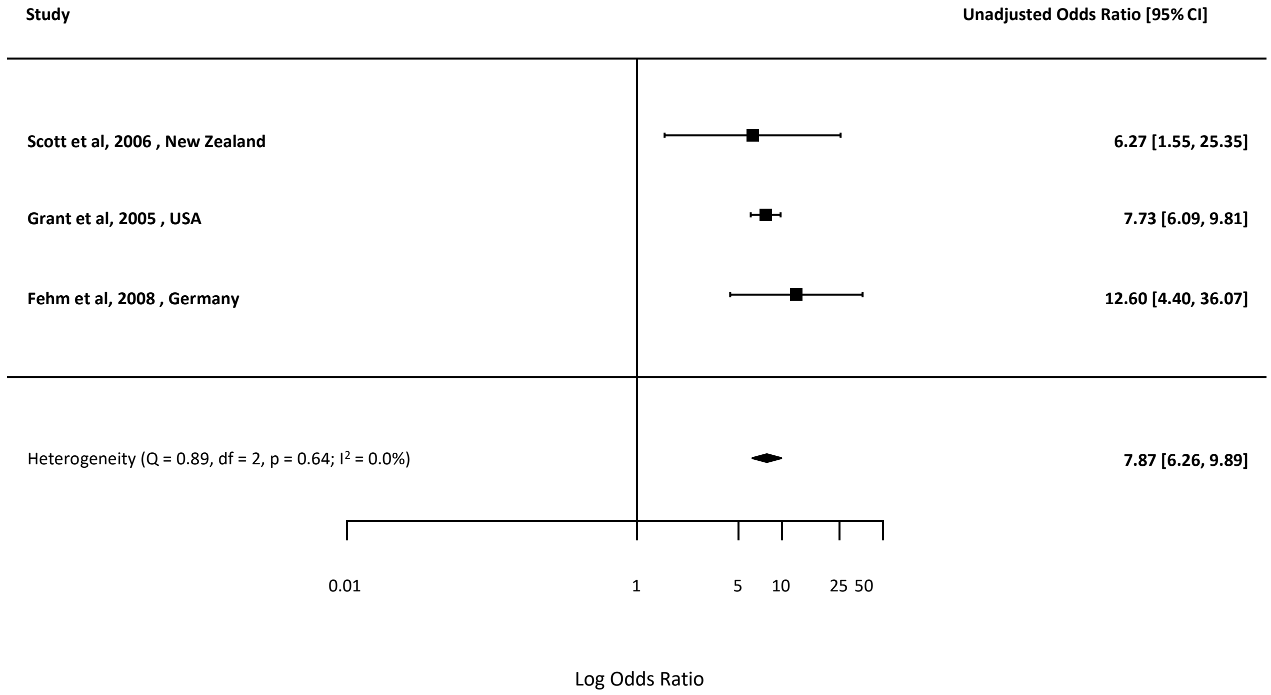

efigure 88 Forest plot of the random-effects meta-analysis of period prevalence comorbidity between broadly-defined bipolar disorders and social phobia (adjusted)

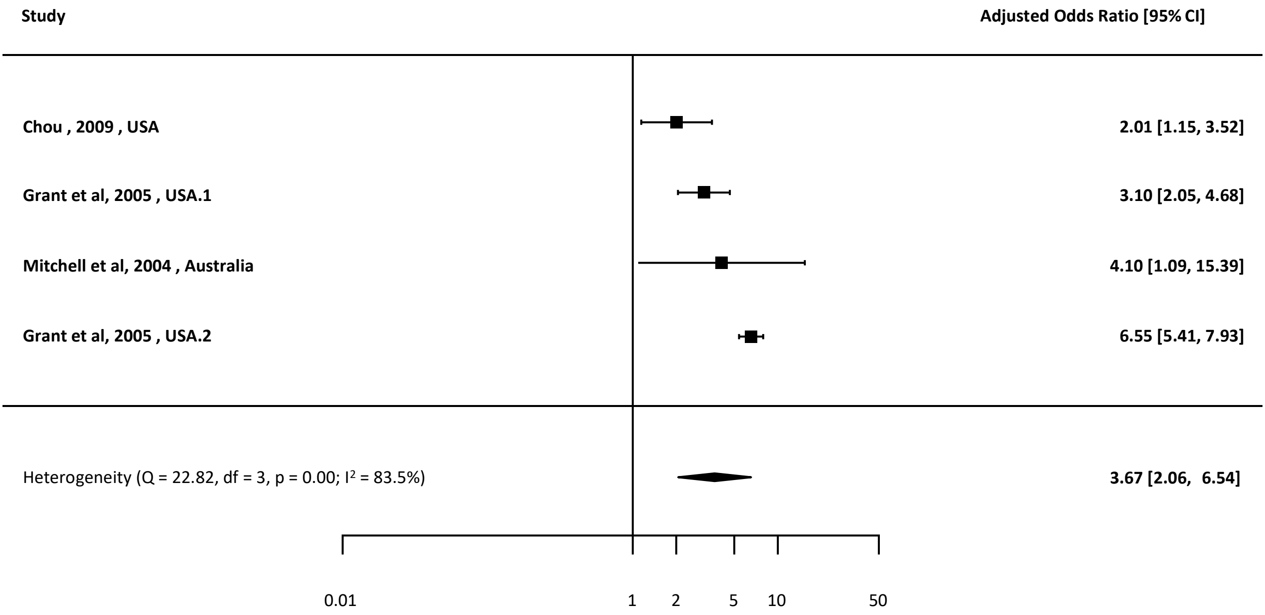

efigure 89 Forest plot of the random-effects meta-analysis of period prevalence comorbidity between broadly-defined bipolar disorders and specific phobia (unadjusted)

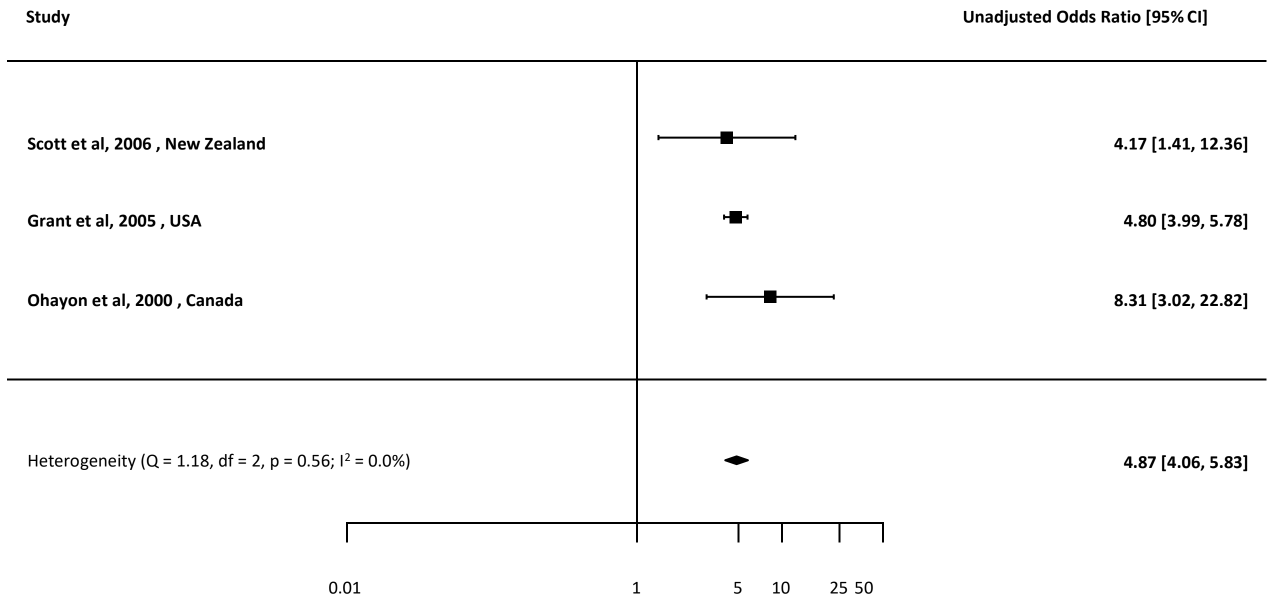

efigure 90 Forest plot of the random-effects meta-analysis of period prevalence comorbidity between broadly-defined bipolar disorders and broadly-defined anxiety disorder (unadjusted)

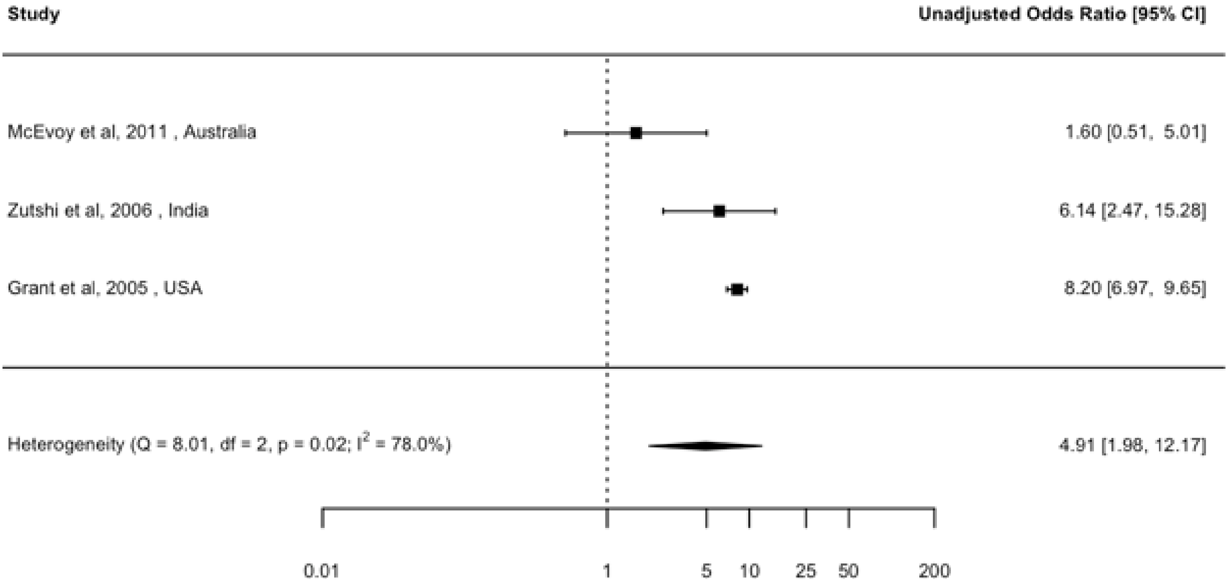

Supplement: Supplementary file 1 — Supporting information. [file DA-38-286-s001.pdf]
